# Comparative metatranscriptomics reveals extracellular electron transfer pathways conferring microbial adaptivity to surface redox potential changes

**DOI:** 10.1038/s41396-018-0238-2

**Published:** 2018-07-26

**Authors:** Shun’ichi Ishii, Shino Suzuki, Aaron Tenney, Kenneth H. Nealson, Orianna Bretschger

**Affiliations:** 1 R&D Center for Submarine Resources, Japan Agency for Marine-Earth Science and Technology (JAMSTEC), Nankoku, Kochi, 783-8502 Japan; 2 Department of Microbial and Environmental Genomics, J. Craig Venter Institute, La Jolla, CA 92037 USA; 3 Kochi Institute for Core Sample Research, JAMSTEC, Nankoku, Kochi, 783-8502 Japan; 4 Project Team for Development of New-generation Research Protocol for Submarine Resources, JAMSTEC, Nankoku, Kochi, 783-8502 Japan; 5 Department of Earth Sciences, University of Southern California, Los Angeles, CA 90089 USA; 6 Aquacycl LLC, San Diego, CA 92121 USA; 7 San Diego State University, San Diego, CA 92182 USA; 8 Present Address: Sigma-Aldrich, St. Louis, MO 63103 USA

## Abstract

Some microbes can capture energy through redox reactions with electron flow to solid-phase electron acceptors, such as metal-oxides or poised electrodes, via extracellular electron transfer (EET). While diverse oxide minerals, exhibiting different surface redox potentials, are widely distributed on Earth, little is known about how microbes sense and use the minerals. Here we show electrochemical, metabolic, and transcriptional responses of EET-active microbial communities established on poised electrodes to changes in the surface redox potentials (as electron acceptors) and surrounding substrates (as electron donors). Combination of genome-centric stimulus-induced metatranscriptomics and metabolic pathway investigation revealed that nine *Geobacter/Pelobacter* microbes performed EET activity differently according to their preferable surface potentials and substrates. While the *Geobacter/Pelobacter* microbes coded numerous numbers of multi-heme *c-*type cytochromes and conductive e-pili, wide variations in gene expression were seen in response to altering surrounding substrates and surface potentials, accelerating EET via poised electrode or limiting EET via an open circuit system. These flexible responses suggest that a wide variety of EET-active microbes utilizing diverse EET mechanisms may work together to provide such EET-active communities with an impressive ability to handle major changes in surface potential and carbon source availability.

## Introduction

Microbial reduction of solid iron- and manganese-oxides is known to occur as a result of microbial EET [1–3]. These electron transfer activities coupled with the oxidation of organic carbon or hydrogen are considered to play important roles within complex microbial communities in soil, sediment, and the deep subsurface [4–6]. The surface redox potentials of solid-phase iron and manganese hydroxides are known to be highly variable because of variations in the crystal structure, pH, salinity, and levels of hydration [7], posing major challenges for EET-capable microbes with regard to adaptation in anaerobic habitats [8, 9]. In fact, the in situ redox potentials of solid-state electron acceptors have been regarded as significant geochemical variables linked to the regulation of functional genes involved with anaerobic electron flow [10]. However, mechanism(s) that microbes use to perform respiration of insoluble metal-oxides via EET are complex and are not yet well understood [11, 12]. In comparison to other environmental parameters such as substrate concentration, the kind of electron acceptor, pH, salinity and temperature, the redox condition is more difficult to control and analyze because the form of solid metals and their redox potentials can change during the process of microbial metal-reduction.

Almost all that is known about the mechanism(s) of EET has been derived from pure culture studies [3, 13], which were conducted by microbes affiliated with the *δ-Proteobacterial* genus *Geobacter* and *γ-Proteobacterial* genus *Shewanella*. A common feature among these EET-capable microbes is the presence of many multi-heme *c-*type cytochromes (MH-cytCs) in their genomes. However, it is still mystery why they possess such a large number of MH-cytCs on their genomes, many of which have no known function. For example, *Geobacter sulfurreducens* PCA, encodes 117 *c*-type cytochromes including 75 MH-cytCs [14]. While molecular biological and physiological studies have shown that at least 16 MH-cytCs play crucial roles in the EET activity [3, 13]; the function of the rest of the MH-cytCs remain elusive. Furthermore, although members of the genus *Geobacter* are known to be abundant in many anaerobic EET-active environments [15], knowledge from pure culture studies cannot be directly applied to describe the ecosystem in terms of the communities, nor predict their cooperative and/or competitive relationships between community members especially under conditions of different redox potentials.

In order to identify metabolic networks within complex EET-active microbial communities, we recently reported a genome-centric stimulus-induced metatranscriptomic analytic scheme by examination of the short-term gene expression responses to specific EET-related stimuli [16]. By using the scheme, quantitative comparison of gene expression profiles can be achieved for each community member respectively, because the samples are analyzed on a time scale that precludes growth-related changes in community composition by the given stimulus. When this approach was used to analyze the response of an electrogenic microbial community on the anode in a wastewater-fed microbial fuel cell, we successfully identified the metabolic network and the shift of the metabolic pathways in response to both a more positive surface redox potential that facilitated EET activity, and an open-circuit condition in which no EET reaction could occur [16]. However, the coulombic efficiency of the wastewater-fed microbial fuel cell was only ~15%, which indicated the presence of other competitive reactions in the system, such as fermentation, methanogenesis and sulfate reduction to digest the diverse organic compounds in the loaded waste water [16]. These results indicate that various noises were included when wastewater was used for addressing the community responses to the surface redox potential alternations.

Here we report a systematic extension of our studies of the responses of mixed microbial communities to changes in surface redox potentials, which was enabled by using multiple EET-active communities previously adapted to three different potentials with sucrose as a sole electron donor and poised electrode as a sole electron acceptor [17]. These communities, which were enriched from sediment samples of a coastal lagoon, contained many phylotypes known to be EET-active, and showed higher coulombic efficiencies (~40%) [17]. These three communities were then three different conditions: (1) changes in electron acceptors (by modifying the potential of the electrodes); (2) changes in electron donors (by modifying the carbon and energy sources); or (3) changes to an open circuit in which no EET was possible. These changes induced major alterations in community physiology as measured by electron flow rates, as well as induction and/or repression of genes in many of the dominant microbes as quantified by our newly-developed genome-centric stimulus-induced metatranscriptomic analysis.

## Methods

### Set-potential bioelectrochemical systems and long-term operating condition

A single-chamber, bioelectrochemical systems (BESs) were used for controlling anode surface redox potential potentiostatically with electric current generation as described previously [17]. The BES was a bottle-type reactor (400 ml in capacity) with anode working electrodes made of carbon clothes (108 cm^2^ projected surface area; TMIL, Tsukuba, Japan) connected by Ti wires to an 8-channel potentiostat (MultEchem System, Gamry, USA) [17]. A Pt-catalyzed air cathode as a counter electrode and an Ag/AgCl reference electrode (+200 mV vs standard hydrogen electrode, SHE, RE-5B, BASi, USA) were placed in the side ports of the BES reactors, and also connected to the potentiostat to control the surface redox potential on the anode electrode. The three sets of duplicate reactors were prepared, filled by carbonate-buffered anaerobic freshwater basal salt medium [17] fed with 3.2 mM sucrose as the sole electron donor, inoculated by 2 g of lagoon sediment slurry, sampled from San Elijo Lagoon (San Diego, CA, USA), gently mixed with a magnetic stirrer, and incubated at 30°C. The anode surface potential was potentiostatically controlled to +100 mV vs SHE (SP-H reactors), −50 mV vs SHE (SP-M reactors), and −200 mV vs SHE (SP-L reactors), and electric current production was recorded using the potentiostat. When the electric current decreased by substrate depletion, the anode solutions were replaced with fresh medium containing 3.2 mM sucrose. Volatile fatty acid (VFA) concentrations were measured using a high-pressure liquid chromatography (HPLC) instrument, while sucrose concentrations were determined by the phenol-sulfuric acid method [17]. This repeat-batch set-potential operation was continued for over 6 months, and the microbial community composition dynamics based on 16S rRNA gene clone analyses have been reported elsewhere [17].

### EET-stimuli for stimulus-induced metatranscriptomic approach

At day 185 of the repeat-batch set-potential (SP) operations, one of the duplicated reactors (reactor a in the previous study [17] was used for applying three different stimuli and for harvesting five different samples (conditions 1–5) (Table 1). After 2 h of operation after medium exchange fed with sucrose, one-fifth (20 cm^2^) of the carbon cloth anode was collected as a standard condition 1 [SP] by ethanol sterilized scissors in an anaerobic glove box, immediately frozen by liquid nitrogen, and stored in a −80 °C freezer.

**Table 1:** Operational conditions for the stimulus-induced metatranscriptomic analysis

Condition^a^	Stimulus	Time after stimulus	Anode potential (mV vs SHE)		Current density (A/m^2^)	Sucrose (mM)	Lactate (mM)	Acetate (mM)	Propionate (mM)
			SP^b^	OC^b^					
SP-H									
con1	SP	2 h	+100	—	2.723	1.85	1.84	1.01	1.26
con2	SP+	45 min	+300	—	2.535	0.85	4.04	10.61	2.41
con3	OC_short_	45 min	—	−276	0	0.02^c^	5.13	10.66	3.36
con4	OC_long_	2 h	—	−298	0	1.97	2.04	1.31	1.12
con5	AcPro	16 h	+100	—	3.707	0.01	0.00	3.66	5.65
SP-M									
con1	SP	2 h	−50	—	2.344	1.85	0.95	0.63	0.77
con2	SP+	45 min	+100	—	2.569	1.53	2.84	8.97	1.68
con3	OC_short_	45 min	—	−306	0	0.67	5.68	10.02	2.41
con4	OC_long_	2 h	—	−317	0	2.12	1.66	0.69	0.50
con5	AcPro	16 h	−50	—	2.976	0.01	0.00	3.04	5.83
SP-L									
con1	SP	3 h	−200	—	0.937	2.21	0.00	2.05	0.47
con2	SP+	45 min	+100	—	2.109	1.22	0.27	12.32	0.85
con3	OC_short_	45 min	—	−318	0	0.55	0.46	12.59	1.27
con4	OC_long_	3 h	—	−319	0	1.62	0.57	2.44	0.52
con5	AcPro	16 h	−200	—	0.769	0.01	0.27	8.62	3.80

^a^Con1-con3 were short-term responses in same batch

^b^SP showed controlled anode surface potentials, while OC showed open-circuit anode potentials

^c^Stimulus for SP-H con3 was OC_short_, but sucrose was depleted by rapid sucrose fermentation, which also worked as a stimulus

After a 20 min recovery period under respective SP operation from the first sampling, the anode potential was controlled to +300 mV vs SHE for SP-H, or +100 mV vs SHE for SP-M/SP-L with supplying 10 mM of acetate (Stimulus SP+). A sample was collected at 45 min after applying the SP + stimulus when current stabilization was observed under the new condition. The same size of anode (20 cm^2^) was collected as condition 2 [SP+] and stored as described above.

After another 20 min recovery phase under original SP operations, the anode working electrodes were disconnected from the counter electrode and operated under open circuit non-poised conditions with zero current production and 0% electron recovery (Stimulus OC). The open circuit anode potential was determined relative to an Ag/AgCl electrode. The third anode sample was collected at 45 min after applying the OC stimulus when anode potential was stabilized under the new condition. A similar size of anode (20 cm^2^) was collected as condition 3 [OC_short_] and stored as described above.

After these three samplings, the reactors were replaced to fresh medium with sucrose, and operated under original SP conditions. After 20–22 h, sucrose and lactate have already been consumed, thereby acetate and propionate were main carbon sources for microbes to conduct EET reaction on anodes (Stimulus AcPro) (Table 1). A similar size of anode (20 cm^2^) was collected as condition 5 [AcPro] and stored as described above.

After another 1 day operation from condition 5 sampling, the reactors were again replaced to fresh medium with sucrose, and operated under open circuit condition (Stimulus OC). After 2 h of the OC operation, a similar size of anode (20 cm^2^) was collected as condition 4 [OC_long_ 2 h] and stored as described above. The conditions 1 and 4 yielded more comparative gene expression profiles by the OC stimulus than the comparison between conditions 1 and 3, because the chemical compositions of organic substrates were similar between them (Table 1).

### DNA and RNA extraction, separation, and sequencing

Both DNA and RNA were coextracted using a MObio PowerBiofilm RNA Isolation Kit (MO BIO, San Diego, CA, USA) with modifications described below [18]. The time for physical disruption to lyse cells by beads beating was modified to 3 min, and we skipped the DNA removal process. The extracted total nucleotides were eluted in nuclease free water, separated using the AllPrep DNA/RNA Mini Kit (Qiagen, Germantown, MD, USA), and the DNA was sheared and applied for paired-end library preparation in fragment insert sizes of approximately 250 nt. Total RNA was treated with Turbo DNA free kit (Thermo Fisher Scientific, Waltham, MA, USA) to completely remove contaminating DNA. The quality of total RNA was evaluated using an Agilent 2100 Bioanalyzer with RNA 6000 Pico reagents and RNA Pico Chips (Agilent Technologies, Santa Clara, CA, USA) according to the manufacturer’s instruction, and we confirmed that the RNA integrity number was greater than 7. The rRNAs were removed by Ribo-Zero rRNA Removal Kit (Bacteria) (Illumina, San Diego, CA, USA), then the RNA samples were used for fragment library construction. The DNAs and RNAs for five conditions at three different communities were both separately sequenced using Illumina HiSeq2000 platform (Illumina, San Diego, CA, USA) as the 101 bp PE for DNA and as the 101 bp fragment for RNA by Illumina’s standard protocol [19]. The DNA and RNA nucleotide sequences have been deposited in the NCBI Short Read Archive under accession number SRR4102211-SRR4102240.

### Assembly of metagenomic data sets

The *de novo* assembly of metagenomic sequences was conducted by CLC de novo Assembly Cell version 4.0 (CLCbio, Boston, MA, USA). All five DNA sequence data for each electrogenic community was mixed, assembled into contigs with scaffolding based on paired information using different kmer sizes (23, 33, 43, 53, and 63) and bubble lengths (100, 400, and 800 bp). The assemblies were compared using total bases of the assembled contigs, N50, and % unmapped raw DNA reads to contigs. From a total of 15 sets for each community, SP-H assembly with kmer size 53, SP-M assembly with kmer size 33, and SP-L assembly with kmer size 23 with bubble length 800 bp were selected. The contigs over 500 bp were used in subsequent analyses. The contigs including scaffolds have been deposited at DDBJ/EMBL/GenBank under Bioproject PRJNA340218 as biosample SAMN05710165 for SP-H, SAMN05712604 for SP-M, and SAMN05712605 for SP-L.

### ORF calling and functional annotation

Estimated taxonomy of contigs was assigned via JCVI prokaryotic metagenomic pipeline [20] as described elsewhere [18]. Open reading fames (ORFs) were called from contigs by MetaGeneMark [21], while rRNAs were identified by the JCVI prokaryotic metagenomic pipeline. For the KEGG orthologous (KO) group assignment [22], KEGG Automatic Annotation Server (KAAS) [23] was used with the SBH (single-directional best hit) method set to 45 as the threshold assignment score. Further KO assignment was conducted by KOG functional annotation in WebMGA server [24], and KO with e-value below 10^−6^ was additionally assigned. Clusters of orthologous groups (COGs) [25] were assigned by COG functional annotation in the WebMGA server [24]. ORFs encoding *c-*type cytochromes were identified based on a CXXCH motif search [18] as for covalent heme binding domain, and ORFs contained more than two occurrences of the motif indicating multi-heme *c-*type cytochromes (MH-cytCs). Conserved protein orthologous groups of *c-*type cytochromes generated from six *Geobacter* genomes were used for assigning to the *c-*type cytochrome family ID [26] and associated gene code from the *G. sulfurreducens* genomic information (NC_002939). ORFs encoding *c-*type cytochrome was assigned to the family ID by the best BLAST hit [27] to the *Geobacter c-*type cytochromes [26] using an e-value cut-off of 1e^−6^.

### Bin-genome clustering

Bin-genomes (draft genomes) were extracted by grouping contigs by using coverage-GC% and differential coverage plots [18, 28]. The contig clusters were then refined by tetra nucleotide frequency method and paired-end connections between contigs [28]. The connection graph was constructed by using Cytoscape v2.8.3. Smoot et al. [29] from the numbers of paired-end connections between contig ends that counted by Collect Paired Read Statistics function in CLC Genome Finishing Module (CLCbio, Boston, MA, USA), which allowed association of additional contigs and repeat regions (for example, rRNA genes) with the correct bin-genome, and removal of contigs wrongly included [28].

To assess the genome “completeness” of bin-genomes, we first used 107 single-copied housekeeping genes for Bacteria and 137 single-copied housekeeping genes for Archaea, and percentage of the genes per genome were used to estimate the genome completeness [18]. To obtain the minimum information about a Metagenome-Assembled Genome (miMAG) proposed by Genomic Standards Consortium (GSC) [30], genome completeness and contamination were also analyzed by using CheckM software [31]. The numbers of tRNA per each bin-genome were counted by using tRNAscan-SE server [32]. The genome quality classification was assigned from miMAG criteria [30].

Ribosomal RNA-associated regions were assigned to phylotypes (clone IDs) by using the BLAST program to link bin-genomes to phylotypes from the 16S rRNA gene clone analyses [17]. If direct linkages were not established, associations were estimated by comparing phylogenetic positions of 16S rRNA gene and three housekeeping proteins (GyrB, RpsG, and TilS) using phylogenetic trees created by the neighbor-joining algorithms in CLC Genomics Workbench version 5.0. All bin-genomes affiliated with similar taxa were compared in a pairwise manner based on average nucleotide identity (ANI) and correlation of the tetranucleotide signatures (TETRA) with the default parameters in JSpecies V1.2.1 [33]. A group of bin-genomes showing both ANIb > 97% and TETRA > 0.99 was defined as the same taxonomic group having species/strain level similarity, and named operational candidate species (OCS) as described elsewhere [34].

### Read mapping of raw reads to ORFs

RPKM values, Reads Per Kilobase per Million mapped reads [35], for both DNA and mRNA samples under five different operational conditions (conditions 1–5) were separately generated by the RNA-Seq Analysis pipeline in CLC Genomics Workbench (version 6.5), and used to analyze ORF frequency (DNA-RPKM) and gene expression levels (mRNA-RPKM). All ORFs for each community were used as references, and read mapping was conducted using 0.6 as the minimum length and 0.95 as the minimum similarity fractions. The calculated mean of the DNA-RPKM for five conditions was used to determine gene existence level of each bin-genome and associated ORFs, while mRNA-RPKM values for each condition were normalized by using the ratio between corresponding DNA-RPKM for each condition and the mean of DNA-RPKM. A weighted multidimensional scale (wMDS) plot was constructed for clustering bin-genomes related to the gene expression dynamics by stimuli application, while a weighted canonical correspondence analysis (wCCA) diagram was constructed for correlating the gene expression dynamics and the given stimuli (Sucrose concentration, OC, EET, and AcPro), both of which used XLSTAT (Addinsoft, New York, NY, USA) and average mRNA-RPKM for each bin-genome. After the RNA raw read mapping against ORFs, rRNA raw reads were further subtracted from the unmapped reads by using BWA [36] and BLAST [27] against SILVA ribosomal RNA gene database [37] as well as identified rRNA sequences from our metagenomic contigs.

### Metagenomic community composition analysis

Relative frequencies of bin-genomes within each community were determined using several different methods. Seventeen single-copied housekeeping genes were selected from the 107 bacterial marker gene sets according to one copy each, and the relative frequencies of each gene set were determined from the mean coverage [16]. The average of the relative frequencies for the marker genes was calculated as “Core genes” community composition. Since several bin-genomes from closely related strains (strain heterogeneity) coded quite similar sequences for those core genes, the bin-genomes were unable to code the seventeen core genes correctly. The “Coverage” basis adjustment was conducted based on average coverage for those bin-genomes. The “Raw reads” community composition was analyzed based on metagenomic raw read frequencies based on DNA raw read mapping to contigs.

### Selection of marker genes for analyzing *Geobacteraceae* metabolisms

EET-related metabolic pathway for *Geobacteraceae* microbes [14] was constructed based on possible electron donors and acceptors including *c-*type cytochrome families [26]. Marker gene families were selected from the metabolic pathway based on top 50 highly expressed KOs (Supplementary Data 4–6) and top 15 highly expressed *c-*type cytochrome families (Supplementary Data 7–9) for further analyzing the metabolic pathway of each bin-genome. The gene expression levels were normalized by dividing the mRNA-RPKM by the DNA-RPKM (mRNA/DNA ratio), and the values were visualized as heatmaps using the MultiExperiment Viewer (MeV) software [38]. Weighted CCA (wCCA) diagrams for each marker gene were constructed based on three operational valuables; SucEET (con1 [SP] and con2 [SP+], performing sucrose-consuming EET reaction), SucOC (con3 [OC_short_] and con4 [OC_long_], under no EET operation) and AcProEET (con 5 [AcPro], performing acetate/propionate-consuming EET reaction). From the midpoint of arrows of each valuable in the wCCA diagrams, the regions associated to the valuable were partitioned, and stimulus-associated genes (related to metabolic pathway) were identified.

## Results

### Three electrogenic microbial communities and given stimuli

**Fig. 1:**
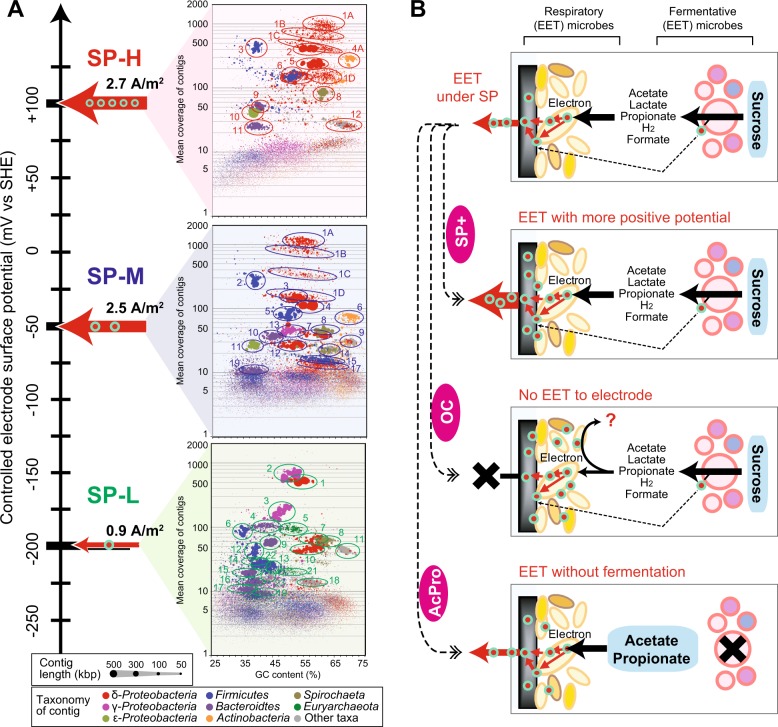
Schematic for identifying extracellular electron transport (EET)-active microbes associated with different surface redox potentials. Panel **a** shows scheme of metagenomics for three EET-active communities adapting to different electrode surface potentials; +100 mV (SP-H), −50 mV (SP-M), or −200 mV (SP-L) vs SHE. The bin-genome clusters (colored circles, ID described near circles) were established using the estimated taxonomic classification of contigs (color of dots), contig lengths (size of dots), GC content of contigs (%), and mean coverage of contig. Panel **b** shows scheme for stimulus-induced metatranscriptomics approach to emerge EET reaction-related microbes and genes. The three stimuli, SP+, OC and AcPro (white letter in purple oval), were applied to the EET-active communities. Fermentative (EET-active) microbes and respiratory EET-active microbes on the electrode (black rectangle) were separately shown in the scheme

Previous work has shown that when different surface redox potentials were used to enrich for electrogenic microbes, the resulting current generating trends and the associated microbial communities and were very different [17]. For the experiments here, we began with the three different electrogenic communities that were enriched using sucrose as the carbon and energy source at three different anode surface potentials (SP-H = + 100 mV; SP-M = −50 mV; SP-L = −200 mV; vs SHE). These potentials were chosen to reflect a range of environmentally available mineral oxides that might be encountered in soils or sediments. The SP-H and SP-M showed similar current generation of ~2.5 A/m^2^, while the SP-L showed remarkably low current generation (0.9 A/m^2^) because of the difficulty of EET respiration under the surface redox condition (Table 1, Fig. 1a). The three communities were then analyzed for community structure by using genome-centric metagenomic analyses [16, 34], which revealed significantly different microbes within the communities (Fig. 1a). Because the community diversities were relatively low, it was possible to construct draft genomes (bin-genomes) for the major species, allowing us to identify genes from the abundant members in each community.

The three different communities (SP-H, SP-M, and SP-L) were then analyzed using stimulus-induced metatranscriptomic approach [16, 18], enabling us to interrogate the individual members of each community with regard to gene regulation at the transcriptional level. For each of these three stable communities, we changed the conditions with regard to surface potentials (electron acceptor) and electron donors for addressing the respiratory electron flow (Fig. 1b, Table 1). The first change was to increase the anodic potential by +100 mV vs SHE (+300 mV vs SHE for SP-H), providing a better electron acceptor resulting in a higher current production (stimulus SP+). The second change was to remove the potential from the electrode by using open circuit operation, creating a condition of no electron flow, and forcing the community into a fermentative mode (stimulus OC). The third change was to replace the sucrose in the growth medium with non-fermentable carbon source—a mixture of acetate and propionate, removing the ability of fermentation, and providing abundant substrates for the respiratory EET-active microbes (stimulus AcPro). In all of these cases, both the current flow (community respiration) associated with substrate concentration were monitored (Table 1, Supplementary Fig. S1), and the metagenomic and metatranscriptomic profiles (Supplementary Table S1-S2) were analyzed and compared for addressing community response to the changes in the surface potentials and/or electron donors.

### Microbial communities adapted to different surface redox conditions

The metagenomic analyses resulted in ~97% of sequence reads assembling into contigs, which were used in clustering to identify draft genomes (bin-genomes) and relative frequencies within communities (Fig. 1a, Supplementary Fig. S2-S6, Table S1). We successfully extracted 15 bin-genomes from SP-H (including pan-genome H1geo), 23 bin-genomes from SP-M (including pan-genome M1geo), and 25 bin-genomes from SP-L, respectively, while microbes coding these bin-genomes occupied ~98% of community compositions (Supplementary Table S3-S5). This result indicates that the surface redox condition affected the community diversity, and that the +100 mV vs SHE minimized microbial diversity the most. Many of the identified bin-genomes were high-quality draft genomes by miMAG criteria (33 bin-genomes from total 63 bin-genome), taxonomically placed, and linked to the previously-reported 16S rRNA gene sequences [17] (Supplementary Fig. S7-S9, Table S3-S11).

**Fig. 2:**
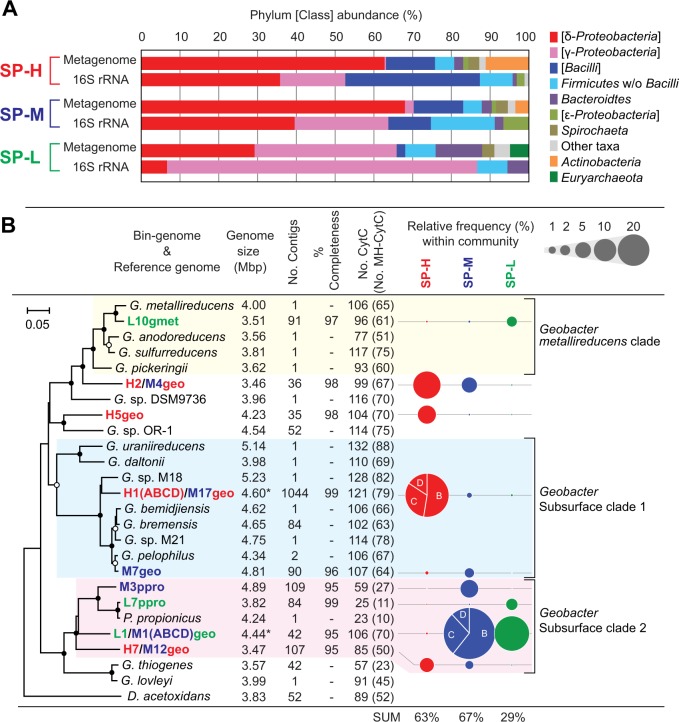
Microbial community compositions and *Geobacteraceae* diversities. Panel **a** shows comparison of microbial community compositions based on Phylum or Class between 16S rRNA gene clone analysis and metagenomic analysis. The metagenomic composition was determined based on averaged coverage for each taxon. Panel **b** shows stats of OCSs affiliated with family *Geobacteraceae* with the phylogenetic position based on DNA gyrase beta subunit (GyrB). Branch points supported with boot-strap values of >99% are indicated with closed circles. Genome completeness (% completeness), number of *c-*type cytochrome (CytC) and multi-heme *c-*type cytochrome (MH-CytC) were shown. An OCS H1(ABCD)/M17geo was represented by the mixture of bin-genomes H1geoA and H1geoB, while an OCS L1/M1(ABCD)geo was represented by only bin-genome L1geo. Relative frequencies of the *Geobacteraceae* bin-genomes within the communities were shown by size of dots. Full lists of relative frequencies of bin-genomes were shown in Supplementary Fig. S7

The metagenomic analyses yielded a more accurate view of the community compositions than had been obtained using 16S rRNA gene-based analyses (Fig. 2a, Supplementary Fig. S10), showing that members of the *δ-Proteobacteria* were numerically dominant under the more electropositive surface redox potentials, SP-H and SP-M. While the SP-M and SP-H communities were similar in relative phylum/class level taxonomic composition, the SP-L community was very different. The SP-L consortia featured a smaller *δ-Proteobacterial* portion, larger *γ-Proteobacterial* portion and the only detected methanogen (L5met) or homo-acetogen (L13fir) working as potential competitors of the EET-active respiratory microbes [39] (Fig. 2a, Supplementary Table S5), which was likely in response to the difficulty of EET respiration under the lower surface redox potential with less electron yield for the current generation [17].

### Geobacter/Pelobacter microbes as dominant community members

The *δ-Proteobacterial* populations in all communities were occupied by the members of the family *Geobacteraceae*, and nine distinct operational candidate species (OCS) that were affiliated to genus *Geobacter* (or *Pelobacter*) (Fig. 2b). The OCS was defined by comparing bin-genomes based on a pairwise manner with high average nucleotide identity (ANIb) values (over 97%) and high correlation of the tetranucleotide (TETRA) signatures (over 0.99) as reported elsewhere [34] (Supplementary Table S12-S13). Four highly abundant *Geobacter* OCSs were found in multiple communities adapted to different redox conditions, and their relative abundances suggest associations to specific redox potentials (Fig. 2b). All of the *Geobacter* OCSs contained large numbers of multi-heme *c-*type cytochromes (MH-cytCs) that are known to be key EET pathway components that mediate electron transport from the inner membrane to the outer membrane for the two well-studied model organisms (*S. oneidensis* and *G. sulfurreducens*) [3].

The most abundant OCS in the SP-H (36%) was represented by a pan-genome H1geo (mixture of three closely related substrains, Supplementary Table S12) affiliated to *Geobacter* Subsurface clade 1. The graph visualization of the H1geoA-D contig connections clearly showed the three substrain-specific contig clusters (H1geoB, C and D) and core contigs (H1geoA): together these substrains define the strain heterogeneity of pan-genome H1geo(pan) (supplementary Fig. S11A). The most abundant OCS in the SP-M (53%) was also represented by pan-genome M1geo (mixture of three closely related substrains, Supplementary Table S12, Fig. S11B) affiliated to *Geobacter* Subsurface clade 2. Interestingly, the most abundant OCS in the SP-L (L1geo, 24%) was same OCS as the dominant OCS M1geo(pan) in the SP-M community (Fig. 2b). However, the bin-genome L1geo did not contain the strain heterogeneity in the SP-L electrogenic biofilm. The second most abundant OCS in the SP-H was H2geo (15%), which was also observed in SP-M (M4geo, 5%). The OCS H2/M4geo was distinct from the known *Geobacter* clade. The second most dominant *Geobacteraceae* OCS in the SP-M was M3ppro (6%), while the second most dominant *Geobacteraceae* OCS in the SP-L was L7ppro (3%), both of which were closely related to *Pelobacter propionicus*, a different genus affiliated to *Geobacter* Subsurface clade 2 [40]. In these two *Pelobacter* OCSs, fewer MH-cytCs were encoded in the genomes (Fig. 2b), which may indicate a significant functional difference from the other *Geobacter* OCSs. Only the OCS L10gmet (2%) in the SP-L was closely affiliated to the model *Geobacter* strain, *G. sulfurreducens*, which has provided the basis of EET mechanistic understanding for *Geobacter* species [3, 13]. However, none of the dominant *Geobacter* OCSs in these three communities was closely related to *G. sulfurreducens*, rendering it impossible to describe their EET mechanistic and responses to the surface redox changes.

### Expression dynamics correlated with different stimuli

**Fig. 3:**
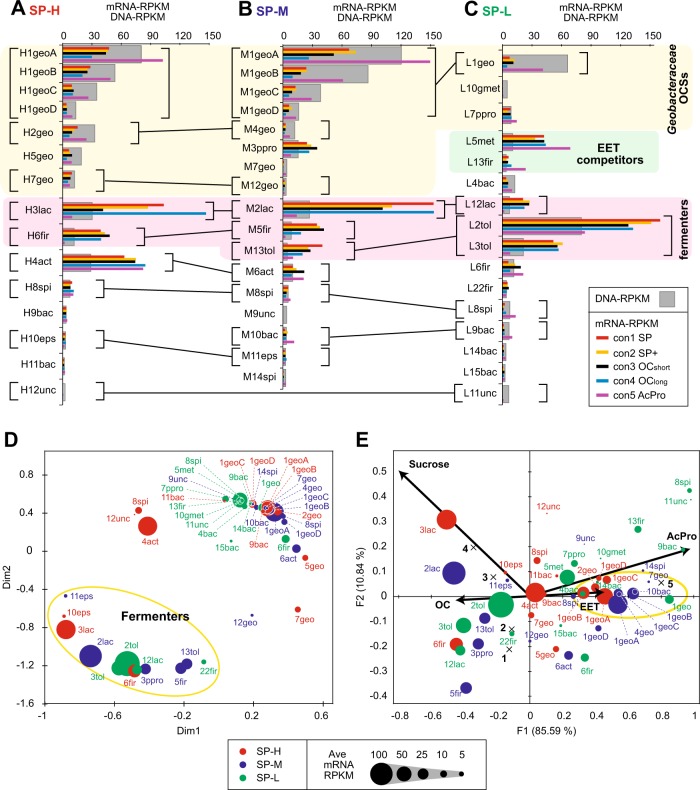
Bin-genome basis gene expression dynamics to identify responses to specific surface potential or substrate stimuli in the electrogenic microbial communities. Relative abundance of DNA and mRNA for bin-genomes within the EET-active biofilms of SP-H (**a**), SP-M (**b**), and SP-L (**c**). The mRNA relative abundance was separately calculated by mRNA-RPKM for five different conditions, while the DNA relative abundance was calculated by normalized DNA-RPKM for all five conditions. Same OCSs within the community or between communities were connected by black bars with brackets. *Geobacteraceae* OCSs, potential EET competitor OCSs and potential fermenter OCSs were highlighted. Panel **d** shows weighted multidimensional scale (MDS) plot, while panel **e** shows weighted canonical correspondence analysis (wCCA) diagram. Size of dots indicates averaged gene expression levels within five conditions, while color of dots indicates community ID. Bin-genome IDs were described near dots. The wCCA diagram showing the relationships between four operational variables (EET, OC, Sucrose, and AcPro as acetate/propionate, red arrows), five operation conditions (cross mark), and bin-genomes of dominant microbes (dots)

**Fig. 4:**
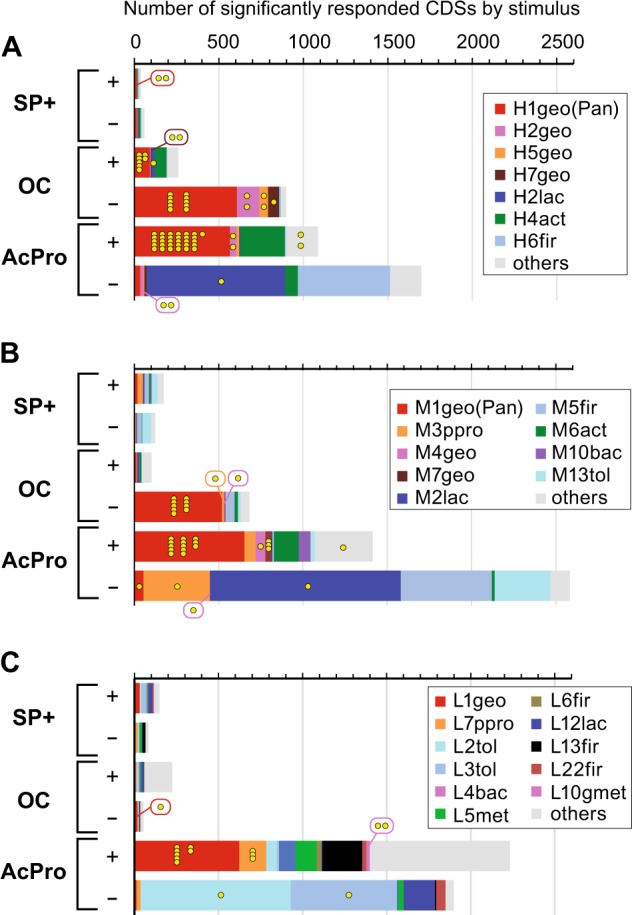
Highly responded CDSs by three EET stimuli for three different communities. CDSs that showed a remarkably positive (+) or negative (–) response (5 times change and mRNA-RPKM after/before stimulus over 50) to the given three different stimuli (SP+, OC, or AcPro) are summed relative to taxonomic assignment (color in right boxes) for three different EET-active communities; panel **a** for SP-H, panel **b** for SP-M, and panel **c** for SP-L. MH-cytCs are shown in yellow dots

Comprehensive gene expression profiles of each community were characterized as a function of the three different stimuli (SP+, OC, and AcPro, Fig. 1b) to address EET mechanisms, carbon metabolisms and surface potential adaptation within microbes (Figs. 3 and 4, Supplementary Fig. S12-S16, Table S2, S14-S16). Global expression changes of the dominant bin-genomes were compared to understand how each stimulus had affected to the dominant members in the communities (Fig. 3a–c). A weighted multidimensional scale (wMDS) plot (Fig. 3d) and a weighted canonical correspondence analysis (wCCA) diagram (Fig. 3e) were generated from the gene expression trends in order to cluster for associating each OCS with specific metabolic function. These data clearly indicated that *Tolumonas*, *Lactococcus* and *Firmicutes* OCSs and *Pelobacter* OCS M3ppro were functioning as sucrose fermenters and/or were positively responding to the stimulus OC, while almost all of *Geobacter/Pelobacter* OCSs were functioning as EET-active microbes and/or reacting positively to the stimulus AcPro.

The highly responsive genes (over 5 times change and mRNA-RPKM over 50) by the stimulus application were then analyzed for the coding sequences, with particular attention being paid to the genes coding MH-cytCs known to be involved in EET (Fig. 4, Additional Data 1-3). It is noteworthy that application of the EET-enhancing stimulus (SP+), while producing a physiological response of enhanced current flow in SP-M and SP-L, resulted in only a minor response at the level of gene expression (Fig. 4a). Of around 100,000 genes in each community, no more than 175 genes were either positively or negatively stimulated. The SP-H showed the least response, likely because it was already growing via rapid respiration, and many of the key genes related to this function were already induced. Only H1geo(pan) showed positively responsive genes that are all coding tetra-heme MH-cytC, *cytT* (cytochrome c554, cytC family 2686, or pfam 13435), suggesting that the MH-cytC was necessary to adapt to the more positive surface redox potential (Supplementary Fig. S12). The SP-L also showed little response after SP + stimuli with 2 times more EET reaction (Table 1), not surprisingly, as the community composition may well be adapted to a fermentative life style, and thus a longer SP + stimulus may be required to induce significant changes to gene expression, community composition and function. Of note, two of the SP-L members, a hydrogenotrophic methanogen (L5met closely affiliated with *Methanocorpusculum bavaricum* [41]) and a homo-acetogen (L13fir closely affiliated with *Acetobacterium submarinus* [42]) capable of growth with CO_2_ as another electron sink except for the anode electrode (Supplementary Fig. S16, S17), exhibited a strong negative response to the SP + stimulus: they clearly preferred the lower redox potential, and were likely competitors of EET respiration under −200 mV vs SHE condition (Fig. 4a).

In contrast, the OC stimulus condition had a much greater impact on all three communities (Fig. 4b). For example, when electron flow was stopped for the SP-H and SP-M communities, 100–250 genes were positively stimulated, while 700–900 genes (mostly relating to the H1geo(pan) and M1geo(pan) including many MH-cytCs) were negatively stimulated (Supplementary Fig. S15). The SP-L showed the least response (only 54 genes were negatively stimulated), likely because the community was already adapted to a non-respiratory lifestyle as described above. These results imply that the more active electrogenic communities (SP-H and SP-M) were shifting their metabolism to another mode in response to the OC stimulus, by downregulating many of the key genes involved in EET. The remarkable upregulation of MH-cytC genes in response to the OC stimulus was revealed in only several *Geobacter* OCSs of the SP-H (Fig. 4b), which suggests that these OCSs managed the lack of solid-phase electron acceptor via the induction of the other MH-cytCs (Supplementary Fig. S18).

Application of the AcPro stimulus to eliminate community fermentation had a very significant effect on all three communities, with more than 1000 genes being positively or negatively stimulated (Fig. 4c). The positively stimulated group included many MH-CytCs, and mainly correlated to the dominant *Geobacter* OCSs for each community, indicating that a greater availability of these non-fermentable fatty acids as carbon sources enhanced the EET process [15]. In marked contrast, a strong negative response after OC stimulus was noted in several of the fermentative *Lactococcus*, *Tolumonas* and *Firmicutes* OCSs. These included a *Lactococcus* OCS (H2lac/M2lac/L12lac) that was a dominant member at more positive surface potentials, and three *Tolumonas* OCSs (L2tol/M18tol, L3tol, and M13tol/L20tol), which were more prevalent under negative surface potentials. As expected, the highly downregulated genes by the AcPro stimulus for the fermenters included those encoding sugar transporters, key genes for glycolysis and translation-related ribosomal proteins, supporting the notion that their metabolic role is sucrose fermentation (Supplementary Table S17, Additional Data 1-3). One *Firmicutes* OCS (H6fir/M5fir closely affiliated to a ferric iron-reducing fermenter *Anaeroarcus burkinabensis* [43]) was also negatively stimulated under the AcPro condition (Fig. 4c), indicating the association with the sucrose fermentation process. However, the gene expression dynamics indicated that the OCS did not perform sucrose fermentation directly (Supplementary Table S17), suggesting that this OCS consumed lactate but not acetate/propionate. The *Pelobacter* OCS M3ppro exhibited a negative response to the AcPro stimulus, which was similar to that seen in the sucrose fermenter OCSs (Fig. 4c). This trend also suggests a different role of the *Pelobacter* OCS from the other *Geobacter* OCSs.

### Stimulus-induced expression changes of *Geobacter/Pelobacter* OCSs

**Fig. 5:**
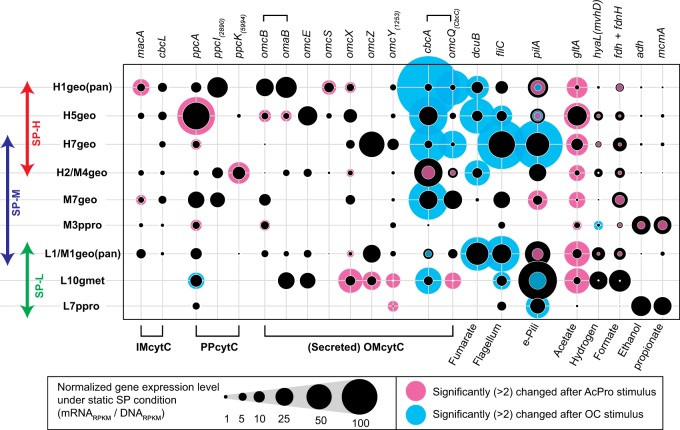
Gene expression trends of key metabolic marker genes for nine *Geobacteraceae* OCSs. Gene expression levels and dynamics of key marker genes related to EET metabolisms within nine EET-active *Geobacter/Pelobacter* OCSs that were shown in different SP conditions (double-headed arrows). The selected marker genes encode MH-cytCs, flagellin, e-pili, fumarate transporter, and enzymes related to consumption of different substrates. Normalized gene expression levels (mRNA_RPKM_ / DNA_RPKM_) under the basic SP operational condition are shown by the size of black circles, while only gene families that had a fold-change (>2) are shown by the background pink circles (AcPro stimulus) or blue circles (OC stimulus)

To further investigate the respiratory strategies of the nine *Geobacter/Pelobacter* OCSs with regard to adaption to different surface redox conditions, we summarized the gene expression trends of key marker genes selected from the potential metabolic map of *Geobacter/Pelobacter* microbes (Supplementary Fig. S19). The key marker gene sets include 5 metabolic genes, 3 appendage/transporter genes, and 14 MH-cytC genes (Fig. 5). The full list of the marker gene sets in the metabolic map and their gene expression trends are also shown in Additional Data 4–9.

The metabolic problem that must be solved for EET process to occur in Gram-negative bacteria is the movement of electrons from inner membrane (IM), across the periplasm (PP), to and across the outer membrane (OM), which is accomplished through the activity of a number of MH-cytCs [3]. To this end, we selected a subset of 14 MH-cytCs as key EET genes (Fig. 5) from 173 known CytC families [26] (Supplementary Table S18), which showed large gene expressions for the nine *Geobacter/Pelobacter* OCSs in the enriched electrogenic communities (Additional Data 7-9). These 14 proteins included most of the essential MH-cytCs for EET metabolism in *G. sulfurreducens* [44–50]. An analysis of expression patterns of these key MH-cytCs to the three applied stimuli yielded surprisingly diverse expression responses (Fig. 5), suggesting that the various MH-cytCs may be performing different functions even within the same taxon.

Expression profiles suggested that IM-cytC genes *macA* and *cbcL* are important electron carriers under all conditions; with *cbcL* being favored under more positive surface potentials (Fig. 5). Examination of the PP-cytC genes indicated that cytochrome c3, *ppcA*, was conserved in all OCSs as an electron carrier. However, the expression patterns suggest that the uncharacterized cytochrome c3 genes, *ppcI* and *ppcK*, were more important electron carriers than *ppcA*, as is seen for more positive surface redox-favoring OCSs H1geo and H2/M4geo. Although the gene expression levels of IM- and PP-cytCs indicated their redox preferences, significant gene expression changes for these genes were not induced by either the SP+ or the OC stimulus, except for the *ppcA* gene in OCS L10gmet (Fig. 5). On the other hand, the MH-cytCs had greater differential gene expression trends under the AcPro stimulus showing higher expression levels for *macA*, *ppcA* and *ppcK* for those *Geobacter/Pelobacter* OCSs associated with the higher redox potential (SP-M and SP-H) communities.

The gene expression profiles of OM-cytCs were also quite diverse, with the poorly characterized *omcX*, *omcY*, and *cbcA*-*omcQ* genes being highly expressed by all OCSs; while the well-characterized *omcS* [48], *omcB*-*omaB* type gene cluster [46], *omcE* [48] and *omcZ* [45] showed OCS-specific expressions (Fig. 5). It was not possible to define any clear redox potential preferences for any of the OM-cytC genes given the overall expression levels. However, the gene expression dynamics of the OM-cytCs showed that the AcPro stimulus had the most impact on upregulation of *omcB*/*omaB*, *omcS*, and *omcX* genes associated with the OCSs in the SP-H and SP-M (Fig. 5). Significant upregulation of the *omcX*, *omcZ*, *omcY*, and *omcQ* genes was also observed for the OCS L10gmet in the SP-L. These trends suggest that those six OM-cytCs were the important electron carriers for performing EET especially under respiratory conditions. In contrast, the OCS H2/M4geo showed a significant downregulation of the *cbcA-omcQ* gene cluster in response to the AcPro condition, while the other *Geobacter* OCSs showed strong responses of the *cbcA-omcQ* gene cluster to the OC stimulus that stopped EET reactions; however, the dynamics (up-regulation, down-regulation, or no change) were different in each OCS (Fig. 5). Two OCSs H1geo and H7geo showed strong up-regulation (>10) of both the *cbcA-omcQ* genes in response to the OC stimulus, although almost 75% of coding sequences (CDSs) were downregulated in response to the OC stimulus for the OCSs (Supplementary Fig. S15). The *cbcA* gene alone showed more dynamic responses across multiple OCSs from each redox enrichment including H5geo, M7geo, and L10gmet. These observations suggest that the same OM-MH-cytC family proteins may be decidedly different in their functional roles depending on environmental conditions.

The OC condition also induced a strong upregulation of the fumarate transporter gene (*dcuB*) and the flagellin gene (*fliC*) for several *Geobacter* OCSs (Fig. 5). The *dcuB* gene encodes a key protein for anaerobic fumarate reduction in *G. sulfurreducens* [51], and the gene expression comparison between electrode-respiring and fumarate-reducing conditions also showed massive upregulation of the gene under the fumarate-reducing condition [49]. The *fliC* gene encodes a principal component of the flagellum [52], while the gene expression level of *fliC* gene was usually most abundant within the flagellum complex, and was related to the swimming activity for bacteria [53]. These gene expression trends indicate that *Geobacter* OCSs rapidly sensed and responded to the OC stimulus; and that the responses could be related to various strategies for seeking alternate electron acceptors, e.g., swimming away from the non-conducting electrode, transporting fumarate, or inducing different OM-MH-cytCs. All of these OC responses showed greater gene expression changes than the responses to the AcPro stimulus, which indicates that the limitation of the EET respiration was an emergency condition for the *Geobacter* OCSs, requiring to adapt rapidly to the new condition.

### Stimulus-induced expression changes of conductive pili

In addition to the MH-cytCs, electrically conductive pili (e-Pili) have been proposed as an important element for long distance electron transfer in the thick electrogenic biofilm, especially for model *G. sulfurreducens* [54]. The e-pili are composed of type IV pilus monomers that synthesized by the product of the *pilA* gene, and are proposed to provide metallic-type conductivity [55]. The gene expression profiles of *pilA* genes in the *Geobacter/Pelobacter* OCSs showed relatively high gene expression under the static SP conditions, especially in more negative surface redox-favoring three OCSs (Fig. 5). In addition, the gene expression responses (upregulation, downregulation, or no change) to the given two stimuli were quite different in each OCS, similar to the situation seen with the *cbcA-omcQ* gene cluster (Fig. 5). Two OCSs H7geo and L7ppro showed upregulation in response to the OC stimulus, while three OCSs H1geo(pan), H5geo, and L10gmet showed down-regulation in response to the same OC stimulus. As for AcPro stimulus, only M7geo showed upregulation, and three OCSs H1geo(pan), H5geo and L1/M1geo(pan) showed down-regulation. These results suggest that the functionality of e-pili for each OCS may differ, which may indicate different functional roles for the OCSs within the electrogenic microbial communities.

**Fig. 6:**
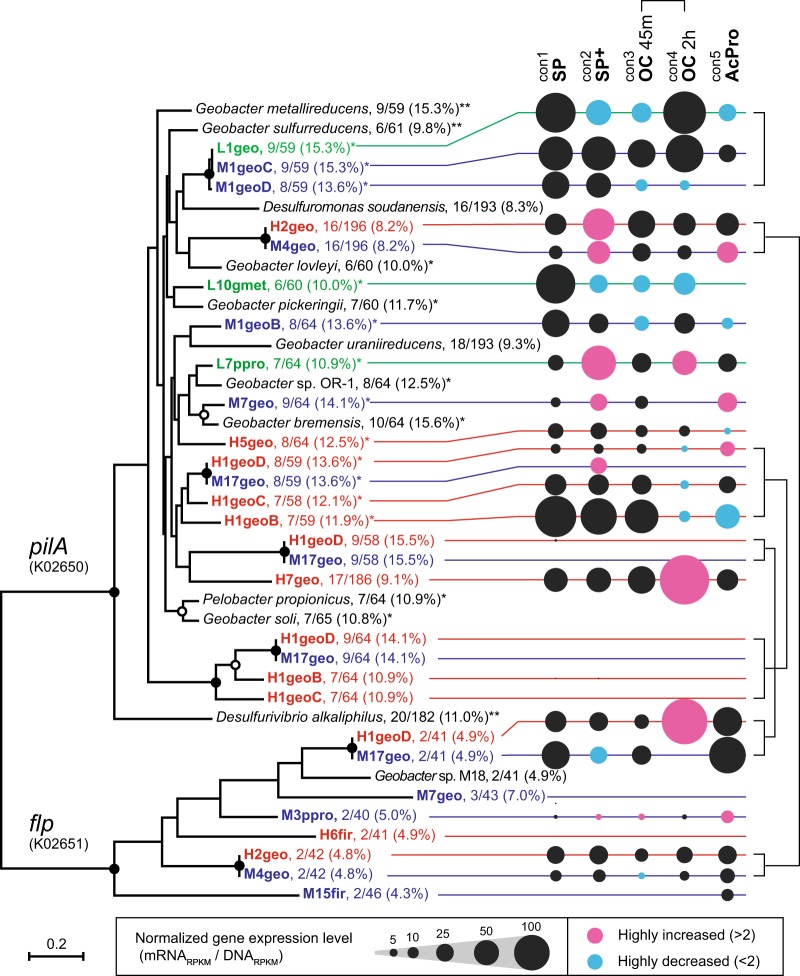
Phylogeny and gene expression trends of potential e-pili (PilA) and flp pilus (Flp). Normalized gene expression levels (mRNA-RPKM / DNA-RPKM) of pilus main body genes, *pilA* and *flp*, under five operational conditions after applying different stimuli are shown by the size of black circles, while gene expressions that had a fold-change of >2 (pink circles) or <2 (blue circles) from the original SP condition are highlighted. Phylogenic tree of PilA/Flp short pilin protein sequences is generated using Maximum Likelihood method. Branch points supported with boot-strap values of >90% are indicated with closed circles, while those between 70 and 90% are indicated with open circles. The peptides are described as “bin-genome ID or reference species name, numbers of aromatic amino acids (FYWH)/length of peptide after maturation (% of aromatic amino acids in pilin)”, while potential e-pili are marked by * and confirmed e-pili are marked by **

In order to further characterize the e-pili coding ORFs, the alignment (Supplementary Figure S20), gene expression dynamics (Fig. 6, Supplementary Table S19), protein-based phylogenic tree, and aromatic amino-acid frequencies (Fig. 6) were summarized for all *pilA* (potential e-pili) and *flp* (coding another peritrichal pilus) genes in each OCS. The conductivity of potential e-pili was estimated from % aromatic amino acid per pilin (as over 9%) and seven essential aromatic amino acids for EET activity that were identified from either the mutant work in model *G. sulfurreducens* [56] or comparison between conductive e-pili of *Desulfurivibrio alkaliphilus* and non-conductive pili of *G. uraniireducens* for long-type PilA proteins [57]. From the results, 12 of the total 21 PilA proteins are proposed to be conductive, and many of which were expressed in the community and also highly responsive to the given stimuli (Fig. 6). Interestingly, most-dominant pan-genomes in SP-H and SP-M communities showed different *pilA*-encoding profiles for their sub-strains (H1geoB-D or M1geoB-D) respectively, and no *pilA* gene was shown in core genome sets for each pan-genome (H1geoA or M1geoA). As for OCS H1geo(pan), one of two PilA proteins in bin-genome H1geoB showed remarkably high gene expression within the pan-genome, and reduced the gene expression in response to both OC and AcPro stimuli (Fig. 6). On the other hands, bin-genome H1geoD coded three different PilA proteins, but two of them were not likely conductive e-pili with no gene expression, while the Flp pilus was more highly expressed than the conductive e-pili for the bin-genome H1geoD (Fig. 6). These results suggest that the differences of e-pili functionality might correlate with sub-strain heterogeneity in these pan-genomes, and provide different functional roles for each sub-strain within the electrogenic microbial communities.

### Stimulus-induced expression changes of metabolic marker genes

Gene expression trends of several metabolic marker genes that might be involved with the consumption of fermentation byproducts (acetate, hydrogen, formate, propionate, or ethanol) were also analyzed as a function of OCS and stimulus. A key gene, citrate synthase, *gltA* [58], proposed as an activity marker for acetate consumption via the TCA cycle in family *Geobacteraceae* microbes [59], was remarkably up-regulated during the AcPro stimulus for all *Geobacter/Pelobacter* OCSs. However, key genes associated with formate oxidation (*fdh* and *fdnH*, [60]) were highly downregulated in all OCSs during the AcPro stimulus; and the key genes relating to hydrogen metabolism (*hyaL* and *mvhD*, [61]) were differently up- or downregulated for each OCS without sucrose being available for fermentation (Fig. 5). These results suggest that formate and occasionally hydrogen are the usual substrates supplied during active sucrose fermentation. Key genes associated with propionate (*mcmA*) and ethanol (*adh*) metabolisms were highly expressed in only *Pelobacter* OCSs M3ppro and L7ppro, and not differently expressed in any of the nine OCSs except M3ppro (Fig. 5). The OCS M3ppro demonstrated a remarkable downregulation of the *mcmA* and *adh* genes when sucrose was not available, suggesting that ethanol and/or propionate were the preferred substrates correlated to EET activity for this OCS.

### Metabolic pathway maps for unique EET-active OCSs

**Fig. 7:**
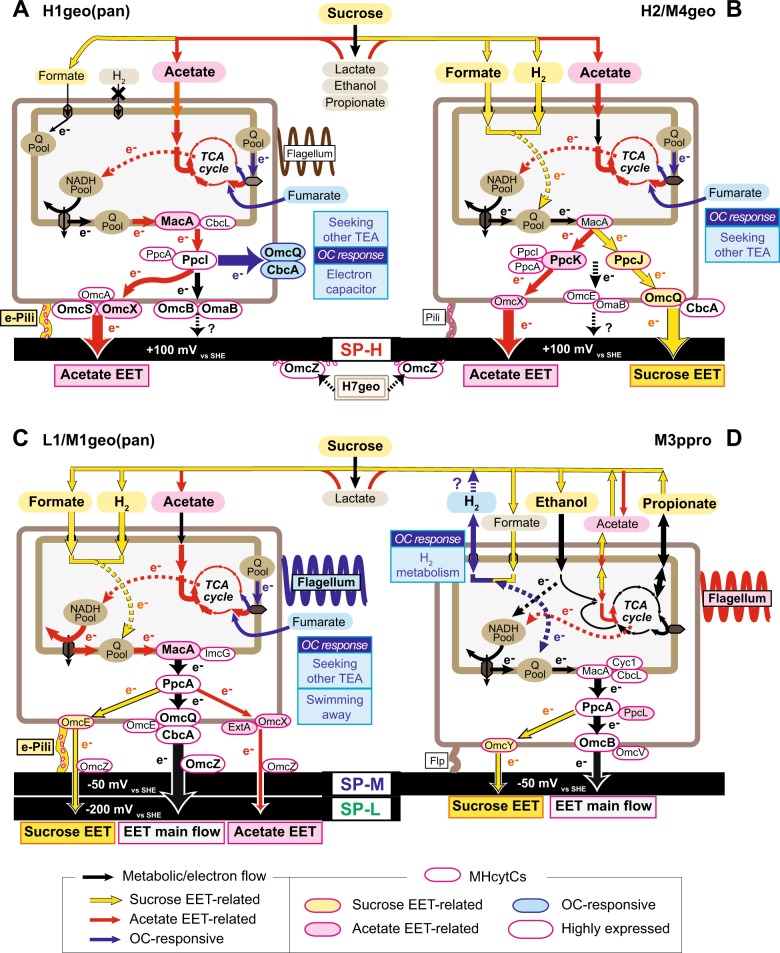
Stimuli responses of EET-related metabolic pathways of four abundantly shown *Geobacteraceae* OCSs. EET-related metabolic pathway map constructed by gene expression profiles of selected marker genes (refer to Supplementary Fig. S19) in the OCS H1geo(pan) (**a**), H2/M4geo (**b**), L1/M1geo(pan) (**c**), and M3ppro (**d**). The operational variable-associated metabolic flows and electron (e-) flow are analyzed by the wCCA analysis (Supplementary Fig. S21-S24). Sucrose-consuming EET (Sucrose EET) pathway was shown by yellow arrows, acetate-consuming EET (Acetate EET) pathway was shown by red arrows, and open circuit responses were shown by blue arrows with the detailed description about OC responses for each OCS in the blue box. Genes coding MH-cytC proteins for EET reactions are shown as oval (Names are described inside)

The gene expression profiles and dynamics were separately analyzed by using the heat-map and wCCA diagram for four dominant EET-active OCSs (Supplementary Fig. S21-S25), and the metabolic maps were constructed to describe the association of metabolic pathways to each EET/carbon stimulus (Fig. 7). The two dominant OCSs in the SP-H (H1geo(pan) and H2/M4geo) showed notably different expression responses to stimuli, suggesting different metabolic pathways and roles in the same community (Fig. 7a, b). The difference indicates that both metabolic functions were important for the positive surface redox-associated community. The H1geo(pan) exhibited a preferred acetate-consuming EET metabolic pathway with electrons moving through the TCA cycle to the quinone pool and using MacA and PpcI for IM and PP electron transfer to OM-MH-cytCs, OmcS, and OmcX, as well as conductive e-pili (Fig. 7a). The H1geo(pan) also showed remarkable upregulation of the CbcA-OmcQ complex in response to the lack of a solid-phase electron acceptor after OC stimulus. On the other hand, the H2/M4geo appeared to use the CbcA-OmcQ complex as the favored OM-MH-cytCs under sucrose-EET conditions, indicating that this complex could play strain-dependent roles (Fig. 7b). The H2/M4geo also utilized formate and hydrogen under the sucrose-EET condition, while acetate was only used when sucrose was depleted. Along with this metabolic switching, the OCS also changed the EET pathways depending on its substrate. The OCS stopped using the OmcQ-CbcA complex on OM-cytC and PpcJ on PP-cytC in favor of OmcX and PpcK for EET reaction to the electrode when acetate was used as the primary carbon source (Fig. 7b, Supplementary Fig. S22). These trends suggest that the H2/M4geo is more adaptable to changes in its surrounding environment than the most dominant OCS H1geo(pan), perhaps providing flexibility in the EET network and toughness from (sudden) environmental condition changes within the SP-H community.

Similar EET and carbon source flexibilities were also shown in OCS L1/M1geo(pan), the most abundant member within both SP-M and SP-L communities. This OCS mainly used the MacA as IM-cytC, PpcA as PP-cytC, CbcA-OmcQ complex as OM-cytC and OmcZ as secreted MH-cytC upon the switch from hydrogen/formate to acetate after sucrose depletion, and was able to modify the EET pathway to the conductive e-pili and the other OM-MH-cytCs, OmcE, OmcX and ExtA dependent with the redox and carbon source availability (Fig. 7c). This trend suggests that these metabolic flexibilities are a key for adapting to the more negative surface redox potential.

The metabolic pathway of the *Pelobacter* OCS M3ppro was considerably different from the other dominant *Geobacter* OCSs, and preferentially consumed ethanol to produce acetate and/or propionate under the sucrose-consuming EET condition (Fig. 7d). It appears that EET respiration may provide an optional pathway for the release of electron equivalents for the M3ppro, which was supported by the high gene expression of MH-cytCs, *omcB* (139th) and *ppcA* (150th) in 4506 M3ppro ORFs under sucrose-consuming SP-M condition. When EET was stopped, release of hydrogen via membrane bound hydrogenase became the preferred lifestyle. Although the *Pelobacter* OCS is affiliated with the *Geobacter* subsurface clade 2 (Fig. 2b), its metabolic role in this system was found to be different from the other *Geobacter* microbes as reported in the previous studies [40, 62].

## Discussion

In this study, several different anodic surface redox potentials were used to begin to understand how different members of EET-active microbial communities respond to the changes in surface redox potential and electron flow. Previously, population dynamics from a 16S rRNA gene-based community analysis showed different responses to changes in surface potentials for both electrogenic and fermentative phylotypes within sucrose-fed anodic communities [17]. The results indicated that the electrogenic *Geobacter* microbes affiliated with the well-known *G. metallireducens* clade increased current generation under lower surface potentials, while other strains affiliated with *Geobacter* subsurface clades 1 and 2 microbes increased current generation under higher surface potentials [17]. In the study reported here, we used a genome-centric metagenomic approach, successfully extracting nine draft genomes of the dominant *Geobacteraceae* members. This approach indicated a higher proportion of *Geobacteraceae* than previously reported in the 16S rRNA gene-based communities (Fig. 2a), presumably due to the lower copy number of rRNA operons per genome for this taxon [63]. In addition, we identified *Euryarchaeota* and *Actinobacteria* which had not been seen in the 16S rRNA gene-based communities, presumably because of the primer mismatch and/or difficulty of the annealing for high G + C content chromosomes. These results indicate that our metagenomics-based population analyses provide a more accurate view of the electrogenic biofilms.

Of the nine *Geobacteraceae* seen by their draft genomes, the most abundant members in all the three communities were affiliated to *Geobacter* Subsurface clade 1 or 2 (Fig. 2b). Although *Geobacter* subsurface clade microbes have often been observed as major members of metal reducing subsurface environments or electrogenic communities [17, 64, 65], only a few studies have characterized EET mechanisms in these members [10, 66, 67]. Recently, electrochemical characteristics of six *Geobacteraceae* isolates were compared between model *G. metallireducens* clade species and subsurface clade species [67]. The electrochemical characterization indicated that the model *G. metallireducens* clade species used only one EET pathway irrespective of two different anode potentials (+200 mV or −200 mV vs SHE), while species from the subsurface clades used multiple EET pathways, which can be optimized depending on the anode potentials [67]. These results strongly suggest that the subsurface clade microbes are able to sense the surface redox potential and coordinate the EET pathways. Our stimulus-induced metatranscriptomic analyses (Fig. 5) and the metabolic pathway reconstruction (Fig. 7) clearly revealed that the dominant *Geobacter* subsurface clade microbes regulated the EET and metabolic pathways in response to changes in the surface redox potential, carbon source conditions, and the electron flow. These diverse metabolic and EET capabilities may be a key for the subsurface clade microbes to dominate in EET-active biofilms fed with fermentable sugars under both positive and negative surface redox conditions.

The 2nd most dominant *Geobacteraceae* OCSs within SP-M and SP-L communities, M3ppro and L7ppro respectively, showed remarkably less MH-cytCs in their genomes than those in the other *Geobacteraceae* OCSs (Fig. 2b). The M3ppro and L7ppro ware both closely related to *Pelobacter propionicus*, a genus affiliated to *Geobacter* Subsurface clade 2 in family *Geobacteraceae* (Fig. 2b). The *Pelobacter* species can be divided into two distinct subgroups: the *Geobacter* and *Desulfuromonas* clusters [40]. Both subgroups are capable of functioning either as fermenters or syntrophs by partially oxidizing organic compounds to hydrogen and acetate [68], which is different from those of the genera *Geobacter* and/or *Desulfuromonas* [62]. The *P. propionicus* consumed ethanol to produce propionate and acetate via methylmalonyl CoA [69], and the genes coding for the reported active enzymes for the pathways were all highly expressed in the OCS M3ppro (Supplementary Data 5), suggesting that the main metabolic role for the OCS was ethanol consumption in the community. The stimulus-induced metatranscriptomic comparison also suggested the *Pelobacter* OCSs to be metabolically robust, operating in several different metabolic modes depending on the surrounding conditions: (1) consumption of ethanol with EET respiration via MH-cytCs and release of propionate and acetate under sucrose fermentation with EET condition, (2) hydrogen release instead of EET respiration under OC condition, and (3) likely consumption of acetate with EET respiration under AcPro condition (Fig. 7d). The complete oxidation of acetate with EET respiration has not been reported for *P. propionicus*, and no acetate transporter is coded in the genome [70]; however, our gene expression trends (Supplementary Fig. S24) showed that another acetate transporter YaaH (K07034) [71] was expressed instead of typical acetate transporter ActP (K14393) for *Geobacteraceae*, and also citrate synthase GltA (K01647) that is a key enzyme for complete acetate oxidation via TCA cycle [58] was highly expressed in response to the AcPro stimulus. The YaaH-type acetate transporter gene was also upregulated for several subsurface clade *Geobacter* OCSs including L1/M1geo(pan) (Additional data 5, 6). These results suggest that acetate consumption was likely introduced for the OCS M3ppro by the AcPro stimulus. This metabolic flexibility for the *Pelobacter* OCSs may also provide a resilience for the SP-M/SP-L electrogenic microbial community in response to environmental perturbations.

The molecular mechanism of EET has been widely investigated, in the model species, *G. sulfurreducens*, where several MH-cytCs and conductive e-pili were found to play crucial roles in this process [45, 46, 48, 54, 55, 72]. When the *G. sulfurreducens* was grown on poised electrodes, only two IM-cytCs were seen to correlate with different surface redox potentials: CbcL was induced at –100 mV vs SHE or below, while ImcH was induced at potentials above −100 mV [50, 73, 74]. *Geobacter* subsurface clade species, on the other hand, have not been examined with regard to their responses to different surface redox potentials. Results presented here (Fig. 5) revealed the divergent characteristic EET pathways within the subsurface clade OCSs, and how this group of microbes responded to the environmental perturbation related to the surface redox potentials. For example, MacA and CbcL were induced under all conditions, while CbcL was favored under more positive surface potentials (Fig. 5), which is decidedly different from that seen in *G. sulfurreducens* [50, 73, 74]. With regard to the PP-cytCs, several uncharacterized cytochrome c3 genes were expressed under positive surface redox-favored subsurface clade microbes, even though PpcA, known to be important as an electron carrier in the periplasm for *G. sulfurreducens* [26, 47], was conserved in the subsurface clade microbes. These differences suggest that different EET pathways function for the subsurface clade species than have been reported for *G. sulfurreducens*.

Among OM MH-cytCs, we found that the CbcA–OmcQ complex exhibited a strong response to the EET related stimuli for many of the subsurface clade microbes; however, the dynamics (upregulation, downregulation, or no change) were different in each OCS (Fig. 5). The CbcA–OmcQ cluster is structurally similar to the OM porin-MH-cytC complex that is an essential conduit for EET reaction in the model species [46, 72], suggesting that this cluster transfers electrons outside of the cell body in response to new surface redox conditions. In fact, L1/M1geo(pan) and H2/M4geo utilized this conduit for the central EET pathway (Fig. 7C) or under hydrogen/formate-consuming EET conditions (Fig. 7b), respectively. Interestingly, the other positive surface redox-favored subsurface clade OCSs, H1geo(pan), H5geo, H7geo and M7geo, showed significant up-regulation of the CbcA–OmcQ porin cluster or CbcA alone in response to the OC stimulus, suggesting that the CbcA-OmcQ conduit was important for these OCSs to adapt to the no-EET condition (Figs. 5, 7a). Recently, it was proposed that EET-active *Geobacter* biofilms could work as electron capacitors by using MH-cytCs [75]. Our gene expression results are consistent with the idea that the OmcQ–CbcA conduit might have a role in the capture and storage of excess electrons in response to EET limitation, though it seems equally likely that this is a response aimed at increasing EET-capacity in the “search” for other electron acceptors.

In addition to the OM-cytCs, we also found that wide variety of potential conductive or non-conductive e-pili coded by *pilA* gene in the nine *Geobacteraceae* microbes (Fig. 6), and their diverse gene expression responses to the given stimuli (Fig. 5). From the transcripts comparison between EET respiration and fumarate reduction of model *G. sulfurreducens* strain, significant increases have been shown in *pilA* gene along with two MH-cytCs, *omcB* and *omcZ* under EET condition [49], suggesting that the high gene expression of conductive e-pili was correlated with the EET reaction. The further confirmation of the importance of PilA has achieved by the mutant-based studies, and aromatic amino acids in the protein are key for the conductivity [49, 56]. On the other hands, the PilA of *G. uraniireducens* belonging to *Geobacter* subsurface clade 1 was longer than that of *G. sulfurreducens*, and showed no conductivity [57]. Within the nine *Geobacteraceae* OCSs in this study, only one OCS H2/M4geo showed the longer non-conductive type e-pili, but other OCSs except for OCS M3ppro had one or more short-type e-pili (Fig. 6). Interestingly, two most dominant *Geobacteraceae* OCSs, H1geo(pan) and L1/M1geo(pan), in the electrogenic microbial communities showed remarkably high gene expression of conductive short-type e-pili, even though the OCSs affiliate with *Geobacter* Subsurface clades (Fig. 6). These results indicate that the conductive e-pili was also functional for the EET-active *Geobacter* subsurface clade microbes, and important for the electrogenic microbial communities adapted to the different surface redox potentials.

In conclusion, our systematic stimulus-induced metatranscriptomic approach has revealed specialized metabolic niches and EET characteristics for nine different EET-active *Geobacteraceae* microbes (OCSs), several of which are affiliated to poorly characterized subsurface groups. Distribution of their functional roles under different surface redox potential conditions was defined by electron donor preference, metabolic flexibility, surface redox (solid-state electron acceptor) preference, and by their responses to changes in each of these parameters, including an emergency response to a no EET (open circuit) environment. These differences were clearly demonstrated by their gene expression dynamics after the given stimuli, which included the regulatory responses of genes controlling the synthesis of different MH-cytCs and conductive e-pili involved with EET, as well as genes related to the substrate consumption. While many members of the *Geobacteraceae* are known to encode 100 or more *c-*type cytochromes, a wide variety of gene expression responses were shown to the various EET stimuli, indicating that the wide variety of the EET-active *Geobacteraceae* OCSs may have provided the communities with an impressive ability to handle major changes in surface potential and carbon source availability. The results presented here offer a new perspective on the complexity of electrogenic communities observed, and some initial insights into how they may regulate community function and provide adaptability and robustness to a variety of (sudden) environmental perturbation. Overall, these results have opened a door toward unraveling the diverse microbial life within EET-active communities adapted to the different surface redox potentials.

## Electronic supplementary material

Supporting information

Datasets
