# Supplementary material for: Comparative metatranscriptomics reveals extracellular electron transfer pathways conferring microbial adaptivity to surface redox potential changes
Source: ISME J. 2018 Jul 26;12(12):2844–63. doi: 10.1038/s41396-018-0238-2 (PMC6246609; doi:10.1038/s41396-018-0238-2)
Supplement: Supplementary file 1 — Supporting information [file 41396_2018_238_MOESM1_ESM.pdf]

Comparative metatranscriptomics reveals extracellular electron transfer pathways conferring microbial adaptivity to surface redox potential changes

Shun'ichi Ishii*, Shino Suzuki* Aaron Tenney, Kenneth H. Nealson, Orianna Bretschger

* Corresponding authors
SI, E-mail: sishii@jamstec.go.jp; Tel: +81-88-878-2279; Fax: +81-88-878-2192
SS, E-mail: sisuzuki@jamstec.go.jp; Tel: +81-88-878-2224; Fax: +81-88-878-2192

SUPPLEMENTARY INFORMATION

Table of Contents

SUPPLEMENTARY FIGURES 2

SUPPLEMENTARY TABLES.. 24

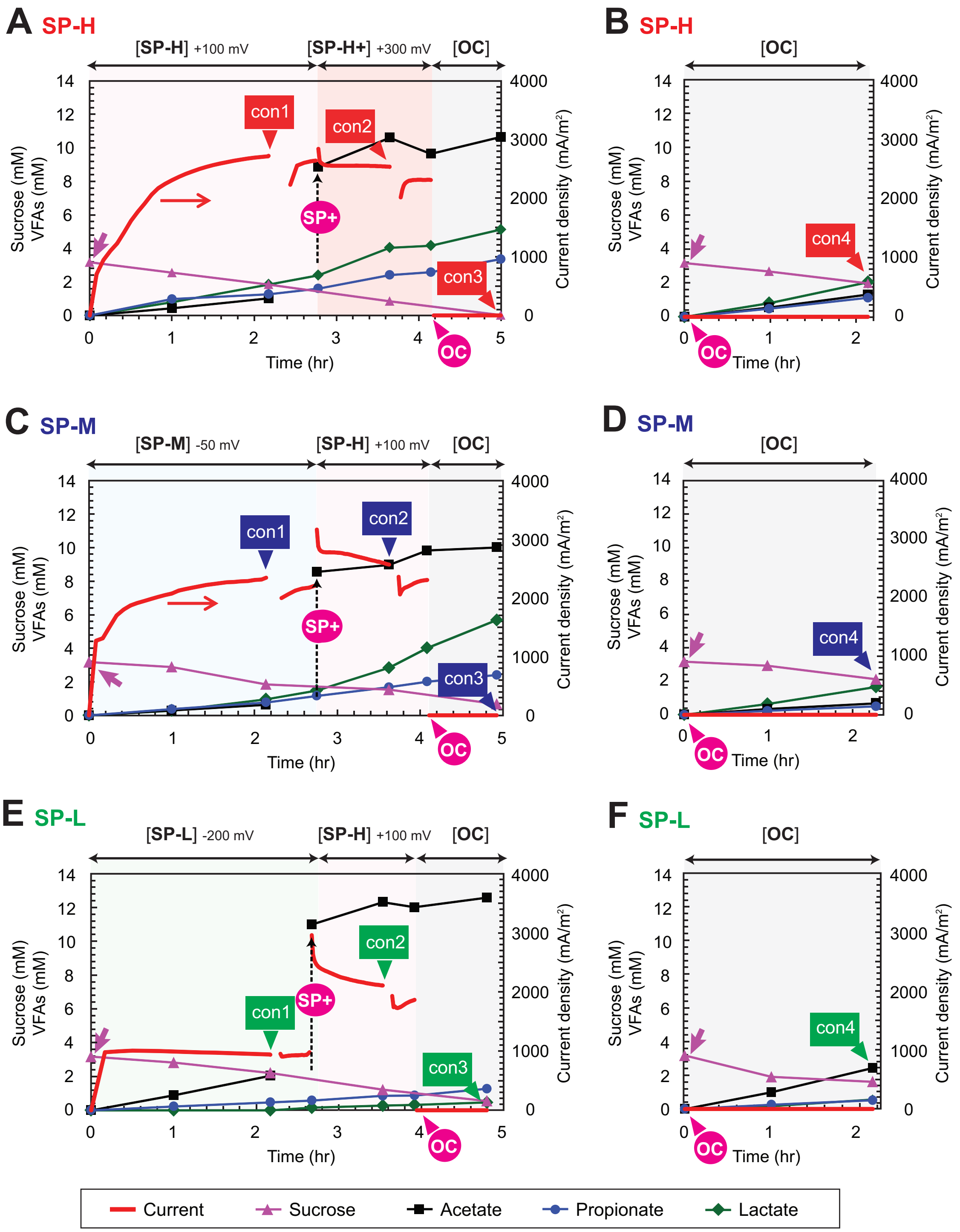

Supplementary Fig. S1 | Operational data of four sampling conditions (con1 - con4).

Current and substrate/byproducts responses throughout the different sampling conditions during the sucrose consuming conditions in SP-H (AB), SP-M (CD), and SP-L (EF) comminities. The 2.5 mM sucrose was injected at the starting point of batch (purple arrow). Initial three conditions, con1 after SP operation, con2 after SP+ stimulus, and con3 after OC stimulus (red arrowhead) were harvested in first batch (A, C, E), subsequently con5 samples (acetate/propiponate consuming EET condition, stimulus AcPro) were collected in second batch (data not shown). In third batch (B, D, F), con4 samples (sucrose-consuming OC as similar substartae condition to con1) were collected after OC stimulus. As for SP+ stimulus, anode surface potentials were changed to more positive potentials and ~9 mM of acetate was injected (dashed arrow) at 2.8 hr (A, C, E).

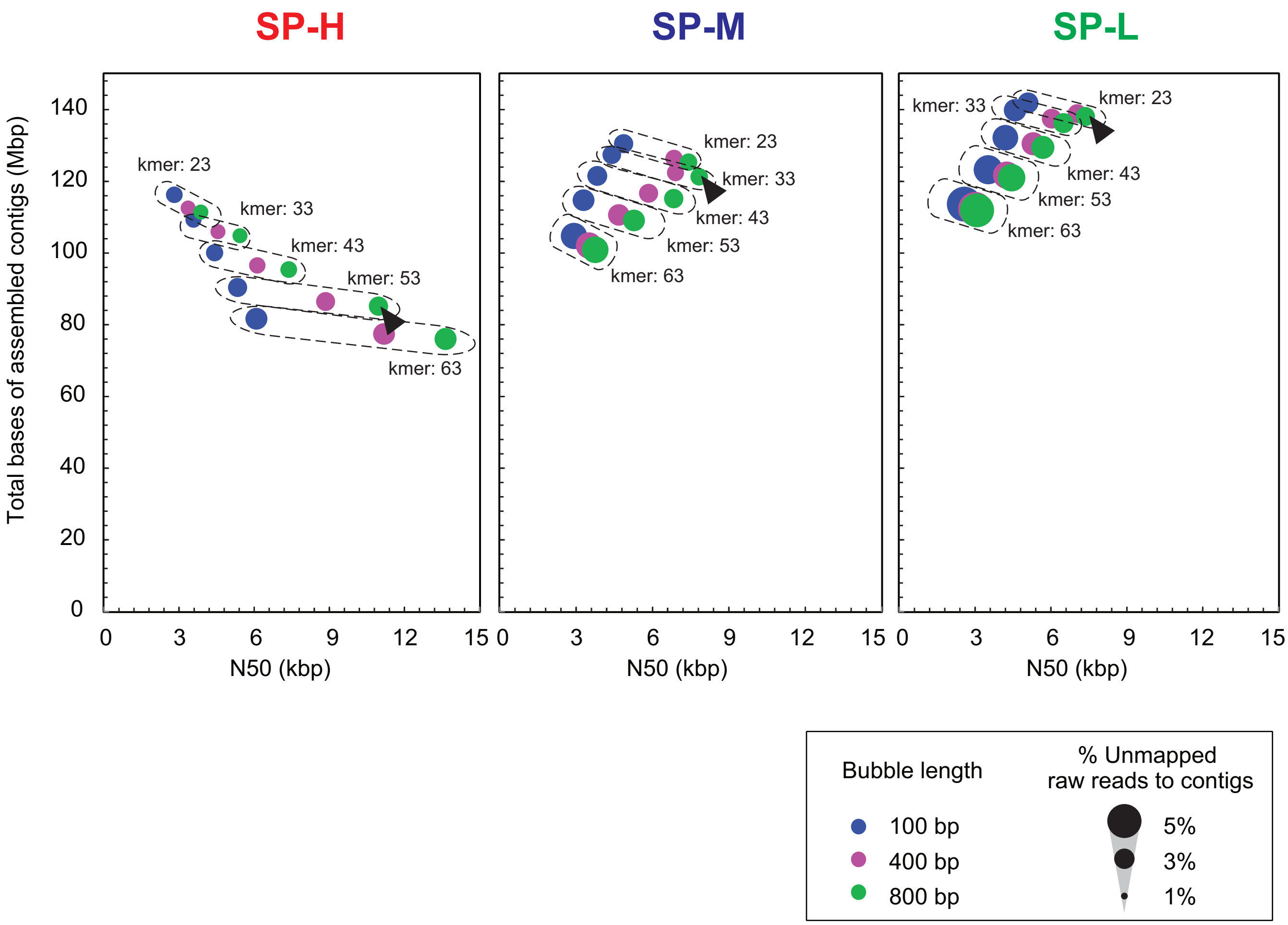

Supplementary Fig. S2 | Metagenomic assembly metrics with the various parameters on *de novo* assembly software.

Three metagenomic assembly metrics, total bases (y-axis), N50 (x-axis), and % unmapped raw reads back to the assmbled contigs (size of bubble), are shown with different parameters of both kmer size (dashed line) and bubble length (color) in CLC *de novo* assembly cell 4.0. Filled arrowhead indicates an assembly used in this study.

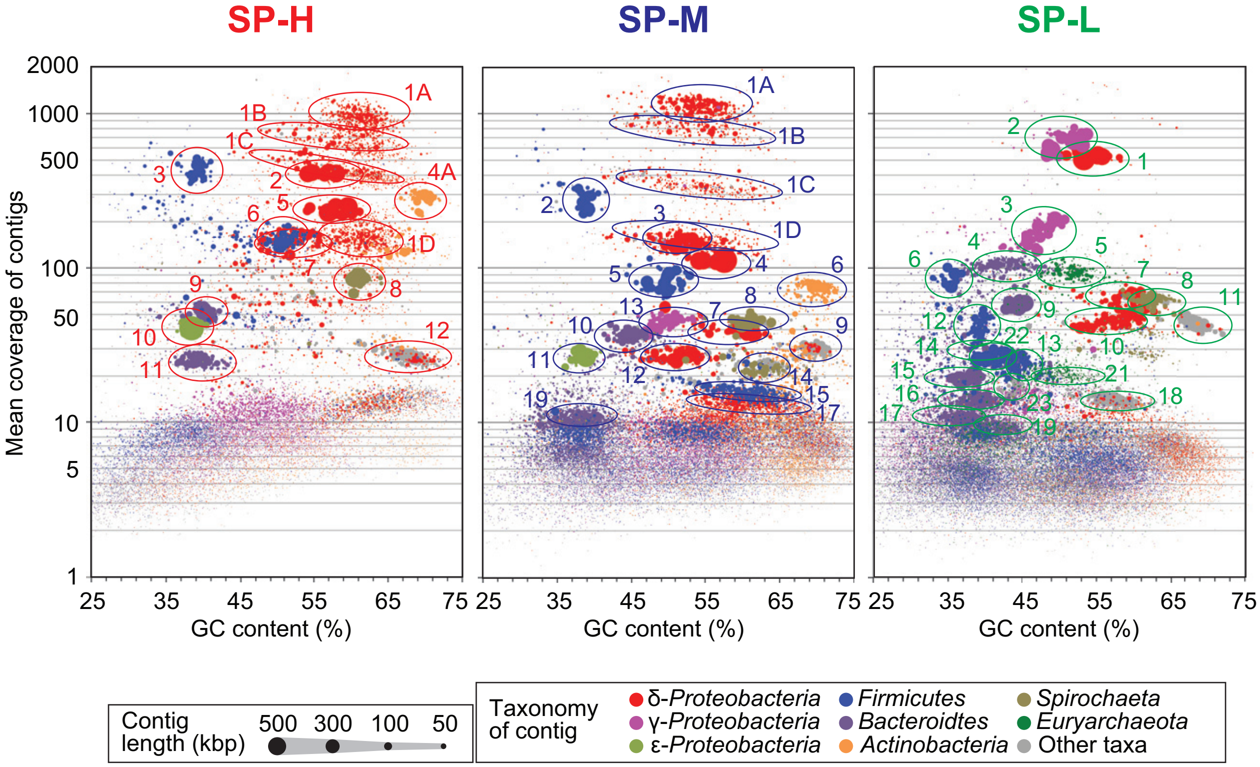

Supplementary Fig. S3 | Identified bin-genomes of dominant strains within the EET-active microbial communities established under different electrode surface potentials SP-H, SP-M, or SP-L.

Bin-genome clusters (colored circles, ID described near circles) which were established using the estimated taxonomic classification of contigs (color of dots), contig lengths (size of dots), GC content of contigs (%), and mean coverage of contig. Microbial communities were established under +100 mV vs SHE for SP-H, -50 mV vs SHE for SP-M, and -200 mV vs SHE for SP-L, respectively.

A

	Hcon1	Hcon2	Hcon3	Hcon4	Hcon5	Mcon1	Lcon1
Hcon1	1	0.995	**0.955**	0.993	0.966	**0.087**	**-0.113**
Hcon2	0.995	1	0.945	0.997	0.957	0.082	-0.109
Hcon3	0.955	0.945	1	0.940	0.984	0.101	-0.115
Hcon4	0.993	0.997	0.940	1	0.952	0.081	-0.104
Hcon5	0.966	0.957	0.984	0.952	1	0.116	-0.106
Mcon1	0.087	0.082	0.101	0.081	0.116	1	0.029
Lcon1	-0.113	-0.109	-0.115	-0.104	-0.106	0.029	1

B

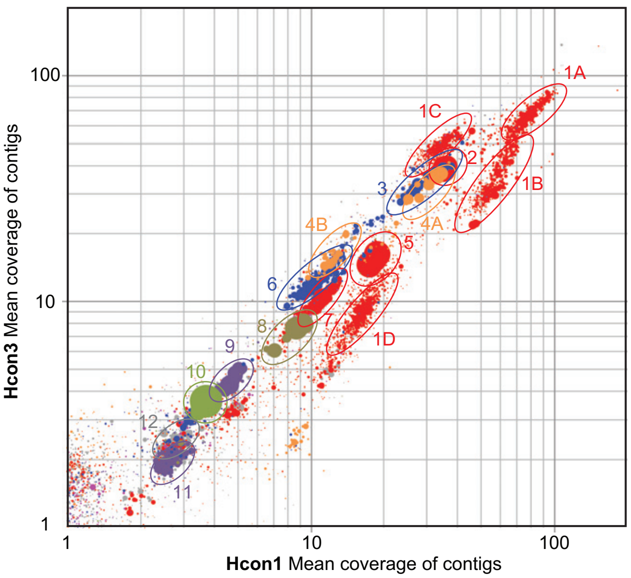

C

D

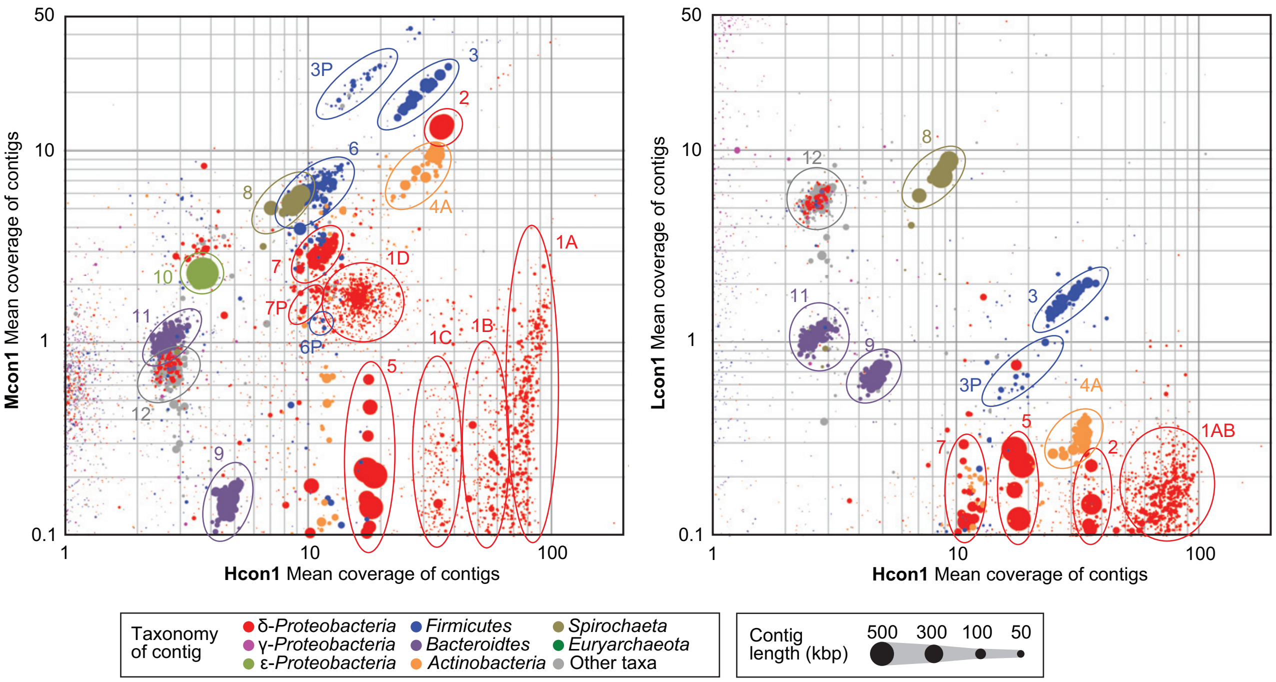

Supplementary Fig. S4 | Identification of bin-genomes of dominant strains within SP-H microbial communities by using cross read mapping.

Panel A shows similarity materix of mean coverage of contigs between five different samples of SP-H, and con1 samples for SP-M and SP-L. Raw reads for each condition were separately mapped to SP-H contigs by using similarity cut-off 0.95 and length cut-off 0.7. Associations used for further bin-genome cluster clean up by cross mapping chart were highlighted by white letters on red background. Panel B-D show SP-H bin-genome clusters (colored circles, ID described near circles) which were used for refining the bin-genomes; panel B for Hcon1 vs Hcon3, panel C for Hcon1 vs Mcon1, and panel D for Hcon1 vs Lcon1. Color of dots indicate estimated taxonomy of contigs, while size of dots indicate length of contigs.

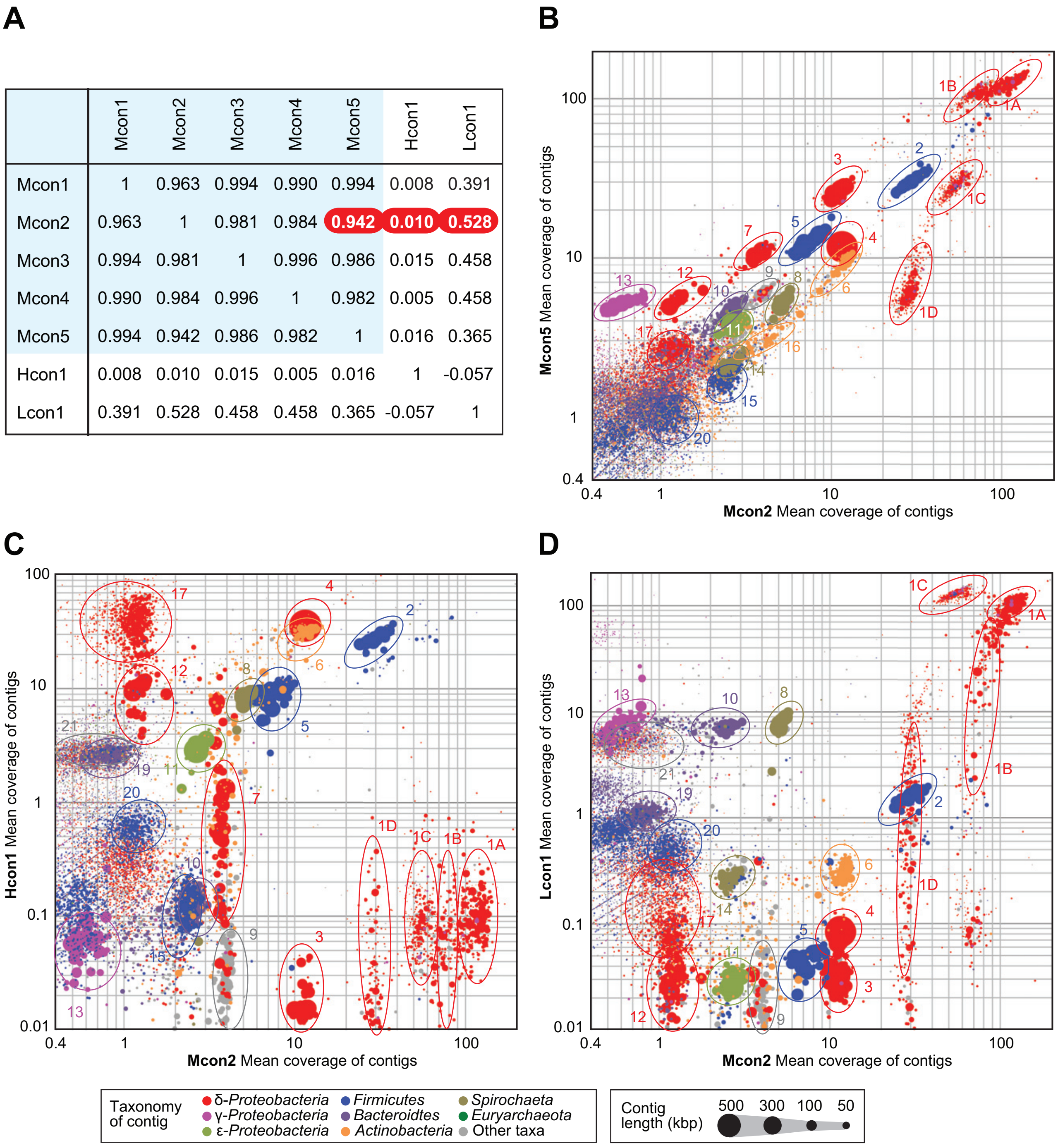

	Mcon1	Mcon2	Mcon3	Mcon4	Mcon5	Hcon1	Lcon1
Mcon1	1	0.963	0.994	0.990	0.994	0.008	0.391
Mcon2	0.963	1	0.981	0.984	**0.942**	**0.010**	**0.528**
Mcon3	0.994	0.981	1	0.996	0.986	0.015	0.458
Mcon4	0.990	0.984	0.996	1	0.982	0.005	0.458
Mcon5	0.994	0.942	0.986	0.982	1	0.016	0.365
Hcon1	0.008	0.010	0.015	0.005	0.016	1	-0.057
Lcon1	0.391	0.528	0.458	0.458	0.365	-0.057	1

Supplementary Fig. S5 | Identification of bin-genomes of dominant strains within SP-M microbial communities by using cross read mapping.

Panel A shows similarity materix of mean coverage of contigs between five different samples of SP-M, and con1 samples for SP-H and SP-L. Raw reads for each condition were separately mapped to SP-M contigs by using similarity cut-off 0.95 and length cut-off 0.7. Associations used for further bin-genome cluster clean up by cross mapping chart were highlighted by white letters on red background. Panel B-D show SP-M bin-genome clusters (colored circles, ID described near circles) which were used for refining the bin-genomes; panel B for Mcon2 vs Mcon4, panel C for Mcon2 vs Hcon1, and panel D for Mcon2 vs Lcon1. Color of dots indicate estimated taxonomy of contigs, while size of dots indicate length of contigs.

A

	Lcon1	Lcon2	Lcon3	Lcon4	Lcon5	Hcon1	Mcon1
Lcon1	1	0.904	0.764	0.808	0.844	0.147	0.290
Lcon2	0.904	1	0.955	0.959	0.947	0.126	0.208
Lcon3	0.764	0.955	1	0.977	0.942	0.094	0.125
Lcon4	0.808	0.959	0.977	1	0.978	0.151	0.159
Lcon5	0.844	0.947	0.942	0.978	1	0.189	0.186
Hcon1	0.147	0.126	0.094	0.151	0.189	1	0.199
Mcon1	0.290	0.208	0.125	0.159	0.186	0.199	1

B

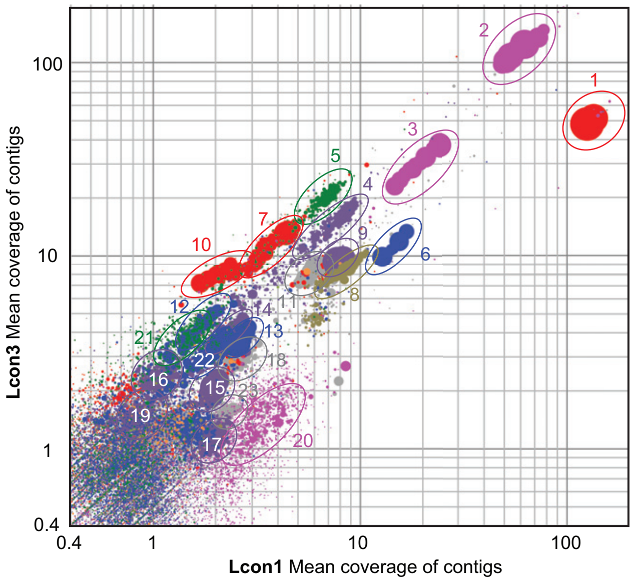

C **D**

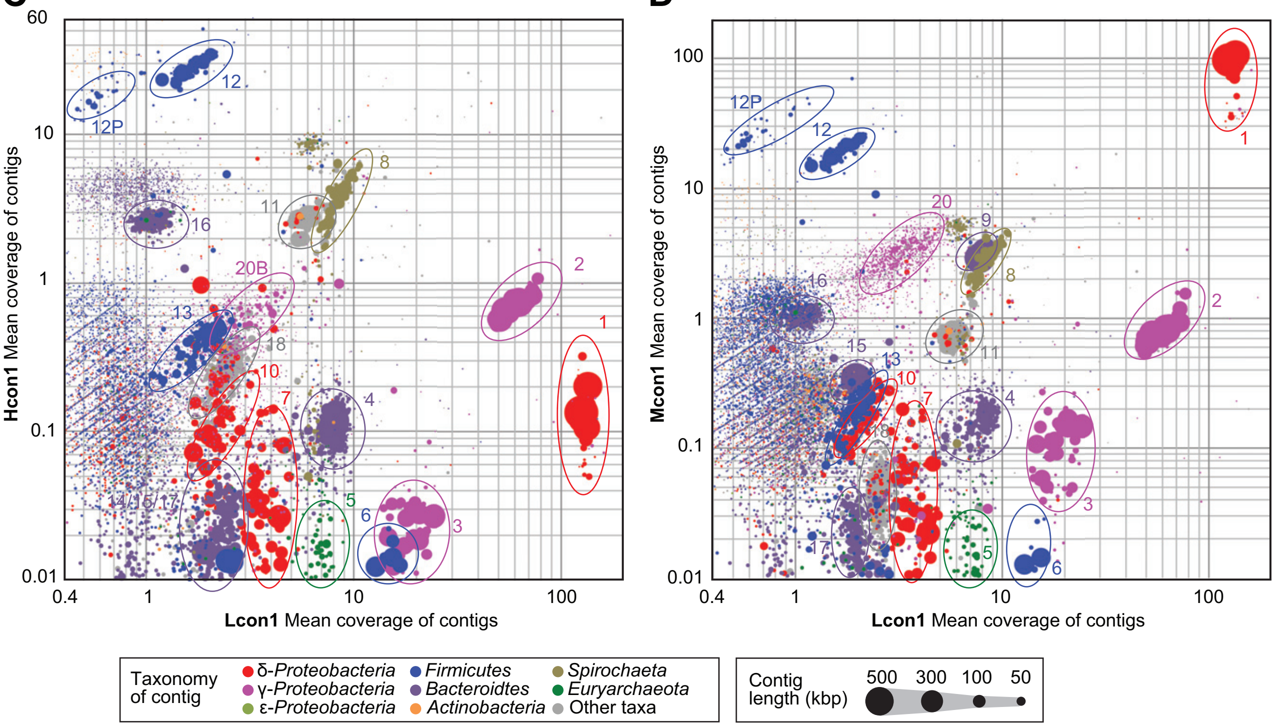

Supplementary Fig. S6 | Identification of bin-genomes of dominant strains within SP-L microbial communities by using cross read mapping.

Panel A shows similarity materix of mean coverage of contigs between five different samples of SP-L, and con1 samples for SP-H and SP-L. Raw reads for each condition were separately mapped to SP-L contigs by using similarity cut-off 0.95 and length cut-off 0.7. Associations used for further bin-genome cluster clean up by cross mapping chart were highlighted by white letters on red background. Panel B-D show SP-L bin-genome clusters (colored circles, ID described near circles) which were used for refining the bin-genomes; panel B for Lcon1 vs Lcon3, panel C for Lcon1 vs Hcon1, and panel D for Lcon1 vs Mcon1. Color of dots indicate estimated taxonomy of contigs, while size of dots indicate length of contigs.

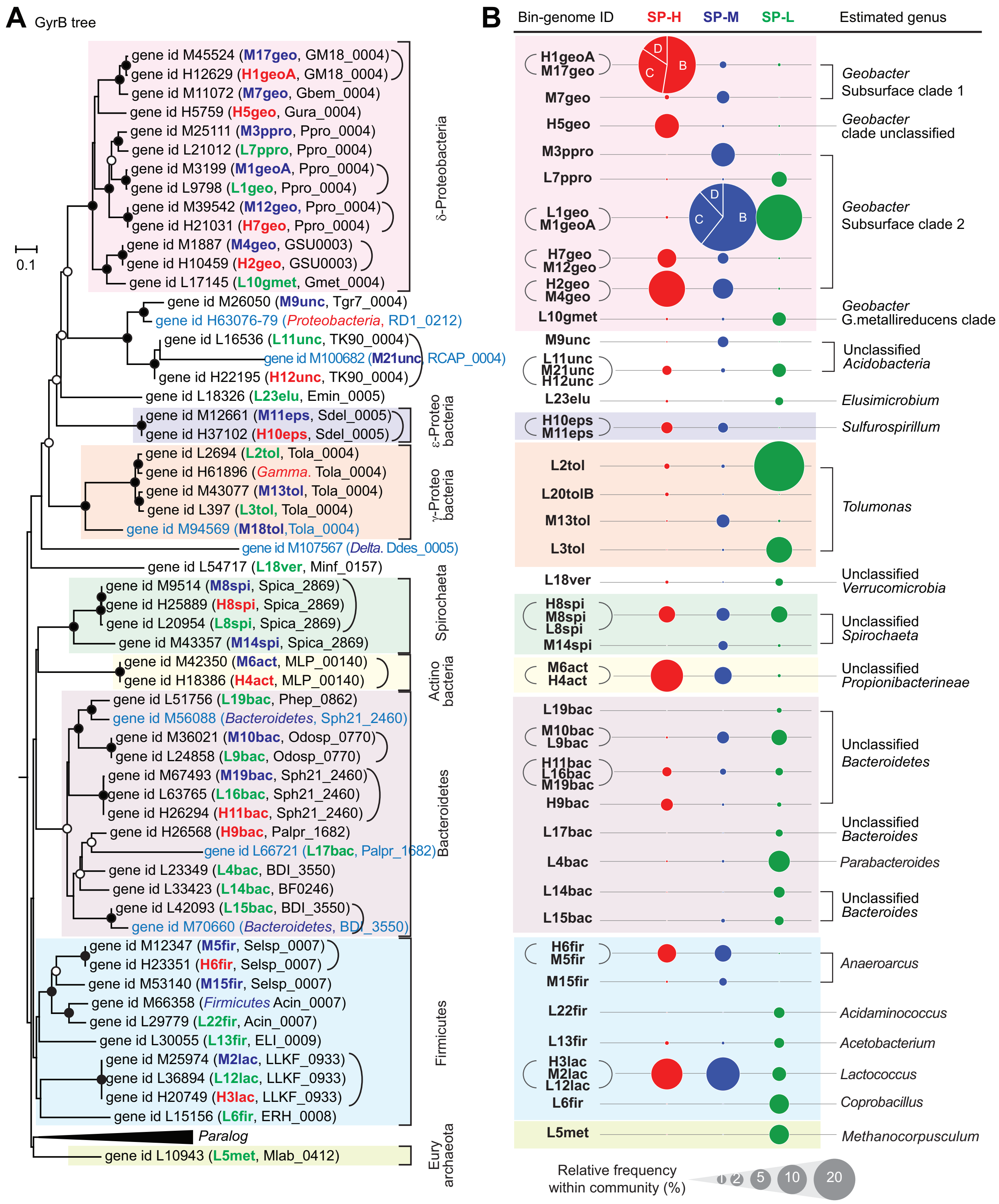

Supplementary Fig. S7 | Taxonomic positions and relative frequencies of bin-genomes.
Panel A shows a neighbor-joining phylogenetic tree of DNA gyrase beta subunit (GyrB) genes for bin-genomes observed in metagenomics analysis. Blue letters indicate using partial peptide information. The peptides are described as "orf ID (**bin-genome ID**, closest relative in RefSeq)". Branch points supported with bootstrap values (100 trials) of >90% are indicated with closed circles, while those between 70% and 90% are indicated with open circles. Panel B shows relative frequencies within communities among SP-H, SP-M, and SP-L.

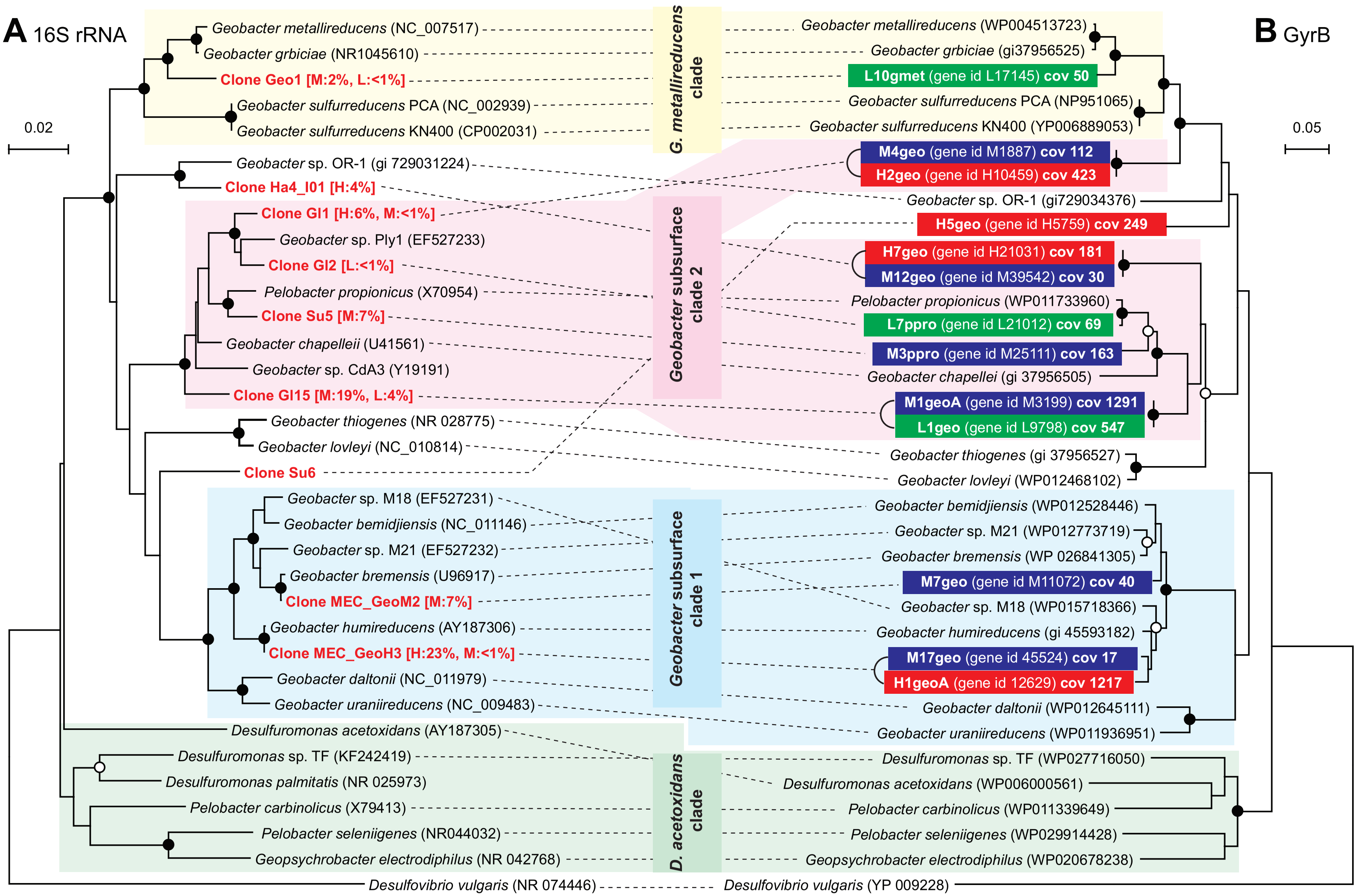

Supplementary Fig. S8 | Neighbor-joining phylogenetic trees of 16S rRNA and GyrB showing positions of *Desulfuromonadales* strains. Panel A for 16S rRNA partial gene includes major *Desulfuromonadales* phylotypes observed in clone library analysis. The phylotypes are described as “clone ID [Community ID: % frequency]”. Panel B for DNA gyrase beta subunit (GyrB) gene includes major *Desulfuromonadales* peptides observed in metagenomics analysis. The peptides are described as “bin-genome ID (orf ID) mean coverage”. Branch points supported with bootstrap values (100 trials) of >90% are indicated with closed circles, while those between 70% and 90% are indicated with open circles. Accession numbers of reference sequences are indicated in parentheses.

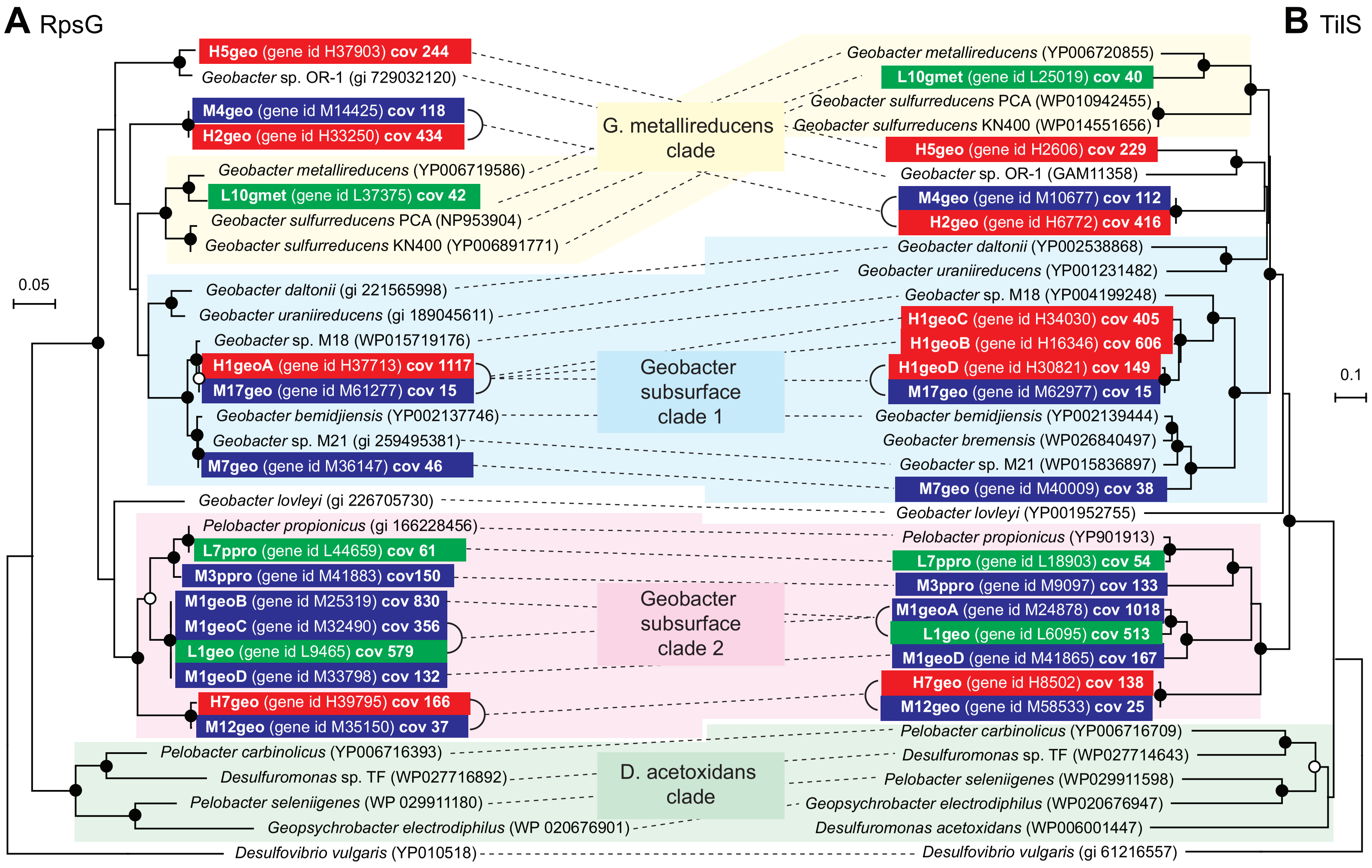

Supplementary Fig. S9 | Neighbor-joining phylogenetic trees of RpsG and TilS showing positions of *Desulfuromonadales* strains. Panel A is a phylogenetic tree for small subunit ribosomal protein S7 (RpsG) gene includes major *Desulfuromonadales* peptides observed in metagenomics analysis, while panel B is a phylogenetic tree for tRNA(Ile)-lysidine synthase (TilS) gene. The peptides are described as "bin-genome ID (orf ID) mean coverage". Branch points supported with bootstrap values (100 trials) of >90% are indicated with closed circles, while those between 70% and 90% are indicated with open circles. Accession numbers of reference sequences are indicated in parentheses.

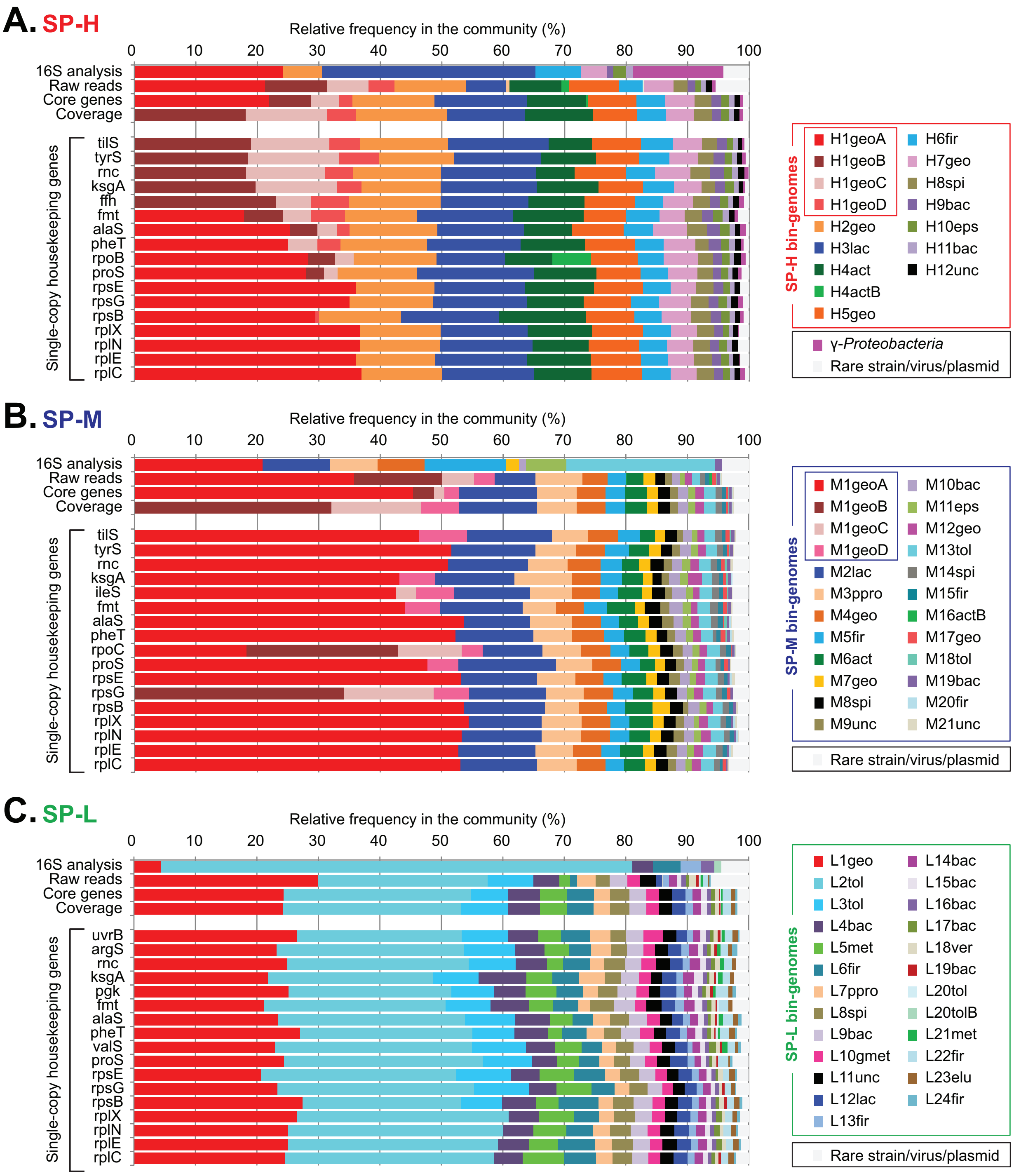

Supplementary Fig. S10. Single-copy housekeeping gene-based analysis to profile microbial diversity using metagenomic analyses and bin-genome clustering

Relative frequencies of each strain within SP-H (A), SP-M (B) and SP-L (C) estimated from a relative abundance of 16S rRNA gene (16S analysis), percentage of mapped raw reads to each bin-genome (Raw reads), an average value of reads per kilobase per million mapped reads (RPKMs) for 12 single-copied housekeeping genes (Core genes), an average RPKM value of contigs associated with each bin-genome (Coverage), and a RPKM value of twelve individual single-copied housekeeping genes.

A

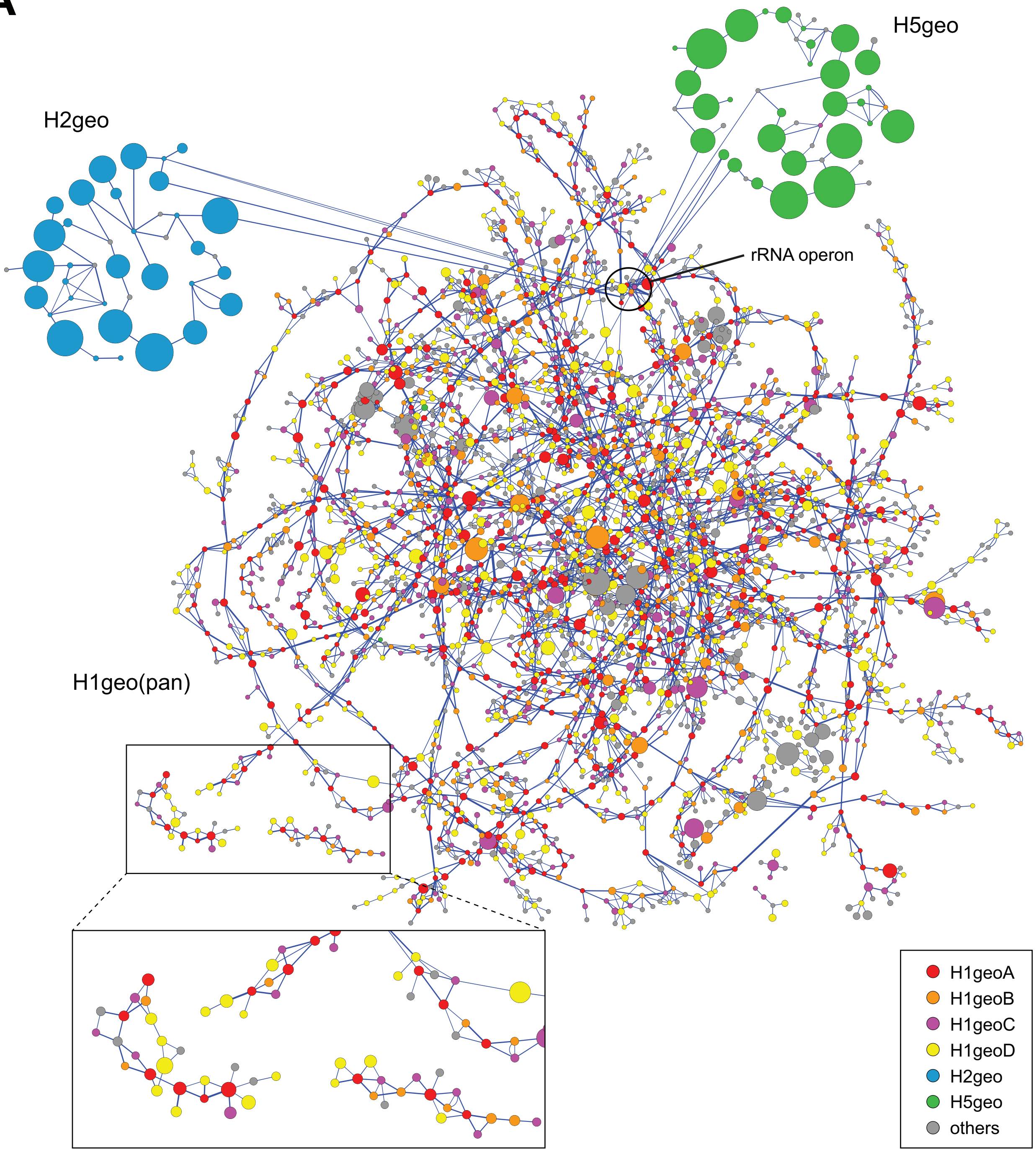

Supplementary Fig. S11 | Visualization of paired-end connections between ends of contigs for dominant *Geobacter/Pelobacter* members in each community.

Contigs (colored bubbles) are linked by blue lines to show paired-end connections (>5) between edges of contigs. A network graph is generated based on three dominant *Geobacter/Pelobacter* bin-genomes in SP-H (panel A), SP-M (panel B), or SP-L (panel C). The bin-genome ID for each contig is shown by using different colors. The line width is scaled by the number of connections, while the size of bubbles is scaled by the length of contigs. The network graph was generated directly from a read mapping file to contigs using CLC Genomics Workbench and visualised using Cytoscape.

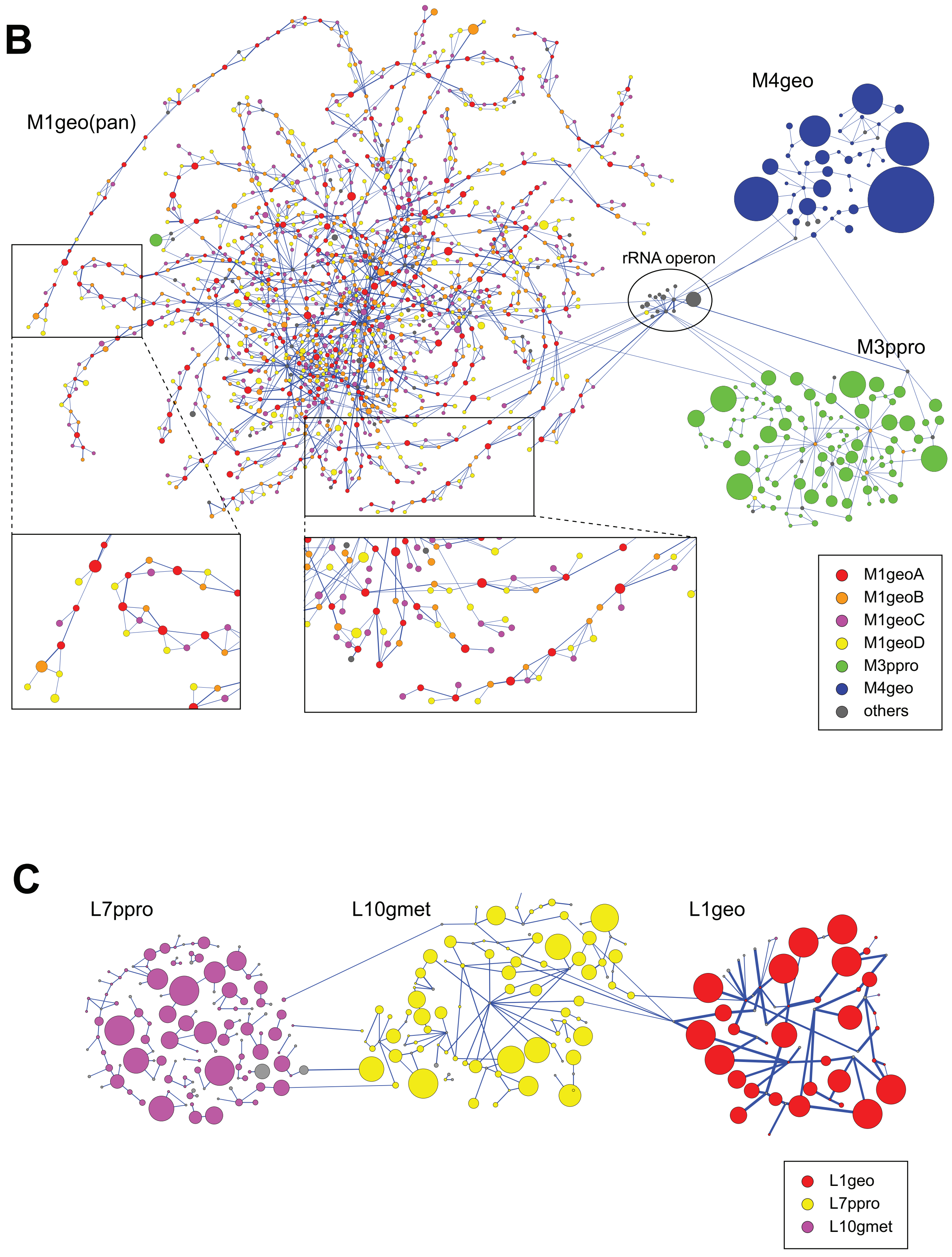

Supplementary Fig. S11 | *Continued*.

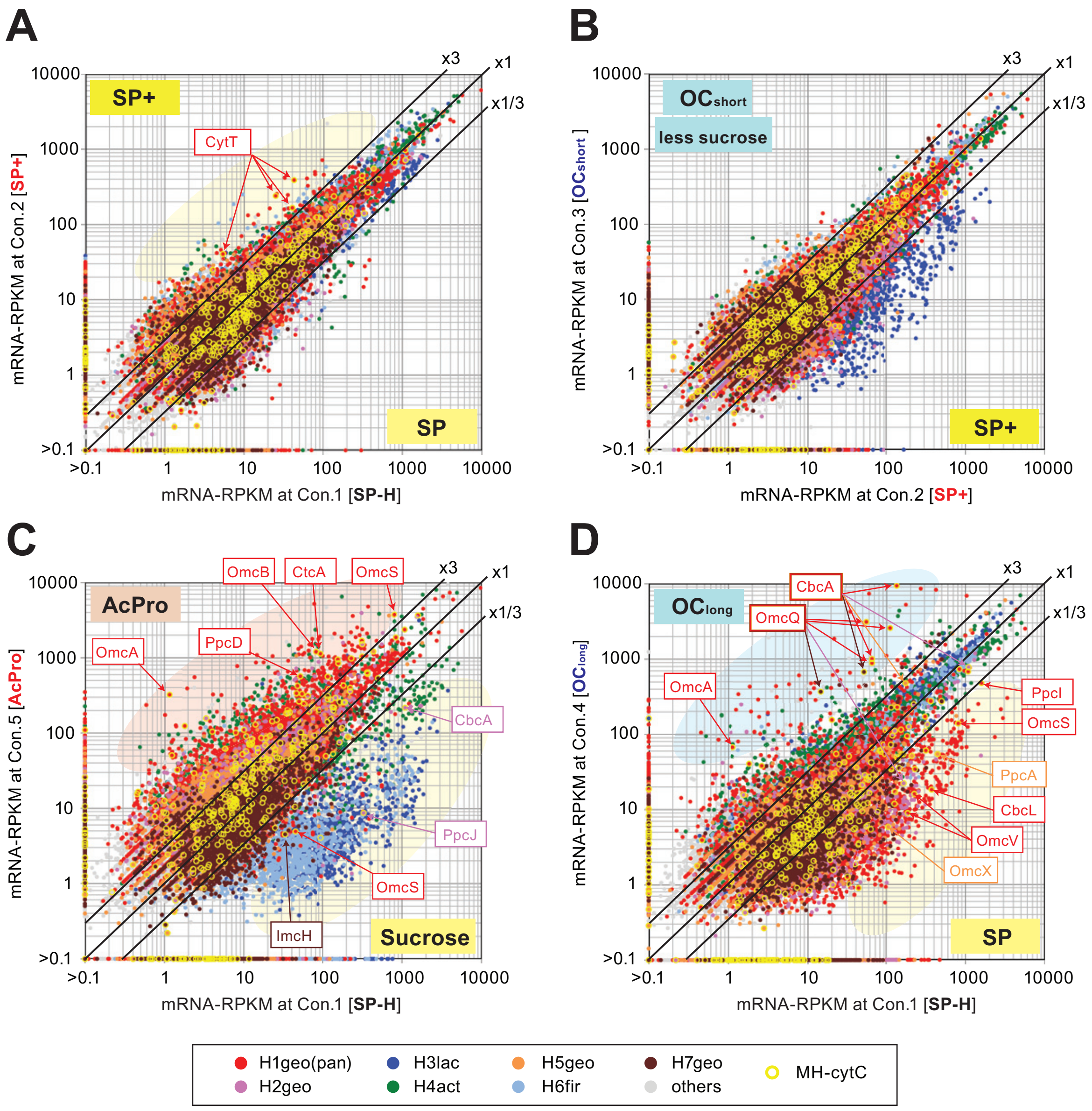

Supplementary Fig. S12 | Scatter plots comparing gene expression changes responsive to given EET stimuli in SP-H bin-genomes.

Scatter plots of all CDS responses as measured by mRNA frequency (mRNA-RPKM) for the SP-H electrogenic community between con.1 (SP-H) and con.2 (SP+) conditions in panel A, between con.2 (SP+) and con.3 (OC_{short}) conditions in panel B, between con.1 (SP-H) and con.5 (AcPro) in panel C, and between con.1 (SP-H) and con.4 (OC_{long}) in panel D. Highly abundant strains are shown as unique colors, all other groups are shown in gray. MH-cytC CDSs (CXXCH ≥2) are highlighted by yellow circle, and MH-cytCs with remarkable expression change are labeled with color of representative bin-genome.

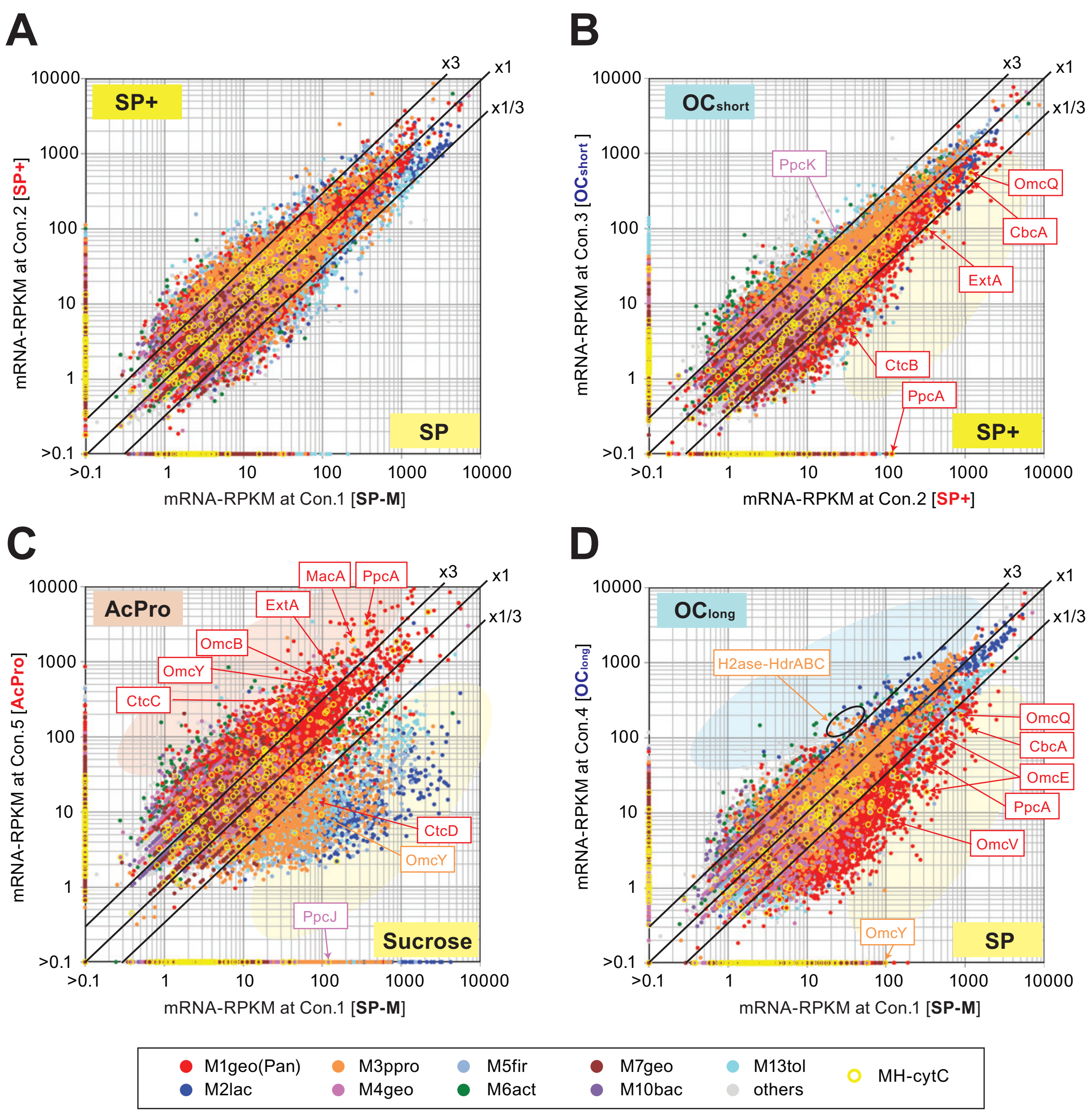

Supplementary Fig. S13 | Scatter plots comparing gene expression changes responsive to given EET stimuli in SP-M bin-genomes.

Scatter plots of all CDS responses as measured by mRNA frequency (mRNA-RPKM) for the SP-M electrogenic community between con.1 (SP-M) and con.2 (SP+) conditions in panel A, between con.2 (SP+) and con.3 (OC_{short}) conditions in panel B, between con.1 (SP-M) and con.5 (AcPro) in panel C, and between con.1 (SP-M) and con.4 (OC_{long}) in panel D. Highly abundant strains are shown as unique colors, all other groups are shown in gray. MH-cytC CDSs (CXXCH ≥2) are highlighted by yellow circle, and MH-cytCs with remarkable expression change are labeled with color of representative bin-genome.

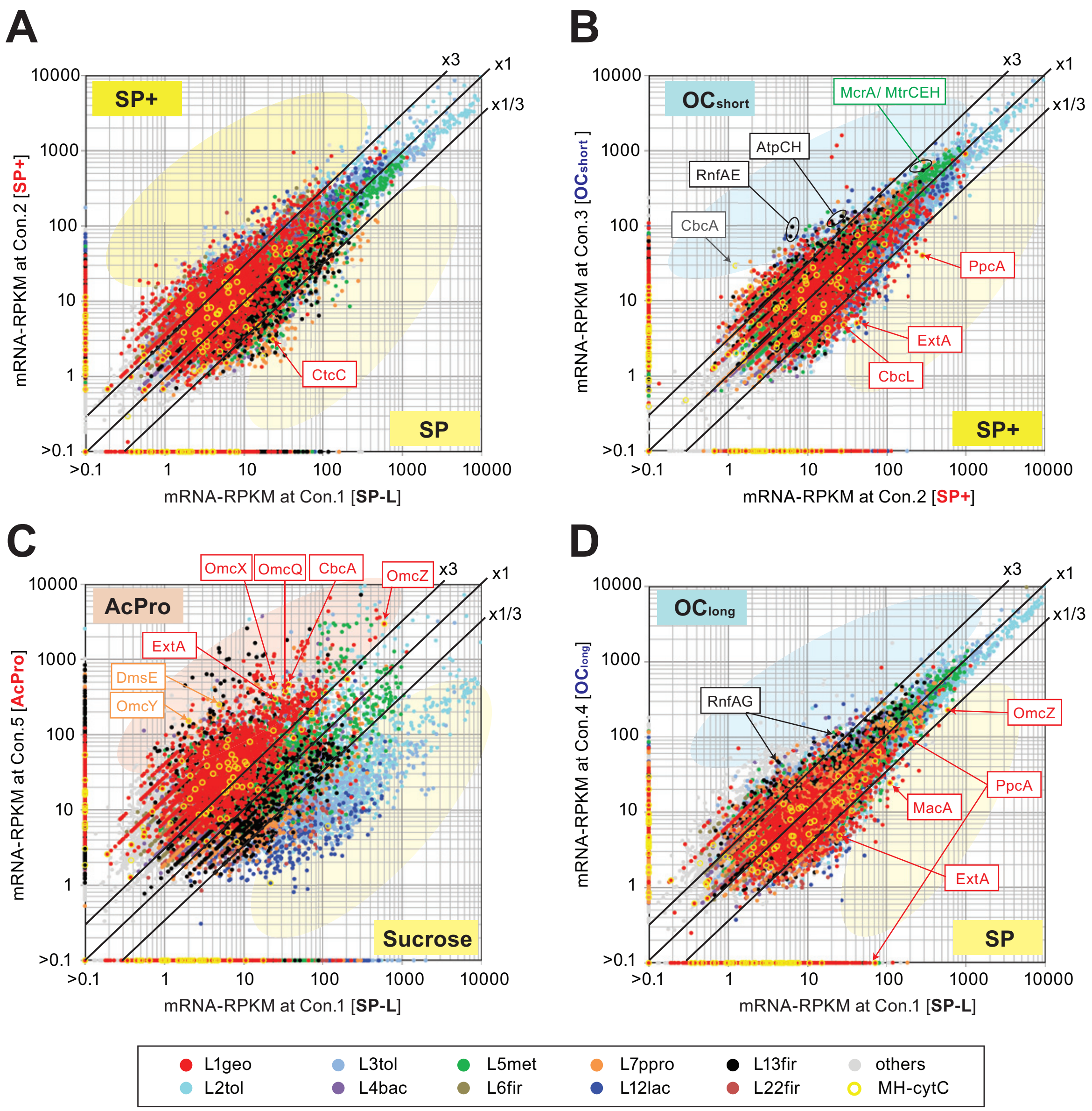

Supplementary Fig. S14 | Scatter plots comparing gene expression changes responsive to given EET stimuli in SP-L bin-genomes.

Scatter plots of all CDS responses as measured by mRNA frequency (mRNA-RPKM) for the SP-L electrogenic community between con.1 (SP-L) and con.2 (SP+) conditions in panel A, between con.2 (SP+) and con.3 (OC_{short}) conditions in panel B, between con.1 (SP-L) and con.5 (AcPro) in panel C, and between con.1 (SP-L) and con.4 (OC_{long}) in panel D. Highly abundant strains are shown as unique colors, all other groups are shown in gray. MH-cytC CDSs (CXXCH ≥2) are highlighted by yellow circle, and MH-cytCs with remarkable expression change are labeled with color of representative bin-genome.

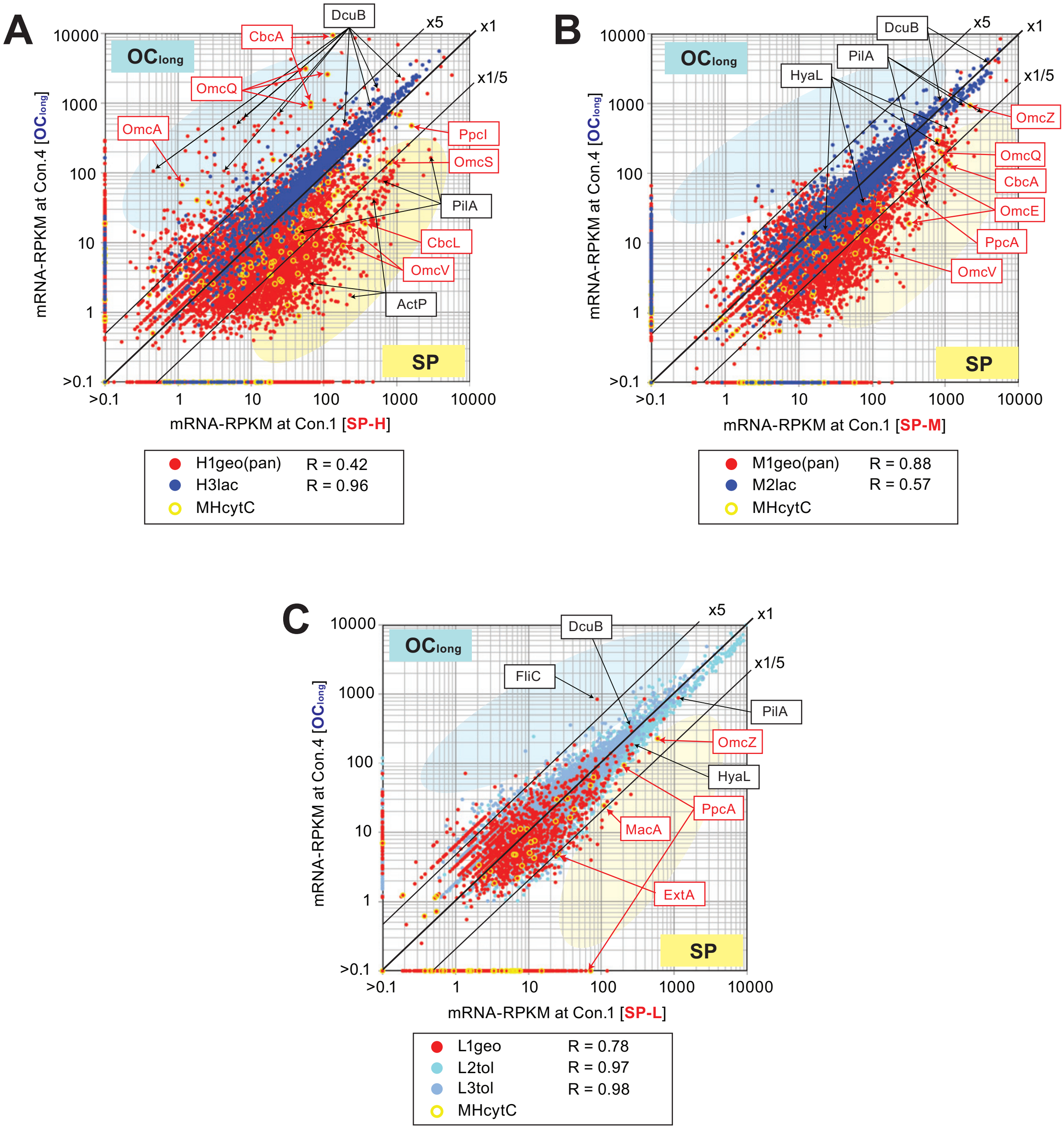

Supplementary Fig. S15 | Scatter plots comparing gene expression changes of dominant electrogenic and fermenter bin-genomes between EET and OC conditions.

Scatter plots of all CDS responses as measured by mRNA frequency (mRNA-RPKM) for dominant electrogenic and fermenter bin-genomes between con.1 (SP basal condition) and con.4 (OC_{long}). Panel A, OCSs H1geo(pan) and H3lac in the SP-H electrogenic community; panel B, OCSs M1geo(pan) and M2lac in the SP-M electrogenic community; and panel C, OCSs L1geo, L2tol, and L3tol in the SP-L electrogenic community. MH-cytC CDSs (CXXCH ≥2) are highlighted by yellow circle, and marker genes with remarkable expression change are labeled. Correlation coefficient (R) is shown next to the labels.

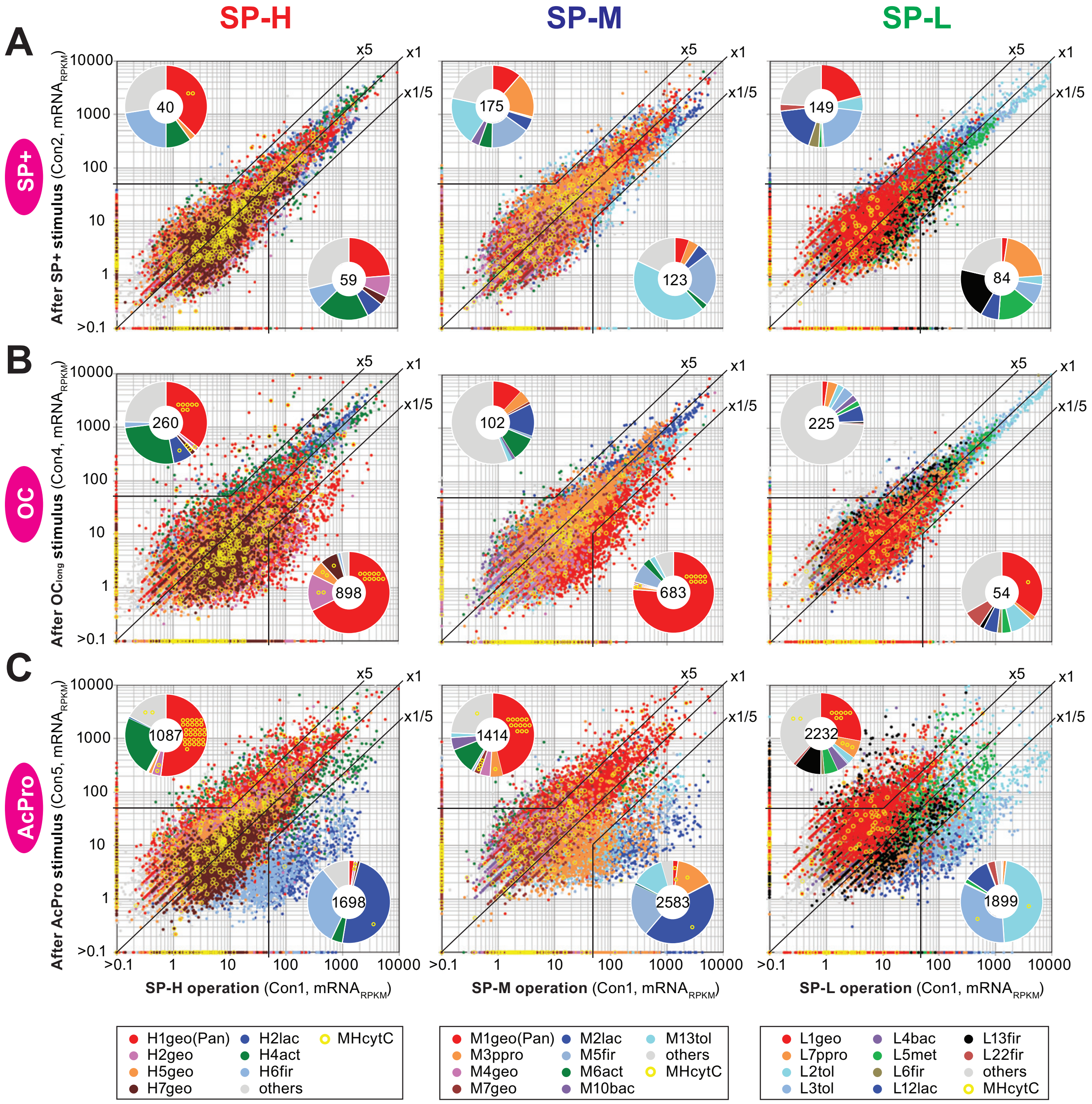

Supplementary Fig. S16 | Gene expression changes by three EET stimuli for three different communities.

Scatter plots of all CDS responses as measured by mRNA frequency (mRNA-RPKM) after SP+ stimulus (panels A), OC_{long} stimulus (panels B) or AcPro stimulus (panels C) compared to static EET operations of SP-H (left), SP-M (middle), or SP-L (right). Several dominant OCSs are shown as unique color dots, all other groups are shown in grey dots, and MH-cytCs are shown in yellow circles. CDSs that showed remarkabe responses (5-times change and mRNA-RPKM after/before stimulus over 50) to the given stimulus were summed relative to OCSs in ring charts and numbers of MH-cytCs (yellow circles). The ring charts of upper left showed up-regulation by the stimulus, while those of downer right showed down regulation by the stimulus in each scatter plot. The numbers of remarkably changed CDSs were shown in the center of rings.

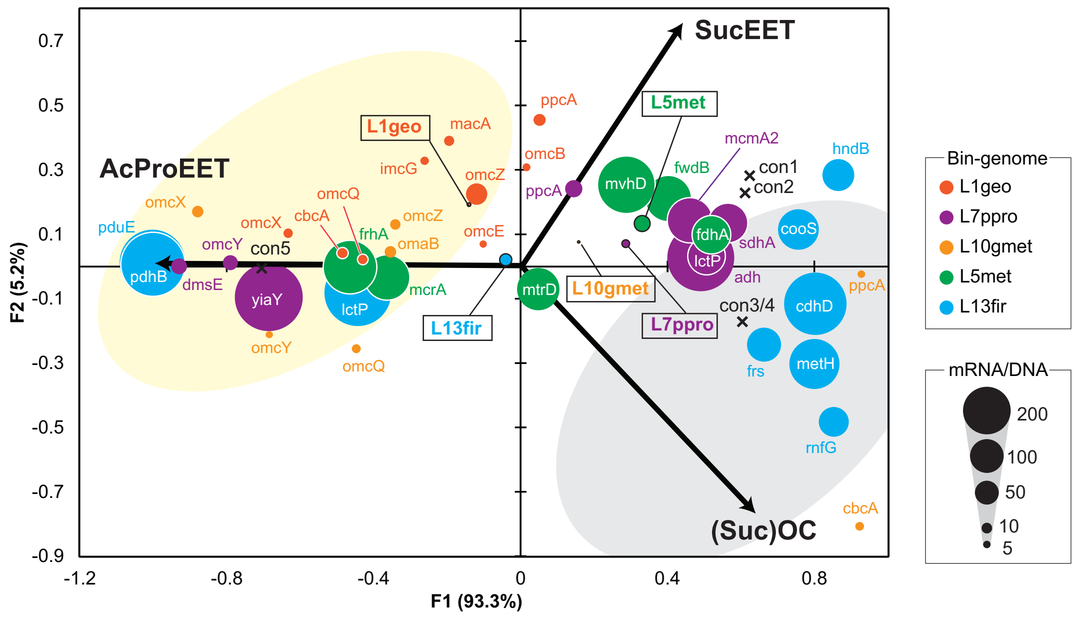

Supplementary Fig. S17 | wCCA diagrams comparing gene expression dynamics in five SP-L bin-genomes that reposonsive to EET stimuli.

Weighted canonical correspondence analysis (wCCA) diagram comparing gene expression dynamics of MH-cytCs for *Geonacteraceae* strain L1geo, L7ppro, L10gmet, methanogenesis genes for L5met, and reductive acetyl-coA pathway genes for L13fir under five operational conditions with the stilumi addition for electrogenic microbial communty SP-L. Size of dots indicates summed gene expression levels (mRNA-RPKM per DNA-RPKM) for the five conditions, while color of dots indicates bin-genomes. wCCA diagram showing the relationships between three operational variables (sucrose-consuming EET as SucEET, acetate/propionate-consuming EET as AcProEET, and open circuit as OC, black arrows), five operation conditions (cross marks), and highly expressed genes (dots) as well as whole gene expression (dots with black border) of the dominant microbes. Gene names were described near or within dots, while bin-genome IDs were described near dots with boxes.

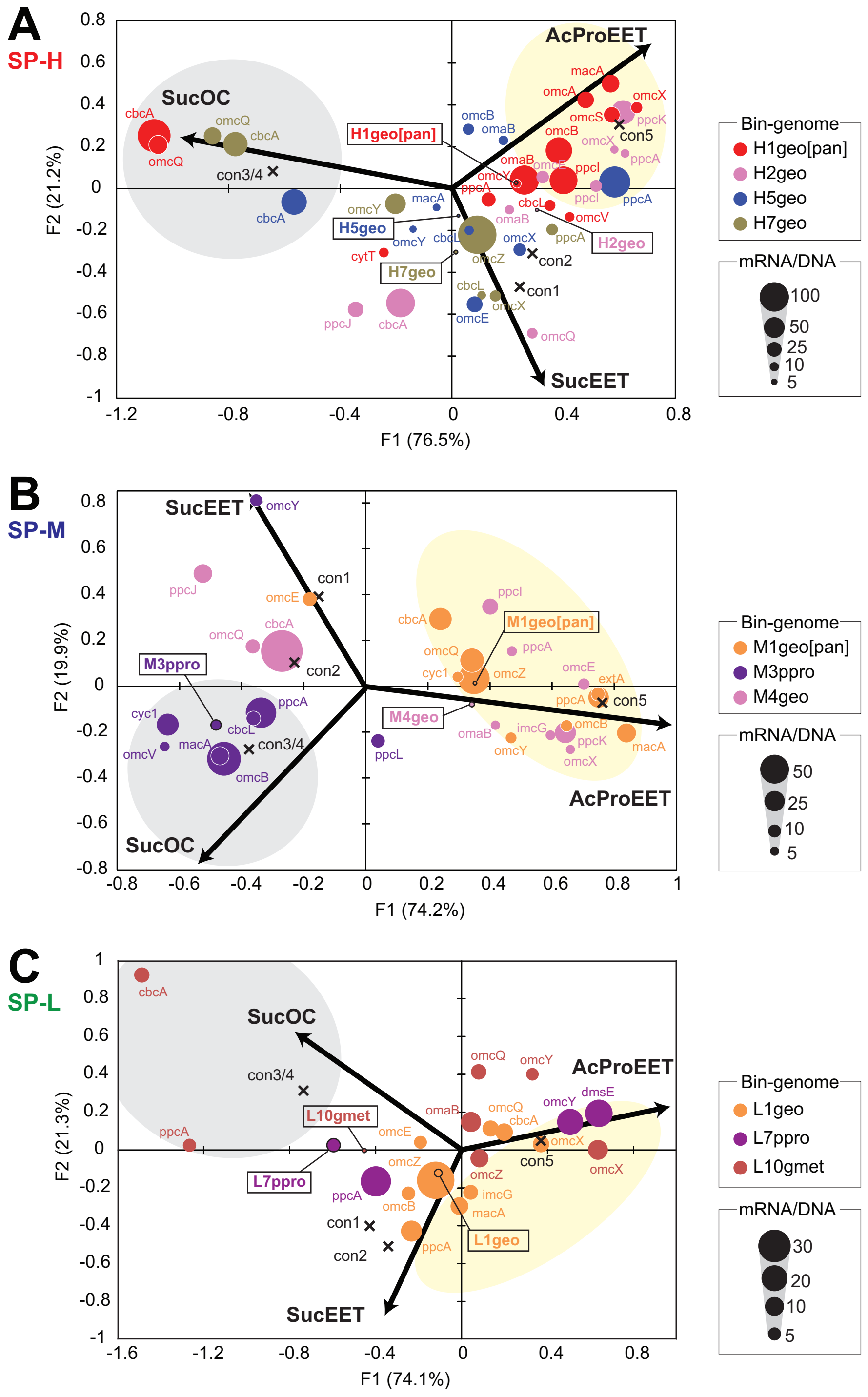

Supplementary Fig. S18 | wCCA diagrams comparing gene expression dynamics of multi-heme *c*-type cytochromes in dominant *Geobacteraceae* OCSs to the given stimuli.

Weighted canonical correspondence analysis (wCCA) diagram comparing gene expression dynamics of MH-cytCs under five operational conditions with the stilumi addition for three electrogenic microbial communties SP-H (A), SP-M (B) and SP-L (C). Size of dots indicates summed gene expression levels (mRNA-RPKM per DNA-RPKM) for the five conditions, while color of dots indicates bin-genomes. wCCA diagram showing the relationships between three operational variables (sucrose-consuming EET as SucEET, acetate/propionate-consuming EET as AcProEET, and open circuit as OC, black arrows), five operation conditions (cross marks), and highly expressed MH-cytCs (dots) as well as whole gene expression (dots with black border) of dominant microbes. MH-cytC IDs were described near dots, while bin-genome IDs were described near dots with boxes.

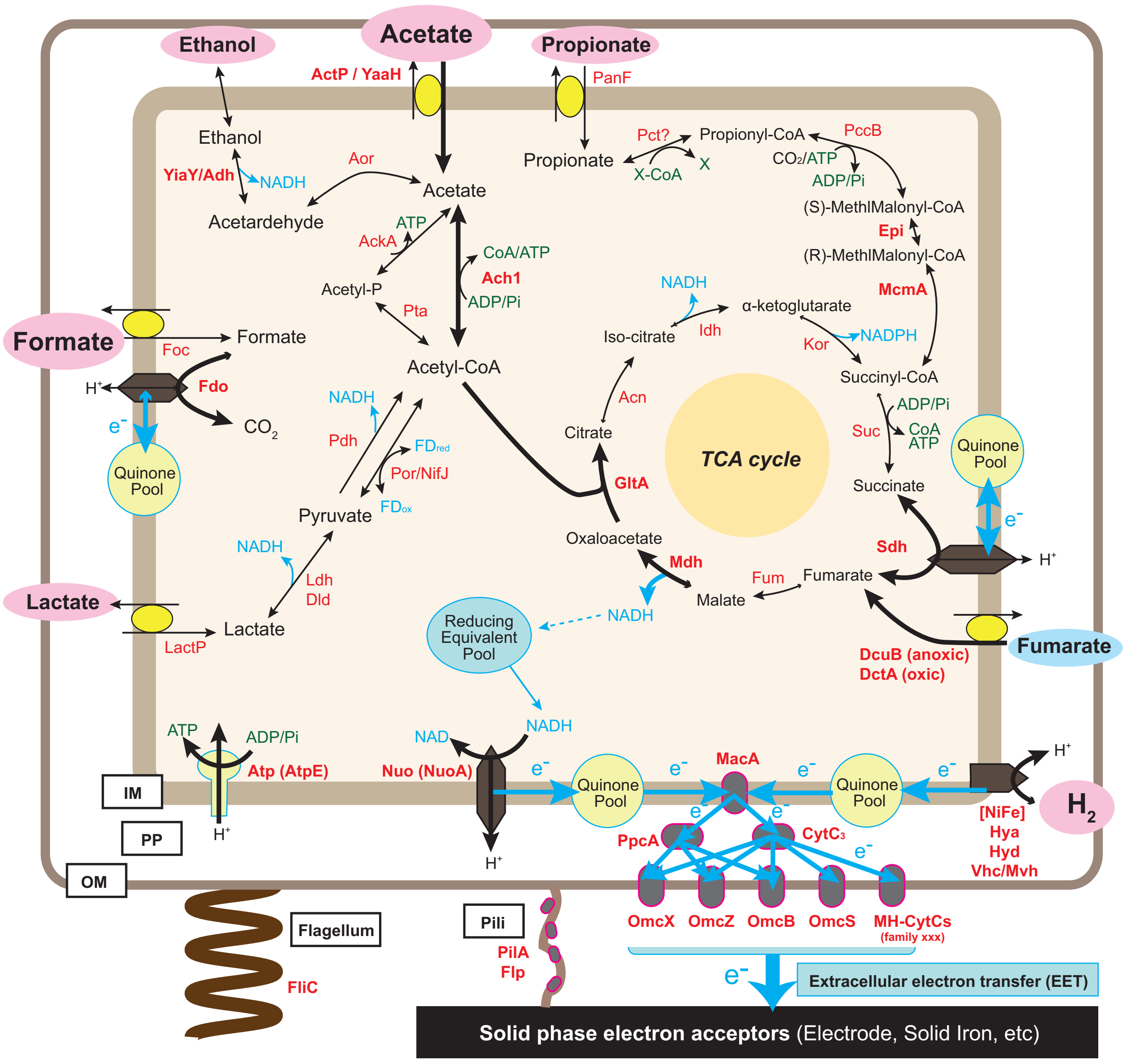

Supplementary Fig. S19 | EET-related metabolic pathway correlated with electron donors and accepters within *Geobacter* microbes.

Selected genes (bold red letter) and related metabolic functions (thick arrow) are used for comparative gene expression analyses to identify their responses to the given stimuli. ActP, cation/acetate symporter (K14393); YaaH, acetate transporter, putative (K07034); Ach1, acetyl-CoA hydrolase (K01067); GltA, citrate synthase (K01647); SdhA, succinate dehydrogenase flavoprotein subunit (K00239); Mdh, malate dehydrogenase (K00024); Epi, methylmalonyl-CoA epimerase (K05606); McmA2, methylmalonyl-CoA mutase, C-terminal domain (K01849); NuoA, NADH-quinone oxidoreductase subunit A (K00330); AtpE, F-type H^+-transporting ATPase subunit c (K02110); HyaL, hydrogenase large subunit (K06281); HyaS, hydrogenase small subunit (K06282); MvhD, F420-non-reducing hydrogenase iron-sulfur subunit D (K14127); HydB2, [NiFe] hydrogenase large subunit (K00437); FdoG, formate dehydrogenase, alpha subunit (K00123); HydA, ferredoxin hydrogenase (K00532); FdoH, formate dehydrogenase, beta subunit (K00124); Adh, alcohol dehydrogenase (K00001); YiaY, alcohol dehydrogenase (K13954); FliC, flagellin (K02406); PilA, type IV pilus assembly protein PilA (K02650); Flp; pilus assembly protein Flp/PilA (K02651); dctA; aerobic C4-dicarboxylate transport protein (K11103); DcuB, anaerobic C4-dicarboxylate transporter (K07792); MacA, cytochrome c peroxidase; PpcA/CytC$_3$, cytochrome c3; OmcX/OmcZ/OmcB/OmcS/MH-CytCs, outer membrane multi-heme *c*-type cytochrome proteins; e^-, electron; IM, inner membrane; PP, periplasm; OM, outer membrane.

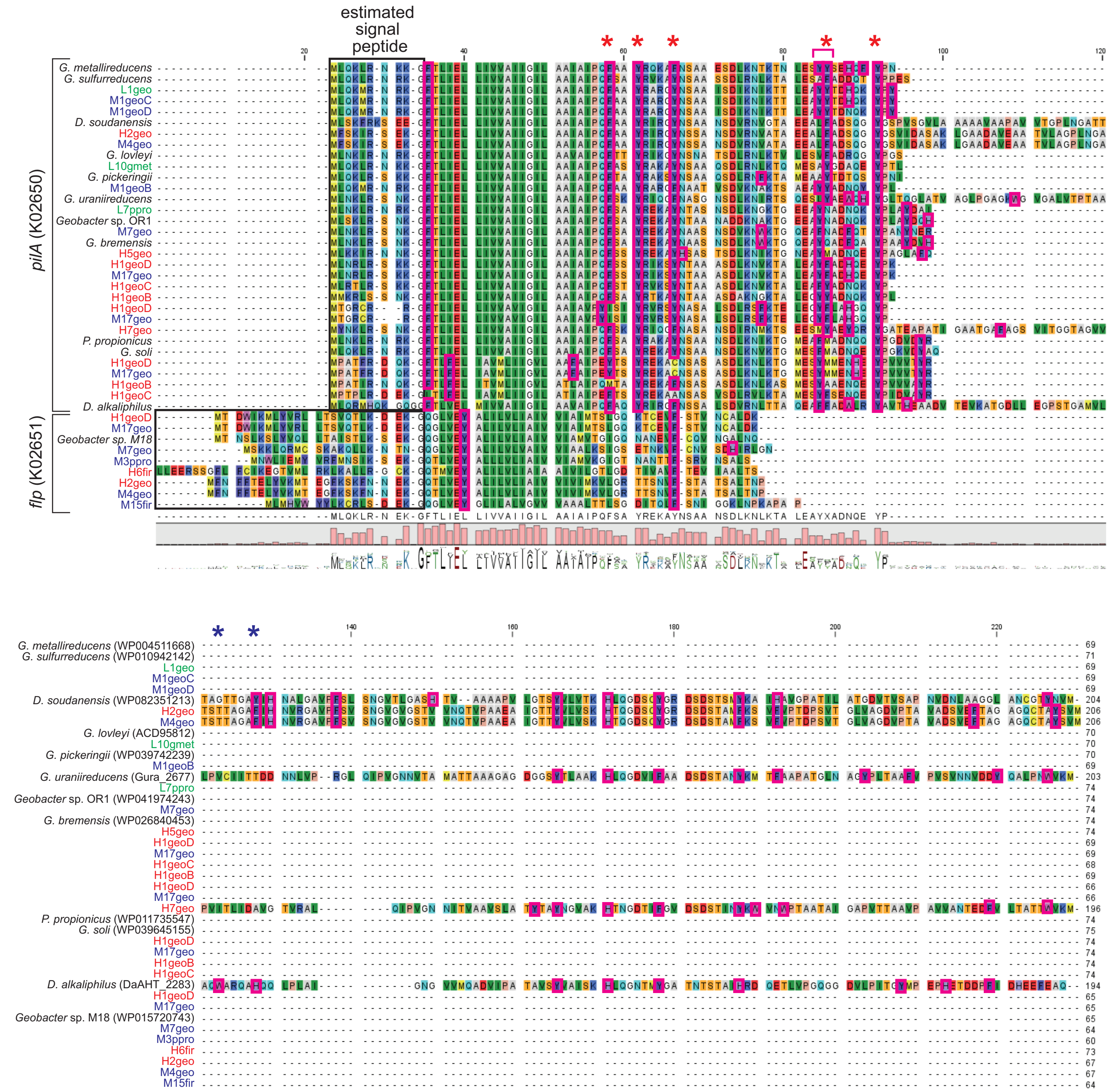

Supplementary Fig. S20 | Alignment of PilA and Flp amino acids sequences of *Geobacter/Pelobacter* bin-genomes and the reference strains.

Aromatic amino-acide (FYWH) placement is shown by purple rectangles, while estimated signal peptides for *pilA* (MLQKLRNRKG/ as cleavage site for model *G. sulfurreducens*) and *flp* (G/XXXXEY as cleavage site) are shown by black rectangles. Seven potential essential aromatic amino acids for conductive e-pili are marked by * above purple rectangles. Five essential aromatic amino acids were selected based on reduced EET mutanr strain Aro-5 (red *), while two of them were selected from the comparison between confirmed e-pili from *D. alkaliphilus* and non e-pili from *G. uranireducens* (blue *). The accesstion number of the reference sequences is only shown in parentheses of lower panel.

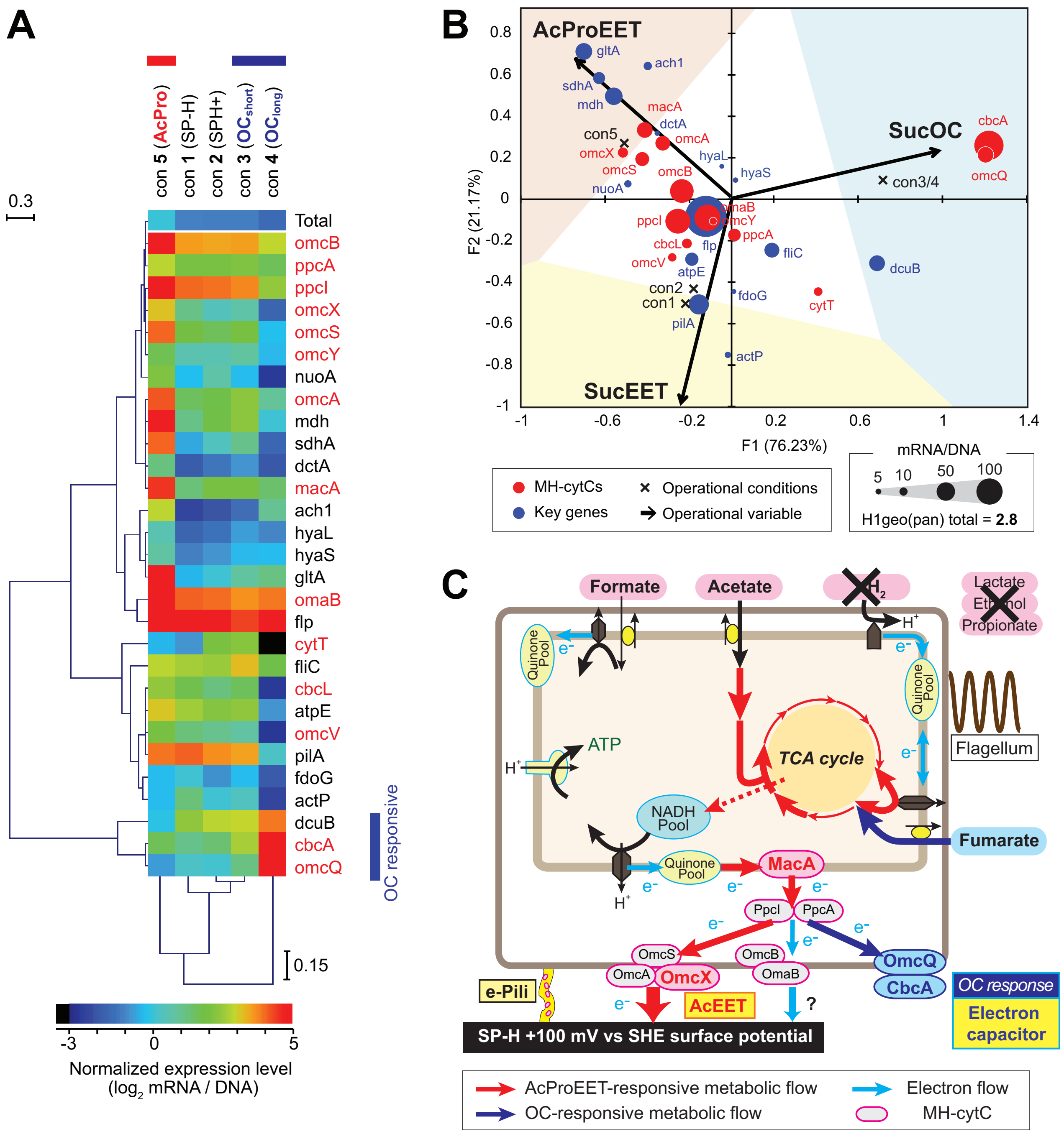

Supplementary Fig. S21 | Gene expression profiles and the stimuli responses of EET-related metabolic pathway of H1geo(pan) microbes.

(A) The gene expression levels of selected marker genes (refer to Supplementary Fig. S19) for each condition in the H1geo pan-genome. Genes coding multi-heme *c*-type cytochrome (MH-cytC) proteins are shown by red letter. **(B)** Weighted canonical correspondence analysis (wCCA) diagram shows relationship between marker genes (dots), five operational conditions (cross marks), and three operational variables (sucrose-consuming EET as SucEET, acetate/propionate-consuming EET as AcProEET, and open circuit as SucOC, black arrows). Size of dots indicates summed gene expression levels (mRNA-RPKM per DNA-RPKM) for the five conditions, while red dots indicate MH-cytCs and blue dots indicate key metabolic genes. **(C)** EET-related metabolic pathway map constructed by gene expression profiles in the H1geo(pan). The operational variable-associated metabolic flows and electron (e^-) flow are highlighted by thick colored arrows. MH-cytC proteins for EET reactions are shown as oval (IDs are described inside).

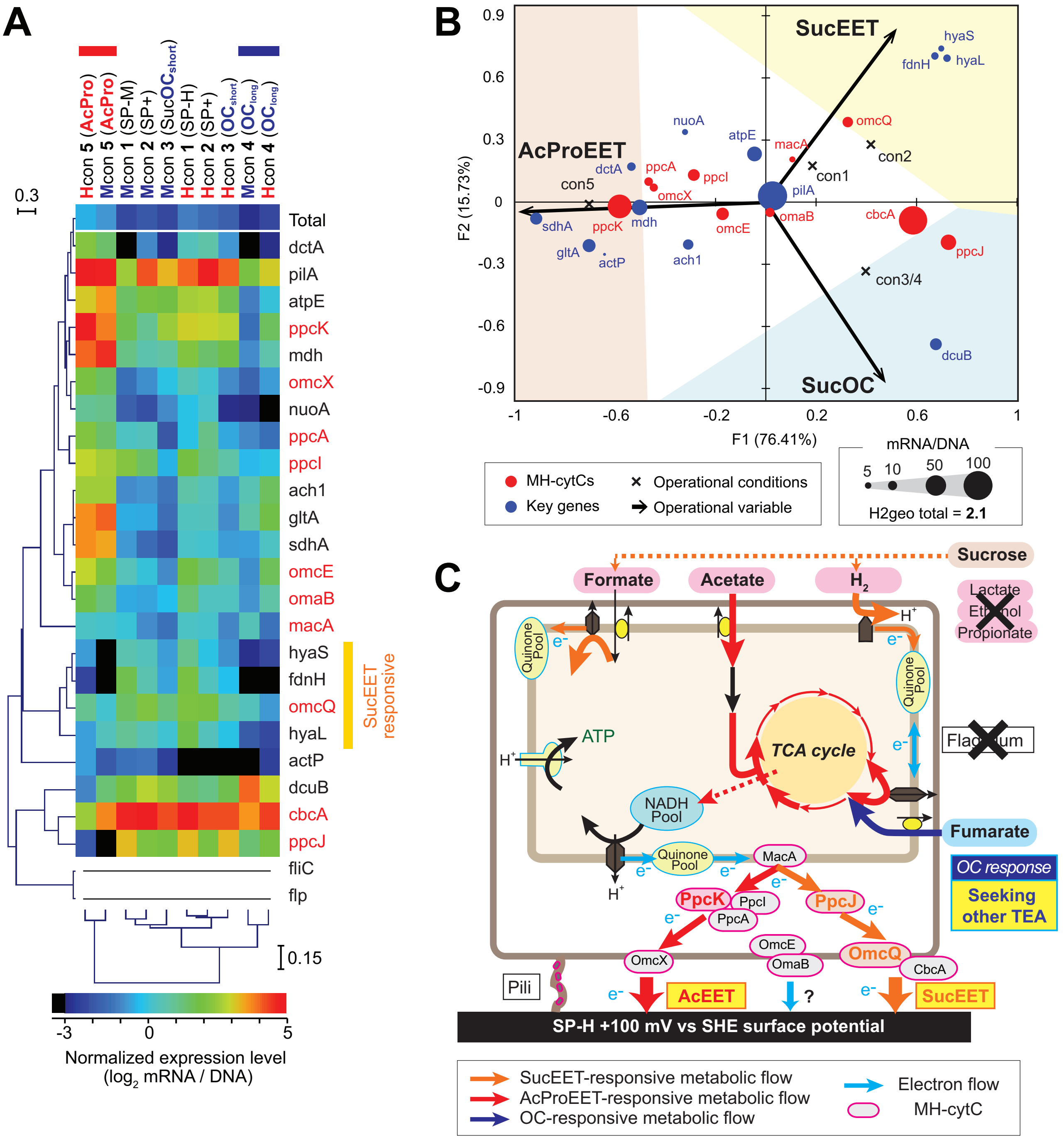

Supplementary Fig. S22 | Gene expression profiles and the stimuli responses of EET-related metabolic pathway of OCS H2geo/M4geo.

(A) The gene expression levels of selected marker genes (refer to Supplementary Fig. S19) for each condition in the OCS H2geo/M4geo. Genes coding multi-heme *c*-type cytochrome (MH-cytC) proteins are shown by red letter. The fliC and flp genes are not existed in the bin-genome. **(B)** Weighted canonical correspondence analysis (wCCA) diagram for only bin-genome H2geo shows relationship between marker genes (dots), five operational conditions (cross marks), and three operational variables (sucrose-consuming EET as SucEET, acetate/propionate-consuming EET as AcProEET, and open circuit as SucOC, black arrows). Size of dots indicates summed gene expression levels (mRNA-RPKM per DNA-RPKM) for the five conditions, while red dots indicate MH-cytCs and blue dots indicate key metabolic genes. The wCCA diagram for bin-genome M4geo is shown in Supplementary Fig. S25. **(C)** EET-related metabolic pathway map constructed by gene expression profiles in the OCS H2/M4geo. The operational variable-associated metabolic flows and electron (e^-) flow are highlighted by thick colored arrows. MH-cytC proteins for EET reactions are shown as oval (IDs are described inside).

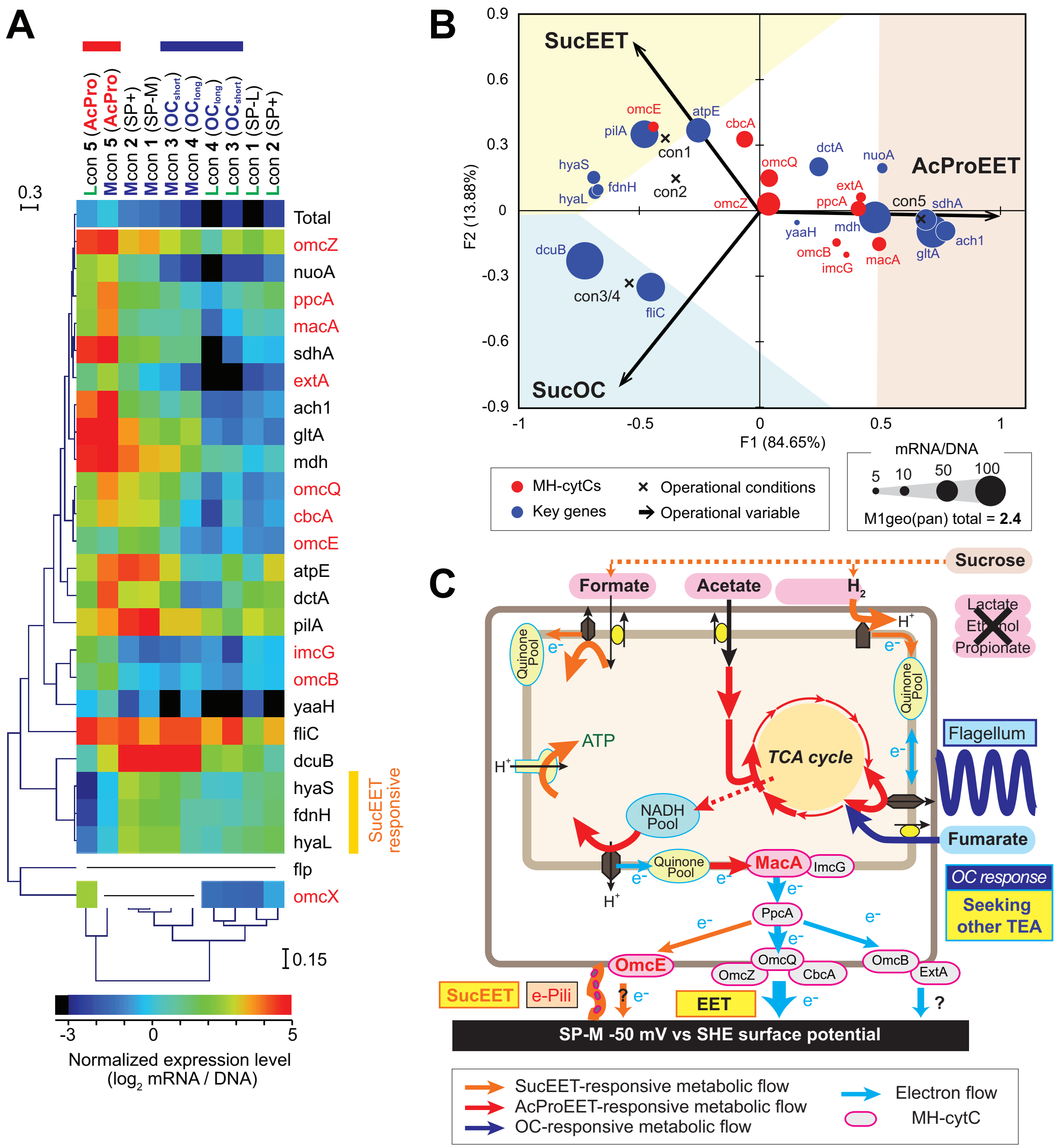

Supplementary Fig. S23 | Gene expression profiles and the stimuli responses of EET-related metabolic pathway of OCS L1geo/M1geo(pan).

(A) The gene expression levels of selected marker genes (refer to Supplementary Fig. S19) for each condition in the M1geo pan-genome and L1geo bin-genome. Genes coding multi-heme *c*-type cytochrome (MH-cytC) proteins are shown by red letter. The flp and omcX (only for L1geo) genes are not existed in the bin-genomes. **(B)** Weighted canonical correspondence analysis (wCCA) diagram for only OCS M1geo(pan) shows relationship between marker genes (dots), five operational conditions (cross marks), and three operational variables (sucrose-consuming EET as SucEET, acetate/propionate-consuming EET as AcProEET, and open circuit as SucOC, black arrows). Size of dots indicates summed gene expression levels (mRNA-RPKM per DNA-RPKM) for the five conditions, while red dots indicate MH-cytCs and blue dots indicate key metabolic genes. The wCCA diagram for OCS L1geo is shown in Supplementary Fig. S25. **(C)** EET-related metabolic pathway map constructed by gene expression profiles in the M1geo(pan)/L1geo microbe. The operational variable-associated metabolic flows and electron (e^-) flow are highlighted by thick colored arrows. MH-cytC proteins for EET reactions are shown as oval (IDs are described inside).

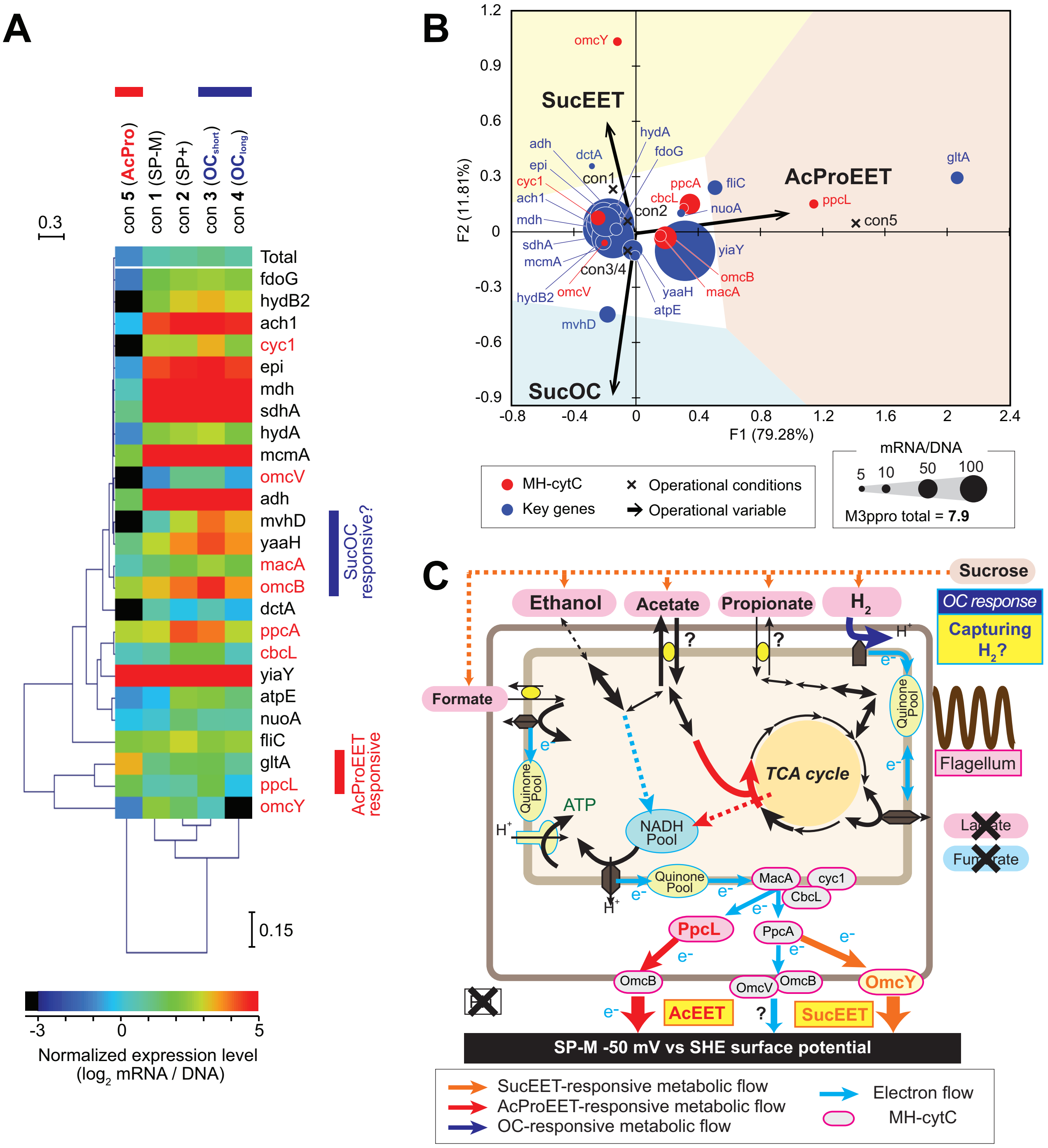

Supplementary Fig. S24 | Gene expression profiles and the stimuli responses of EET-related metabolic pathway of OCS M3ppro.

(A) The gene expression levels of selected marker genes (refer to Supplementary Fig. S19) for each condition in the M3ppro bin-genome. Genes coding multi-heme *c*-type cytochrome (MH-cytC) proteins are shown by red letter. **(B)** Weighted canonical correspondence analysis (wCCA) diagram shows relationship between marker genes (dots), five operational conditions (cross marks), and three operational variables (sucrose-consuming EET as SucEET, acetate/propionate-consuming EET as AcProEET, and open circuit as SucOC, black arrows). Size of dots indicates summed gene expression levels (mRNA-RPKM per DNA-RPKM) for the five conditions, while red dots indicate MH-cytCs and blue dots indicate key metabolic genes. **(C)** EET-related metabolic pathway map constructed by gene expression profiles in the OCS M3ppro. The operational variable-associated metabolic flows and electron (e^-) flow are highlighted by thick colored arrows. MH-cytC proteins for EET reactions are shown as oval (IDs are described inside).

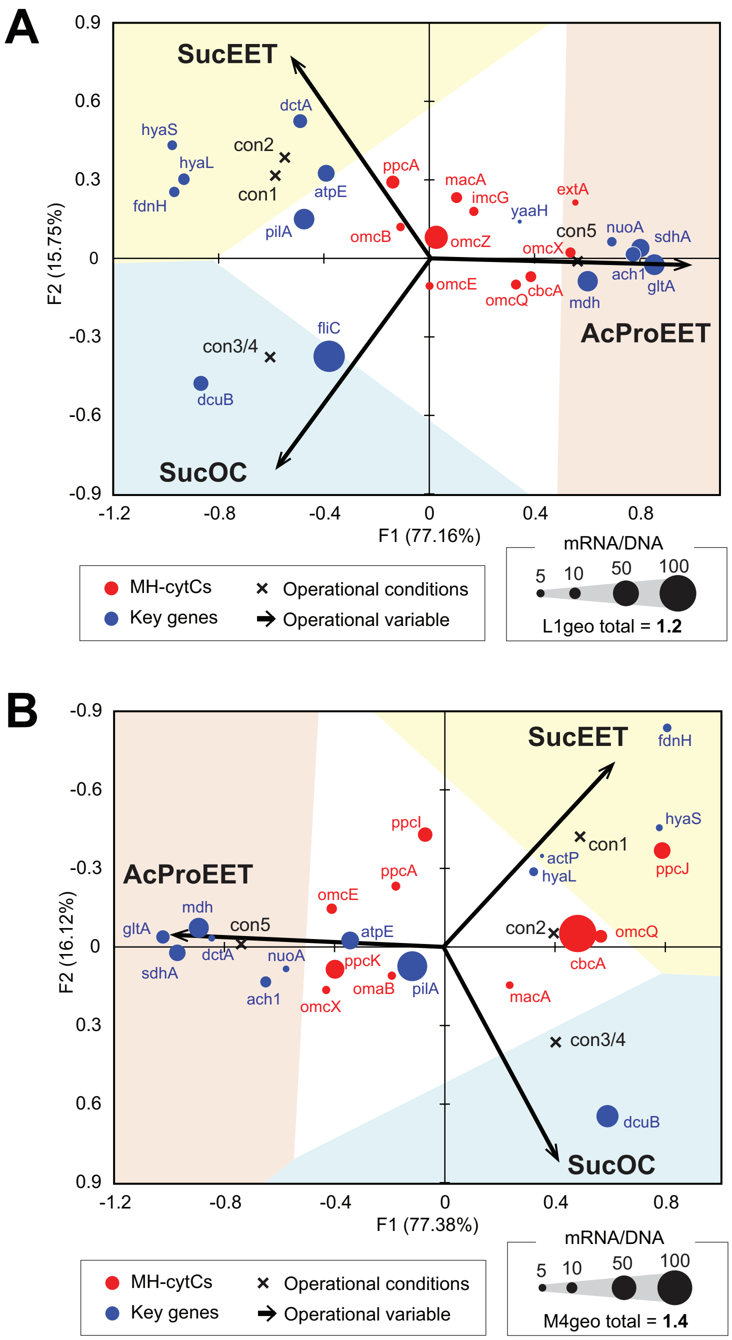

Supplementary Fig. S25 | Weighted canonical correspondence analysis diagram for OCS L1geo and OCS M4geo.

Weighted canonical correspondence analysis (wCCA) diagram for OCS L1geo (A) and OCS M4geo (B) shows relationship between marker genes (dots), five operational conditions (cross marks), and three operational variables (sucrose-consuming EET as SucEET, acetate/propionate-consuming EET as AcProEET, and open circuit as SucOC, black arrows). Size of dots indicates summed gene expression levels (mRNA-RPKM per DNA-RPKM) for the five conditions, while red dots indicate MH-cytCs and blue dots indicate key metabolic genes. The wCCA diagram for OCS M1geo(pan) that is same OCS of L1geo in SP-M biofilm is shown in Supplementary Fig. S23, while the wCCA diagram for OCS H2geo that is same OCS of M4geo in SP-H biofilm is shown in Supplementary Fig. S22.

Supplementary Table S1. Statistical parameters of metagenomic assemblies .

Community	kmer (Bubble size)	No. of raw reads[a]	Filtering[b]	Reads Mapped[a]	% Mapped[a]	No. Contigs	Total Bases (bp)	Max contig size (bp)	N50 (bp)	No. contigs >1 kbp
**SP-H**	53 bp (800 bp)	136,822,919	≥ 200 bp	133,327,400	97.5%	66,781	85,176,296	902,175	10,964	8,808
			≥ 500 bp	131,040,285	95.8%	19,400	70,873,878	902,175	22,768	8,808
			Bin-genomes	123,153,365	90.0%	3,870	49,410,450	902,382	48,747	3,361
**SP-M**	33 bp (800 bp)	103,431,637	≥ 200 bp	100,924,528	97.6%	88,351	121,290,853	876,693	7,832	15,304
			≥ 500 bp	99,439,728	96.1%	31,040	102,736,699	876,693	14,395	15,304
			Bin-genomes	95,180,985	92.0%	9,098	73,311,907	876,693	35,524	6,696
**SP-L**	23 bp (800 bp)	86,097,008	≥ 200 bp	83,818,222	97.4%	110,648	138,095,058	696,411	7,360	16,164
			≥ 500 bp	82,449,067	95.8%	40,757	115,007,795	696,411	16,307	16,164
			Bin-genomes	77,341,799	89.8%	6,261	75,303,664	696,411	42,736	4,814

[a] Numbers of reads to perform mapping raw reads back to the assembled contigs (with length cutoff 0.8 and similarity cutoff 0.8).

[b] Original parameter of length cut-off for CLC *de novo* assembly cell was 200 bp, while contig length cut-off of 500 bp was used for further metagenomic analyses. Stats of contigs associated with Bin-genomes for each community were also shown.

Supplementary Table S2. Summary of mRNA read counts for metatranscriptomic analyses.

Condition[a]		Time after stimulus	Total RNA reads	Total mRNA reads[b]	Mapped mRNA reads[c]			
					ORFs (%)[d]		Contigs (%)[e]	
**SP-H**								
con1	SP	2 hr	22,634,752	2,125,261	1,432,775	(67)	1,970,198	(93)
con2	SP+	45 min	24,744,087	1,845,776	1,246,473	(68)	1,696,388	(92)
con3	OC_{short}	45 min	20,492,732	1,570,703	1,066,465	(68)	1,414,017	(90)
con4	OC_{long}	2 hr	19,686,643	1,088,960	618,827	(57)	910,506	(84)
con5	AcPro	16 hr	18,329,605	1,245,790	847,595	(68)	1,122,232	(90)
**SP-M**								
con1	SP	2 hr	10,848,606	783,889	566,246	(72)	712,230	(91)
con2	SP+	45 min	10,960,172	1,240,115	781,360	(63)	1,059,975	(85)
con3	OC_{short}	45 min	11,961,906	1,113,151	707,397	(64)	958,208	(86)
con4	OC_{long}	2 hr	12,825,598	377,062	171,319	(45)	295,793	(78)
con5	AcPro	16 hr	13,152,708	887,317	610,414	(69)	765,433	(86)
**SP-L**								
con1	SP	3 hr	12,302,272	1,041,465	808,661	(78)	941,517	(90)
con2	SP+	45 min	12,655,429	680,113	524,394	(77)	614,565	(90)
con3	OC_{short}	45 min	12,484,744	733,830	554,784	(76)	663,430	(90)
con4	OC_{long}	3 hr	11,307,619	501,029	270,592	(54)	427,606	(85)
con5	AcPro	16 hr	11,524,413	595,758	451,531	(76)	533,517	(90)

[a] Con1-Con3 were short-term responses in same batch. Suc, sucrose-consuming; AcPro, acetate/propionate-consuming.

[b] rRNA was subtracted from unmapped RNA reads to ORFs using SILVA database, then total mRNA reads were calculated.

[c] mRNA was mapped to metagenomic contigs or ORFs with the parameter as 0.95 of identity and 0.6 of length coverage.

[d] Numbers of mRNA reads mapped to all metagenomic ORFs. Parenthesis indicates % mRNA mapped.

[d] Numbers of mRNA reads mapped to all metagenomic contigs. Parenthesis indicates % mRNA mapped.

Supplementary Table S3. Summary of SP-H Bin-genomes clustered from metagenomic assembly.

Bin-genome ID	*Taxa* [a]	SP-H frequency (%)[b]	Genome size (Mbp)	GC content (%)	Numbers of ORF[c]	COG- assigned ORFs	KO- assigned ORFs	Number of c-type cytochromes[d]	Number of contigs[e]	N50 (kbp)[e]	Longest contig (kbp)[e]	% Completeness[ef]	Average coverage of contigs[g]						
													Hcon1	Hcon2	Hcon3	Hcon4	Hcon5	Mcon1	Lcon1
**H1geoA**	*δ-Proteobacteria*	**-**	2.75	61	2960	2208	2164	64 (44)	586	7.0	27.4	**93**	77	82	66	293	66	1	0
**H1geoB**		**18.1**	1.85	60	2003	1292	1268	57 (35)	458	7.2	54.8	13	54	60	34	214	37	0	0
**H1geoC**		**13.3**	1.93	61	2121	1329	1287	60 (41)	642	4.7	80.8	15	33	32	46	110	40	0	0
**H1geoD**		**4.7**	3.25	61	3525	2480	2442	79 (44)	901	6.0	31.9	26	16	13	10	44	14	2	0
**H2geo**	*δ-Proteobacteria*	**14.8**	3.46	57	3098	2322	2212	99 (67)	**36**	**377.3**	**500.1**	**98**	42	36	46	137	40	15	0
**H3lac**	*Firmicutes*	**12.6**	1.91	38	1848	1476	1370	5 (2)	**54**	**63.7**	**136.0**	**98**	30	31	36	107	47	23	2
**H4act**	*Actinomycetes*	**11.1**	3.42	70	3271	2469	2257	10 (1)	**74**	**100.2**	**208.3**	**97**	33	33	36	80	29	9	0
**H5geo**	*δ-Proteobacteria*	**7.2**	4.23	54	3684	2740	2621	104 (70)	**35**	**357.5**	**602.7**	**98**	18	21	15	73	24	0	0
**H6fir**	*Firmicutes*	**4.7**	3.13	50	3002	2458	2298	14 (0)	**197**	29.2	**135.6**	**98**	11	11	13	35	20	6	0
**H7geo**	*δ-Proteobacteria*	**4.6**	3.47	51	3191	2470	2368	85 (50)	**107**	**60.0**	**281.2**	**95**	11	12	10	35	20	3	0
**H8spi**	*Spirochaeta*	**2.8**	3.22	59	2939	2206	2054	10 (0)	**35**	**200.4**	**450.3**	**96**	9	9	7	21	8	5	7
**H9bac**	*Bacteroidetes*	**1.5**	3.28	39	2706	1820	1725	7 (0)	**66**	**83.7**	**307.7**	**98**	5	4	4	10	5	0	1
**H10eps**	*ε-Proteobacteria*	**1.3**	2.72	39	2712	2183	2084	55 (20)	**22**	**216.0**	**902.4**	**99**	4	4	4	8	4	3	0
**H11bac**	*Bacteroidetes*	**0.8**	4.25	39	3275	2180	2130	17 (5)	**191**	31.1	**110.8**	**97**	3	2	2	4	2	1	1
**H12unc**	*Acidobacteria*	**1.0**	4.55	68	4398	3121	2884	31 (16)	311	23.4	**113.0**	**97**	3	3	2	6	4	1	5
H3lacP	Plasmid	-	0.28	34	341	128	109	0 (0)	28	18.6	25.7	0	18	19	23	65	27	23	1
H4actB	Plasmid?	-	1.02	66	1021	502	444	3 (0)	109	27.5	82.2	3	15	16	17	38	14	3	0
H6firP	Plasmid	-	0.14	42	137	85	77	1 (0)	11	38.3	43.6	0	10	11	12	34	18	0	0
H6firP2	Plasmid	-	0.05	45	51	33	30	0 (0)	4	17.4	19.9	0	11	11	13	35	19	1	0
H7geoP	Plasmid	-	0.52	48	566	233	229	6 (2)	30	99.8	**199.0**	3	9	10	8	23	14	0	0
TOTAL in Bin-genomes		**98.5**	49.44	-	46849	33735	32053	707 (397)	3897	48.7	902.4	-	411	420	403	1374	450	97	18
TOTAL in Sp-H metagenome		**100.0**	70.87	-	82776	53951	52553	911 (478)	19400	22.8	902.4	-	na	na	na	na	na	na	na

[a] Taxonomy used for bin-genome clustering. Clusters potentially assigned as plasmid were separated based on their coverage differences and less KO assignmenet.

[b] Relative frequency within the SP-H metagenome was calculated based on the coverage of 17 universal single-copied core genes. Micro-diversity of H1geoA-D was separately considered as to one core gene group (H1geoA) and three substrains (H1geoB-D).

[c] Numbers of ORFs have potential errors in metagenomic ORF calling because of incomplete assemblies.

[d] Number of ORFs assigned as *c*- type cytochrome by CXXCH domain search. Numbers of multi-heme *c*- type cytochromes are descrined in parentheses.

[e] Bold letters indicate well-clusterred bin-genomes in terms of the parameters.

[f] Values were calculated from frequency of KO assignment to universal single-copied gene family lists (Supplementary Table S5).

[g] Raw reads for each condition were separately mapped to SP-H contigs by using similarity cut-off by 0.95 and length cut-off by 0.7.

Supplementary Table S4. Summary of SP-M Bin-genomes clustered from metagenomic assembly.

Bin-genome ID	*Taxa* [a]	SP-M frequency (%)[b]	Genome size (Mbp)	GC content (%)	Numbers of ORF[c]	COG- assigned ORFs	KO- assigned ORFs	Number of c-type cytochromes[d]	Number of contigs[e]	N50 (kbp)[e]	Longest contig (kbp)[e]	% Complete-ness[ef]	Average coverage of contigs[g]						
													Mcon1	Mcon2	Mcon3	Mcon4	Mcon5	Hcon1	Lcon1
**M1geoA**	*δ-Proteobacteria*	**-**	3.01	55	2915	2176	2037	71 (44)	287	18.6	54.3	88	123	117	127	137	128	0	103
**M1geoB**		**32.1**	1.63	53	1675	962	877	47 (31)	337	9.5	76.9	8	98	75	93	100	107	0	18
**M1geoC**		**14.5**	1.39	54	1449	771	736	38 (28)	421	6.1	38.7	9	33	55	47	49	30	0	127
**M1geoD**		**6.2**	2.17	54	2235	1316	1199	49 (30)	465	8.8	61.5	19	15	30	15	22	8	0	4
**M2lac**	*Firmicutes*	**12.7**	2.24	36	2226	1613	1451	7 (2)	**66**	**124.7**	**205.1**	**98**	27	40	36	57	40	28	1
**M3ppro**	*δ-Proteobacteria*	**6.4**	4.89	52	4511	3184	2844	59 (27)	**109**	**102.9**	**254.3**	**95**	15	12	17	22	28	0	0
**M4geo**	*δ-Proteobacteria*	**4.8**	3.46	56	3102	2320	2136	99 (67)	**42**	**442.8**	**876.7**	**98**	17	15	19	23	15	45	0
**M5fir**	*Firmicutes*	**3.2**	3.46	50	3283	2583	2317	19 (0)	**37**	**151.1**	**295.3**	**97**	7	8	9	12	13	9	0
**M6act**	*Actinomycetes*	**3.5**	3.31	70	3182	2410	2136	10 (1)	217	26.7	78.3	**97**	9	12	11	8	9	32	0
**M7geo**	*δ-Proteobacteria*	**1.8**	4.81	57	4223	3064	2827	107 (64)	**90**	**113.7**	**326.9**	**96**	3	4	5	1	10	2	0
**M8spi**	*Spirochaeta*	**2.0**	3.20	61	2874	2189	1969	10 (0)	**48**	**114.0**	**302.6**	**95**	6	5	6	6	5	9	8
**M9unc**	*Acidobacteria*	**1.3**	3.26	68	3131	2353	2078	40 (15)	**126**	44.9	**155.6**	87	3	4	6	1	6	0	0
**M10bac**	*Bacteroidetes*	**1.7**	3.56	45	3037	2169	2012	7 (0)	**189**	44.0	94.2	**99**	3	2	3	4	4	0	7
**M11eps**	*ε-Proteobacteria*	**1.1**	2.78	38	2797	2218	2016	53 (18)	**30**	**159.5**	**531.5**	**99**	3	3	3	3	4	3	0
**M12geo**	*δ-Proteobacteria*	**1.4**	3.72	51	3692	2772	2527	90 (56)	**78**	**98.0**	**284.1**	**96**	3	1	3	3	5	10	0
**M13tol**	*γ-Proteobacteria*	**1.9**	2.96	49	2766	2399	2293	9 (1)	**64**	**114.1**	**212.7**	**98**	4	1	6	9	5	0	8
**M14spi**	*Spirochaeta*	**1.0**	3.11	61	2954	2290	2078	12 (2)	**147**	34.9	**102.9**	**94**	4	3	3	3	2	0	0
**M15fir**	*Firmicutes*	**0.7**	4.44	61	4820	3531	3197	22 (2)	412	17.0	60.5	**96**	2	2	2	2	2	0	0
**M17geo**	*δ-Proteobacteria*	**0.4**	4.55	59	5327	3784	3626	112 (66)	1051	7.2	41.4	63	2	1	2	1	2	39	0
**M18tol**	*γ-Proteobacteria*	**0.1**	2.11	48	3324	2543	2541	5 (0)	1607	1.5	11.3	49	1	0	1	1	2	1	51
**M19bac**	*Bacteroidetes*	**0.4**	3.84	39	3393	2202	2063	17 (5)	704	7.8	29.7	**92**	1	1	1	1	1	3	1
**M20fir**	*Firmicutes*	**0.1**	0.85	54	1132	851	781	2 (0)	323	3.9	13.4	29	1	1	1	2	1	0	1
**M21unc**	Unclassified	**0.2**	2.69	68	4182	2665	2370	25 (12)	1952	1.6	8.2	63	1	1	1	1	1	3	5
M5firP	Firmicutes	-	0.06	48	69	25	23	1 (0)	3	30.4	30.4	0	8	8	11	13	14	0	0
M16actB	Actinomycetes	-	1.34	68	1363	713	584	1 (0)	291	8.5	57.7	3	4	5	4	3	4	8	0
TOTAL in Bin-genomes		**97.6**	72.82	-	73662	53103	48718	912 (471)	9096	35.5	876.7	-	394	403	432	485	447	192	336
TOTAL in Sp-M metagenome		**100.0**	102.74	-	119295	80485	74089	1114 (549)	31040	14.4	876.7	-	na	na	na	na	na	na	na

[a] Taxonomy used for bin-genome clustering. Clusters potentially assigned as plasmid were separated based on their coverage differences and less KO assignmenet.

[b] Relative frequency within the SP-M metagenome was calculated based on the coverage of 17 universal single-copied core genes. Micro-diversity of M1geoA-D was separately considered as to one core gene group (M1geoA) and three substrains (M1geoB-D).

[c] Numbers of ORFs have potential errors in metagenomic ORF calling because of incomplete assemblies.

[d] Number of ORFs assigned as *c*- type cytochrome by CXXCH domain search. Numbers of multi-heme *c*- type cytochromes are descrined in parentheses.

[e] Bold letters indicate well-clusterred bin-genomes in terms of the parameters.

[f] Values were calculated from frequency of KO assignment to universal single-copied gene family lists (Supplementary Table S6).

[g] Raw reads for each condition were separately mapped to SP-M contigs by using similarity cut-off by 0.95 and length cut-off by 0.7.

Supplementary Table S5. Summary of SP-L Bin-genomes clustered from metagenomic assembly.

Bin-genome ID	*Taxa* [a]	SP-L frequency (%)[b]	Genome size (Mbp)	GC content (%)	Numbers of ORF[c]	COG- assigned ORFs	KO- assigned ORFs	Number of c-type cytochromes[d]	Number of contigs[e]	N50 (kbp)[e]	Longest contig (kbp)[e]	% Completeness[ef]	Average coverage of contigs[g]						
													Lcon1	Lcon2	Lcon3	Lcon4	Lcon5	Hcon1	Mcon1
**L1geo**	*δ-Proteobacteria*	**24.3**	4.44	54	3845	2792	2582	106 (70)	**42**	**268.4**	**696.4**	**95**	159	102	61	65	68	0	92
**L2tol**	*γ-Proteobacteria*	**28.9**	3.41	49	3169	2692	2543	8 (1)	**26**	**279.8**	**525.8**	**99**	69	119	135	106	86	1	1
**L3tol**	*γ-Proteobacteria*	**7.7**	3.47	45	3170	2659	2528	11 (1)	**63**	**147.7**	**358.1**	73	19	19	30	27	22	0	0
**L4bac**	*Bacteroidetes*	**5.2**	3.40	42	2846	2128	1947	18 (5)	361	16.6	62.3	**94**	8	12	15	16	14	0	0
**L5met**	*Euryarchaeota*	**4.4**	1.47	51	1670	1305	1172	7 (0)	225	11.6	45.9	**94**	6	13	19	9	6	0	0
**L6fir**	*Firmicutes*	**4.4**	1.12	35	1056	774	705	5 (0)	**15**	**143.9**	**253.4**	**95**	20	11	16	17	22	0	0
**L7ppro**	*δ-Proteobacteria*	**2.7**	3.82	58	3435	2646	2391	25 (11)	**84**	**82.8**	**347.8**	88[h]	4	10	12	12	12	0	0
**L8spi**	*Spirochaeta*	**3.1**	2.84	61	2600	2012	1816	7 (0)	**102**	45.5	**140.7**	**95**	9	9	9	6	5	4	3
**L9bac**	*Bacteroidetes*	**2.8**	3.96	45	3305	2290	2099	7 (0)	**24**	**228.4**	**556.0**	**99**	8	9	9	6	6	0	3
**L10gmet**	*δ-Proteobacteria*	**2.1**	3.51	57	3216	2445	2235	96 (61)	**91**	**77.0**	**222.8**	**97**	2	11	8	4	2	0	0
**L11unc**	*Acidobacteria*	**2.1**	4.50	68	3973	2947	2598	33 (16)	**91**	**90.6**	**319.3**	**95**	6	8	8	7	6	3	1
**L12lac**	*Firmicutes*	**2.2**	1.95	38	1905	1489	1361	7 (2)	**69**	**55.5**	**188.4**	**97**	2	3	4	15	18	28	21
**L13fir**	*Firmicutes*	**1.1**	3.43	45	3234	2601	2319	16 (0)	**177**	35.2	**173.9**	**98**	2	3	3	4	8	0	0
**L14bac**	*Bacteroidetes*	**1.4**	3.90	39	3230	2237	2053	9 (3)	308	28.8	98.8	**96**	2	3	4	4	5	0	0
**L15bac**	*Bacteroidetes*	**0.9**	3.13	37	2586	1770	1609	12 (2)	**25**	**264.4**	**437.3**	**97**	2	2	2	5	4	0	0
**L16bac**	*Bacteroidetes*	**0.7**	3.94	39	3092	2098	1943	17 (5)	251	22.7	75.3	**92**	1	2	2	2	2	3	1
**L17bac**	*Bacteroidetes*	**0.6**	3.85	37	3311	2243	2120	11 (2)	326	20.0	**107.8**	86	2	1	1	2	4	0	0
**L18ver**	*Verrucomicrobia*	**0.7**	6.49	57	5195	3458	3087	51 (20)	411	26.4	**102.5**	**92**	3	2	3	1	3	0	0
**L19bac**	*Bacteroidetes*	**0.3**	3.33	38	3323	2063	1892	9 (2)	725	7.1	42.7	80	1	1	1	1	1	0	0
**L20tol**	*γ-Proteobacteria*	**0.1**	1.54	41	2725	2004	2115	4 (1)	1133	1.6	14.2	29	3	1	1	1	3	0	3
**L20tolB**	*γ-Proteobacteria*	**0.0**	1.06	42	1934	1359	1399	2 (0)	860	1.3	27.1	17	2	1	1	1	2	0	0
**L21met**	*Euryarchaeota*	**0.3**	1.00	50	1336	958	855	7 (0)	416	3.4	16.3	40	2	3	4	2	1	0	0
**L22fir**	*Firmicutes*	**1.3**	2.38	40	2335	1899	1728	11 (0)	**43**	**143.9**	**363.6**	**96**	3	3	4	5	7	0	0
**L23elu**	*Elusimicrobia*	**0.7**	1.39	44	1312	993	884	3 (1)	**79**	30.0	65.1	**92**	2	1	1	4	3	0	0
**L24fir**	*Firmicutes*	**0.3**	1.78	39	1906	1319	1166	8 (0)	300	7.9	24.3	87	2	1	1	1	1	0	0
L12lacP	Plasmid	-	0.25	34	300	123	86	1 (0)	39	13.0	25.6	0	1	1	2	6	6	20	26
TOTAL in Bin-genomes		**98.2**	75.36	-	70009	51304	47233	489 (201)	6261	42.7	696.4	-	336	351	356	329	317	61	152
TOTAL in Sp-L metagenome		**100.0**	115.01	-	132905	88116	81603	684 (238)	40757	16.3	696.4	-	na	na	na	na	na	na	na

[a] Taxonomy used for bin-genome clustering. Clusters potentially assigned as plasmid were separated based on their coverage differences and less KO assignmenet.
[b] Relative frequency within the SP-L metagenome was calculated based on the coverage of 17 universal single-copied core genes.
[c] Numbers of ORFs have potential errors in metagenomic ORF calling because of incomplete assemblies.
[d] Number of ORFs assigned as *c*-type cytochrome by CXXCH domain search. Numbers of multi-heme *c*-type cytochromes are descrined in parentheses.
[e] Bold letters indicate well-clusterred bin-genomes in terms of the parameters.
[f] Values were calculated from frequency of KO assignment to universal single-copied gene family lists (Supplementary Table S7 and S8).
[g] Raw reads for each condition were separately mapped to SP-L contigs by using similarity cut-off by 0.95 and length cut-off by 0.7.
[h] The genome completeness of L7ppro was improved to 99% if different ORF calling program of MetaGene annotater was applied.

Supplementary Table S6. Single-copied housekeeping gene list for validation of SP-H bin-genomes.

Number of ORF annotated to specific KO within bin-genomes[g]: 0 | 1 | 2 | 3 | >4

KO	Gene symbol	Description	Average peptide length ± SD	House keeping (%)[a]	Single copied (%)[b]	Bin-genome[h]															Plasmid etc.				
						H1geoA	H1geoB	H1geoC	H1geoD	H2geo	H3lac	H4Aact	H5geo	H6fir	H7geo	H8spi	H9bac	H10eps	H11bac	H12unc	H3lacP	H4actB	H6firP	H6firP2	H7geoP
K04075	tilS[ce]	tRNA(Ile)-lysidine synthetase	413 ± 61	96.4	99.8	0	1	1	1	1	1	1	1	1	1	1	1	1	1	1	0	0	0	0	0
K01866	tyrS[c]	tyrosyl-tRNA synthetase	412 ± 18	99.5	94.2	0	1	1	1	1	1	1	1	1	1	1	1	1	1	1	0	0	0	0	0
K03685	rnc[c]	ribonuclease III	243 ± 42	96.0	99.0	0	1	1	1	1	1	1	1	1	1	1	1	1	1	1	0	0	0	0	0
K02528	ksgA[c]	dimethyladenosine transferase	279 ± 22	98.1	98.8	0	1	1	1	1	1	2	1	1	1	1	1	1	1	1	0	0	0	0	0
K03106	ffh[c]	signal recognition particle subunit SRP54	469 ± 34	96.7	99.9	0	1	1	1	1	1	1	1	1	1	1	1	1	1	1	0	0	0	0	0
K01874	fmt[c]	methionyl-tRNA formyltransferase	624 ± 75	98.0	94.9	1	1	1	1	1	1	1	1	1	2	1	1	1	1	2	0	0	0	0	0
K01872	alaS[c]	Alanyl-tRNA synthetase	865 ± 80	98.3	99.1	2	1	1	1	1	1	1	1	1	1	2	2	2	1	1	0	0	0	0	0
K01890	pheT[c]	phenylalanyl-tRNA synthetase, beta subunit	797 ± 44	97.7	99.9	2	0	2	2	1	1	1	1	1	1	1	1	1	1	1	0	0	0	0	0
K03043	rpoB[cd]	DNA-directed RNA polymerase, beta subunit	1274 ± 108	95.5	99.5	2	1	1	0	1	1	1	1	1	1	1	1	1	1	2	0	1	0	0	0
K01881	proS[c]	prolyl-tRNA synthetase	543 ± 55	98.4	95.1	1	1	1	0	1	1	2	1	1	1	1	1	1	1	1	0	0	0	0	0
K02988	rpsE[cd]	ribosomal protein S5 [rpc operon]	180 ± 23	98.7	99.9	1	0	0	0	1	1	1	1	1	1	1	1	1	1	1	0	0	0	0	0
K02992	rpsG[ce]	ribosomal protein S7	159 ± 12	99.7	99.7	1	0	0	0	1	1	1	1	1	1	1	1	1	1	2	0	0	0	0	0
K02967	rpsB[c]	ribosomal protein S2	260 ± 29	98.1	99.9	1	0	0	1	1	1	1	1	1	1	1	1	1	1	1	0	0	0	0	0
K02895	rplX[c]	ribosomal protein L24 [rpc operon]	106 ± 11	99.0	99.5	1	0	0	0	1	1	1	1	1	1	1	1	1	1	1	0	0	0	0	0
K02874	rplN[cd]	ribosomal protein L14 [rpc operon]	123 ± 5	98.3	99.6	1	0	0	0	1	1	1	1	1	1	1	1	1	1	1	0	0	0	0	0
K02931	rplE[cd]	ribosomal protein L5 [rpc operon]	182 ± 8	98.3	99.6	1	0	0	0	1	1	1	1	1	1	1	1	1	1	1	0	0	0	0	0
K02906	rplC[cd]	ribosomal protein L3 [S10 operon]	215 ± 16	98.1	99.7	1	0	0	0	1	1	1	1	1	1	1	1	1	1	1	0	0	0	0	0
K02982	rpsC[d]	ribosomal protein S3 [S10 operon]	237 ± 23	98.8	99.8	1	0	0	0	1	1	1	1	1	1	1	1	1	1	1	0	0	0	0	0
K02994	rpsH	ribosomal protein S8 [rpc operon]	132 ± 17	98.1	99.9	1	0	0	0	1	1	1	1	1	1	1	1	1	1	1	0	0	0	0	0
K02948	rpsK[d]	ribosomal protein S11 [alpha operon]	130 ± 7	98.0	99.8	1	0	0	0	1	1	1	1	1	1	1	1	1	1	1	0	0	0	0	0
K02952	rpsM[d]	ribosomal protein S13 [alpha operon]	124 ± 11	98.5	99.8	1	0	0	0	1	1	1	1	1	1	1	1	1	1	1	0	0	0	0	0
K02933	rplF[cd]	ribosomal protein L6 [rpc operon]	178 ± 7	98.7	99.8	1	0	0	0	1	1	1	1	1	1	1	1	1	1	1	0	0	0	0	0
K03687	grpE[c]	co-chaperone GrpE	203 ± 31	97.6	97.3	1	0	0	0	1	1	1	1	1	1	1	1	1	1	1	0	0	0	0	0
K02600	nusA[d]	transcription termination factor NusA	424 ± 100	97.6	100.0	1	0	0	1	1	1	1	1	1	1	1	1	1	1	1	0	0	0	0	0
K03979	cgtA	Obg family GTPase CgtA	398 ± 56	97.6	99.8	1	0	0	0	1	1	1	1	1	1	1	1	2	2	2	0	0	0	0	0
K01875	serS	seryl-tRNA synthetase	429 ± 11	99.6	97.7	1	0	0	0	1	1	1	1	1	1	1	1	1	1	1	0	0	0	0	0
K01889	pheS	phenylalanyl-tRNA synthetase, alpha subunit	355 ± 47	97.9	99.2	1	0	0	0	1	1	1	1	1	1	1	1	1	1	1	0	0	0	0	0
K01873	valS	valyl-tRNA synthetase	904 ± 79	99.7	98.2	2	0	0	1	1	1	1	1	1	1	1	1	1	1	1	0	0	0	0	0
K02863	rplA[d]	ribosomal protein L1	231 ± 9	97.8	99.9	1	0	0	0	1	1	1	1	1	1	1	1	1	1	1	0	0	0	0	0
K02886	rplB[d]	ribosomal protein L2 [S10 operon]	274 ± 11	98.8	99.7	1	0	0	0	1	1	2	1	1	1	1	1	1	1	1	0	0	0	0	0
K02864	rplJ	ribosomal protein L10	172 ± 10	99.1	99.9	1	0	0	0	1	1	1	1	1	1	1	1	1	1	1	0	0	0	0	0
K02867	rplK[d]	ribosomal protein L11	144 ± 24	98.2	99.5	1	0	0	0	1	1	1	1	1	1	1	1	1	1	1	0	0	0	0	0
K02871	rplM[d]	ribosomal protein L13	147 ± 20	98.2	99.9	1	0	0	0	1	1	1	1	1	1	1	1	1	1	1	0	0	0	0	0
K02876	rplO	ribosomal protein L15 [rpc operon]	148 ± 14	98.1	99.9	1	0	0	0	1	1	1	1	1	1	1	1	1	1	1	0	0	0	0	0
K02881	rplR	ribosomal protein L18 [rpc operon]	124 ± 19	99.3	99.9	1	0	0	0	1	1	1	1	1	1	1	1	1	1	1	0	0	0	0	0
K02890	rplV	ribosomal protein L22 [S10 operon]	123 ± 21	99.3	99.8	1	0	0	0	1	1	1	1	1	1	1	1	1	1	1	0	0	0	0	0
K02892	rplW	ribosomal protein L23 [S10 operon]	99 ± 11	97.3	99.7	1	0	0	0	1	1	1	1	1	1	1	1	1	1	1	0	0	0	0	0
K02904	rpmC	ribosomal protein L29 [S10 operon]	69 ± 12	96.7	99.8	1	0	0	0	1	1	1	1	1	1	1	1	1	1	1	0	0	0	0	0
K02986	rpsD	ribosomal protein S4	203 ± 10	98.4	96.4	1	0	0	0	1	1	1	1	1	1	1	1	1	1	1	0	0	0	0	0
K02996	rpsI[d]	ribosomal protein S9	138 ± 15	98.7	99.9	1	0	0	0	1	1	1	1	1	1	1	1	1	1	1	0	0	0	0	0
K02946	rpsJ[d]	ribosomal protein S10 [S10 operon]	103 ± 4	97.6	99.7	1	0	0	0	1	1	1	1	1	1	1	1	1	1	1	0	0	0	0	0
K02950	rpsL	ribosomal protein S12	129 ± 11	97.7	99.6	1	0	0	0	0	1	1	1	1	1	1	1	1	1	1	0	0	0	0	0
K02956	rpsO	ribosomal protein S15	93 ± 16	97.7	99.8	1	0	0	0	1	1	1	1	1	1	1	1	1	1	1	0	0	0	0	0
K02961	rpsQ	ribosomal protein S17 [S10 operon]	90 ± 14	97.7	99.8	1	0	0	0	1	1	1	1	1	0	1	1	1	1	1	0	0	0	0	0
K02965	rpsS[d]	ribosomal protein S19 [S10 operon]	95 ± 15	97.3	99.8	1	0	0	0	1	1	1	1	1	1	1	1	1	1	1	0	0	0	0	0
K02926	rplD[d]	ribosomal protein L4 [S10 operon]	209 ± 17	98.8	99.7	1	0	0	0	1	1	2	1	1	1	1	1	1	1	1	0	0	0	0	0
K02935	rplL[d]	ribosomal protein L7/L12	125 ± 6	98.0	99.9	1	0	0	0	1	1	1	1	1	1	1	1	1	1	1	0	0	0	0	0
K02939	rplI	ribosomal protein L9	156 ± 22	97.0	99.9	1	0	0	1	1	1	1	1	1	1	1	1	1	1	1	0	0	0	0	0
K02878	rplP[d]	ribosomal protein L16 [S10 operon]	139 ± 7	98.7	99.8	1	0	0	0	1	1	1	1	1	1	1	1	1	1	2	0	0	0	0	0
K02879	rplQ	ribosomal protein L17 [alpha operon]	138 ± 31	97.7	99.8	1	0	0	0	1	1	1	1	1	1	1	1	1	1	1	0	0	0	0	0
K02884	rplS[d]	ribosomal protein L19	122 ± 13	97.6	99.8	1	0	0	1	1	1	1	1	1	1	1	1	1	1	1	0	0	0	0	0
K02887	rplT[d]	ribosomal protein L20	120 ± 9	98.7	99.9	1	0	0	0	1	1	1	1	1	1	1	1	1	1	1	0	0	0	0	0
K02888	rplU	ribosomal protein L21	109 ± 20	96.8	99.9	1	0	0	0	1	1	1	1	1	1	1	1	1	1	1	0	0	0	0	0
K02899	rpmA[d]	ribosomal protein L27	89 ± 7	97.6	99.9	1	0	0	0	1	1	1	1	1	1	1	1	1	1	1	0	0	0	0	0
K02916	rpmI	ribosomal protein L35	66 ± 5	97.0	99.9	1	0	0	0	1	1	1	1	1	1	1	1	1	1	1	0	0	0	0	0
K02990	rpsF	ribosomal protein S6	119 ± 23	97.1	100.0	1	0	0	0	1	1	1	1	1	1	1	1	1	1	1	0	0	0	0	0
K02959	rpsP	ribosomal protein S16	104 ± 33	99.0	99.8	1	0	0	0	1	1	1	1	1	1	1	1	1	1	1	0	0	0	0	0
K02968	rpsT	ribosomal protein S20	88 ± 8	97.8	99.9	0	1	1	0	1	1	1	1	1	1	1	1	1	1	1	0	0	0	0	0
K03040	rpoA	DNA-directed RNA polymerase, alpha subunit [alpha operon]	330 ± 16	98.4	98.5	1	0	0	0	1	1	1	1	1	1	1	1	1	1	1	0	0	0	0	0
K03076	secY	preprotein translocase, SecY subunit [rpc operon]	441 ± 21	99.1	95.1	1	0	0	0	1	1	1	1	1	1	1	1	1	1	1	0	0	0	0	0

Supplementary TableS6. *Continued.*

Number of ORF annotated to specific KO within bin-genomes[g] | 0 | 1 | 2 | 3 | >4 |

KO	Genesymbol	Description	Average peptide length ± SD	House keeping (%)	Single copied (%)	Bin-genome[h] H1geoA	H1geoB	H1geoC	H1geoD	H2geo	H3lac	H4Aact	H5geo	H6clo	H7geo	H8spi	H9bac	H10eps	H11bac	H12unc	Plasmid etc. H3lacP	H4Bact	H6cloP	H6cloP2	H7geoP
K01868	thrS	threonyl-tRNA synthetase	634 ± 51	99.1	93.4	1	0	0	0	1	1	1	1	1	1	1	1	1	1	1	0	0	0	0	0
K03110	ftsY	signal recognition particle-docking protein FtsY	392 ± 86	97.1	99.9	1	0	0	1	1	1	1	1	1	2	1	1	1	1	1	0	0	0	0	0
K01870	ileS	isoleucyl-tRNA synthetase	983 ± 75	98.2	97.3	1	0	0	0	1	1	1	1	1	1	1	1	1	1	1	0	0	0	0	1
K02601	nusG	transcription termination/antitermination factor NusG	193 ± 39	97.6	97.5	1	0	0	0	1	1	1	1	1	1	2	1	1	2	1	0	0	0	0	0
K06942	ychF	GTP-binding protein YchF	367 ± 10	99.1	99.6	1	0	0	0	1	1	1	1	1	1	1	1	1	1	2	0	0	0	0	0
K01887	argS	Arginyl-tRNA synthetase	571 ± 38	97.4	95.4	1	0	0	0	1	1	1	1	1	1	1	1	1	1	1	0	0	0	0	0
K03702	uvrB	excinuclease ABC, B subunit	687 ± 65	96.7	99.4	2	0	0	2	1	1	1	1	1	1	1	1	1	1	1	0	0	0	0	0
K01876	aspS	aspartyl-tRNA synthetase	584 ± 28	98.5	98.8	1	0	0	0	1	2	2	1	1	1	2	1	1	1	1	0	0	0	0	0
K01892	hisS	histidyl-tRNA synthetase	434 ± 25	98.4	96.8	1	1	1	1	1	2	1	1	2	1	1	1	1	1	1	0	0	0	0	0
K01883	cysS	cysteinyl-tRNA synthetase	473 ± 39	97.3	97.3	0	0	0	1	1	1	2	1	1	1	1	1	1	1	1	0	0	0	0	0
K02343	dnaX	DNA polymerase III, subunits gamma and tau	614 ± 141	97.5	95.3	2	0	0	1	1	1	1	1	1	1	2	1	1	1	2	0	0	0	0	0
K01937	pyrG[d]	CTP synthase	544 ± 15	94.1	99.8	1	0	0	1	1	1	1	1	1	1	1	1	1	1	1	0	0	0	0	0
K00927	pgk[d]	phosphoglycerate kinase	399 ± 15	94.5	98.4	1	0	0	0	1	1	1	1	1	1	1	1	1	1	1	0	0	0	0	0
K02834	rfbA	ribosome-binding factor A	133 ± 47	97.0	99.9	1	0	0	0	1	1	1	1	1	1	1	1	1	1	1	0	0	0	0	0
K02838	frr[d]	ribosome recycling factor	185 ± 5	98.6	99.9	1	0	0	0	1	1	1	1	1	1	1	1	1	1	1	0	0	0	0	0
K02357	tsf[d]	translation elongation factor Ts	285 ± 36	98.1	99.9	1	0	0	0	1	1	1	1	1	1	1	1	1	1	1	0	0	0	0	0
K02835	prfA	peptide chain release factor 1	358 ± 12	97.9	99.8	2	0	0	1	2	1	1	1	1	3	1	1	1	1	1	0	0	0	0	0
K03977	engA	ribosome-associated GTPase EngA	467 ± 46	97.3	99.8	1	0	0	2	1	1	1	1	1	1	1	1	1	1	1	0	0	0	0	0
K02519	infB	translation initiation factor IF-2	865 ± 127	98.5	99.6	1	0	0	0	1	1	1	1	2	1	1	1	1	1	1	0	1	0	0	0
K03664	smpB[d]	SmpB protein	156 ± 10	98.0	99.5	1	0	0	0	1	1	1	1	1	1	1	1	1	1	1	0	0	0	0	0
K03596	lepA	GTP-binding protein LepA	604 ± 22	98.4	99.4	2	0	2	0	1	1	1	1	1	1	1	1	1	1	1	0	0	0	0	0
K03545	tig	trigger factor	445 ± 23	97.9	99.4	1	0	0	0	1	1	1	1	1	1	1	1	1	1	1	0	0	0	0	0
K03438	mraW	MraW methylase family	318 ± 22	98.5	99.3	2	0	0	0	1	1	1	1	1	1	1	1	1	1	2	0	0	0	0	0
K02520	infC[d]	translation initiation factor IF-3	176 ± 39	96.4	98.7	1	0	0	0	1	1	1	1	1	1	1	1	1	1	1	0	0	0	0	0
K03046	rpoC	DNA-directed RNA polymerase, beta' or beta'' subunit	1335 ± 148	96.5	97.4	1	0	0	0	1	1	1	1	1	1	1	1	1	1	1	0	0	0	0	0
K02316	dnaG[d]	DNA primase	606 ± 57	97.8	98.2	1	0	0	1	1	1	1	2	1	1	1	2	1	1	1	0	9	0	0	2
K03553	recA	recA protein	358 ± 61	96.6	96.8	2	0	0	1	1	1	1	1	1	1	1	1	1	1	1	0	0	0	0	0
K02963	rpsR	ribosomal protein S18	83 ± 11	98.2	96.8	1	0	0	0	1	1	1	1	1	1	1	1	1	1	1	0	0	0	0	0
K00942	gmk	guanylate kinase	207 ± 24	95.7	96.6	1	0	0	0	1	1	1	1	1	1	0	1	1	1	1	0	0	0	0	0
K02338	dnaN	DNA polymerase III, beta subunit	375 ± 16	98.3	94.7	1	0	0	0	1	1	1	1	1	1	1	1	1	1	1	0	0	0	0	1
K02902	rpmB	ribosomal protein L28	75 ± 13	96.1	94.4	1	0	0	0	1	1	1	1	0	0	1	1	1	1	1	0	0	0	0	0
K02313	dnaA	chromosomal replication initiator protein DnaA	469 ± 54	96.6	94.1	2	1	2	1	2	1	1	2	1	2	1	1	1	1	1	0	0	0	0	0
K07042	ybeY	probable rRNA maturation factor	164 ± 31	93.8	99.9	1	0	0	0	1	1	1	1	1	1	1	1	1	1	1	0	0	0	0	0
K00859	coaE	dephospho-CoA kinase	212 ± 42	94.9	99.7	1	0	0	1	1	1	1	2	1	1	1	2	1	2	1	0	0	0	0	0
K03595	era	GTP-binding protein Era	306 ± 19	92.2	99.6	1	0	0	0	1	1	1	1	1	1	1	1	1	1	2	0	0	0	0	0
K03075	secG[f]	preprotein translocase, SecG subunit	105 ± 30	95.2	99.8	1	0	0	0	1	1	1	1	0	1	1	1	1	1	0	0	0	0	0	0
K03073	secE[f]	preprotein translocase, SecE subunit	92 ± 32	94.7	99.6	1	0	0	0	1	1	1	1	1	0	1	1	1	1	0	0	0	0	0	0
K04043	dnaK[f]	chaperone protein DnaK	629 ± 35	97.9	91.6	1	0	0	0	1	1	3	1	1	1	3	1	1	1	4	0	1	0	0	0
K02470	gyrB[fe]	DNA gyrase, B subunit	724 ± 94	97.6	88.5	1	0	0	0	1	1	2	1	1	1	2	1	1	1	1	0	0	0	0	0
K01972	ligA[f]	DNA ligase, NAD-dependent	675 ± 80	96.9	88.3	1	0	0	2	1	1	1	1	1	1	1	1	1	2	1	0	0	0	0	1
K02469	gyrA[f]	DNA gyrase, A subunit	852 ± 82	97.6	86.6	1	0	0	0	1	1	2	1	1	1	2	1	1	1	1	0	0	0	0	0
K03070	secA[f]	preprotein translocase, SecA subunit	889 ± 79	99.2	86.1	1	1	1	3	1	1	1	2	1	1	1	1	2	1	1	0	0	0	0	0
K02911	rpmF[f]	ribosomal protein L32	59 ± 7	93.5	96.6	0	0	0	0	0	1	1	0	0	0	0	0	1	0	0	0	0	0	0	0
K00566	mnmA[f]	tRNA (5-methylaminomethyl-2-thiouridylate)-methyltransferase	370 ± 25	95.0	93.7	2	1	2	3	2	1	1	3	1	2	1	2	2	1	1	0	0	0	0	0
K02914	rpmH[f]	ribosomal protein L34	46 ± 4	88.2	99.9	0	0	0	0	0	0	0	0	0	0	0	1	1	0	1	0	0	0	0	0
K01879	glyS[f]	glycyl-tRNA synthetase beta chain	693 ± 32	64.6	100.0	1	0	0	0	1	1	0	1	1	1	0	0	1	0	1	0	0	0	0	0
K01869	leuS[f]	leucyl-tRNA synthetase	859 ± 61	47.4	26.6	0	4	0	4	1	1	1	1	1	1	1	1	1	1	1	0	0	0	0	0

[a] Bacterial genomes having the housekeeping gene / Total 1943 KEGG organisms (in Nov, 2012) × 100 (%). Red colored, <95%; Orange colored, <97%.

[b] Bacterial genomes having the housekeeping gene with single copy / Total bacterial genomes having the housekeeping gene × 100 (%). Red colored, <95%; Orange colored, <97%.

[c] Seventeen single-copied housekeeping genes (seven for ribosomal proteins) used for core gene-based microbial community analysis.

[d] Thirty protein encoding phylogenetic marker genes used for AMPHORA program (Wu et al. 2008). The column yellow-highlighted.

[e] Three single-copied housekeeping genes (rpsG, tilS, and gyrB) used for phylogenetic tree analysis.

[f] Twelve single-copied housekeeping genes were not used for draft genome completeness estimation because of their low qualities.

[g] Column was colored depend on number of ORF annotated to specific KO within each bin-genome. White column was counted for draft genome completeness.

[h] Column with red letter showed multi-copied partial ORFs annotated to same KO, and the sum of the peptide length was similar to averaged peptide length, which treated as single copy existence.

Supplementary Table S7. Single-copied housekeeping gene list for validation of SP-M bin-genomes.

Number of ORF annotated to specific KO within bin-genomes[g] 0 | 1 | 2 | 3 | >4

KO	Gene symbol	Description	Average peptide length ± SD	House keeping (%)[a]	Single copied (%)[b]	Bin-genome[h]																								
						M1geoA	M1geoB	M1geoC	M1geoD	M2lac	M3ppro	M4geo	M5fir	M6act	M7geo	M8spi	M9unc	M10bac	M11eps	M12geo	M13tol	M14spi	M15fir	M17geo	M18tol	M19bac	M20fir	M21unc	M5firP	M16actB
K04075	tilS[ce]	tRNA(Ile)-lysidine synthetase	413 ± 61	96.4	99.8	1	0	0	1	1	1	1	1	1	1	1	1	1	1	1	1	1	2	1	1	1	0	2	0	0
K01866	tyrS[c]	tyrosyl-tRNA synthetase	412 ± 18	99.5	94.2	1	0	0	0	1	1	1	1	1	1	1	1	1	1	1	1	1	1	1	2	1	0	1	0	0
K03685	rnc[c]	ribonuclease III	243 ± 42	96.0	99.0	1	0	0	0	1	1	1	1	1	1	1	1	1	1	1	1	1	1	2	1	1	0	1	0	0
K02528	ksgA[c]	dimethyladenosine transferase	279 ± 22	98.1	98.8	1	0	0	1	1	2	1	1	1	1	1	1	1	1	1	1	1	1	1	3	1	1	0	0	0
K01870	ileS[c]	isoleucyl-tRNA synthetase	983 ± 75	98.2	97.3	1	0	1	1	1	1	1	1	1	1	1	1	1	1	1	1	1	1	2	3	2	0	2	0	0
K01874	fmt[c]	methionyl-tRNA formyltransferase	624 ± 75	98.0	94.9	1	0	0	1	1	1	1	1	1	1	1	1	1	1	1	1	1	1	1	1	1	0	1	0	0
K01872	alaS[c]	Alanyl-tRNA synthetase	865 ± 80	98.3	99.1	1	0	0	0	1	1	1	1	1	1	2	2	1	2	1	1	2	2	5	2	2	1	1	0	0
K01890	pheT[c]	phenylalanyl-tRNA synthetase, beta subunit	797 ± 44	97.7	99.9	1	0	0	0	1	1	1	1	2	1	1	1	1	1	1	1	2	1	1	6	1	1	1	0	0
K03046	rpoC[c]	DNA-directed RNA polymerase, beta' or beta'' subunit	1335 ± 148	96.5	97.4	1	2	2	2	1	1	1	1	1	1	1	1	1	1	1	1	1	1	3	1	3	2	1	0	0
K01881	proS[c]	prolyl-tRNA synthetase	543 ± 55	98.4	95.1	1	0	0	1	1	1	1	1	2	1	1	1	1	1	1	1	1	1	1	0	1	0	2	0	0
K02988	rpsE[cd]	ribosomal protein S5 [rpc operon]	180 ± 23	98.7	99.9	1	0	0	0	1	1	1	1	1	1	1	1	1	1	1	1	1	1	0	0	1	1	1	0	0
K02992	rpsG[ce]	ribosomal protein S7	159 ± 12	99.7	99.7	0	1	1	2	1	1	1	1	1	1	1	0	1	1	1	1	1	1	1	0	1	0	0	0	0
K02967	rpsB[c]	ribosomal protein S2	260 ± 29	98.1	99.9	1	0	0	0	1	1	1	1	1	2	1	1	1	1	1	1	1	1	0	0	1	0	1	0	0
K02895	rplX[c]	ribosomal protein L24 [rpc operon]	106 ± 11	99.0	99.5	1	0	0	0	1	1	1	1	1	1	1	1	1	1	1	1	1	1	0	0	1	1	1	0	0
K02874	rplN[cd]	ribosomal protein L14 [rpc operon]	123 ± 5	98.3	99.6	1	0	0	0	1	1	1	1	1	1	1	1	1	1	1	1	1	1	0	0	1	1	1	0	0
K02931	rplE[cd]	ribosomal protein L5 [rpc operon]	182 ± 8	98.3	99.6	1	0	0	0	1	1	1	1	1	1	1	1	1	1	1	1	1	1	0	0	2	1	1	0	0
K02906	rplC[cd]	ribosomal protein L3 [S10 operon]	215 ± 16	98.1	99.7	1	0	0	0	1	1	1	1	1	1	1	1	1	1	1	1	1	1	1	0	2	0	1	0	0
K02982	rpsC[d]	ribosomal protein S3 [S10 operon]	237 ± 23	98.8	99.8	1	0	0	0	1	1	1	1	1	1	1	1	1	1	1	1	1	1	0	0	1	0	1	0	0
K02994	rpsH	ribosomal protein S8 [rpc operon]	132 ± 17	98.1	99.9	1	0	0	0	1	1	1	1	1	1	1	1	1	1	1	1	1	1	0	0	1	1	0	0	0
K02948	rpsK[d]	ribosomal protein S11 [alpha operon]	130 ± 7	98.0	99.8	1	0	0	0	1	1	1	1	1	1	1	1	1	1	1	1	1	1	0	0	1	1	1	0	0
K02952	rpsM[d]	ribosomal protein S13 [alpha operon]	124 ± 11	98.5	99.8	1	0	0	0	1	1	1	1	1	1	1	1	1	1	1	1	1	1	0	0	1	1	1	0	0
K02933	rplF[cd]	ribosomal protein L6 [rpc operon]	178 ± 7	98.7	99.8	1	0	0	0	1	1	1	1	1	1	1	1	1	1	1	1	1	1	0	0	1	1	1	0	0
K03687	grpE[c]	co-chaperone GrpE	203 ± 31	97.6	97.3	1	0	0	0	1	1	1	1	1	1	1	1	1	1	1	1	1	1	1	1	1	0	0	0	0
K02600	nusA[d]	transcription termination factor NusA	424 ± 100	97.6	100.0	1	0	0	0	1	1	1	1	1	1	1	2	1	1	1	1	1	0	0	1	1	1	0	0	0
K03979	cgtA	Obg family GTPase CgtA	398 ± 56	97.6	99.8	1	0	0	0	1	1	1	2	1	1	1	1	2	2	1	1	1	2	1	4	2	1	1	0	0
K01875	serS	seryl-tRNA synthetase	429 ± 11	99.6	97.7	1	0	0	0	1	1	1	1	1	1	1	3	1	1	1	1	1	1	1	0	1	1	0	0	0
K01889	pheS	phenylalanyl-tRNA synthetase, alpha subunit	355 ± 47	97.9	99.2	1	0	0	0	1	1	1	1	1	1	1	1	1	1	1	1	1	1	1	0	1	1	1	0	0
K01873	valS	valyl-tRNA synthetase	904 ± 79	99.7	98.2	1	0	0	1	1	1	1	1	1	1	1	0	1	1	3	1	1	1	2	0	2	0	3	0	0
K02863	rplA[d]	ribosomal protein L1	231 ± 9	97.8	99.9	1	0	0	0	1	1	1	1	1	1	1	1	1	1	1	1	1	2	2	0	2	0	1	0	0
K02886	rplB[d]	ribosomal protein L2 [S10 operon]	274 ± 11	98.8	99.7	1	0	0	0	1	1	1	1	1	1	1	2	1	1	1	1	1	1	0	0	1	0	0	0	0
K02864	rplJ	ribosomal protein L10	172 ± 10	99.1	99.9	1	0	0	0	1	1	1	1	1	1	1	1	1	1	1	1	1	1	0	0	1	0	1	0	0
K02867	rplK[d]	ribosomal protein L11	144 ± 24	98.2	99.5	1	0	0	0	1	1	1	1	1	1	1	1	1	1	1	1	1	1	1	1	1	0	1	0	0
K02871	rplM[d]	ribosomal protein L13	147 ± 20	98.2	99.9	1	0	0	0	1	1	1	1	1	1	1	1	1	1	1	1	1	1	1	0	1	0	1	0	0
K02876	rplO	ribosomal protein L15 [rpc operon]	148 ± 14	98.1	99.9	1	0	0	0	1	1	1	1	1	1	1	1	1	1	1	1	1	1	0	0	1	0	1	0	0
K02881	rplR	ribosomal protein L18 [rpc operon]	124 ± 19	99.3	99.9	1	0	0	0	1	1	1	1	1	1	1	1	1	1	1	1	1	1	0	0	1	1	1	0	0
K02890	rplV	ribosomal protein L22 [S10 operon]	123 ± 21	99.3	99.8	1	0	0	0	1	1	1	1	1	1	1	1	1	1	1	1	1	1	0	0	1	0	0	0	0
K02892	rplW	ribosomal protein L23 [S10 operon]	99 ± 11	97.3	99.7	1	0	0	0	1	1	1	1	1	1	1	1	1	1	1	1	1	1	0	0	1	0	0	0	0
K02904	rpmC	ribosomal protein L29 [S10 operon]	69 ± 12	96.7	99.8	1	0	0	0	1	1	1	1	1	1	1	0	1	1	1	1	1	1	0	0	1	0	0	0	0
K02986	rpsD	ribosomal protein S4	203 ± 10	98.4	96.4	1	0	0	0	1	1	1	1	1	1	1	1	1	1	1	1	1	1	1	0	2	0	0	0	0
K02996	rpsI[d]	ribosomal protein S9	138 ± 15	98.7	99.9	1	0	0	0	1	1	1	1	1	1	1	0	1	1	1	1	1	1	1	1	1	0	1	0	0
K02946	rpsJ[d]	ribosomal protein S10 [S10 operon]	103 ± 4	97.6	99.7	1	0	0	0	1	1	1	1	1	1	1	1	1	1	1	1	1	1	1	1	1	0	1	0	0
K02950	rpsL	ribosomal protein S12	129 ± 11	97.7	99.6	0	1	1	1	1	1	0	1	1	1	1	0	1	1	1	1	1	1	1	0	1	0	0	0	0
K02956	rpsO	ribosomal protein S15	93 ± 16	97.7	99.8	1	0	0	0	1	1	1	1	1	1	1	1	1	1	1	1	1	1	1	1	1	0	0	0	0
K02961	rpsQ	ribosomal protein S17 [S10 operon]	90 ± 14	97.7	99.8	1	0	0	0	1	1	1	1	1	1	1	1	1	1	0	1	1	1	0	0	1	0	0	0	0
K02965	rpsS[d]	ribosomal protein S19 [S10 operon]	95 ± 15	97.3	99.8	1	0	0	0	1	1	1	1	1	1	1	1	1	1	1	1	1	1	0	0	1	0	0	0	0
K02926	rplD[d]	ribosomal protein L4 [S10 operon]	209 ± 17	98.8	99.7	1	0	0	0	1	1	1	1	2	1	1	1	1	1	1	1	1	1	0	0	2	0	0	0	0
K02935	rplL[d]	ribosomal protein L7/L12	125 ± 6	98.0	99.9	1	0	0	0	1	1	1	1	1	1	1	1	1	1	1	1	2	1	0	0	1	0	1	0	0
K02939	rplI	ribosomal protein L9	156 ± 22	97.0	99.9	1	0	0	0	1	1	1	1	1	1	1	0	1	1	1	1	1	1	3	1	1	0	1	0	0
K02878	rplP[d]	ribosomal protein L16 [S10 operon]	139 ± 7	98.7	99.8	1	0	0	0	1	1	1	1	1	1	1	1	1	1	1	1	1	1	0	0	1	0	2	0	0
K02879	rplQ	ribosomal protein L17 [alpha operon]	138 ± 31	97.7	99.8	1	0	0	0	1	1	1	1	1	1	1	1	1	1	1	1	1	1	1	0	1	0	2	0	0
K02884	rplS[d]	ribosomal protein L19	122 ± 13	97.6	99.8	1	0	0	0	1	1	1	1	1	1	1	1	1	1	1	1	1	1	0	1	1	0	0	0	0
K02887	rplT[d]	ribosomal protein L20	120 ± 9	98.7	99.9	1	0	0	0	1	1	1	1	1	1	1	1	1	1	1	1	1	1	1	0	0	0	1	0	0
K02888	rplU	ribosomal protein L21	109 ± 20	96.8	99.9	1	0	0	1	1	1	1	1	1	1	1	1	1	1	1	1	1	1	1	1	1	1	1	0	0
K02899	rpmA[d]	ribosomal protein L27	89 ± 7	97.6	99.9	1	0	0	1	1	1	1	1	1	1	1	1	1	1	1	1	1	1	1	1	1	1	1	0	0
K02916	rpmI	ribosomal protein L35	66 ± 5	97.0	99.9	1	0	0	0	1	1	1	1	1	1	1	1	1	1	1	0	1	1	0	0	1	0	1	0	0
K02990	rpsF	ribosomal protein S6	119 ± 23	97.1	100.0	1	0	0	0	1	1	1	1	1	1	1	1	1	1	1	1	1	1	1	0	1	0	0	0	0
K02959	rpsP	ribosomal protein S16	104 ± 33	99.0	99.8	1	0	0	0	1	1	1	1	1	1	1	1	1	1	1	0	1	1	2	0	2	0	0	0	0
K02968	rpsT	ribosomal protein S20	88 ± 8	97.8	99.9	0	0	0	0	1	1	1	1	1	1	1	1	1	1	1	1	1	1	0	0	1	0	1	0	0
K03040	rpoA	DNA-directed RNA polymerase, alpha subunit [alpha operon]	330 ± 16	98.4	98.5	1	0	0	1	1	1	1	1	1	1	1	1	1	1	1	1	1	1	3	0	1	0	1	0	0
K03076	secY	preprotein translocase, SecY subunit [rpc operon]	441 ± 21	99.1	95.1	1	0	1	2	1	1	1	1	1	1	1	1	1	1	1	1	1	1	0	1	1	0	1	0	0
K01868	thrS	threonyl-tRNA synthetase	634 ± 51	99.1	93.4	1	0	0	0	1	1	1	1	1	1	1	1	1	1	2	1	1	1	0	0	1	0	3	0	0
K03110	ftsY	signal recognition particle-docking protein FtsY	392 ± 86	97.1	99.9	2	0	0	0	1	1	1	1	1	1	1	1	1	1	2	1	1	1	1	1	1	0	1	0	0
K03106	ffh	signal recognition particle subunit SRP54	469 ± 34	96.7	99.9	1	0	0	0	1	1	1	1	1	1	1	1	1	1	1	1	1	1	1	1	1	0	0	0	0
K02601	nusG	transcription termination/antitermination factor NusG	193 ± 39	97.6	97.5	1	0	0	0	1	1	1	1	1	1	2	1	1	1	1	1	2	1	1	1	2	0	2	0	0

Supplementary TableS7. *Continued.*

Number of ORF annotated to specific KO within bin-genomes[g]: 0 | 1 | 2 | 3 | >4

KO	Gene symbol	Description	Average peptide length ± SD	House keeping (%)[a]	Single copied (%)[b]	M1geoA	M1geoB	M1geoC	M1geoD	M2lac	M3ppro	M4geo	M5fir	M6act	M7geo	M8spi	M9unc	M10bac	M11eps	M12geo	M13tol	M14spi	M15fir	M17geo	M18tol	M19bac	M20fir	M21unc	M5firP	M16actB
						Bin-genome[h]																								
K06942	ychF	GTP-binding protein YchF	367 ± 10	99.1	99.6	0	1	1	1	1	1	1	1	1	1	1	1	1	1	1	1	1	1	1	1	3	1	3	0	0
K01887	argS	Arginyl-tRNA synthetase	571 ± 38	97.4	95.4	1	0	0	0	1	1	1	1	1	1	1	1	1	1	1	1	1	1	5	2	1	0	1	0	0
K03702	uvrB	excinuclease ABC, B subunit	687 ± 65	96.7	99.4	2	0	0	1	1	1	1	1	1	1	1	1	1	1	1	1	1	1	1	2	0	0	1	0	0
K01876	aspS	aspartyl-tRNA synthetase	584 ± 28	98.5	98.8	0	2	0	1	2	1	1	1	2	1	2	1	1	1	1	1	1	1	4	0	1	1	0	0	1
K01892	hisS	histidyl-tRNA synthetase	434 ± 25	98.4	96.8	1	0	0	0	2	1	1	2	1	1	1	1	1	1	1	1	1	3	1	2	1	0	2	0	0
K01883	cysS	cysteinyl-tRNA synthetase	473 ± 39	97.3	97.3	1	0	0	0	1	1	1	1	2	1	1	1	1	1	1	1	1	2	2	1	1	0	1	0	0
K02343	dnaX	DNA polymerase III, subunits gamma and tau	614 ± 141	97.5	95.3	1	0	0	0	1	1	1	1	1	1	2	1	1	1	1	1	2	1	4	1	1	0	1	0	0
K01937	pyrG[d]	CTP synthase	544 ± 15	94.1	99.8	1	0	0	0	1	1	1	1	1	1	1	2	1	1	1	1	1	1	1	3	1	0	1	0	0
K00927	pgk[d]	phosphoglycerate kinase	399 ± 15	94.5	98.4	2	0	0	1	1	1	1	1	1	1	1	1	1	1	1	1	1	1	1	0	1	0	0	0	0
K02834	rfbA	ribosome-binding factor A	133 ± 47	97.0	99.9	1	0	0	0	1	1	1	1	1	1	1	1	1	1	1	1	1	1	0	2	1	0	1	0	0
K02838	frr[d]	ribosome recycling factor	185 ± 5	98.6	99.9	1	0	0	0	1	1	1	1	1	2	1	1	1	1	1	1	1	1	0	1	1	0	2	0	0
K02357	tsf[d]	translation elongation factor Ts	285 ± 36	98.1	99.9	1	0	0	0	1	1	1	1	1	2	1	1	1	1	1	1	2	1	0	0	1	0	0	0	0
K02835	prfA	peptide chain release factor 1	358 ± 12	97.9	99.8	2	0	0	0	1	2	2	1	1	3	1	1	1	1	3	1	1	1	1	2	1	0	1	0	0
K03977	engA	ribosome-associated GTPase EngA	467 ± 46	97.3	99.8	1	0	0	0	1	1	1	1	1	1	1	1	1	1	1	1	1	1	1	2	1	0	0	0	0
K02519	infB	translation initiation factor IF-2	865 ± 127	98.5	99.6	1	0	0	0	1	1	1	1	1	1	1	2	1	1	1	1	1	1	2	3	3	1	2	0	0
K03664	smpB[d]	SmpB protein	156 ± 10	98.0	99.5	1	0	0	0	1	1	1	1	1	1	1	1	1	1	1	1	1	1	1	1	1	0	1	0	0
K03596	lepA	GTP-binding protein LepA	604 ± 22	98.4	99.4	0	2	0	0	1	1	1	1	1	2	1	1	1	1	1	1	1	1	1	2	1	1	1	0	0
K03545	tig	trigger factor	445 ± 23	97.9	99.4	1	0	0	0	1	1	1	1	1	2	1	1	1	1	1	1	1	1	3	0	1	0	0	0	0
K03438	mraW	MraW methylase family	318 ± 22	98.5	99.3	1	0	0	0	1	1	1	1	1	1	1	2	1	1	1	1	2	1	2	0	1	1	4	0	0
K02520	infC[d]	translation initiation factor IF-3	176 ± 39	96.4	98.7	1	0	0	0	1	1	1	1	1	1	1	1	1	1	1	1	1	1	0	0	1	0	1	0	0
K03043	rpoB[d]	DNA-directed RNA polymerase, beta subunit	1274 ± 108	95.5	99.5	1	0	0	0	1	1	1	1	1	1	1	1	1	1	1	1	6	1	1	4	1	0	0	0	1
K02316	dnaG[d]	DNA primase	606 ± 57	97.8	98.2	1	1	1	0	1	3	1	1	1	3	1	1	1	1	1	1	1	1	3	3	1	0	1	0	4
K03553	recA	recA protein	358 ± 61	96.6	96.8	1	0	0	0	1	1	1	1	1	1	1	1	1	1	1	1	1	1	1	2	1	1	2	0	0
K02963	rpsR	ribosomal protein S18	83 ± 11	98.2	96.8	0	0	0	0	1	0	1	1	1	1	1	1	1	1	1	1	1	1	1	0	1	0	0	0	0
K00942	gmk	guanylate kinase	207 ± 24	95.7	96.6	1	0	0	0	1	1	1	1	1	1	0	1	1	1	1	1	0	1	2	1	1	0	1	0	0
K02338	dnaN	DNA polymerase III, beta subunit	375 ± 16	98.3	94.7	1	0	0	0	1	1	1	1	1	1	2	1	1	1	1	1	1	1	1	1	1	1	2	0	0
K02902	rpmB	ribosomal protein L28	75 ± 13	96.1	94.4	0	1	0	0	1	1	1	0	1	1	1	0	1	1	0	1	0	1	0	0	1	0	0	0	0
K02313	dnaA	chromosomal replication initiator protein DnaA	469 ± 54	96.6	94.1	1	1	1	0	1	2	2	1	1	2	1	1	1	1	2	1	1	1	2	2	1	1	1	0	1
K07042	ybeY	probable rRNA maturation factor	164 ± 31	93.8	99.9	1	0	0	0	1	2	1	1	1	1	1	1	1	1	1	1	1	1	1	1	1	1	1	0	0
K00859	coaE	dephospho-CoA kinase	212 ± 42	94.9	99.7	1	0	1	1	1	1	1	1	1	1	1	0	1	1	1	1	1	1	1	3	2	0	0	0	0
K03595	era	GTP-binding protein Era	306 ± 19	92.2	99.6	1	0	0	0	1	1	1	1	1	1	1	1	1	1	1	1	1	1	1	1	2	2	1	0	0
K03075	secG[f]	preprotein translocase, SecG subunit	105 ± 30	95.2	99.8	0	0	0	0	1	1	1	0	1	1	1	0	1	1	1	1	1	0	1	1	1	0	0	0	0
K03073	secE[f]	preprotein translocase, SecE subunit	92 ± 32	94.7	99.6	0	0	0	0	1	1	1	0	1	1	1	1	1	1	0	1	0	1	0	0	1	0	0	0	0
K04043	dnaK[f]	chaperone protein DnaK	629 ± 35	97.9	91.6	1	0	0	0	1	1	1	1	3	1	3	2	1	1	1	1	1	1	2	2	1	1	3	0	1
K02470	gyrB[fe]	DNA gyrase, B subunit	724 ± 94	97.6	88.5	1	0	0	0	1	1	1	1	1	1	2	2	1	1	2	1	2	2	1	1	1	2	2	0	0
K01972	ligA[f]	DNA ligase, NAD-dependent	675 ± 80	96.9	88.3	0	1	2	2	1	1	1	1	1	1	1	1	1	2	1	1	1	1	1	4	3	0	3	0	0
K02469	gyrA[f]	DNA gyrase, A subunit	852 ± 82	97.6	86.6	1	0	0	0	1	1	1	1	2	4	2	1	1	1	2	1	2	2	2	2	2	1	1	0	0
K03070	secA[f]	preprotein translocase, SecA subunit	889 ± 79	99.2	86.1	1	1	1	1	1	1	1	1	1	1	1	1	1	1	1	1	1	1	2	2	1	1	1	0	0
K02911	rpmF[f]	ribosomal protein L32	59 ± 7	93.5	96.6	0	0	0	0	1	0	0	0	0	0	0	1	0	1	0	1	0	1	0	0	0	0	0	0	0
K00566	mnmA[f]	tRNA (5-methylaminomethyl-2-thiouridylate)-methyltransferase	370 ± 25	95.0	93.7	1	1	1	1	1	3	2	1	1	3	1	1	1	2	2	1	1	1	5	1	1	0	1	0	0
K02914	rpmH[f]	ribosomal protein L34	46 ± 4	88.2	99.9	0	0	0	0	0	0	0	0	0	0	0	1	0	0	0	0	1	0	0	0	0	0	1	0	0
K01879	glyS[f]	glycyl-tRNA synthetase beta chain	693 ± 32	64.6	100.0	1	1	1	0	1	1	1	1	0	1	0	1	0	1	1	1	0	2	2	1	0	0	2	0	0
K01869	leuS[f]	leucyl-tRNA synthetase	859 ± 61	47.4	26.6	1	0	0	1	1	1	1	1	1	1	1	1	2	1	1	1	1	1	2	2	1	0	1	0	0

[a] Bacterial genomes having the housekeeping gene / Total 1943 KEGG organisms (in Nov, 2012) × 100 (%). Red colored, <95%; Orange colored, <97%.
[b] Bacterial genomes having the housekeeping gene with single copy / Total bacterial genomes having the housekeeping gene × 100 (%). Red colored, <95%; Orange colored, <97%.
[c] Seventeen single-copied housekeeping genes (seven for ribosomal proteins) used for core gene-based microbial community analysis.
[d] Thirty protein encoding phylogenetic marker genes used for AMPHORA program (Wu et al. 2008). The column yellow-highlighted.
[e] Three single-copied housekeeping genes (rpsG, tilS, and gyrB) used for phylogenetic tree analysis.
[f] Twelve single-copied housekeeping genes were not used for draft genome completeness estimation because of their low qualities.
[g] Column was colored depend on number of ORF annotated to specific KO within each bin-genome. White column was counted for draft genome completeness.
[h] Column with red letter showed multi-copied partial ORFs annotated to same KO, and the sum of the peptide length was similar to averaged peptide length, which treated as single copy existence.

Supplementary Table S8. Single-copied housekeeping gene list for validation of SP-L bin-genomes.

Number of ORF annotated to specific KO within bin-genomes[g]: 0 | 1 | 2 | 3 | >4

KO	Gene symbol	Description	Average peptide length ± SD	House keeping (%)[a]	Single copied (%)[b]	Bin-genome[h] L1geo	L2tol	L3tol	L4bac	L5met	L6fir	L7ppro	L8spi	L9bac	L10gmet	L11pro	L12lac	L13fir	L14bac	L15bac	L16bac	L17bac	L18ver	L19bac	L20tol	L20tolB	L21met	L22fir	L23elu	L24fir	L12lacP
K03702	uvrB[c]	excinuclease ABC, B subunit	687 ± 65	96.7	99.4	1	1	1	1	1	1	1	1	1	1	1	1	1	1	1	1	1	2	1	3	2	2	1	1	1	0
K01887	argS[c]	Arginyl-tRNA synthetase	571 ± 38	97.4	95.4	1	1	1	1	1	1	1	1	1	1	1	1	1	1	1	1	1	1	1	0	0	2	1	1	2	0
K03685	rnc[c]	ribonuclease III	243 ± 42	96.0	99.0	1	1	1	1	1	1	1	1	1	1	1	1	1	1	1	1	1	1	0	1	0	1	1	1	0	0
K02528	ksgA[c]	dimethyladenosine transferase	279 ± 22	98.1	98.8	1	1	1	2	1	1	2	1	1	1	1	1	1	2	2	1	1	1	1	2	0	1	1	1	0	0
K00927	pgk[cd]	phosphoglycerate kinase	399 ± 15	94.5	98.4	1	1	1	1	1	1	1	1	1	1	1	1	1	1	1	1	1	1	1	0	0	1	1	1	1	0
K01874	fmt[c]	methionyl-tRNA formyltransferase	624 ± 75	98.0	94.9	1	1	1	1	2	1	1	1	1	1	1	1	1	1	1	1	1	1	2	3	0	1	1	1	0	0
K01872	alaS[c]	Alanyl-tRNA synthetase	865 ± 80	98.3	99.1	1	1	1	2	2	1	1	2	2	1	1	1	1	2	2	1	3	1	3	2	2	2	2	1	3	0
K01890	pheT[c]	phenylalanyl-tRNA synthetase, beta subunit	797 ± 44	97.7	99.9	1	1	1	1	2	1	1	1	1	1	1	1	1	1	1	1	1	1	1	4	2	2	1	1	1	0
K01873	valS	valyl-tRNA synthetase	904 ± 79	99.7	98.2	1	1	1	1	1	1	3	1	1	1	1	1	1	2	2	1	3	2	1	0	0	2	1	2	1	0
K01881	proS[c]	prolyl-tRNA synthetase	543 ± 55	98.4	95.1	1	1	1	1	1	1	1	1	1	1	1	1	2	1	1	1	1	1	2	0	0	0	3	2	1	0
K02988	rpsE[cd]	ribosomal protein S5 [rpc operon]	180 ± 23	98.7	99.9	1	1	1	1	1	1	1	1	1	1	1	1	1	1	1	1	1	1	0	0	0	0	1	1	1	0
K02992	rpsG[ce]	ribosomal protein S7	159 ± 12	99.7	99.7	1	1	1	1	1	1	1	1	1	1	1	1	1	1	1	1	0	1	1	0	0	0	1	1	1	0
K02967	rpsB[c]	ribosomal protein S2	260 ± 29	98.1	99.9	1	1	2	1	1	1	1	1	1	1	1	1	1	1	1	1	1	1	1	0	0	0	1	1	1	0
K02895	rplX[c]	ribosomal protein L24 [rpc operon]	106 ± 11	99.0	99.5	1	1	0	1	1	1	1	1	1	1	1	1	1	1	1	1	2	1	0	0	0	0	1	1	1	0
K02874	rplN[cd]	ribosomal protein L14 [rpc operon]	123 ± 5	98.3	99.6	1	1	0	1	1	1	1	1	1	1	1	1	1	1	1	1	2	1	0	0	0	0	1	1	1	0
K02931	rplE[cd]	ribosomal protein L5 [rpc operon]	182 ± 8	98.3	99.6	1	1	0	1	1	1	1	1	1	1	1	1	1	1	1	1	2	1	0	0	0	0	1	1	1	0
K02906	rplC[cd]	ribosomal protein L3 [S10 operon]	215 ± 16	98.1	99.7	1	1	0	1	1	1	1	1	1	1	1	1	1	1	1	1	1	1	1	0	0	0	1	1	1	0
K02982	rpsC[d]	ribosomal protein S3 [S10 operon]	237 ± 23	98.8	99.8	1	1	0	1	1	1	1	1	1	1	1	1	1	1	1	1	0	1	1	0	0	0	1	1	1	0
K02994	rpsH	ribosomal protein S8 [rpc operon]	132 ± 17	98.1	99.9	1	1	0	1	1	1	1	1	1	1	1	1	1	1	1	1	1	1	0	0	0	0	2	1	1	0
K02948	rpsK[d]	ribosomal protein S11 [alpha operon]	130 ± 7	98.0	99.8	1	1	0	1	1	1	0	1	1	1	1	1	1	1	1	1	1	1	1	0	0	0	1	1	1	0
K02952	rpsM[d]	ribosomal protein S13 [alpha operon]	124 ± 11	98.5	99.8	1	1	1	1	1	1	0	1	1	1	1	1	1	1	1	1	1	1	1	0	0	0	1	1	1	0
K02933	rplF[cd]	ribosomal protein L6 [rpc operon]	178 ± 7	98.7	99.8	1	1	0	1	1	1	1	1	1	1	1	1	1	1	1	1	1	1	0	0	0	0	2	1	1	0
K03687	grpE[c]	co-chaperone GrpE	203 ± 31	97.6	97.3	1	1	1	1	1	1	1	1	1	1	1	1	1	1	1	1	1	1	1	1	0	0	1	1	2	0
K02600	nusA[d]	transcription termination factor NusA	424 ± 100	97.6	100.0	1	1	1	1	2	1	1	1	1	1	1	1	1	1	1	1	1	1	1	1	0	2	1	1	0	0
K03979	cgtA	Obg family GTPase CgtA	398 ± 56	97.6	99.8	1	1	1	3	1	1	1	0	2	1	1	1	1	2	2	2	2	2	3	1	0	1	1	1	1	0
K01875	serS	seryl-tRNA synthetase	429 ± 11	99.6	97.7	1	1	1	1	2	1	1	1	1	1	1	1	1	1	1	1	1	1	1	0	0	0	1	1	0	0
K01889	pheS	phenylalanyl-tRNA synthetase, alpha subunit	355 ± 47	97.9	99.2	1	1	1	1	2	1	1	1	1	1	1	1	1	1	1	1	1	1	1	0	0	1	1	1	1	0
K03046	rpoC	DNA-directed RNA polymerase, beta' or beta'' subunit	1335 ± 148	96.5	97.4	1	1	1	1	0	1	1	1	1	3	1	1	1	1	1	1	4	3	2	1	0	0	1	1	1	0
K02863	rplA[d]	ribosomal protein L1	231 ± 9	97.8	99.9	1	1	0	1	1	1	1	1	1	1	1	1	1	2	1	1	1	1	1	0	0	0	1	1	1	0
K02886	rplB[d]	ribosomal protein L2 [S10 operon]	274 ± 11	98.8	99.7	1	1	0	1	1	1	0	1	1	1	1	1	1	1	1	1	0	1	1	0	0	0	1	1	1	0
K02864	rplJ	ribosomal protein L10	172 ± 10	99.1	99.9	1	1	0	1	1	1	1	1	1	1	1	1	1	1	1	1	1	1	1	0	0	0	1	1	1	0
K02867	rplK[d]	ribosomal protein L11	144 ± 24	98.2	99.5	1	1	0	1	1	1	1	1	1	1	1	1	1	1	1	1	1	1	1	0	0	0	1	1	1	0
K02871	rplM[d]	ribosomal protein L13	147 ± 20	98.2	99.9	1	1	1	1	1	1	1	1	1	1	1	1	1	1	1	1	1	1	1	0	0	0	1	1	0	0
K02876	rplO	ribosomal protein L15 [rpc operon]	148 ± 14	98.1	99.9	1	1	1	1	1	1	1	1	1	1	1	1	1	1	1	1	1	1	0	0	0	0	1	1	1	0
K02881	rplR	ribosomal protein L18 [rpc operon]	124 ± 19	99.3	99.9	1	1	1	1	1	1	0	1	1	1	1	1	1	1	1	1	1	1	0	0	0	0	1	1	1	0
K02890	rplV	ribosomal protein L22 [S10 operon]	123 ± 21	99.3	99.8	1	1	0	1	1	1	0	1	1	1	1	2	1	1	1	1	0	1	1	0	0	0	1	1	1	0
K02892	rplW	ribosomal protein L23 [S10 operon]	99 ± 11	97.3	99.7	1	1	0	1	1	1	1	1	1	1	1	1	1	1	1	1	1	1	1	0	0	0	1	1	1	0
K02904	rpmC	ribosomal protein L29 [S10 operon]	69 ± 12	96.7	99.8	1	1	0	1	1	1	0	1	1	1	0	1	1	1	1	1	1	1	0	0	1	0	1	1	1	0
K02986	rpsD	ribosomal protein S4	203 ± 10	98.4	96.4	1	1	0	1	1	1	1	1	1	1	1	1	1	1	1	1	1	1	1	0	0	0	1	1	1	0
K02996	rpsI[d]	ribosomal protein S9	138 ± 15	98.7	99.9	1	1	1	1	1	1	1	1	1	1	1	1	1	1	1	1	1	1	1	0	0	0	1	1	0	0
K02946	rpsJ[d]	ribosomal protein S10 [S10 operon]	103 ± 4	97.6	99.7	1	1	1	1	1	1	1	1	1	1	1	1	1	1	1	1	1	1	1	0	0	0	1	1	1	0
K02950	rpsL	ribosomal protein S12	129 ± 11	97.7	99.6	1	1	1	1	1	1	0	1	1	1	1	1	1	1	1	1	1	1	1	0	0	0	1	1	1	0
K02956	rpsO	ribosomal protein S15	93 ± 16	97.7	99.8	1	1	1	1	1	1	1	1	1	1	1	1	1	1	1	1	1	1	1	0	0	0	1	1	1	0
K02961	rpsQ	ribosomal protein S17 [S10 operon]	90 ± 14	97.7	99.8	0	1	0	1	1	1	0	1	1	1	1	1	1	1	1	1	2	1	0	0	1	0	1	1	1	0
K02965	rpsS[d]	ribosomal protein S19 [S10 operon]	95 ± 15	97.3	99.8	1	1	0	1	1	1	0	1	1	1	1	1	1	1	1	1	0	1	1	0	0	0	1	1	1	0
K02926	rplD[d]	ribosomal protein L4 [S10 operon]	209 ± 17	98.8	99.7	1	1	0	1	0	1	1	1	1	1	1	1	1	1	1	1	1	1	1	0	0	0	1	1	1	0
K02935	rplL[d]	ribosomal protein L7/L12	125 ± 6	98.0	99.9	1	1	0	1	0	1	1	1	1	1	1	1	1	1	1	1	1	1	2	0	0	0	1	1	1	0
K02939	rplI	ribosomal protein L9	156 ± 22	97.0	99.9	1	1	1	1	0	1	1	1	1	1	1	1	1	1	1	1	1	1	1	0	0	0	1	1	1	0
K02878	rplP[d]	ribosomal protein L16 [S10 operon]	139 ± 7	98.7	99.8	1	1	0	1	0	1	1	1	1	1	1	1	1	1	1	1	1	1	0	0	1	0	1	1	1	0
K02879	rplQ	ribosomal protein L17 [alpha operon]	138 ± 31	97.7	99.8	1	1	1	1	0	1	1	1	1	1	1	1	1	1	1	1	1	1	1	0	0	0	1	1	1	0
K02884	rplS[d]	ribosomal protein L19	122 ± 13	97.6	99.8	1	1	0	1	0	1	1	1	1	1	1	1	1	1	1	1	1	1	1	0	0	0	1	1	1	0
K02887	rplT[d]	ribosomal protein L20	120 ± 9	98.7	99.9	1	1	1	1	0	1	1	1	1	1	1	1	1	1	1	1	1	1	1	0	0	0	1	1	1	0
K02888	rplU	ribosomal protein L21	109 ± 20	96.8	99.9	1	1	1	1	0	1	1	1	1	1	1	1	1	1	1	1	1	1	1	0	0	0	1	1	1	0
K02899	rpmA[d]	ribosomal protein L27	89 ± 7	97.6	99.9	1	1	1	1	0	1	1	1	1	1	1	1	1	1	1	1	1	1	1	0	0	0	1	1	1	0
K02916	rpmI	ribosomal protein L35	66 ± 5	97.0	99.9	1	1	1	0	0	1	1	1	1	0	1	1	1	1	1	1	1	1	1	0	0	0	1	1	1	0
K02990	rpsF	ribosomal protein S6	119 ± 23	97.1	100.0	1	1	1	1	0	1	1	1	1	1	1	1	1	1	1	1	1	1	1	0	0	0	1	1	1	0
K02959	rpsP	ribosomal protein S16	104 ± 33	99.0	99.8	1	1	0	1	0	1	1	1	1	1	1	1	1	1	1	1	1	1	1	0	0	0	1	1	1	0
K02968	rpsT	ribosomal protein S20	88 ± 8	97.8	99.9	1	1	1	0	0	1	1	1	1	1	1	1	1	1	1	1	1	1	1	0	0	0	1	1	1	0
K03040	rpoA	DNA-directed RNA polymerase, alpha subunit [alpha operon]	330 ± 16	98.4	98.5	1	1	1	1	0	1	1	1	1	1	1	1	1	1	1	1	1	1	2	0	0	0	1	1	2	0
K03076	secY	preprotein translocase, SecY subunit [rpc operon]	441 ± 21	99.1	95.1	1	1	1	1	2	1	1	1	1	1	1	1	1	1	1	1	1	1	0	0	0	0	1	1	2	0
K01868	thrS	threonyl-tRNA synthetase	634 ± 51	99.1	93.4	1	1	1	1	1	1	1	1	1	1	1	2	1	1	1	1	1	1	1	0	0	2	1	1	1	0
K03110	ftsY	signal recognition particle-docking protein FtsY	392 ± 86	97.1	99.9	2	1	1	1	1	1	2	1	1	1	1	1	1	1	1	1	1	1	1	1	2	1	1	0	1	0
K03106	ffh	signal recognition particle subunit SRP54	469 ± 34	96.7	99.9	1	1	1	1	1	1	1	1	1	1	1	1	1	1	1	1	1	1	1	1	0	0	1	0	1	0
K02601	nusG	transcription termination/antitermination factor NusG	193 ± 39	97.6	97.5	1	1	0	1	1	1	1	2	1	1	1	1	1	1	1	2	0	2	1	0	0	0	1	1	1	0

Supplementary TableS8. *Continued.*

Number of ORF annotated to specific KO within bin-genomes[g]: 0 | 1 | 2 | 3 | >4

KO	Gene symbol	Description	Average peptide length ± SD	House keeping (%)[a]	Single copied (%)[b]	Bin-genome[h] L1geo	L2tol	L3tol	L4bac	L5met	L6fir	L7ppro	L8spi	L9bac	L10gmet	L11pro	L12lac	L13fir	L14bac	L15bac	L16bac	L17bac	L18ver	L19bac	L20tol	L20toL	L21met	L22fir	L23elu	L24fir	L12lacP
K06942	ychF	GTP-binding protein YchF	367 ± 10	99.1	99.6	1	1	1	1	1	1	1	1	1	1	2	1	1	1	1	1	1	2	1	1	3	1	1	1	1	0
K04075	tilS[e]	tRNA(Ile)-lysidine synthetase	413 ± 61	96.4	99.8	1	1	1	1	0	1	1	1	1	1	1	1	1	1	1	1	1	1	1	2	1	0	1	1	1	0
K01866	tyrS	tyrosyl-tRNA synthetase	412 ± 18	99.5	94.2	1	2	2	1	1	1	1	1	1	1	1	1	1	1	1	1	1	1	1	0	2	0	1	1	1	0
K01876	aspS	aspartyl-tRNA synthetase	584 ± 28	98.5	98.8	1	1	1	1	1	1	1	2	1	1	1	2	1	1	1	1	2	1	1	0	0	1	1	1	1	0
K01892	hisS	histidyl-tRNA synthetase	434 ± 25	98.4	96.8	1	1	1	1	2	1	1	1	1	1	1	2	1	1	1	1	1	1	0	3	2	1	2	1	1	0
K01883	cysS	cysteinyl-tRNA synthetase	473 ± 39	97.3	97.3	1	1	1	1	0	1	1	1	1	1	1	1	1	1	1	1	1	1	1	3	0	0	1	1	1	0
K02343	dnaX	DNA polymerase III, subunits gamma and tau	614 ± 141	97.5	95.3	1	1	1	1	0	2	1	2	1	1	2	1	1	1	1	1	1	2	1	1	1	0	1	2	2	0
K01937	pyrG[d]	CTP synthase	544 ± 15	94.1	99.8	1	1	1	1	1	0	1	1	1	1	1	1	1	1	1	0	2	1	1	2	0	0	1	1	0	0
K01870	ileS	isoleucyl-tRNA synthetase	983 ± 75	98.2	97.3	1	1	1	1	4	1	1	1	1	1	1	1	2	1	1	1	1	1	1	1	1	1	1	1	1	0
K02834	rfbA	ribosome-binding factor A	133 ± 47	97.0	99.9	1	1	1	1	0	1	1	1	1	1	1	1	1	1	1	1	1	1	1	0	0	0	1	1	1	0
K02838	frr[d]	ribosome recycling factor	185 ± 5	98.6	99.9	1	1	1	1	0	1	1	1	1	1	1	1	1	1	1	1	1	1	1	0	0	0	1	0	1	0
K02357	tsf[d]	translation elongation factor Ts	285 ± 36	98.1	99.9	1	1	1	1	0	1	1	1	1	1	1	1	1	1	1	1	1	2	1	0	0	0	3	1	1	0
K02835	prfA	peptide chain release factor 1	358 ± 12	97.9	99.8	1	1	1	1	0	1	1	1	1	2	1	1	1	1	1	0	1	1	2	0	0	0	1	1	1	0
K03977	engA	ribosome-associated GTPase EngA	467 ± 46	97.3	99.8	1	1	1	1	0	1	1	1	1	1	1	1	1	1	1	0	1	1	0	1	2	0	1	0	1	0
K02519	infB	translation initiation factor IF-2	865 ± 127	98.5	99.6	1	1	1	1	1	1	1	1	1	1	1	1	1	1	1	2	2	1	1	0	0	0	2	1	1	0
K03664	smpB[d]	SmpB protein	156 ± 10	98.0	99.5	2	1	1	1	0	1	1	1	1	1	1	1	1	1	1	0	1	1	1	1	0	0	1	1	1	0
K03596	lepA	GTP-binding protein LepA	604 ± 22	98.4	99.4	1	1	1	1	0	1	1	1	1	1	1	1	2	1	1	1	2	1	3	3	1	0	1	1	1	0
K03545	tig	trigger factor	445 ± 23	97.9	99.4	1	1	1	1	0	1	1	1	1	1	1	1	1	1	1	1	1	1	1	0	0	0	1	0	1	0
K03438	mraW	MraW methylase family	318 ± 22	98.5	99.3	1	1	1	1	0	1	1	1	1	1	2	1	2	1	1	1	2	2	1	1	0	0	1	1	1	0
K02520	infC[d]	translation initiation factor IF-3	176 ± 39	96.4	98.7	1	1	1	1	0	1	1	1	1	1	1	1	1	1	1	1	1	3	1	0	0	0	1	1	1	0
K03043	rpoB[d]	DNA-directed RNA polymerase, beta subunit	1274 ± 108	95.5	99.5	1	2	1	1	1	1	1	2	1	1	1	1	1	1	2	1	1	1	2	2	0	0	3	1	3	0
K02316	dnaG[d]	DNA primase	606 ± 57	97.8	98.2	1	1	1	3	1	1	1	1	1	1	1	1	1	2	2	1	1	1	2	5	0	1	1	1	1	0
K03553	recA	recA protein	358 ± 61	96.6	96.8	1	1	1	1	0	1	1	1	1	1	2	1	1	1	1	1	1	1	3	3	3	0	1	1	1	0
K02963	rpsR	ribosomal protein S18	83 ± 11	98.2	96.8	1	1	1	1	0	1	1	1	1	1	1	1	1	1	1	1	1	1	1	0	0	0	1	1	1	0
K00942	gmk	guanylate kinase	207 ± 24	95.7	96.6	1	1	1	1	0	2	1	0	1	1	1	1	1	1	1	1	1	1	1	0	0	0	1	1	1	0
K02338	dnaN	DNA polymerase III, beta subunit	375 ± 16	98.3	94.7	1	1	1	1	0	1	1	1	1	1	1	1	1	2	1	1	1	1	2	0	0	0	1	1	1	0
K02902	rpmB	ribosomal protein L28	75 ± 13	96.1	94.4	0	1	0	1	0	0	1	1	1	1	1	1	0	1	1	1	1	1	1	0	0	0	0	0	0	0
K02313	dnaA	chromosomal replication initiator protein DnaA	469 ± 54	96.6	94.1	2	1	1	1	0	1	2	1	1	2	1	1	1	1	1	1	1	1	1	0	0	0	1	2	1	0
K07042	ybeY	probable rRNA maturation factor	164 ± 31	93.8	99.9	1	1	1	0	0	1	1	1	1	1	1	1	1	1	1	1	1	2	1	0	0	0	1	1	1	0
K00859	coaE	dephospho-CoA kinase	212 ± 42	94.9	99.7	2	1	1	1	1	0	1	1	1	1	0	1	1	1	1	2	1	1	2	1	0	1	1	1	1	0
K03595	era	GTP-binding protein Era	306 ± 19	92.2	99.6	1	1	1	1	0	1	1	1	1	1	1	1	1	1	1	0	1	0	1	2	0	0	1	1	1	0
K03075	secG[f]	preprotein translocase, SecG subunit	105 ± 30	95.2	99.8	0	1	1	1	0	0	1	1	1	0	0	1	1	1	1	1	1	0	1	0	0	0	0	1	0	0
K03073	secE[f]	preprotein translocase, SecE subunit	92 ± 32	94.7	99.6	0	1	0	1	0	0	1	1	1	1	0	1	1	0	1	1	0	0	0	0	0	0	1	0	0	0
K04043	dnaK[f]	chaperone protein DnaK	629 ± 35	97.9	91.6	1	1	1	1	1	1	1	3	1	1	2	1	1	1	1	1	0	4	1	0	0	0	1	1	2	0
K02470	gyrB[fe]	DNA gyrase, B subunit	724 ± 94	97.6	88.5	1	1	1	1	1	2	1	2	1	1	1	1	1	1	1	1	2	3	4	3	1	0	1	1	2	0
K01972	ligA[f]	DNA ligase, NAD-dependent	675 ± 80	96.9	88.3	1	1	1	1	0	1	1	1	1	1	1	1	1	1	1	2	1	1	1	3	3	0	1	1	1	0
K02469	gyrA[f]	DNA gyrase, A subunit	852 ± 82	97.6	86.6	1	1	1	1	3	2	1	2	1	1	1	1	1	1	1	1	1	2	1	1	0	0	1	1	2	0
K03070	secA[f]	preprotein translocase, SecA subunit	889 ± 79	99.2	86.1	2	1	1	1	0	1	1	1	1	1	1	1	1	1	1	1	2	3	2	2	0	0	1	1	2	0
K02911	rpmF[f]	ribosomal protein L32	59 ± 7	93.5	96.6	0	0	1	1	0	1	0	0	0	0	0	1	0	1	1	0	1	0	0	0	0	0	1	1	1	0
K00566	mnmA[f]	tRNA (5-methylaminomethyl-2-thiouridylate)-methyltransferase	370 ± 25	95.0	93.7	2	1	1	1	0	1	2	1	1	2	1	1	2	2	1	1	2	0	0	0	0	0	2	1	1	0
K02914	rpmH[f]	ribosomal protein L34	46 ± 4	88.2	99.9	0	0	0	0	0	0	0	0	0	0	1	0	0	0	1	0	1	0	0	0	0	0	0	0	1	0
K01879	glyS[f]	glycyl-tRNA synthetase beta chain	693 ± 32	64.6	100.0	1	1	1	0	0	0	1	0	0	1	1	1	0	0	0	0	0	0	0	2	0	0	1	1	0	0
K01869	leuS[f]	leucyl-tRNA synthetase	859 ± 61	47.4	26.6	1	1	1	1	1	1	1	1	2	1	1	1	1	1	1	1	2	1	1	0	0	1	1	1	1	0

[a] Bacterial genomes having the housekeeping gene / Total 1943 KEGG organisms (in Nov, 2012) × 100 (%). Red colored, <95%; Orange colored, <97%.
[b] Bacterial genomes having the housekeeping gene with single copy / Total bacterial genomes having the housekeeping gene × 100 (%). Red colored, <95%; Orange colored, <97%.
[c] Seventeen single-copied housekeeping genes (seven for ribosomal proteins) used for core gene-based microbial community analysis.
[d] Thirty protein encoding phylogenetic marker genes used for AMPHORA program (Wu et al. 2008). The column yellow-highlighted.
[e] Three single-copied housekeeping genes (rpsG, tilS, and gyrB) used for phylogenetic tree analysis.
[f] Twelve single-copied housekeeping genes were not used for draft genome completeness estimation because of their low qualities.
[g] Column was colored depend on number of ORF annotated to specific KO within each bin-genome. White column was counted for draft genome completeness.
[h] Column with red letter showed multi-copied partial ORFs annotated to same KO, and the sum of the peptide length was similar to averaged peptide length, which treated as single copy existence.

Supplementary Table S9. Single copied gene list for validation of draft genome assembly within domain *Archaea* [a]

KO	KO discription	Bin-genome L5met	Bin-genome L21met	*Methanosalsum zhilinae*	*Methanococcoides burtonii*
K00096	glycerol-1-phosphate dehydrogenase [NAD(P)] [EC:1.1.1.261]	1	2	1	1
K00555	tRNA (guanine-N2-)-methyltransferase [EC:2.1.1.32]	1	0	1	1
K00586	diphthine synthase [EC:2.1.1.98]	3	1	1	1
K00609	aspartate carbamoyltransferase catalytic subunit [EC:2.1.3.2]	1	0	1	1
K00611	ornithine carbamoyltransferase [EC:2.1.3.3]	1	0	1	1
K00946	thiamine-monophosphate kinase [EC:2.7.4.16]	1	0	1	1
K01251	adenosylhomocysteinase [EC:3.3.1.1]	2	2	1	1
K01265	methionyl aminopeptidase [EC:3.4.11.18]	1	0	1	1
K01591	orotidine-5'-phosphate decarboxylase [EC:4.1.1.23]	1	1	1	1
K01803	triosephosphate isomerase (TIM) [EC:5.3.1.1]	1	2	1	1
K01807	ribose 5-phosphate isomerase A [EC:5.3.1.6]	1	1	1	1
K01868	threonyl-tRNA synthetase [EC:6.1.1.3]	1	2	1	1
K01870	isoleucyl-tRNA synthetase [EC:6.1.1.5]	4	1	1	1
K01872	alanyl-tRNA synthetase [EC:6.1.1.7]	2	2	1	1
K01873	valyl-tRNA synthetase [EC:6.1.1.9]	1	2	1	1
K01874	methionyl-tRNA synthetase [EC:6.1.1.10]	2	1	1	1
K01876	aspartyl-tRNA synthetase [EC:6.1.1.12]	1	1	1	1
K01880	glycyl-tRNA synthetase [EC:6.1.1.14]	2	1	1	1
K01881	prolyl-tRNA synthetase [EC:6.1.1.15]	1	0	1	1
K01885	glutamyl-tRNA synthetase [EC:6.1.1.17]	1	0	1	1
K01887	arginyl-tRNA synthetase [EC:6.1.1.19]	1	2	1	1
K01889	phenylalanyl-tRNA synthetase alpha chain [EC:6.1.1.20]	2	1	1	1
K01890	phenylalanyl-tRNA synthetase beta chain [EC:6.1.1.20]	2	2	1	1
K01892	histidyl-tRNA synthetase [EC:6.1.1.21]	2	1	1	1
K01937	CTP synthase [EC:6.3.4.2]	1	0	1	1
K01975	2'-5' RNA ligase [EC:6.5.1.-]	1	1	1	1
K02117	V-type H+transporting ATPase subunit A [EC:3.6.3.14]	1	0	1	1
K02118	V-type H+transporting ATPase subunit B [EC:3.6.3.14]	1	0	1	1
K02528	16S rRNA -dimethyltransferase [EC:2.1.1.182]	1	1	1	1
K02601	transcriptional antiterminator NusG	1	0	1	1
K02683	DNA primase [EC:2.7.7.-]	1	1	1	1
K02863	large subunit ribosomal protein L1	1	0	1	1
K02864	large subunit ribosomal protein L10	1	0	1	1
K02866	large subunit ribosomal protein L10e	1	1	1	1
K02867	large subunit ribosomal protein L11	1	0	1	1
K02869	large subunit ribosomal protein L12	1	0	1	1
K02871	large subunit ribosomal protein L13	1	0	1	1
K02874	large subunit ribosomal protein L14	1	0	1	1
K02876	large subunit ribosomal protein L15	1	0	1	1
K02877	large subunit ribosomal protein L15e	1	0	1	1
K02881	large subunit ribosomal protein L18	1	0	1	1
K02883	large subunit ribosomal protein L18e	1	0	1	1
K02885	large subunit ribosomal protein L19e	1	0	1	1
K02886	large subunit ribosomal protein L2	1	0	1	1
K02889	large subunit ribosomal protein L21e	1	1	1	1
K02890	large subunit ribosomal protein L22	1	0	1	1
K02895	large subunit ribosomal protein L24	1	0	1	1
K02896	large subunit ribosomal protein L24e	1	0	1	1
K02906	large subunit ribosomal protein L3	1	0	1	1
K02907	large subunit ribosomal protein L30	1	0	1	1
K02910	large subunit ribosomal protein L31e	1	0	1	1
K02912	large subunit ribosomal protein L32e	1	0	1	1
K02921	large subunit ribosomal protein L37Ae	1	0	1	1
K02927	large subunit ribosomal protein L40e	0	0	1	1
K02929	large subunit ribosomal protein L44e	1	0	1	1
K02930	large subunit ribosomal protein L4e	1	0	1	1
K02931	large subunit ribosomal protein L5	1	0	1	1
K02933	large subunit ribosomal protein L6	1	0	1	1
K02936	large subunit ribosomal protein L7Ae	1	0	1	1
K02946	small subunit ribosomal protein S10	1	0	1	1
K02948	small subunit ribosomal protein S11	1	0	1	1
K02950	small subunit ribosomal protein S12	1	0	1	1
K02952	small subunit ribosomal protein S13	1	0	**0**	1
K02956	small subunit ribosomal protein S15	1	0	1	1
K02961	small subunit ribosomal protein S17	1	0	1	1
K02965	small subunit ribosomal protein S19	1	0	1	1
K02966	small subunit ribosomal protein S19e	1	0	1	1
K02967	small subunit ribosomal protein S2	1	0	1	1
K02974	small subunit ribosomal protein S24e	1	1	1	1
K02978	small subunit ribosomal protein S27e	1	0	1	1

Supplementary Table S9. *Continued.*

KO	KO discription	Bin-genome L5met	Bin-genome L21met	*Methanosalsum zhilinae*	*Methanococcoides burtonii*
K02979	small subunit ribosomal protein S28e	1	0	1	1
K02982	small subunit ribosomal protein S3	1	0	1	1
K02984	small subunit ribosomal protein S3Ae	1	1	1	1
K02986	small subunit ribosomal protein S4	1	0	1	1
K02987	small subunit ribosomal protein S4e	1	0	1	1
K02988	small subunit ribosomal protein S5	1	0	1	1
K02991	small subunit ribosomal protein S6e	1	1	1	1
K02992	small subunit ribosomal protein S7	1	0	1	1
K02994	small subunit ribosomal protein S8	1	0	1	1
K02995	small subunit ribosomal protein S8e	1	0	1	1
K02996	small subunit ribosomal protein S9	1	0	1	1
K03041	DNA-directed RNA polymerase subunit A' [EC:2.7.7.6]	1	0	1	1
K03042	DNA-directed RNA polymerase subunit A" [EC:2.7.7.6]	1	0	1	1
K03047	DNA-directed RNA polymerase subunit D [EC:2.7.7.6]	1	0	1	1
K03049	DNA-directed RNA polymerase subunit E' [EC:2.7.7.6]	1	1	1	1
K03050	DNA-directed RNA polymerase subunit E" [EC:2.7.7.6]	1	1	1	1
K03053	DNA-directed RNA polymerase subunit H [EC:2.7.7.6]	0	0	1	1
K03055	DNA-directed RNA polymerase subunit K [EC:2.7.7.6]	1	0	1	1
K03056	DNA-directed RNA polymerase subunit L [EC:2.7.7.6]	1	0	1	1
K03058	DNA-directed RNA polymerase subunit N [EC:2.7.7.6]	0	0	1	1
K03076	preprotein translocase subunit SecY	2	0	1	1
K03106	signal recognition particle subunit SRP54	1	0	1	1
K03110	fused signal recognition particle receptor	1	1	1	1
K03232	elongation factor 1-beta	1	1	1	0
K03234	elongation factor 2	1	0	1	1
K03237	translation initiation factor 2 subunit 1	1	1	1	1
K03242	translation initiation factor 2 subunit 3	1	1	1	1
K03243	translation initiation factor 5B	1	0	1	1
K03263	translation initiation factor 5A	1	2	1	1
K03264	translation initiation factor 6	1	0	1	1
K03265	peptide chain release factor subunit 1	1	1	1	1
K03330	glutamyl-tRNA(Gln) amidotransferase subunit E [EC:6.3.5.7]	1	2	1	1
K03470	ribonuclease HII [EC:3.1.26.4]	2	2	1	1
K03626	nascent polypeptide-associated complex subunit alpha	1	0	1	1
K03627	putative transcription factor	1	1	1	1
K04483	DNA repair protein RadA	1	1	1	1
K04794	peptidyl-tRNA hydrolase, PTH2 family [EC:3.1.1.29]	1	0	1	1
K04795	fibrillarin-like pre-rRNA processing protein	2	1	1	1
K04798	prefoldin beta subunit	1	1	1	1
K04799	flap endonuclease-1 [EC:3.-.-.-]	1	1	1	1
K04800	replication factor C large subunit	1	0	1	1
K06174	ATP-binding cassette, sub-family E, member 1	3	2	1	1
K06865	ATPase	2	1	1	1
K06875	programmed cell death protein 5	1	0	1	1
K06943	nucleolar GTP-binding protein	1	1	1	1
K06944	Predicted GTPase	1	1	1	1
K06961	ribosomal RNA assembly protein	1	0	1	1
K06965	protein pelota	1	0	1	1
K07041	Predicted metal-dependent Rnase	1	1	1	1
K07060	UPF0271 protein	1	1	1	1
K07178	RIO kinase 1 [EC:2.7.11.1]	1	0	1	1
K07558	tRNA nucleotidyltransferase (CCA-adding enzyme) [EC:2.7.7.72]	1	1	1	1
K07561	diphthamide synthase subunit DPH2	1	1	1	1
K07566	tRNA threonylcarbamoyladenosine biosynthesis protein	2	1	1	1
K07572	putative nucleotide binding protein	1	1	1	1
K07575	PUA domain protein	1	2	0	1
K07580	hypothetical protein	0	0	1	1
K07583	tRNA pseudouridine synthase 10 [EC:5.4.99.-]	1	1	1	1
K07732	riboflavin kinase, archaea type [EC:2.7.1.161]	1	1	1	1
K09141	hypothetical protein	1	0	1	1
K09482	glutamyl-tRNA(Gln) amidotransferase subunit D [EC:6.3.5.7]	1	1	1	1
K09735	hypothetical protein	1	1	1	1
K09903	uridylate kinase [EC:2.7.4.22]	1	1	1	1
K11130	H/ACA ribonucleoprotein complex subunit 3	0	0	1	1
K14415	tRNA-splicing ligase RtcB [EC:6.5.1.3]	1	1	1	1
K14574	ribosome maturation protein SDO1	1	0	1	1

[a] Column was colored depend on number of ORF annotated to specific KO within each bin-genome. White column was counted for draft genome completeness. Column with red letter showed multi-copied partial ORFs annotated to same KO, and the sum of the peptide length was similar to averaged peptide length, which treated as single copy existence.

Supplementary Table S10. Minimum Information about a Metagenome-Assembled Genome (MIMAG)

Bin Name	Genome classification	Completeness[a]	Contamination[a]	No. of tRNAs	5S	16 S	23 S	Marker Lineage[a]	No. Markers[a]	0[a]	1[a]	2[a]	3+[a]
**HAgeoA**	Medium-quality draft	78.5	1.5	15	Y	Y	Y	c__Deltaproteobacteria	247	42	202	3	0
**H1geoB**	Low-quality draft	9.4	1.8	11	Y	N	Y	k__Bacteria	103	93	9	1	0
**H1geoC**	Low-quality draft	7.7	0.0	10	N	N	N	k__Bacteria	103	95	8	0	0
**H1geoD**	Low-quality draft	28.9	0.0	10	N	N	N	k__Bacteria	103	84	19	0	0
**H2geo**	High-quality draft	**99.4**	0.0	20	Y	Y	Y	c__Deltaproteobacteria	247	1	246	0	0
**H3lac**	High-quality draft	**97.4**	0.1	18	Y	Y	Y	o__Lactobacillales	472	13	458	1	0
**H4act**	High-quality draft	**95.2**	0.0	20	Y	Y	Y	o__Actinomycetales	330	12	318	0	0
**H5geo**	High-quality draft	**100.0**	1.0	20	Y	Y	Y	c__Deltaproteobacteria	247	1	243	3	0
**H6fir**	High-quality draft	**100.0**	1.5	19	Y	Y	Y	p__Firmicutes	295	0	292	3	0
**H7geo**	Medium-quality draft	**98.7**	0.0	20	N	N	Y	c__Deltaproteobacteria	246	3	243	0	0
**H8spi**	High-quality draft	**98.9**	0.0	20	Y	Y	Y	k__Bacteria	141	1	140	0	0
**H9bac**	High-quality draft	**100.0**	0.8	20	Y	Y	Y	k__Bacteria	278	0	276	2	0
**H10eps**	High-quality draft	**99.6**	0.7	20	N	Y	Y	p__Proteobacteria	395	1	391	3	0
**H11bac**	High-quality draft	**95.6**	1.6	20	Y	Y	Y	k__Bacteria	278	11	264	3	0
**H12unc**	High-quality draft	94.7	1.3	20	Y	Y	Y	k__Bacteria	185	7	176	2	0
**M1geoA**	High-quality draft	91.5	0.6	18	Y	Y	Y	c__Deltaproteobacteria	246	20	225	1	0
**M1geoB**	Low-quality draft	10.1	1.8	6	N	N	N	k__Bacteria	103	94	8	1	0
**M1geoC**	Low-quality draft	6.5	0.0	5	N	N	N	k__Bacteria	103	97	6	0	0
**M1geoD**	Low-quality draft	14.4	0.0	7	N	N	N	k__Bacteria	103	92	11	0	0
**M2lac**	High-quality draft	**98.1**	0.5	18	Y	Y	Y	o__Lactobacillales	472	10	460	2	0
**M3ppro**	High-quality draft	**100.0**	0.0	19	N	Y	Y	c__Deltaproteobacteria	246	0	246	0	0
**M4geo**	High-quality draft	**99.4**	0.0	19	Y	N	Y	c__Deltaproteobacteria	247	1	246	0	0
**M5fir**	High-quality draft	**100.0**	1.3	20	Y	Y	Y	p__Firmicutes	295	0	293	2	0
**M6act**	High-quality draft	**96.4**	0.1	20	Y	Y	Y	o__Actinomycetales	330	9	320	1	0
**M7geo**	High-quality draft	**98.1**	3.2	19	Y	N	Y	c__Deltaproteobacteria	247	4	235	8	0
**M8spi**	High-quality draft	**98.9**	0.0	20	Y	Y	Y	k__Bacteria	141	1	140	0	0
**M9unc**	High-quality draft	91.3	0.7	20	N	Y	Y	k__Bacteria	185	14	167	4	0
**M10bac**	High-quality draft	**97.1**	4.5	19	Y	Y	Y	p__Bacteroidetes	316	7	298	11	0
**M11eps**	High-quality draft	**100.0**	1.2	20	N	Y	Y	p__Proteobacteria	395	0	390	5	0
**M12geo**	Medium-quality draft	**97.4**	1.4	13	N	N	N	c__Deltaproteobacteria	246	7	235	4	0
**M13tol**	High-quality draft	**99.9**	1.1	18	Y	Y	Y	c__Gammaproteobacteria	312	1	309	2	0
**M14spi**	High-quality draft	93.8	1.9	19	Y	Y	Y	k__Bacteria	141	9	129	3	0
**M15fir**	Medium-quality draft	**96.7**	4.8	20	Y	N	N	p__Firmicutes	295	12	272	10	1
**M17geo**	Medium-quality draft	78.5	1.2	16	N	N	N	c__Deltaproteobacteria	247	79	164	4	0
**M18tol**	Medium-quality draft	54.3	0.0	5	N	N	N	k__Bacteria	104	66	38	0	0
**M19bac**	Medium-quality draft	88.1	4.7	18	Y	Y	Y	k__Bacteria	278	27	240	11	0
**M20fir**	Low-quality draft	27.0	0.6	5	N	N	N	p__Firmicutes	294	214	79	1	0
**M21unc**	Low-quality draft	45.4	2.6	13	Y	N	N	k__Bacteria	185	92	90	3	0
**L1geo**	High-quality draft	**99.3**	1.3	20	N	Y	Y	c__Deltaproteobacteria	246	3	241	2	0
**L2tol**	High-quality draft	**100.0**	0.6	18	Y	Y	Y	c__Gammaproteobacteria	312	0	310	2	0
**L3tol**	Medium-quality draft	**96.8**	0.5	18	Y	N	N	c__Gammaproteobacteria	312	35	276	1	0
**L4bac**	High-quality draft	**96.8**	1.0	19	Y	Y	Y	o__Bacteroidales	427	13	410	4	0
**L5met**	Medium-quality draft	86.2	0.9	19	Y	Y	Y	p__Euryarchaeota	234	27	205	2	0
**L6fir**	High-quality draft	**98.9**	1.1	20	Y	Y	Y	k__Bacteria	149	1	147	1	0
**L7ppro**	High-quality draft	**96.5**	0.2	20	N	Y	Y	c__Deltaproteobacteria	246	18	227	1	0
**L8spi**	High-quality draft	92.9	0.0	20	Y	Y	Y	k__Bacteria	141	9	132	0	0
**L9bac**	High-quality draft	**99.5**	4.5	20	Y	N	Y	p__Bacteroidetes	316	1	304	11	0
**L10gmet**	High-quality draft	**98.1**	0.1	20	Y	Y	Y	c__Deltaproteobacteria	247	6	238	2	1
**L11pro**	High-quality draft	**99.1**	1.8	20	Y	Y	Y	k__Bacteria	185	1	182	2	0
**L12lac**	Medium-quality draft	**97.9**	0.1	17	Y	Y	Y	o__Lactobacillales	472	11	460	1	0
**L13fir**	Medium-quality draft	**98.6**	0.7	15	Y	N	Y	o__Clostridiales	249	2	244	3	0
**L14bac**	Medium-quality draft	**97.7**	0.8	19	N	N	N	o__Bacteroidales	486	7	470	9	0
**L15bac**	Medium-quality draft	**98.4**	0.0	18	Y	N	N	o__Bacteroidales	406	10	396	0	0
**L16bac**	Medium-quality draft	91.4	1.6	15	N	N	N	k__Bacteria	278	16	259	3	0
**L17bac**	Medium-quality draft	92.1	2.3	17	N	N	N	k__Bacteria	278	27	241	10	0
**L18ver**	Medium-quality draft	94.4	4.4	18	Y	N	N	k__Bacteria	230	13	209	8	0
**L19bac**	Medium-quality draft	84.3	1.8	14	Y	Y	N	p__Bacteroidetes	316	55	253	8	0
**L20tol**	Low-quality draft	9.5	0.0	0	N	N	N	k__Bacteria	104	98	6	0	0
**L20tolB**	Low-quality draft	3.9	0.0	0	N	N	N	k__Bacteria	104	99	5	0	0
**L21met**	Low-quality draft	37.9	0.0	11	N	N	N	k__Archaea	149	103	46	0	0
**L22fir**	High-quality draft	**98.1**	1.6	18	N	Y	Y	o__Selenomonadales	334	10	312	11	1
**L23elu**	Medium-quality draft	91.0	0.0	19	Y	N	N	k__Bacteria	143	8	135	0	0
**L24fir**	Medium-quality draft	75.3	1.9	17	N	N	N	k__Bacteria	161	36	123	2	0

[a] Genome completeness and contamination were anlyzed by using CheckM software.

Supplementary Table S11. Bin-genome linkage to phylotypes observed in 16S rRNA clone analyses.

Taxonomy Phylotype	H-a5[a]	M-a5[a]	L-a5[a]	% match	Best matched sequence	Accesion No.	Bin-genome linkage		
							SPH	SPM	SPL
Proteobacteria - d-Proteobacteria									
**MEC_GeoH3**	23	-	-	98	*Geobacter humireducens*	AY187306	H1geo(pan)	M17geo	-
**Gl1**	6	-	-	96	*Geobacter* sp. CdA-2	Y19190	H2geo	M4geo	-
**Su5**	-	7	-	98	*Pelobacter propionicus* DSM 2379	CP000482	-	M3ppro	-
**Gl2**	-	-	-	97	*Geobacter* sp. Ply1	EF527233	-	-	L7ppro[b]
**Gl15**	-	19	4	95	*Geobacter chapelleii*	U41561	-	M1geo(pan)	L1geo
**MEC_GeoM2**	-	7	-	99	*Geobacter bremensis*	JN795198	-	M7geo[b]	-
**Su6**	-	-	-	99	*Geobacter* sp. OSK6	AB682759	H5geo	-	-
**Ha4_I01**	4	-	-	99	*Geobacter* sp. CLFeRB	DQ086800	H7geo	M12geo	-
**Geo1**	-	2	-	96	*Geobacter metallireducens* GS-15	CP000148	-	-	L10gmet
**Ha2_G01**	-	1	-	99	*Desulfovibrio desulfuricans* SRB-22	FJ873799	-	-	-
Proteobacteria - g-Proteobacteria									
							-	M13tol	L3tol
**Tol5**	3	22	69	99	*Tolumonas* sp. OCF	GU370947	-	-	L2tol
							-	M18tol	-
**MEC_TOL2**	10	-	1	96	*Tolumonas auensis* DSM 9187	CP001616	γ-Proteobacteria	-	L20tol
**Gl13**	1	-	-	97	*Aeromonas sharmana* GPTSA-6	DQ013306	-	-	-
Proteobacteria - e-Proteobacteria									
**Ma3_I21**	2	6	-	98	*Sulfurospirillum deleyianum* DSM 6946	CP001816	H10eps	M11eps	-
Firmicutes - Bacilli									
**Gl17**	33	10	-	100	*Lactococcus* sp. JXZ-2	JF496551	H3lac	M2lac	L12lac
Firmicutes - Clostridia / Negativicutes									
**Gl18**	7	10	-	99	*Anaerovibrio burkinabensis* DSM6283	AJ010961	H6fir	M5fir	-
**Gl13**	-	1	4	98	*Acetobacterium submarinus*	AY485791	-	-	L13fir
**Ma4_P11**	-	2	-	99	*Anaeroarcus burkinensis* DSM 6283	NR_025298	-	M15fir[b]	-
**Hb2_E04**	-	-	3	92	*Phascolarctobacterium* sp. YIT 12068	AB490812	-	-	L6fir
**Hb1_O19**	-	-	-	90	*Acidaminococcus intestini* ADV 255.99	NR_041894	-	-	L22fir
**Ma3_I07**	1	1	-	94	*Acidaminococcus fermentans* DSM20731	CP001859	-	-	-
Unlinked	-	-	-		Unlinked		-	M20fir	-
Bacteroidetes									
**Su2**	-	-	3	99	*Parabacteroides* sp. Lind7H	HQ020488	-	-	L4bac
**La4_A15**	-	-	-	90	*Paludibacter propionicigenes* WB4	CP002345	H9bac	-	-
**La3_B11**	-	1	-	90	*Porphyromonadaceae* bacterium C941	JF803519	-	M10bac	L9bac
**Gl10**	1	1	2	85	*Bacteroidetes* bacterium 4F6B	AB623230	H11bac	M19bac	L16bac
Unlinked	-	-	-		Unlinked *Bacteroidetes*		-	-	L14bac
Unlinked	-	-	-		Unlinked *Bacteroidetes*		-	Bact	L15bac
Unlinked	-	-	-		Unlinked *Bacteroidetes*		-	-	L17bac
Unlinked	-	-	-		Unlinked *Bacteroidetes*		-	-	L19bac
Other clones	4	1	4						
**Hb4_A12**	-	-	-	95	*Geothrix fermentans* H5	NR_036779	H12unc	M21unc	L11pro
Unlinked	-	-	-	98	*Geothrix fermentans* HradG1	HF559181	-	M9unc	-
Unlinked	-	-	-	99	*Propionicimonas paludicola* Wd	NR_104769	H4act	M6act	-
Unlinked	-	-	-	95	*Treponema stenostreptum* 3S-CHLI6-a	MG264272	H8spi	M8spi	L8spi
Unlinked	-	-	-	94	*Treponema* sp. HM	KP297860	-	M14spi	-
Unlinked	-	-	-		Unlinked *Verrucomicrobia*		-	-	L18ver
Unlinked	-	-	-		Unlinked Bacteria		-	-	L23elu
Euryarcheota									
Unlinked	-	-	-	99	*Methanocorpusculum sinense* DSM 4274	NR117148	-	-	L5met
Unlinked	-	-	-		Unlinked		-	-	L21met
Total clones	95	91	90			Total Bingenomes	12	20	23

[a] 16S rRNA basis clone analyses werereferred from our previous work (Ishii et al. 2015. ISME J. 8:963)

[b] Linkage was estimated from phylogenetic trees.

Supplementary Table 12. Analysis of ANIb and TETRA among the recovered bin-genomes for family *Geobacteraceae* / *Pelobacteraceae* in the communities

* Operational Candidate Species (OCS) is defined by TETRA >0.99 and ANIb >97

TETRA	H1geoA	M17geo	H1geoD	H1geoB	H1geoC	*G. pelophilus*	M7geo	*G. bremensis*	*G. bemidjiensis*	*Geobacter* sp. M21	*Geobacter* sp. M18	*G. sulfurreducens* KN400	*G. sulfurreducens* PCA	*G. soli*	*G. anodireducens*	*G. pickeringii*	*G. metallireducens*	L10gmet	H2geo	M4geo	*G. uraniireducens*	*G. daltonii*	H5geo	*Geobacter* sp. OR1	M1geoD	M1geoB	M1geoC	L1geo	M1geoA	*P. propionicus*	L7ppro	M3ppro	M12geo	H7geo	*Geopsychrobacter electrodiphilus*	*Geothermobacter* sp. EPRM	*Geoalkalibacter subterraneus*	*Geoalkalibacter ferrihydriticus*	*G. lovleyi*	*G. thiogenes*	*Geothrix fermentans*
H1geoA	---	1.00	1.00	0.99	0.99	0.99	0.99	0.98	0.98	0.98	0.98	0.89	0.89	0.89	0.89	0.93	0.90	0.85	0.85	0.85	0.86	0.79	0.86	0.77	0.81	0.80	0.80	0.78	0.76	0.86	0.85	0.68	0.73	0.71	0.81	0.86	0.83	0.80	0.71	0.63	0.66
M17geo	1.00	---	1.00	1.00	1.00	0.99	0.99	0.98	0.98	0.98	0.98	0.90	0.90	0.90	0.89	0.93	0.90	0.85	0.86	0.86	0.87	0.80	0.87	0.78	0.81	0.81	0.81	0.78	0.77	0.86	0.86	0.69	0.74	0.72	0.82	0.86	0.84	0.80	0.72	0.64	0.66
H1geoD	1.00	1.00	---	1.00	0.99	0.99	0.99	0.98	0.98	0.98	0.98	0.90	0.90	0.90	0.90	0.93	0.91	0.86	0.86	0.86	0.87	0.80	0.87	0.79	0.82	0.81	0.82	0.79	0.78	0.86	0.86	0.71	0.74	0.73	0.82	0.87	0.84	0.80	0.72	0.65	0.66
H1geoB	0.99	1.00	1.00	---	1.00	0.98	0.99	0.98	0.98	0.98	0.98	0.90	0.90	0.90	0.89	0.94	0.91	0.86	0.87	0.87	0.88	0.82	0.88	0.79	0.83	0.82	0.82	0.79	0.78	0.87	0.86	0.72	0.76	0.74	0.83	0.86	0.84	0.80	0.73	0.66	0.64
H1geoC	0.99	1.00	0.99	1.00	---	0.98	0.99	0.98	0.98	0.98	0.98	0.89	0.89	0.89	0.89	0.93	0.91	0.86	0.87	0.87	0.88	0.81	0.87	0.79	0.82	0.81	0.82	0.79	0.77	0.86	0.86	0.71	0.75	0.73	0.82	0.86	0.84	0.79	0.73	0.66	0.64
G. pelophilus	0.99	0.99	0.99	0.98	0.98	---	1.00	1.00	1.00	1.00	0.99	0.90	0.90	0.89	0.89	0.94	0.91	0.85	0.87	0.87	0.87	0.80	0.88	0.79	0.83	0.82	0.82	0.79	0.78	0.89	0.89	0.71	0.76	0.75	0.84	0.87	0.85	0.79	0.72	0.65	0.66
M7geo	0.99	0.99	0.99	0.99	0.99	1.00	---	1.00	1.00	1.00	0.99	0.91	0.91	0.90	0.90	0.94	0.92	0.87	0.88	0.88	0.89	0.82	0.89	0.81	0.84	0.84	0.84	0.81	0.80	0.90	0.90	0.73	0.78	0.77	0.85	0.88	0.86	0.80	0.74	0.67	0.65
G. bremensis	0.98	0.98	0.98	0.98	0.98	1.00	1.00	---	1.00	1.00	0.99	0.90	0.90	0.89	0.89	0.94	0.91	0.86	0.88	0.88	0.89	0.82	0.89	0.80	0.84	0.83	0.84	0.81	0.79	0.89	0.89	0.73	0.78	0.76	0.84	0.88	0.86	0.80	0.74	0.67	0.63
G. bemidjiensis	0.98	0.98	0.98	0.98	0.98	1.00	1.00	1.00	---	1.00	0.99	0.90	0.90	0.89	0.89	0.94	0.92	0.87	0.89	0.89	0.89	0.83	0.89	0.81	0.84	0.83	0.84	0.81	0.80	0.90	0.89	0.74	0.78	0.77	0.85	0.88	0.86	0.80	0.74	0.67	0.62
Geobacter sp. M21	0.98	0.98	0.98	0.98	0.98	1.00	1.00	1.00	1.00	---	0.99	0.89	0.89	0.89	0.89	0.94	0.91	0.86	0.87	0.87	0.88	0.81	0.88	0.79	0.82	0.82	0.83	0.79	0.78	0.89	0.89	0.71	0.76	0.75	0.83	0.87	0.85	0.79	0.73	0.65	0.63
Geobacter sp. M18	0.98	0.98	0.98	0.98	0.98	0.99	0.99	0.99	0.99	0.99	---	0.91	0.90	0.90	0.90	0.94	0.91	0.86	0.87	0.87	0.87	0.80	0.88	0.80	0.84	0.83	0.84	0.81	0.80	0.91	0.90	0.72	0.78	0.76	0.85	0.88	0.86	0.80	0.74	0.66	0.68
G. sulfurreducens KN400	0.89	0.90	0.90	0.90	0.89	0.90	0.91	0.90	0.90	0.89	0.91	---	1.00	1.00	1.00	0.94	0.98	0.96	0.95	0.95	0.95	0.91	0.96	0.91	0.94	0.94	0.94	0.93	0.92	0.94	0.93	0.85	0.87	0.86	0.86	0.89	0.89	0.85	0.83	0.76	0.64
G. sulfurreducens PCA	0.89	0.90	0.90	0.90	0.89	0.90	0.91	0.90	0.90	0.89	0.90	1.00	---	1.00	1.00	0.94	0.98	0.96	0.95	0.95	0.95	0.91	0.96	0.91	0.94	0.94	0.94	0.93	0.92	0.94	0.93	0.86	0.87	0.86	0.86	0.89	0.90	0.85	0.84	0.76	0.63
G. soli	0.89	0.90	0.90	0.90	0.89	0.89	0.90	0.89	0.89	0.89	0.90	1.00	1.00	---	1.00	0.93	0.96	0.95	0.93	0.93	0.93	0.89	0.95	0.89	0.93	0.93	0.93	0.92	0.92	0.93	0.93	0.83	0.85	0.84	0.85	0.89	0.89	0.85	0.82	0.74	0.66
G. anodireducens	0.89	0.89	0.90	0.89	0.89	0.89	0.90	0.89	0.89	0.89	0.90	1.00	1.00	1.00	---	0.93	0.96	0.95	0.93	0.93	0.93	0.89	0.95	0.89	0.93	0.93	0.93	0.92	0.92	0.93	0.93	0.83	0.85	0.84	0.85	0.89	0.89	0.84	0.82	0.74	0.66
G. pickeringii	0.93	0.93	0.93	0.94	0.93	0.94	0.94	0.94	0.94	0.94	0.94	0.94	0.94	0.93	0.93	---	0.98	0.94	0.93	0.93	0.92	0.86	0.91	0.84	0.86	0.86	0.87	0.83	0.82	0.91	0.90	0.77	0.83	0.82	0.82	0.85	0.84	0.77	0.78	0.71	0.60
G. metallireducens	0.90	0.90	0.91	0.91	0.91	0.91	0.92	0.91	0.92	0.91	0.91	0.98	0.98	0.96	0.96	0.98	---	0.98	0.97	0.97	0.96	0.91	0.95	0.89	0.92	0.92	0.92	0.90	0.89	0.94	0.93	0.85	0.88	0.87	0.86	0.88	0.88	0.81	0.82	0.76	0.58
L10gmet	0.85	0.85	0.86	0.86	0.86	0.85	0.87	0.86	0.87	0.86	0.86	0.96	0.96	0.95	0.95	0.94	0.98	---	0.98	0.98	0.97	0.94	0.94	0.91	0.93	0.93	0.93	0.92	0.91	0.92	0.92	0.90	0.90	0.89	0.85	0.86	0.88	0.82	0.83	0.77	0.52
H2geo	0.85	0.86	0.86	0.87	0.87	0.87	0.88	0.88	0.89	0.87	0.87	0.95	0.95	0.93	0.93	0.93	0.97	0.98	---	1.00	0.98	0.96	0.97	0.94	0.94	0.94	0.94	0.93	0.92	0.92	0.92	0.91	0.91	0.90	0.87	0.88	0.88	0.81	0.86	0.81	0.45
M4geo	0.85	0.86	0.86	0.87	0.87	0.87	0.88	0.88	0.89	0.87	0.87	0.95	0.95	0.93	0.93	0.93	0.97	0.98	1.00	---	0.98	0.96	0.97	0.94	0.94	0.94	0.94	0.93	0.92	0.92	0.92	0.91	0.91	0.90	0.87	0.88	0.88	0.81	0.86	0.81	0.45
G. uraniireducens	0.86	0.87	0.87	0.88	0.88	0.87	0.89	0.89	0.89	0.88	0.87	0.95	0.95	0.93	0.93	0.92	0.96	0.97	0.98	0.98	---	0.97	0.97	0.95	0.95	0.95	0.95	0.94	0.94	0.92	0.92	0.92	0.92	0.91	0.88	0.89	0.90	0.83	0.87	0.81	0.46
G. daltonii	0.79	0.80	0.80	0.82	0.81	0.80	0.82	0.82	0.83	0.81	0.80	0.91	0.91	0.89	0.89	0.86	0.91	0.94	0.96	0.96	0.97	---	0.95	0.95	0.94	0.94	0.94	0.94	0.93	0.87	0.87	0.93	0.89	0.88	0.83	0.85	0.87	0.80	0.89	0.84	0.38
H5geo	0.86	0.87	0.87	0.88	0.87	0.88	0.89	0.89	0.89	0.88	0.88	0.96	0.96	0.95	0.95	0.91	0.95	0.94	0.97	0.97	0.97	0.95	---	0.97	0.96	0.96	0.96	0.95	0.95	0.93	0.93	0.89	0.90	0.89	0.87	0.88	0.88	0.80	0.88	0.82	0.49
Geobacter sp. OR1	0.77	0.78	0.79	0.79	0.79	0.79	0.81	0.80	0.81	0.79	0.80	0.91	0.91	0.89	0.89	0.84	0.89	0.91	0.94	0.94	0.95	0.95	0.97	---	0.96	0.96	0.96	0.96	0.96	0.89	0.89	0.93	0.92	0.91	0.85	0.85	0.85	0.76	0.89	0.83	0.38
M1geoD	0.81	0.81	0.82	0.83	0.82	0.83	0.84	0.84	0.84	0.82	0.84	0.94	0.94	0.93	0.93	0.86	0.92	0.93	0.94	0.94	0.95	0.94	0.96	0.96	---	1.00	1.00	0.99	0.99	0.94	0.94	0.93	0.94	0.93	0.89	0.88	0.89	0.83	0.91	0.85	0.52
M1geoB	0.80	0.81	0.81	0.82	0.81	0.82	0.84	0.83	0.83	0.82	0.83	0.94	0.94	0.93	0.93	0.86	0.92	0.93	0.94	0.94	0.95	0.94	0.96	0.96	1.00	---	1.00	0.99	0.99	0.94	0.94	0.93	0.94	0.93	0.88	0.87	0.89	0.83	0.90	0.85	0.51
M1geoC	0.80	0.81	0.82	0.82	0.82	0.82	0.84	0.84	0.84	0.83	0.84	0.94	0.94	0.93	0.93	0.87	0.92	0.93	0.94	0.94	0.95	0.94	0.96	0.96	1.00	1.00	---	0.99	0.98	0.94	0.94	0.93	0.94	0.93	0.88	0.87	0.89	0.82	0.90	0.85	0.51
L1geo	0.78	0.78	0.79	0.79	0.79	0.79	0.81	0.81	0.81	0.79	0.81	0.93	0.93	0.92	0.92	0.83	0.90	0.92	0.93	0.93	0.94	0.94	0.95	0.96	0.99	0.99	0.99	---	1.00	0.93	0.93	0.93	0.93	0.92	0.87	0.87	0.88	0.82	0.90	0.84	0.50
M1geoA	0.76	0.77	0.78	0.78	0.77	0.78	0.80	0.79	0.80	0.78	0.80	0.92	0.92	0.92	0.92	0.82	0.89	0.91	0.92	0.92	0.94	0.93	0.95	0.96	0.99	0.99	0.98	1.00	---	0.92	0.92	0.93	0.92	0.92	0.87	0.86	0.88	0.82	0.90	0.84	0.49
P. propionicus	0.86	0.86	0.86	0.87	0.86	0.89	0.90	0.89	0.90	0.89	0.91	0.94	0.94	0.93	0.93	0.91	0.94	0.92	0.92	0.92	0.92	0.87	0.93	0.89	0.94	0.94	0.94	0.93	0.92	---	1.00	0.87	0.89	0.88	0.88	0.88	0.90	0.82	0.84	0.78	0.59
L7ppro	0.85	0.86	0.86	0.86	0.86	0.89	0.90	0.89	0.89	0.89	0.90	0.93	0.93	0.93	0.93	0.90	0.93	0.92	0.92	0.92	0.92	0.87	0.93	0.89	0.94	0.94	0.94	0.93	0.92	1.00	---	0.87	0.89	0.88	0.88	0.88	0.90	0.82	0.84	0.77	0.59
M3ppro	0.68	0.69	0.71	0.72	0.71	0.71	0.73	0.73	0.74	0.71	0.72	0.85	0.86	0.83	0.83	0.77	0.85	0.90	0.91	0.91	0.92	0.93	0.89	0.93	0.93	0.93	0.93	0.93	0.93	0.87	0.87	---	0.93	0.92	0.82	0.80	0.84	0.78	0.85	0.82	0.33
M12geo	0.73	0.74	0.74	0.76	0.75	0.76	0.78	0.78	0.78	0.76	0.78	0.87	0.87	0.85	0.85	0.83	0.88	0.90	0.91	0.91	0.92	0.89	0.90	0.92	0.94	0.94	0.94	0.93	0.92	0.89	0.89	0.93	---	1.00	0.87	0.82	0.86	0.77	0.87	0.83	0.44
H7geo	0.71	0.72	0.73	0.74	0.73	0.75	0.77	0.76	0.77	0.75	0.76	0.86	0.86	0.84	0.84	0.82	0.87	0.89	0.90	0.90	0.91	0.88	0.89	0.91	0.93	0.93	0.93	0.92	0.92	0.88	0.88	0.92	1.00	---	0.86	0.81	0.85	0.76	0.86	0.83	0.43
Geopsychrobacter electrodiphilus	0.81	0.82	0.82	0.83	0.82	0.84	0.85	0.84	0.85	0.83	0.85	0.86	0.86	0.85	0.85	0.82	0.86	0.85	0.87	0.87	0.88	0.83	0.87	0.85	0.89	0.88	0.88	0.87	0.87	0.88	0.88	0.82	0.87	0.86	---	0.91	0.92	0.86	0.82	0.76	0.53
Geothermobacter sp. EPRM	0.86	0.86	0.87	0.86	0.86	0.87	0.88	0.88	0.88	0.87	0.88	0.89	0.89	0.89	0.89	0.85	0.88	0.86	0.88	0.88	0.89	0.85	0.88	0.85	0.88	0.87	0.87	0.87	0.86	0.88	0.88	0.80	0.82	0.81	0.91	---	0.94	0.89	0.81	0.73	0.62
Geoalkalibacter subterraneus	0.83	0.84	0.84	0.84	0.84	0.85	0.86	0.86	0.86	0.85	0.86	0.89	0.90	0.89	0.89	0.84	0.88	0.88	0.88	0.88	0.90	0.87	0.88	0.85	0.89	0.89	0.89	0.88	0.88	0.90	0.90	0.84	0.86	0.85	0.92	0.94	---	0.92	0.83	0.76	0.58
Geoalkalibacter ferrihydriticus	0.80	0.80	0.80	0.80	0.79	0.79	0.80	0.80	0.80	0.79	0.80	0.85	0.85	0.85	0.84	0.77	0.81	0.82	0.81	0.81	0.83	0.80	0.80	0.76	0.83	0.83	0.82	0.82	0.82	0.82	0.82	0.78	0.77	0.76	0.86	0.89	0.92	---	0.71	0.64	0.56
G. lovleyi	0.71	0.72	0.72	0.73	0.73	0.72	0.74	0.74	0.74	0.73	0.74	0.83	0.84	0.82	0.82	0.78	0.82	0.83	0.86	0.86	0.87	0.89	0.88	0.89	0.91	0.90	0.90	0.90	0.90	0.84	0.84	0.85	0.87	0.86	0.82	0.81	0.83	0.71	---	0.98	0.45
G. thiogenes	0.63	0.64	0.65	0.66	0.66	0.65	0.67	0.67	0.67	0.65	0.66	0.76	0.76	0.74	0.74	0.71	0.76	0.77	0.81	0.81	0.81	0.84	0.82	0.83	0.85	0.85	0.85	0.84	0.84	0.78	0.77	0.82	0.83	0.83	0.76	0.73	0.76	0.64	0.98	---	0.36
Geothrix fermentans	0.66	0.66	0.66	0.64	0.64	0.66	0.65	0.63	0.62	0.63	0.68	0.64	0.63	0.66	0.66	0.60	0.58	0.52	0.45	0.45	0.46	0.38	0.49	0.38	0.52	0.51	0.51	0.50	0.49	0.59	0.59	0.33	0.44	0.43	0.53	0.62	0.58	0.56	0.45	0.36	---

Supplementary Table 12. *Continued*

* Operational Candidate Species (OCS) is defined by TETRA >0.99 and ANIb >97

ANIb	H1geoA	M17geo	H1geoD	H1geoB	H1geoC	*G. pelophilusta*	M7geo	*G. bremensis*	*G. bemidjiensis*	*Geobacter* sp. M21	*Geobacter* sp. M18	*G. sulfurreducens* KN400	*G. sulfurreducens* PCA	*G. soli*	*G. anodireducens*	*G. pickeringii*	*G. metallireducens*	L10gmet	H2geo	M4geo	*G. uraniireducens*	*G. daltonii*	H5geo	*Geobacter* sp. OR1	M1geoD	M1geoB	M1geoC	L1geo	M1geoA	*P. propionicus*	L7ppro	M3ppro	M12geo	H7geo	*Geopsychrobacter electrodiphilus*	*Geothermobacter* sp. EPRM	*Geoalkalibacter subterraneus*	*Geoalkalibacter ferrihydriticus*	*G. lovleyi*	*G. thiogenes*	*Geothrix fermentans*
H1geoA	---	92.5	90.8	73.6	87.7	78.8	78.8	78.7	78.6	78.8	82.1	na	na	na	na	na	na	na	na	na	na	na	na	na	na	na	na	na	na	na	na	na	na	na	na	na	na	na	na	na	na
M17geo	91.7	---	99.0	88.8	88.2	77.4	77.2	77.4	77.3	77.3	80.7	na	na	na	na	na	na	na	na	na	na	na	na	na	na	na	na	na	na	na	na	na	na	na	na	na	na	na	na	na	na
H1geoD	90.8	99.4	---	88.8	88.9	76.6	77.3	76.6	76.5	76.6	80.1	na	na	na	na	na	na	na	na	na	na	na	na	na	na	na	na	na	na	na	na	na	na	na	na	na	na	na	na	na	na
H1geoB	77.5	90.8	89.8	---	92.5	76.9	76.0	77.0	76.6	76.7	79.7	na	na	na	na	na	na	na	na	na	na	na	na	na	na	na	na	na	na	na	na	na	na	na	na	na	na	na	na	na	na
H1geoC	91.1	90.2	89.7	92.6	---	76.2	76.2	76.0	75.9	76.0	79.6	69.8	69.9	70.1	70.0	71.1	70.6	69.5	69.7	69.7	70.5	68.6	70.0	68.9	67.5	67.7	68.0	68.4	68.3	69.0	69.0	67.3	67.6	67.1	64.4	66.0	66.1	65.6	68.2	67.4	63.9
G. pelophilusta	78.0	76.8	75.3	75.1	74.8	---	96.0	87.3	87.4	87.4	79.2	na	na	na	na	na	na	na	na	na	na	na	na	na	na	na	na	na	na	na	na	na	na	na	na	na	na	na	na	na	na
M7geo	78.1	76.6	76.3	74.2	74.0	95.8	---	86.9	86.9	87.0	79.0	na	na	na	na	na	na	na	na	na	na	na	na	na	na	na	na	na	na	na	na	na	na	na	na	na	na	na	na	na	na
G. bremensis	77.9	76.8	75.4	75.9	74.3	87.3	86.9	---	95.3	91.6	79.4	na	na	na	na	na	na	na	na	na	na	na	na	na	na	na	na	na	na	na	na	na	na	na	na	na	na	na	na	na	na
G. bemidjiensis	77.9	76.7	75.4	75.2	74.3	87.3	86.9	95.4	---	91.7	79.3	70.3	70.3	70.5	70.6	71.7	71.2	69.9	70.1	70.1	70.9	69.7	70.0	68.9	68.0	67.4	67.3	68.2	68.0	69.6	69.3	67.4	67.6	67.3	65.9	67.2	66.6	66.7	68.3	67.8	65.3
Geobacter sp. M21	77.9	76.7	75.5	75.0	74.2	87.5	87.0	91.6	91.8	---	79.5	na	na	na	na	na	na	na	na	na	na	na	na	na	na	na	na	na	na	na	na	na	na	na	na	na	na	na	na	na	na
Geobacter sp. M18	81.2	80.1	79.0	77.5	77.9	79.0	78.8	79.2	79.1	79.2	---	na	na	na	na	na	na	na	na	na	na	na	na	na	na	na	na	na	na	na	na	na	na	na	na	na	na	na	na	na	na
G. sulfurreducens KN400	na	na	na	na	na	na	na	na	na	na	na	---	99.2	92.2	92.2	75.7	76.1	74.7	na	na	na	na	na	na	na	na	na	na	na	na	na	na	na	na	na	na	na	na	na	na	na
G. sulfurreducens PCA	na	na	na	na	na	na	na	na	na	na	na	99.2	---	92.3	92.2	75.8	76.1	74.8	na	na	na	na	na	na	na	na	na	na	na	na	na	na	na	na	na	na	na	na	na	na	na
G. soli	na	na	na	na	na	na	na	na	na	na	na	92.2	92.3	---	99.4	76.2	76.5	74.9	na	na	na	na	na	na	na	na	na	na	na	na	na	na	na	na	na	na	na	na	na	na	na
G. anodireducens	na	na	na	na	na	na	na	na	na	na	na	92.3	92.3	99.4	---	76.2	76.4	74.9	na	na	na	na	na	na	na	na	na	na	na	na	na	na	na	na	na	na	na	na	na	na	na
G. pickeringii	na	na	na	na	na	na	na	na	na	na	na	75.8	75.9	76.2	76.3	---	77.8	75.5	na	na	na	na	na	na	na	na	na	na	na	na	na	na	na	na	na	na	na	na	na	na	na
G. metallireducens	70.6	70.0	69.4	70.9	69.9	71.4	71.0	71.5	71.1	71.2	71.3	76.0	76.0	76.5	76.4	77.6	---	80.9	72.1	72.1	71.3	70.7	71.5	69.8	68.7	68.5	68.7	69.0	68.7	70.4	70.0	68.0	67.8	67.8	65.6	67.0	66.7	66.6	68.8	68.2	64.8
L10gmet	na	na	na	na	na	na	na	na	na	na	na	74.7	74.9	75.0	75.0	75.6	81.1	---	71.2	71.2	70.7	69.9	71.2	na	na	na	na	na	na	na	na	na	na	na	na	na	na	na	na	na	na
H2geo	na	na	na	na	na	na	na	na	na	na	na	na	na	na	na	na	72.2	71.1	---	100.0	72.0	70.5	71.5	na	na	na	na	na	na	na	na	na	na	na	na	na	na	na	na	na	na
M4geo	na	na	na	na	na	na	na	na	na	na	na	na	na	na	na	na	72.2	71.1	100.0	---	72.0	70.6	71.6	na	na	na	na	na	na	na	na	na	na	na	na	na	na	na	na	na	na
G. uraniireducens	na	na	na	na	na	na	na	na	na	na	na	na	na	na	na	na	71.4	70.7	71.9	71.9	---	73.8	70.9	na	na	na	na	na	na	na	na	na	na	na	na	na	na	na	na	na	na
G. daltonii	69.0	68.4	67.8	69.1	67.5	69.8	69.3	70.0	69.6	69.8	69.7	69.4	69.5	69.7	69.7	70.2	70.4	69.5	70.3	70.3	73.8	---	69.5	68.8	68.0	67.5	68.0	68.2	68.1	68.6	68.6	67.8	67.3	67.2	65.6	66.5	66.1	66.1	68.2	67.7	63.5
H5geo	na	na	na	na	na	na	na	na	na	na	na	na	na	na	na	na	71.5	71.1	71.5	71.5	70.8	69.5	---	72.0	70.5	70.1	70.3	69.9	69.5	70.1	70.0	68.3	68.3	68.2	na	na	na	na	na	na	na
Geobacter sp. OR1	68.5	67.6	67.1	66.9	67.7	68.7	69.1	68.8	68.8	68.8	69.2	69.5	69.8	69.7	69.7	70.1	69.9	69.6	70.4	70.4	69.9	68.9	72.1	---	69.5	68.3	68.8	68.9	68.7	69.3	69.1	68.0	67.8	67.9	65.3	65.9	65.6	65.6	69.1	68.6	63.2
M1geoD	na	na	na	na	na	na	na	na	na	na	na	na	na	na	na	na	na	na	na	na	na	na	71.6	70.0	---	88.4	88.6	90.0	89.3	72.7	72.4	71.4	71.0	71.3	na	na	na	na	na	na	na
M1geoB	na	na	na	na	na	na	na	na	na	na	na	na	na	na	na	na	na	na	na	na	na	na	71.2	70.1	88.7	---	90.2	93.1	74.8	72.5	72.7	71.1	70.9	71.3	na	na	na	na	na	na	na
M1geoC	na	na	na	na	na	na	na	na	na	na	na	na	na	na	na	na	na	na	na	na	na	na	71.5	71.3	89.4	91.0	---	100.0	87.8	72.1	71.8	70.9	71.4	71.8	na	na	na	na	na	na	na
L1geo	68.3	67.5	67.2	66.6	67.1	68.4	68.2	68.3	68.2	68.4	68.7	68.8	68.8	68.9	68.9	69.2	68.9	68.5	69.7	69.7	69.4	68.5	69.7	68.7	88.2	91.5	98.8	---	96.8	72.1	71.9	70.9	70.8	70.9	64.5	65.7	65.7	65.7	69.9	69.4	63.4
M1geoA	na	na	na	na	na	na	na	na	na	na	na	na	na	na	na	na	na	na	na	na	na	na	69.7	69.0	88.5	72.1	85.6	97.3	---	72.3	72.3	71.4	70.9	71.2	na	na	na	na	na	na	na
P. propionicus	na	na	na	na	na	na	na	na	na	na	na	na	na	na	na	na	na	na	na	na	na	na	70.2	69.6	72.0	70.5	71.0	72.3	72.1	---	95.9	75.3	70.2	70.4	na	na	na	na	na	na	na
L7ppro	na	na	na	na	na	na	na	na	na	na	na	na	na	na	na	na	na	na	na	na	na	na	70.2	69.0	71.5	70.6	70.5	72.2	72.1	96.0	---	75.2	70.3	70.4	na	na	na	na	na	na	na
M3ppro	na	na	na	na	na	na	na	na	na	na	na	na	na	na	na	na	na	na	na	na	na	na	68.5	67.8	70.6	69.5	69.2	71.2	71.3	75.2	75.2	---	69.9	70.0	na	na	na	na	na	na	na
M12geo	na	na	na	na	na	na	na	na	na	na	na	na	na	na	na	na	na	na	na	na	na	na	68.6	68.0	70.8	69.8	70.9	70.9	70.8	70.1	70.3	69.9	---	98.4	na	na	na	na	na	na	na
H7geo	na	na	na	na	na	na	na	na	na	na	na	na	na	na	na	na	na	na	na	na	na	na	68.3	68.1	70.8	70.2	70.7	71.1	70.9	70.3	70.3	70.0	98.7	---	na	na	na	na	na	na	na
Geopsychrobacter electrodiphilus	64.8	64.8	64.4	65.3	63.6	65.1	64.7	65.4	65.4	65.4	65.2	64.9	65.0	64.9	64.9	65.5	65.3	64.8	64.7	64.7	65.1	65.4	65.1	65.3	65.5	65.5	64.7	64.6	64.5	65.1	64.6	64.5	64.9	64.9	---	69.4	66.5	66.6	65.3	65.3	62.2

Supplementary Table 13. Analysis of ANIb and TETRA among the recovered bin-genomes for other dominant microbes in the communities.

* Operational Candidate Species (OCS) is defined by TETRA >0.99 and ANIb >97

TETRA	L2tol	M18tol	L20tolB	*Tolumonas auensis*	M13tol	L20tol	*Tolumonas lignilytica*	L3tol	*Acetobacterium dehalogenans*	*Acetobacterium wieringae*	L13fir	*Acetobacterium bakii*	*Acetobacterium woodii*	L6fir	*Anaeroarcus burkinensis*	M5fir	H6fir	L21met	*Methanocorpusculum labreanum*	L5met	*Methanocorpusculum bavaricum*	M2lac	L12lac	H3lac	H4actB	H4actA	M6act
L2tol	---	1.00	0.98	0.98	0.97	0.96	0.93	0.91	0.85	0.84	0.83	0.79	0.78	0.66	0.57	0.55	0.55	0.51	0.46	0.46	0.44	0.40	0.39	0.39	0.41	0.36	0.36
M18tol	1.00	---	0.98	0.98	0.97	0.97	0.92	0.90	0.85	0.84	0.83	0.79	0.78	0.66	0.56	0.55	0.54	0.49	0.44	0.44	0.43	0.41	0.40	0.39	0.40	0.34	0.34
L20tolB	0.98	0.98	---	0.97	0.97	0.98	0.93	0.90	0.84	0.84	0.84	0.80	0.80	0.64	0.55	0.53	0.53	0.45	0.41	0.39	0.37	0.44	0.43	0.42	0.34	0.27	0.27
Tolumonas auensis	0.98	0.98	0.97	---	0.99	0.98	0.95	0.95	0.83	0.82	0.81	0.78	0.80	0.66	0.53	0.51	0.50	0.48	0.44	0.43	0.42	0.39	0.38	0.38	0.38	0.31	0.31
M13tol	0.97	0.97	0.97	0.99	---	0.99	0.93	0.93	0.81	0.80	0.79	0.76	0.77	0.62	0.49	0.47	0.46	0.45	0.40	0.40	0.39	0.36	0.36	0.35	0.34	0.28	0.28
L20tol	0.96	0.97	0.98	0.98	0.99	---	0.92	0.92	0.81	0.80	0.79	0.76	0.78	0.61	0.48	0.46	0.45	0.42	0.37	0.37	0.36	0.37	0.36	0.36	0.31	0.25	0.25
Tolumonas lignilytica	0.93	0.92	0.93	0.95	0.93	0.92	---	0.97	0.81	0.79	0.79	0.76	0.80	0.67	0.58	0.56	0.55	0.49	0.47	0.43	0.43	0.46	0.45	0.45	0.36	0.26	0.26
L3tol	0.91	0.90	0.90	0.95	0.93	0.92	0.97	---	0.77	0.75	0.75	0.71	0.77	0.63	0.53	0.51	0.50	0.47	0.45	0.42	0.42	0.41	0.39	0.39	0.35	0.26	0.26
Acetobacterium dehalogenans	0.85	0.85	0.84	0.83	0.81	0.81	0.81	0.77	---	0.98	0.98	0.94	0.93	0.77	0.71	0.70	0.69	0.56	0.54	0.50	0.48	0.45	0.43	0.42	0.48	0.38	0.38
Acetobacterium wieringae	0.84	0.84	0.84	0.82	0.80	0.80	0.79	0.75	0.98	---	1.00	0.93	0.91	0.74	0.71	0.70	0.69	0.50	0.47	0.44	0.42	0.43	0.41	0.41	0.42	0.32	0.32
L13fir	0.83	0.83	0.84	0.81	0.79	0.79	0.79	0.75	0.98	1.00	---	0.92	0.91	0.73	0.71	0.70	0.69	0.49	0.46	0.43	0.41	0.44	0.42	0.42	0.40	0.30	0.30
Acetobacterium bakii	0.79	0.79	0.80	0.78	0.76	0.76	0.76	0.71	0.94	0.93	0.92	---	0.92	0.75	0.73	0.72	0.71	0.52	0.49	0.44	0.43	0.46	0.44	0.43	0.34	0.25	0.25
Acetobacterium woodii	0.78	0.78	0.80	0.80	0.77	0.78	0.80	0.77	0.93	0.91	0.91	0.92	---	0.80	0.70	0.68	0.67	0.54	0.51	0.44	0.44	0.53	0.52	0.51	0.36	0.27	0.27
L6fir	0.66	0.66	0.64	0.66	0.62	0.61	0.67	0.63	0.77	0.74	0.73	0.75	0.80	---	0.66	0.65	0.64	0.47	0.46	0.37	0.38	0.59	0.57	0.57	0.37	0.30	0.31
Anaeroarcus burkinensis	0.57	0.56	0.55	0.53	0.49	0.48	0.58	0.53	0.71	0.71	0.71	0.73	0.70	0.66	---	1.00	1.00	0.57	0.60	0.53	0.54	0.45	0.43	0.43	0.47	0.35	0.35
M5fir	0.55	0.55	0.53	0.51	0.47	0.46	0.56	0.51	0.70	0.70	0.70	0.72	0.68	0.65	1.00	---	1.00	0.58	0.61	0.55	0.55	0.45	0.43	0.43	0.50	0.38	0.37
H6fir	0.55	0.54	0.53	0.50	0.46	0.45	0.55	0.50	0.69	0.69	0.69	0.71	0.67	0.64	1.00	1.00	---	0.58	0.61	0.55	0.55	0.45	0.42	0.42	0.49	0.37	0.37
L21met	0.51	0.49	0.45	0.48	0.45	0.42	0.49	0.47	0.56	0.50	0.49	0.52	0.54	0.47	0.57	0.58	0.58	---	0.98	0.97	0.97	0.17	0.15	0.15	0.58	0.48	0.48
Methanocorpusculum labreanum	0.46	0.44	0.41	0.44	0.40	0.37	0.47	0.45	0.54	0.47	0.46	0.49	0.51	0.46	0.60	0.61	0.61	0.98	---	0.96	0.97	0.17	0.15	0.15	0.62	0.52	0.52
L5met	0.46	0.44	0.39	0.43	0.40	0.37	0.43	0.42	0.50	0.44	0.43	0.44	0.44	0.37	0.53	0.55	0.55	0.97	0.96	---	0.99	0.05	0.03	0.03	0.61	0.51	0.51
Methanocorpusculum bavaricum	0.44	0.43	0.37	0.42	0.39	0.36	0.43	0.42	0.48	0.42	0.41	0.43	0.44	0.38	0.54	0.55	0.55	0.97	0.97	0.99	---	0.06	0.04	0.04	0.62	0.51	0.51
M2lac	0.40	0.41	0.44	0.39	0.36	0.37	0.46	0.41	0.45	0.43	0.44	0.46	0.53	0.59	0.45	0.45	0.45	0.17	0.17	0.05	0.06	---	1.00	1.00	0.19	0.12	0.12
L12lac	0.39	0.40	0.43	0.38	0.36	0.36	0.45	0.39	0.43	0.41	0.42	0.44	0.52	0.57	0.43	0.43	0.42	0.15	0.15	0.03	0.04	1.00	---	1.00	0.18	0.11	0.11
H3lac	0.39	0.39	0.42	0.38	0.35	0.36	0.45	0.39	0.42	0.41	0.42	0.43	0.51	0.57	0.43	0.43	0.42	0.15	0.15	0.03	0.04	1.00	1.00	---	0.18	0.11	0.11
H4actB	0.41	0.40	0.34	0.38	0.34	0.31	0.36	0.35	0.48	0.42	0.40	0.34	0.36	0.37	0.47	0.50	0.49	0.58	0.62	0.61	0.62	0.19	0.18	0.18	---	0.94	0.94
H4act	0.36	0.34	0.27	0.31	0.28	0.25	0.26	0.26	0.38	0.32	0.30	0.25	0.27	0.30	0.35	0.38	0.37	0.48	0.52	0.51	0.51	0.12	0.11	0.11	0.94	---	1.00
M6act	0.36	0.34	0.27	0.31	0.28	0.25	0.26	0.26	0.38	0.32	0.30	0.25	0.27	0.31	0.35	0.37	0.37	0.48	0.52	0.51	0.51	0.12	0.11	0.11	0.94	1.00	---

ANIb	L2tol	M18tol	L20tolB	*Tolumonas auensis*	M13tol	L20tol	*Tolumonas lignilytica*	L3tol	*Acetobacterium dehalogenans*	*Acetobacterium wieringae*	L13fir	*Acetobacterium bakii*	*Acetobacterium woodii*	L6fir	*Anaeroarcus burkinensis*	M5fir	H6fir	L21met	*Methanocorpusculum labreanum*	L5met	*Methanocorpusculum bavaricum*	M2lac	L12lac	H3lac	H4actB	H4actA	M6act
L2tol	---	99.1	78.5	83.6	83.3	77.5	79.1	78.5	60.4	60.8	60.3	59.7	60.8	59.1	60.3	60.8	60.6	60.4	59.9	58.8	59.8	60.6	60.2	60.5	64.4	61.5	61.6
M18tol	99.5	---	79.2	82.3	81.6	78.1	77.8	77.5	61.8	61.9	62.3	61.6	62.8	60.3	62.0	61.9	61.9	60.0	62.2	61.1	61.1	61.7	61.4	61.5	61.8	62.7	62.6
L20tolB	80.4	80.6	---	78.8	78.2	76.8	73.4	73.2	62.7	63.0	63.3	61.1	60.1	NaN	58.9	58.2	57.8	NaN	NaN	NaN	NaN	59.3	59.3	59.3	60.9	60.1	60.2
Tolumonas auensis	83.7	81.6	77.6	---	91.9	89.9	79.6	80.6	60.9	63.7	59.9	64.9	64.5	59.5	61.1	63.8	60.9	61.2	61.6	59.3	61.2	61.0	61.0	61.0	61.9	64.2	62.9
M13tol	83.5	81.1	77.1	92.1	---	97.7	79.4	80.4	60.1	60.5	60.4	61.0	60.2	59.7	61.0	60.8	60.6	59.4	58.9	59.0	59.6	61.1	61.0	61.0	59.5	60.9	60.9
L20tol	79.2	78.9	76.8	90.9	98.3	---	76.4	77.4	62.5	62.8	60.6	62.3	60.8	64.0	64.6	63.9	63.5	NaN	57.0	NaN	NaN	63.2	63.2	63.2	NaN	62.9	62.9
Tolumonas lignilytica	79.1	77.3	71.9	79.5	79.3	75.5	---	77.4	61.3	64.2	60.8	63.8	64.5	60.7	60.1	63.0	60.4	59.7	60.8	60.0	61.6	61.8	61.5	61.6	55.5	65.0	63.8
L3tol	78.6	76.9	73.1	80.6	80.4	76.3	77.4	---	60.4	60.9	60.4	60.2	60.5	59.8	60.2	60.2	60.3	59.6	58.4	57.5	57.6	61.5	61.1	61.4	63.0	60.2	59.9
Acetobacterium dehalogenans	60.8	60.9	64.7	61.1	60.9	64.7	61.8	61.0	---	83.2	82.6	73.8	76.6	61.3	62.0	61.7	61.9	59.3	60.2	59.8	59.8	61.0	60.9	60.9	58.8	60.2	60.4
Acetobacterium wieringae	61.2	62.0	62.9	61.4	60.3	62.6	62.2	60.8	83.4	---	93.6	73.4	76.0	60.7	61.9	62.1	61.7	61.5	61.2	60.8	60.6	61.2	61.3	61.2	58.5	62.8	62.4
L13fir	60.6	61.0	63.2	60.3	60.4	62.3	60.4	60.4	82.9	93.7	---	73.3	76.1	59.9	61.8	61.7	61.8	59.9	60.1	59.4	60.2	60.7	60.7	60.7	60.1	60.0	60.1
Acetobacterium bakii	60.5	60.6	63.8	61.0	60.5	63.1	61.0	60.2	73.8	73.4	73.2	---	73.3	60.2	61.4	61.9	61.7	60.3	60.1	60.2	60.1	60.5	60.5	60.6	NaN	62.0	61.5
Acetobacterium woodii	61.9	61.5	58.9	63.8	61.3	59.4	63.9	61.0	76.8	76.0	75.9	73.3	---	61.8	61.8	63.1	61.8	63.2	61.5	61.2	62.0	61.7	61.8	61.7	58.5	66.5	63.8
L6fir	59.9	57.1	NaN	59.2	58.2	NaN	60.1	59.7	61.2	60.9	60.7	60.6	62.0	---	59.0	60.3	59.8	58.7	57.1	56.3	57.2	60.7	60.6	60.8	NaN	60.4	60.1
Anaeroarcus burkinensis	60.4	60.4	57.6	60.4	60.1	61.4	60.0	60.1	61.9	61.9	61.9	61.7	61.8	59.1	---	88.3	88.3	60.2	60.4	61.1	60.2	60.5	60.5	60.4	58.0	60.2	60.2
M5fir	61.2	60.7	60.0	62.0	61.0	65.2	61.2	60.9	62.1	62.4	61.9	62.0	62.4	59.2	88.4	---	98.4	59.8	60.3	60.2	59.7	60.7	60.9	60.8	58.0	61.5	60.8
H6fir	60.5	60.5	60.5	61.0	60.3	61.3	60.0	59.9	62.1	61.7	61.8	61.8	61.7	59.6	88.4	98.4	---	61.0	61.0	60.6	60.5	60.4	60.4	60.4	56.6	60.6	60.5
L21met	62.0	64.9	NaN	63.4	62.2	NaN	61.9	61.0	60.4	60.9	60.6	60.9	60.5	61.5	60.4	61.2	61.2	---	86.4	82.6	83.3	58.1	58.3	58.1	NaN	60.6	60.7
Methanocorpusculum labreanum	61.2	63.5	NaN	60.2	59.8	57.0	59.8	59.9	60.7	60.5	60.3	60.3	60.3	57.0	59.8	60.4	60.0	85.9	---	83.3	83.8	58.8	58.8	58.7	57.4	60.4	60.0
L5met	59.7	62.1	NaN	60.4	59.9	NaN	61.6	59.8	60.8	60.8	60.3	60.5	60.2	58.9	61.3	61.4	61.4	81.8	83.6	---	98.7	62.8	62.7	62.8	NaN	60.8	60.8
Methanocorpusculum bavaricum	62.1	61.7	NaN	60.7	61.1	NaN	60.4	59.5	61.3	61.0	60.5	61.2	60.6	57.9	60.7	60.7	60.6	82.3	83.7	98.7	---	62.0	62.1	62.0	60.8	60.6	60.0
M2lac	61.1	61.2	59.3	60.6	60.5	61.1	60.8	61.1	61.0	60.9	60.8	60.7	61.2	60.9	60.7	60.5	60.5	58.5	59.8	58.9	59.5	---	99.5	99.6	NaN	58.4	58.4
L12lac	61.3	61.5	59.4	61.9	61.0	59.3	61.5	61.7	61.0	61.3	61.0	61.0	61.4	61.2	60.5	60.4	60.4	61.2	60.3	62.6	61.0	99.6	---	99.6	NaN	61.7	61.6
H3lac	60.6	61.4	59.2	61.0	60.4	61.9	60.9	60.5	60.6	61.0	61.0	60.7	61.2	60.5	60.2	60.9	60.8	58.6	60.4	61.3	60.9	99.8	99.6	---	NaN	59.1	59.3
H4actB	60.5	62.7	NaN	59.0	59.2	NaN	59.3	62.1	57.4	60.6	61.7	57.0	58.8	NaN	58.2	58.9	58.2	NaN	NaN	NaN	62.1	NaN	NaN	NaN	---	72.4	87.1
H4act	61.6	62.4	59.6	61.9	60.8	62.7	62.1	60.6	60.1	62.2	60.8	61.6	62.1	56.1	61.1	61.5	61.2	58.5	60.4	59.7	60.1	58.8	58.4	58.5	75.0	---	99.4
M6act	61.5	61.0	59.7	61.8	60.7	59.4	61.7	60.2	59.5	63.1	60.2	63.0	62.4	55.9	60.4	62.0	60.9	58.4	59.1	59.3	60.2	58.1	58.1	58.1	86.3	99.4	---

Supplementary Table S14. Summary of mRNA and DNA read counts and RPKM calculation for SP-H bin-genomes.

Bin-genome ID	Total bases of ORFs	Mapped reads count to ORFs[a]											DNA-RPKM[b]						mRNA-RPKM									
		metagenomic DNA						metatranscriptomic mRNA											before normalization[b]					after normalization[c]				
		Con1	Con2	Con3	Con4	Con5	Total	Con1	Con2	Con3	Con4	Con5	Con1	Con2	Con3	Con4	Con5	**Total**	Con1	Con2	Con3	Con4	Con5	**Con1**	**Con2**	**Con3**	**Con4**	**Con5**
**H1geoA**	2,442,280	1907212	2024868	1642070	7191692	1639202	14405044	164794	139556	102686	65006	104798	77.9	81.2	70.9	89.1	63.3	**80.4**	46.0	46.7	39.0	33.1	80.6	**47.5**	**46.2**	**44.3**	**29.8**	**102.5**
**H1geoB**	1,586,911	884176	973548	556956	3465043	616786	6496509	68141	55099	29853	31913	28089	53.6	58.0	35.7	63.7	35.3	**53.8**	28.2	27.3	16.8	24.1	32.1	**28.3**	**25.4**	**25.4**	**20.3**	**48.9**
**H1geoC**	1,645,543	553662	536042	775996	1849990	680020	4395710	21964	17672	28400	13437	25645	32.2	30.6	47.7	32.7	37.4	**34.9**	8.7	8.4	15.4	9.7	28.1	**9.5**	**9.6**	**11.3**	**10.4**	**26.3**
**H1geoD**	2,848,753	481327	379815	282965	1304439	410132	2858678	20689	13899	10240	10231	15164	16.6	12.9	10.3	13.7	13.4	**13.5**	4.9	3.9	3.3	4.4	9.9	**4.0**	**4.1**	**4.3**	**4.3**	**9.9**
**H2geo**	3,047,821	1025431	888484	1123199	3367952	997408	7402474	70391	38854	37418	17386	37101	33.3	28.3	38.5	33.2	30.6	**32.9**	15.6	10.3	11.3	7.0	22.7	**15.4**	**12.0**	**9.6**	**7.0**	**24.4**
**H3lac**	1,668,484	454182	465034	530435	1599901	704179	3753731	227823	161472	82569	189427	11417	26.7	26.9	33.0	28.6	39.2	**30.2**	91.6	77.8	45.2	138.8	12.7	**103.4**	**87.4**	**41.4**	**146.7**	**9.8**
**H4act**	3,131,599	1014566	1004277	1093265	2442420	866713	6421241	325874	313790	320297	175094	124470	33.3	32.3	37.9	24.3	26.9	**28.8**	73.0	84.3	97.8	71.5	76.9	**63.1**	**75.0**	**74.3**	**84.7**	**82.4**
**H5geo**	3,724,696	637279	721533	536805	2540914	841621	5278152	31712	34047	30587	13675	20286	16.9	18.8	15.1	20.5	21.1	**19.1**	5.7	7.4	7.6	4.5	10.1	**6.5**	**7.5**	**9.6**	**4.2**	**9.2**
**H6fir**	2,786,110	284381	301813	335376	933603	535766	2390939	138168	133160	155904	76214	4637	10.2	10.6	12.7	10.2	18.2	**11.7**	33.9	39.2	52.1	34.1	3.1	**38.9**	**43.2**	**48.1**	**39.3**	**2.0**
**H7geo**	3,185,047	343230	388578	321400	1098554	613104	2764866	38512	27791	23799	11860	18512	11.1	12.3	11.0	10.8	18.7	**12.2**	8.5	7.4	7.2	4.8	11.3	**9.4**	**7.3**	**8.0**	**5.4**	**7.3**
**H8spi**	2,993,938	253760	261249	220276	610209	248306	1593800	47502	29935	26308	22288	17909	8.9	9.0	8.1	6.5	8.2	**7.6**	11.3	8.6	8.5	9.7	11.8	**9.7**	**7.3**	**8.0**	**11.4**	**10.9**
**H9bac**	2,823,157	124964	119244	118466	270477	123640	756791	20073	16476	13416	6902	8230	4.3	4.0	4.3	2.8	4.0	**3.5**	4.7	4.6	4.3	3.0	5.3	**3.9**	**4.1**	**3.5**	**3.7**	**4.7**
**H10eps**	2,555,140	89938	94975	87145	177099	83469	532626	11070	10457	6458	4681	2582	3.7	3.9	3.8	2.2	3.3	**3.0**	3.1	3.5	2.5	2.4	2.0	**2.5**	**2.8**	**2.0**	**3.3**	**1.9**
**H11bac**	3,645,112	93180	86130	69667	154706	78533	482216	12070	9771	6753	4140	5595	2.5	2.2	1.9	1.2	2.0	**1.7**	2.2	2.1	1.7	1.4	2.8	**1.5**	**1.6**	**1.5**	**1.9**	**2.5**
**H12unc**	4,079,749	108985	118763	90340	262245	146308	726641	1905	1321	1015	1594	1539	2.7	2.9	2.4	2.0	3.4	**2.5**	0.3	0.3	0.2	0.5	0.7	**0.3**	**0.2**	**0.2**	**0.6**	**0.5**
H3lacP	243,715	39760	41975	51670	146355	59874	339634	5901	6477	6998	6555	875	15.8	16.4	21.7	17.7	22.5	**18.5**	16.1	21.1	25.9	32.5	6.6	**18.7**	**23.8**	**22.1**	**34.0**	**5.4**
H4actB	876,568	114039	128624	137961	310115	122418	813157	11264	12291	12727	8507	10266	12.5	13.8	16.0	10.3	12.7	**12.2**	8.4	11.0	13.0	11.6	21.2	**8.2**	**9.7**	**9.9**	**13.7**	**20.4**
H6firP	126,010	13454	13624	15137	42228	22666	107109	384	365	390	198	15	10.5	10.4	12.5	10.0	16.7	**11.4**	2.0	2.3	2.8	1.9	0.2	**2.2**	**2.6**	**2.6**	**2.2**	**0.2**
H6firP2	45,007	4802	4993	5737	15116	8271	38919	150	143	173	99	5	10.3	10.6	13.0	9.9	16.8	**11.4**	2.2	2.5	3.5	2.7	0.2	**2.4**	**2.7**	**3.0**	**3.1**	**0.1**
H7geoP	466,315	41294	47480	37692	110428	65723	302617	6463	6246	5308	3742	5253	8.8	10.0	8.5	7.2	13.3	**8.8**	9.4	10.9	10.6	10.0	21.2	**9.5**	**9.7**	**11.0**	**12.3**	**14.1**
Reads to metagenome		8914854	9073242	8433708	29360208	9430577	65212589	1304911	1088423	957349	715456	472917			SUM of RPKMs			**492**	414	409	385	438	386	**422**	**411**	**364**	**469**	**415**
Reads to bin-genomes		8469622	8601049	8032558	27893486	8864139	61860854	1224850	1028822	901299	662949	442388																
% in bin-genome		95	95	95	95	94	95	94	95	94	93	94																

[a] Raw reads for each condition of both metagenomic DNA and metatranscriptomic mRNA were separately mapped to SP-H ORFs by using similarity cut-off by 0.95 and length cut-off by 0.7.

[b] RPKM values, Reads Per Kilobase per Million mapped reads, for both DNA and mRNA samples under five different operational conditions were calculated based on total bases of ORFs in each bin-genome.

[c] mRNA-RPKM values were normalized by DNA existence fluctuation among five conditions based on DNA-RPKM of each condition divided by DNA-RPKM of total reads.

Supplementary Table S15. Summary of mRNA and DNA read counts and RPKM calculation for SP-M bin-genomes.

Bin-genome ID	Total bases of ORFs	Mapped reads count to ORFs[a]: metagenomic DNA						Mapped reads count to ORFs[a]: metatranscriptomic mRNA					DNA-RPKM[b]						mRNA-RPKM: before normalization[b]					mRNA-RPKM: after normalization[c]				
		Con1	Con2	Con3	Con4	Con5	Total	Con1	Con2	Con3	Con4	Con5	Con1	Con2	Con3	Con4	Con5	Total	Con1	Con2	Con3	Con4	Con5	Con1	Con2	Con3	Con4	Con5
**M1geoA**	2,715,252	3322331	3249832	3433435	3747513	3398025	17151136	121133	179745	109485	48115	71117	127.3	124.0	119.9	119.7	110.9	**120.0**	71.2	76.6	51.5	26.2	138.2	**67.1**	**74.1**	**51.6**	**26.3**	**149.6**
**M1geoB**	1,427,715	1390908	1092502	1320923	1423476	1488629	6716438	24906	27305	20553	9384	17622	98.1	76.7	84.9	83.7	89.5	**86.5**	27.0	21.4	17.8	9.4	63.0	**23.8**	**24.2**	**18.1**	**9.7**	**61.0**
**M1geoC**	1,205,514	390824	650479	549870	578586	348191	2517950	8501	20830	9956	5526	4463	32.5	53.8	41.6	40.1	24.6	**38.2**	10.8	19.2	10.2	6.5	18.8	**12.7**	**13.7**	**9.3**	**6.2**	**29.1**
**M1geoD**	1,892,772	278829	542255	278367	406400	135862	1641713	7040	28778	7865	4336	1907	14.8	28.7	13.5	18.0	6.2	**15.9**	5.7	17.0	5.1	3.3	5.1	**6.2**	**9.4**	**6.1**	**2.9**	**13.3**
**M2lac**	1,947,600	385823	568063	511706	825796	577942	2869330	142572	208897	142428	274754	5049	19.9	29.1	24.0	35.5	25.4	**27.0**	112.7	119.6	90.1	201.4	13.2	**153.0**	**110.9**	**101.3**	**153.3**	**14.0**
**M3ppro**	4,287,699	586786	456676	645577	837004	1080753	3606796	60258	75895	107307	84617	9418	13.8	10.7	13.8	16.4	21.7	**15.5**	21.8	19.9	31.0	28.4	11.2	**24.4**	**28.8**	**34.7**	**26.8**	**8.0**
**M4geo**	3,049,983	381124	329298	425948	514888	333432	1984690	5538	7418	6674	4386	3285	12.7	10.9	12.9	14.3	9.4	**12.1**	2.8	2.7	2.7	2.1	5.5	**2.7**	**3.0**	**2.5**	**1.8**	**7.1**
**M5fir**	3,075,351	202228	216429	265950	340242	369662	1394511	53756	86215	97202	43139	1855	6.7	7.2	8.0	9.4	10.5	**8.5**	27.4	31.8	39.7	20.4	3.1	**34.5**	**37.6**	**41.7**	**18.3**	**2.5**
**M6act**	3,029,682	301683	374320	333095	251129	295106	1555333	20126	46662	54165	13315	10571	10.5	13.0	10.6	7.3	8.7	**9.9**	10.7	18.1	23.2	6.6	18.7	**10.1**	**13.8**	**21.7**	**9.0**	**21.1**
**M7geo**	3,968,730	125093	140364	205895	57601	400432	929385	2086	3423	2345	334	4961	3.0	3.3	4.5	1.2	8.2	**4.1**	0.8	0.9	0.7	0.1	6.0	**1.0**	**1.1**	**0.6**	**0.4**	**3.0**
**M8spi**	2,981,820	165096	149203	178914	163814	157371	814398	11029	13249	9332	5695	3783	5.9	5.3	5.9	4.9	4.8	**5.4**	6.1	5.3	4.1	2.9	6.9	**5.5**	**5.3**	**3.8**	**3.2**	**7.7**
**M9unc**	2,992,662	86489	116869	177905	34010	174087	589360	371	491	419	112	310	3.1	4.1	5.7	1.0	5.2	**3.8**	0.2	0.2	0.2	0.1	0.6	**0.3**	**0.2**	**0.1**	**0.2**	**0.4**
**M10bac**	3,188,667	104178	83289	94311	154711	150431	586920	4411	10480	7045	8242	8351	3.4	2.7	2.8	4.2	4.1	**3.5**	2.2	3.8	2.8	3.8	13.7	**2.3**	**4.9**	**3.5**	**3.2**	**11.5**
**M11eps**	2,622,807	62662	68197	69221	65691	93275	359046	2870	6244	4591	5258	1151	2.6	2.8	2.6	2.3	3.3	**2.7**	1.8	2.9	2.3	3.1	2.4	**1.9**	**2.8**	**2.4**	**3.7**	**2.0**
**M12geo**	3,595,401	113241	46924	109302	115947	192108	577522	3596	3452	4446	2839	1665	3.5	1.4	3.1	3.0	5.1	**3.3**	1.7	1.2	1.7	1.3	2.6	**1.6**	**2.7**	**1.8**	**1.4**	**1.7**
**M13tol**	2,678,925	110469	15762	143001	215874	132284	617390	65699	8269	67521	57344	5215	4.3	0.6	5.1	7.0	4.4	**4.4**	39.3	3.6	32.3	31.8	10.3	**40.1**	**25.7**	**28.0**	**19.9**	**10.3**
**M14spi**	2,831,517	104169	73087	96664	84788	60673	419381	1561	1910	2128	1441	1048	3.9	2.7	3.3	2.6	1.9	**2.8**	0.9	0.8	1.0	0.8	2.0	**0.7**	**0.8**	**0.8**	**0.8**	**2.9**
M15fir	3,849,516	76982	87761	84601	70247	64893	384484	5941	14740	11237	6383	2865	2.0	2.3	2.0	1.5	1.4	**1.8**	2.4	4.3	3.6	2.4	3.8	**2.2**	**3.4**	**3.3**	**2.8**	**4.8**
M17geo	3,876,561	68108	44891	83564	30065	101087	327715	612	813	657	117	902	1.7	1.1	1.9	0.6	2.2	**1.5**	0.2	0.2	0.2	0.0	1.2	**0.2**	**0.3**	**0.2**	**0.1**	**0.8**
M18tol	1,822,401	11260	3362	14238	19395	38024	86279	1947	690	3307	1848	768	0.6	0.2	0.7	0.9	1.8	**0.9**	1.6	0.4	2.2	1.4	2.1	**2.3**	**2.0**	**2.7**	**1.4**	**1.0**
M19bac	3,322,566	33905	26038	35872	46456	37422	179693	1599	2437	2460	3040	1782	1.0	0.8	1.0	1.2	1.0	**1.0**	0.7	0.8	0.9	1.3	2.7	**0.7**	**1.0**	**0.9**	**1.1**	**2.8**
M20fir	761,991	10171	3790	6895	12010	5544	38410	847	638	1332	1141	236	1.4	0.5	0.9	1.4	0.6	**1.0**	1.8	1.0	2.2	2.2	1.6	**1.2**	**1.8**	**2.5**	**1.5**	**2.4**
M21unc	2,390,274	18787	14052	31676	23037	31707	119259	131	139	208	291	205	0.8	0.6	1.2	0.8	1.2	**0.9**	0.1	0.1	0.1	0.2	0.4	**0.1**	**0.1**	**0.1**	**0.2**	**0.4**
M5firP	56,427	4590	5073	6115	7412	7969	31159	24	54	47	15	4	8.7	9.6	10.6	11.7	12.9	**10.8**	0.7	1.1	1.1	0.4	0.4	**0.9**	**1.3**	**1.1**	**0.4**	**0.3**
M16actB	1,150,047	42084	52364	48881	35797	42096	221222	1016	3005	3890	940	748	3.6	4.5	3.8	2.6	3.1	**3.5**	1.3	2.9	4.1	1.1	3.3	**1.3**	**2.2**	**3.7**	**1.6**	**3.7**
Reads to metagenome		8686268	8727062	9535085	10423552	10199776	47571743	566246	781360	707397	610414	171319					SUM of RPKMs	**385**	352	356	331	357	337	**397**	**371**	**342**	**296**	**361**
Reads to bin-genomes		8377820	8410880	9151926	10061889	9717005	45719520	547570	751779	676600	582612	159281																
% in bin-genome		96	96	96	97	95	96	97	96	96	95	93																

[a] Raw reads for each condition of both metagenomic DNA and metatranscriptomic mRNA were separately mapped to SP-H ORFs by using similarity cut-off by 0.95 and length cut-off by 0.7.

[b] RPKM values, Reads Per Kilobase per Million mapped reads, for both DNA and mRNA samples under five different operational conditions were calculated based on total bases of ORFs in each bin-genome.

[c] mRNA-RPKM values were normalized by DNA existence fluctuation among five conditions based on DNA-RPKM of each condition divided by DNA-RPKM of total reads.

Supplementary Table S16. Summary of mRNA and DNA read counts and RPKM calculation for SP-L bin-genomes.

Bin-genome ID	Total bases of ORFs	Mapped reads count to ORFs[a]											DNA-RPKM[b]						mRNA-RPKM									
		metagenomic DNA						metatranscriptomic mRNA											before normalization[b]					after normalization[c]				
		Con1	Con2	Con3	Con4	Con5	Total	Con1	Con2	Con3	Con4	Con5	Con1	Con2	Con3	Con4	Con5	**Total**	Con1	Con2	Con3	Con4	Con5	**Con1**	**Con2**	**Con3**	**Con4**	**Con5**
**L1geo**	3,966,585	4934372	3184858	1897243	2011668	2090780	14118921	51147	24408	11029	5083	20852	113.2	70.7	42.9	49.6	53.6	**66.5**	17.8	13.5	6.2	3.2	28.2	**7.5**	**12.6**	**11.2**	**4.9**	**41.3**
**L2tol**	3,088,263	1755692	3011278	3449156	2683542	2228128	13127796	322728	228650	190504	153323	37304	52.4	87.0	101.4	86.1	74.3	**80.4**	146.1	165.1	138.7	124.7	65.6	**160.6**	**151.5**	**128.2**	**133.1**	**83.9**
**L3tol**	3,153,606	573206	583585	902873	826599	672300	3558563	128006	67463	81976	76414	4025	16.8	16.5	26.0	26.0	22.0	**21.4**	56.9	47.8	58.5	61.0	6.9	**51.9**	**61.3**	**56.0**	**57.2**	**8.0**
**L4bac**	3,096,891	244852	393796	484550	497611	439323	2060132	6804	6221	7782	7369	5539	7.3	11.4	14.3	16.0	14.7	**12.7**	3.1	4.5	5.7	6.0	9.8	**3.8**	**5.0**	**5.9**	**5.4**	**10.0**
**L5met**	1,306,779	89188	173349	260446	126398	83750	733131	33317	22263	36820	18431	8889	6.2	11.7	17.8	9.4	6.5	**10.4**	35.1	37.4	62.4	34.9	36.4	**42.4**	**33.3**	**42.6**	**44.1**	**69.1**
**L6fir**	1,042,185	144370	82815	112191	124563	163396	627335	6839	3714	6208	2917	4769	13.1	7.3	10.0	12.2	16.6	**11.7**	9.4	8.2	13.8	7.2	25.5	**6.0**	**13.0**	**18.7**	**7.9**	**21.3**
**L7ppro**	3,352,092	126131	332562	377905	389801	391044	1617443	10729	7973	12256	14238	10410	3.4	8.6	9.9	11.1	11.6	**8.8**	4.3	5.1	8.0	10.3	16.3	**8.2**	**5.3**	**8.3**	**9.3**	**14.7**
**L8spi**	2,677,965	244539	235019	240331	166696	152858	1039443	7360	1376	1050	2871	4343	8.7	8.1	8.5	6.4	6.1	**7.6**	4.0	1.2	0.9	2.8	9.2	**2.5**	**1.1**	**1.0**	**3.8**	**13.5**
**L9bac**	3,521,796	267575	295463	317282	210956	193814	1285090	4942	2866	3204	2338	4614	6.9	7.3	8.0	5.8	5.6	**6.8**	1.9	1.8	2.0	1.6	7.0	**1.4**	**1.6**	**2.0**	**2.2**	**10.0**
**L10gmet**	3,170,025	65656	349130	249706	124671	43982	833145	359	978	567	404	127	1.9	9.8	7.1	3.9	1.4	**5.0**	0.2	0.7	0.4	0.3	0.2	**0.3**	**0.3**	**0.3**	**0.5**	**0.9**
**L11unc**	4,100,073	223400	338528	315655	293063	232706	1403352	484	191	248	595	726	5.0	7.4	7.0	7.1	5.9	**6.5**	0.2	0.1	0.1	0.4	1.0	**0.2**	**0.1**	**0.1**	**0.4**	**1.3**
**L12lac**	1,700,727	26638	46477	67082	240122	287960	668279	7207	7256	8846	25950	1221	1.4	2.3	3.5	13.5	16.8	**7.2**	5.7	9.2	11.3	36.9	3.8	**21.1**	**27.8**	**27.3**	**22.4**	**1.9**
**L13fir**	3,061,467	58587	89917	97884	133815	247257	627460	8928	3782	5076	11176	24565	1.7	2.6	2.9	4.3	8.2	**3.8**	4.0	2.7	3.7	9.0	42.9	**6.3**	**4.0**	**5.7**	**9.2**	**23.6**
**L14bac**	3,572,166	82801	115863	141763	150549	191289	682265	2422	1521	1957	1942	2543	2.2	2.9	3.6	4.2	5.6	**3.6**	1.0	1.0	1.2	1.4	3.9	**1.2**	**1.2**	**1.5**	**1.4**	**3.0**
**L15bac**	2,817,237	51598	50751	53247	126997	116983	399576	1395	1232	1404	2583	1565	1.7	1.6	1.7	4.4	4.3	**2.7**	0.7	1.0	1.1	2.3	3.0	**0.8**	**1.6**	**2.0**	**1.6**	**2.2**
**L22fir**	2,151,693	53742	59901	75428	111077	154201	454349	6463	3237	4227	4318	2311	2.3	2.5	3.2	5.1	7.4	**4.0**	4.2	3.3	4.4	5.0	5.8	**5.2**	**5.3**	**6.4**	**4.5**	**3.7**
L16bac	3,438,726	35580	65456	76963	73117	55873	306989	1057	944	1385	1539	1662	0.9	1.6	2.0	2.0	1.6	**1.6**	0.4	0.6	0.9	1.1	2.5	**0.5**	**0.6**	**0.8**	**1.0**	**3.0**
L17bac	3,426,279	63259	22797	37483	63594	147836	334969	826	294	451	1060	1905	1.7	0.6	1.0	1.8	4.4	**1.8**	0.3	0.2	0.3	0.8	3.0	**0.3**	**0.6**	**0.6**	**0.9**	**1.5**
L18ver	5,758,203	141867	107469	164518	79496	145061	638411	5777	1493	1779	926	1285	2.2	1.6	2.5	1.3	2.5	**2.0**	1.4	0.6	0.7	0.4	1.2	**0.9**	**0.7**	**0.6**	**0.7**	**1.1**
L19bac	3,062,352	23772	42566	41549	37520	31434	176841	248	197	281	302	327	0.7	1.3	1.2	1.2	1.1	**1.1**	0.1	0.1	0.2	0.3	0.6	**0.1**	**0.1**	**0.2**	**0.3**	**0.7**
L20tol	1,166,886	42711	19450	19810	13994	39790	135755	3931	878	828	308	309	2.8	1.2	1.3	1.0	2.9	**1.8**	3.9	1.4	1.3	0.6	1.2	**1.8**	**2.1**	**2.2**	**1.2**	**0.9**
L20tolB	823,299	23154	8234	8877	10354	22554	73173	1720	419	307	248	221	2.2	0.8	0.8	1.1	2.4	**1.4**	2.5	1.0	0.7	0.6	1.2	**1.2**	**1.8**	**1.4**	**1.0**	**0.9**
L21met	894,954	14477	25501	37603	16935	12150	106666	2641	1890	3018	1358	766	1.5	2.5	3.8	1.8	1.4	**2.2**	4.1	4.6	7.5	3.8	4.6	**4.4**	**4.1**	**5.1**	**5.2**	**8.7**
L23elu	1,276,272	26948	13654	17827	46473	40568	145470	853	344	546	1453	1055	2.0	1.0	1.3	3.6	3.3	**2.2**	0.9	0.6	1.0	2.9	4.5	**0.7**	**1.4**	**1.9**	**2.0**	**3.5**
L24fir	1,631,334	24513	14786	19437	13550	21784	94070	785	141	175	450	739	1.4	0.8	1.1	0.8	1.4	**1.1**	0.7	0.2	0.2	0.7	2.5	**0.4**	**0.3**	**0.3**	**1.1**	**2.3**
L12lacP	212,073	1264	2735	3237	11752	11851	30839	108	116	155	420	21	0.5	1.1	1.3	5.2	5.4	**2.6**	0.7	1.1	1.5	4.7	0.5	**2.4**	**2.7**	**3.6**	**2.7**	**0.3**
Reads to metagenome		9845047	10171382	9997513	9161502	8808817	47984261	649020	407156	403655	361385	167151					SUM of RPKMs	**277**	310	313	333	323	287	**332**	**339**	**334**	**324**	**331**
Reads to bin-genomes		9339892	9665940	9470046	8584913	8218672	45279463	617076	389847	382079	338016	142093																
% in bin-genome		95	95	95	94	93	94	95	96	95	94	85																

[a] Raw reads for each condition of both metagenomic DNA and metatranscriptomic mRNA were separately mapped to SP-H ORFs by using similarity cut-off by 0.95 and length cut-off by 0.7.

[b] RPKM values, Reads Per Kilobase per Million mapped reads, for both DNA and mRNA samples under five different operational conditions were calculated based on total bases of ORFs in each bin-genome.

[c] mRNA-RPKM values were normalized by DNA existence fluctuation among five conditions based on DNA-RPKM of each condition divided by DNA-RPKM of total reads.

Supplementary Table S17. Top 30 highly down regulated CDSs responded to AcPro stimulus for fermentation-associated OCSs.

Bin-genom ID	Contig ID	ORF begin	ORF end		Expression dynamics (SP/AcPro)	KO	KO description [Yellow highlight: Fermentation-related metabolic genes] [Blue letter: Ribosomal proteins]	Gene expression level ($mRNA_{RPKM}$) Con1 SP	Con2 SP+	Con3 OC_{short}	Con4 OC_{long}	Con5 AcPro	Ave DNA_{RPKM}
**OCS L2tol**													
**L2tol**	L_contig_8	231584	232117	-	**56.6**	K01507	ppa; inorganic pyrophosphatase [EC:3.6.1.1]	812	798	360	730	14	103
**L2tol**	L_contig_6	279736	280968	-	**53.5**	K00058	serA, PHGDH; D-3-phosphoglycerate dehydrogenase	665	479	280	400	12	76
**L2tol**	L_contig_6	262278	263405	+	**49.2**	K10112	msmX, msmK, malK, sugC, ggtA, msiK; multiple sugar transport system ATP-binding protein	1001	650	538	688	20	74
**L2tol**	L_contig_6	258847	260112	+	**41.8**	K02027	ABC.MS.S; multiple sugar transport system substrate-binding protein	2780	1931	1686	2022	67	76
**L2tol**	L_contig_100	21403	22731	-	**41.5**	K01940	argG; argininosuccinate synthase [EC:6.3.4.5]	478	287	190	251	12	73
**L2tol**	L_contig_12	257148	257378	-	**40.6**	K02963	RP-S18, MRPS18, rpsR; small subunit ribosomal protein S18	4039	2645	1537	3388	99	89
**L2tol**	L_contig_6	260176	261294	+	**40.4**	K02025	ABC.MS.P; multiple sugar transport system permease protein	1105	683	567	786	27	79
**L2tol**	L_contig_10	99521	99913	+	**39.9**	K02994	RP-S8, rpsH; small subunit ribosomal protein S8	6213	3167	2395	4358	156	130
**L2tol**	L_contig_100	2237	3427	+	**38.6**	K00813	aspC; aspartate aminotransferase [EC:2.6.1.1]	496	547	313	305	13	76
**L2tol**	L_contig_12	257405	257782	-	**38.3**	K02990	RP-S6, MRPS6, rpsF; small subunit ribosomal protein S6	2325	1127	848	1773	61	95
**L2tol**	L_contig_13	244524	246005	-	**37.7**	K01212	sacC; levanase [EC:3.2.1.65]	390	262	238	253	10	80
**L2tol**	L_contig_443	14817	15455	-	**37.6**	K10914	crp; CRP/FNR family transcriptional regulator, cyclic AMP receptor protein	451	378	240	416	12	98
**L2tol**	L_contig_8	123529	124977	-	**37.5**	K00703	glgA; starch synthase [EC:2.4.1.21]	396	352	199	324	11	102
**L2tol**	L_contig_12	256658	257107	-	**36.9**	K02939	RP-L9, MRPL9, rplI; large subunit ribosomal protein L9	1885	1203	595	1299	51	91
**L2tol**	L_contig_6	263426	265357	+	**35.9**	K05343	treS; maltose alpha-D-glucosyltransferase/ alpha-amylase	1139	636	492	741	32	78
**L2tol**	L_contig_33	175500	176975	+	**34.9**	K00873	PK, pyk; pyruvate kinase [EC:2.7.1.40]	2354	1383	832	1695	67	106
**L2tol**	L_contig_10	99193	99498	+	**33.3**	K02954	RP-S14, MRPS14, rpsN; small subunit ribosomal protein S14	6674	3375	2440	4487	200	124
**L2tol**	L_contig_147	87879	89093	+	**33.1**	K00647	fabB; 3-oxoacyl-[acyl-carrier-protein] synthase I [EC:2.3.1.41]	626	343	254	447	19	76
**L2tol**	L_contig_13	226253	226813	-	**32.7**	-		447	394	278	321	14	84
**L2tol**	L_contig_34	173623	174549	-	**32.5**	K00847	scrK; fructokinase [EC:2.7.1.4]	537	399	311	411	17	79
**L2tol**	L_contig_1438	949	1452	-	**30.7**	K02864	RP-L10, MRPL10, rplJ; large subunit ribosomal protein L10	7929	3297	2890	5349	258	131
**L2tol**	L_contig_8	42332	43327	-	**30.7**	K00134	GAPDH, gapA; glyceraldehyde 3-phosphate dehydrogenase	10143	6667	3957	7112	331	106
**L2tol**	L_contig_13	110858	111196	-	**29.2**	K03210	yajC; preprotein translocase subunit YajC	659	472	318	418	23	81
**L2tol**	L_contig_10	90123	91034	+	**29.0**	K00611	OTC, argF, argI; ornithine carbamoyltransferase [EC:2.1.3.3]	486	410	229	311	17	100
**L2tol**	L_contig_34	53736	54860	+	**28.9**	K00133	asd; aspartate-semialdehyde dehydrogenase [EC:1.2.1.11]	590	369	208	304	20	91
**L2tol**	L_contig_147	47932	48543	-	**28.8**	K11755	hisIE; phosphoribosyl-ATP pyrophosphohydrolase / phosphoribosyl-AMP cyclohydrolase	360	346	195	217	13	71
**L2tol**	L_contig_33	52759	54270	+	**27.2**	-		276	301	158	176	10	92
**L2tol**	L_contig_34	22102	22728	+	**27.2**	K03775	slyD; FKBP-type peptidyl-prolyl cis-trans isomerase SlyD [EC:5.2.1.8]	332	176	124	297	12	86
**L2tol**	L_contig_33	20661	22076	+	**26.9**	K01193	sacA; beta-fructofuranosidase [EC:3.2.1.26]	727	640	429	505	27	85
**L2tol**	L_contig_6	30191	30736	+	**26.8**	K03607	proQ; ProP effector	375	285	164	184	14	82
**L2tol**	L_contig_12	286878	287348	-	**25.9**	K00794	ribH, RIB4; 6,7-dimethyl-8-ribityllumazine synthase [EC:2.5.1.78]	421	407	214	288	16	88
**L2tol**	L_contig_6	195325	197325	+	**25.7**	K00615	tktA, tktB; transketolase [EC:2.2.1.1]	394	290	176	286	15	76
**OCS L3tol**													
**L3tol**	L_contig_427	111	467	-	**128.6**	K02952	RP-S13, rpsM; small subunit ribosomal protein S13	2477	1652	1455	2708	19	29
**L3tol**	L_contig_427	2090	2524	-	**126.3**	K02876	RP-L15, MRPL15, rplO; large subunit ribosomal protein L15	1997	1334	1254	2103	16	26
**L3tol**	L_contig_228	15732	16103	+	**116.5**	K02888	RP-L21, MRPL21, rplU; large subunit ribosomal protein L21	2154	1560	975	2173	18	39
**L3tol**	L_contig_310	3963	4337	-	**96.9**	K02950	RP-S12, MRPS12, rpsL; small subunit ribosomal protein S12	1777	908	1006	1823	18	33
**L3tol**	L_contig_101	2	649	-	**74.2**	K00873	PK, pyk; pyruvate kinase [EC:2.7.1.40]	788	613	417	658	11	27
**L3tol**	L_contig_9	10029	10259	-	**63.8**	K02963	RP-S18, MRPS18, rpsR; small subunit ribosomal protein S18	1899	1133	1078	1924	30	27
**L3tol**	L_contig_400	640	1026	+	**49.5**	K02879	RP-L17, MRPL17, rplQ; large subunit ribosomal protein L17	879	611	815	846	18	30
**L3tol**	L_contig_337	12708	14018	-	**47.7**	K03545	tig; trigger factor	501	356	255	500	10	23
**L3tol**	L_contig_427	2718	3218	-	**45.0**	K02988	RP-S5, MRPS5, rpsE; small subunit ribosomal protein S5	1855	1076	1108	2184	41	27
**L3tol**	L_contig_192	33762	34055	-	**41.9**	K04078	groES, HSPE1; chaperonin GroES	980	493	573	822	23	29
**L3tol**	L_contig_310	1225	3327	-	**38.9**	K02355	fusA, GFM, EFG; elongation factor G	1400	751	691	1070	36	29
**L3tol**	L_contig_123	12259	13302	+	**37.8**	-		1993	2468	3441	2235	53	24
**L3tol**	L_contig_427	2528	2710	-	**37.2**	K02907	RP-L30, MRPL30, rpmD; large subunit ribosomal protein L30	1398	827	855	1818	38	22
**L3tol**	L_contig_123	1	369	+	**36.4**	K00873	PK, pyk; pyruvate kinase [EC:2.7.1.40]	678	684	411	739	19	27
**L3tol**	L_contig_2	305808	306035	-	**34.1**	K02110	ATPF0C, atpE; F-type H+-transporting ATPase subunit c	1030	1231	926	1014	30	30
**L3tol**	L_contig_41	88000	88887	-	**32.5**	K02357	tsf, TSFM; elongation factor Ts	1007	629	539	1051	31	25
**L3tol**	L_contig_301	119665	120930	-	**32.1**	K02027	ABC.MS.S; multiple sugar transport system substrate-binding protein	873	1303	1441	1074	27	20
**L3tol**	L_contig_2	306079	306858	-	**31.8**	K02108	ATPF0A, atpB; F-type H+-transporting ATPase subunit a	561	627	529	493	18	31
**L3tol**	L_contig_123	186786	188195	+	**31.3**	K01915	glnA; glutamine synthetase [EC:6.3.1.2]	1223	1145	991	1148	39	30
**L3tol**	L_contig_427	751	2082	-	**30.1**	K03076	secY; preprotein translocase subunit SecY	1554	912	1001	1666	52	30
**L3tol**	L_contig_81	22973	24343	+	**29.8**	K02809	PTS-Scr-EIIB, scrA; PTS system, sucrose-specific IIB component	896	929	1136	1118	30	22
**L3tol**	L_contig_9	211050	212045	+	**27.2**	K00134	GAPDH, gapA; glyceraldehyde 3-phosphate dehydrogenase	3574	3217	2381	3765	131	30
**L3tol**	L_contig_310	3	1160	-	**26.8**	K02358	tuf, TUFM; elongation factor Tu	2710	2084	2027	2955	101	56
**L3tol**	L_contig_400	3	596	+	**26.4**	K03040	rpoA; DNA-directed RNA polymerase subunit alpha [EC:2.7.7.6]	1529	940	914	1540	58	27
**L3tol**	L_contig_2	56400	57071	-	**26.0**	-		266	375	459	336	10	31
**L3tol**	L_contig_310	3409	3879	-	**25.3**	K02992	RP-S7, MRPS7, rpsG; small subunit ribosomal protein S7	1851	884	901	1738	73	29
**L3tol**	L_contig_427	3233	3484	-	**25.1**	K02881	RP-L18, MRPL18, rplR; large subunit ribosomal protein L18	1373	864	894	1299	55	27
**L3tol**	L_contig_29	16569	18236	+	**24.9**	K01953	asnB, ASNS; asparagine synthase (glutamine-hydrolysing)	309	265	185	274	12	26
**L3tol**	L_contig_406	37451	38017	+	**24.5**	K02945	RP-S1, rpsA; small subunit ribosomal protein S1	1188	801	736	1242	49	17
**L3tol**	L_contig_107	128359	129273	-	**24.3**	K02406	fliC; flagellin	549	793	863	523	23	22

Supplementary Table S17. *Continued*

Bin-genom ID	Contig ID	ORF begin	ORF end		Expression dynamics (SP/AcPro)	KO	KO description [Yellow highlight: Fermentation-related metabolic genes] [Blue letter: Ribosomal proteins]	Gene expression level ($mRNA_{RPKM}$) Con1 SP	Con2 SP+	Con3 OC_{short}	Con4 OC_{long}	Con5 AcPro	Ave DNA_{RPKM}
**OCS M13tol**													
**M13tol**	M_contig_104	69476	70363	-	**40.0**	K02357	tsf, TSFM; elongation factor Ts	526	238	138	206	13	5
**M13tol**	M_contig_1871	17267	17449	+	**32.1**	K02907	RP-L30, MRPL30, rpmD; large subunit ribosomal protein L30	1024	201	341	494	32	5
**M13tol**	M_contig_630	1427	1774	+	**30.5**	K02884	RP-L19, MRPL19, rplS; large subunit ribosomal protein L19	513	79	95	239	17	5
**M13tol**	M_contig_178	123735	124472	-	**25.9**	K01803	TPI, tpiA; triosephosphate isomerase (TIM) [EC:5.3.1.1]	410	112	99	181	16	4
**M13tol**	M_contig_1379	10062	10298	+	**25.6**	K02078	acpP; acyl carrier protein	631	310	217	269	25	5
**M13tol**	M_contig_1843	2837	3211	-	**24.4**	K02950	RP-S12, MRPS12, rpsL; small subunit ribosomal protein S12	759	172	173	394	31	5
**M13tol**	M_contig_71	79566	81284	+	**23.8**	K08483	PTS-EI.PTSI, ptsI; phosphotransferase system, enzyme I, PtsI	404	112	115	142	17	6
**M13tol**	M_contig_420	121563	122975	-	**22.5**	K00873	PK, pyk; pyruvate kinase [EC:2.7.1.40]	466	143	100	243	21	6
**M13tol**	M_contig_405	9148	10311	-	**22.3**	K00927	PGK, pgk; phosphoglycerate kinase [EC:2.7.2.3]	670	308	136	281	30	5
**M13tol**	M_contig_336	23791	24240	+	**21.3**	K02939	RP-L9, MRPL9, rplI; large subunit ribosomal protein L9	553	123	120	267	26	4
**M13tol**	M_contig_630	94	615	+	**21.0**	K02860	rimM; 16S rRNA processing protein RimM	704	70	173	258	34	5
**M13tol**	M_contig_1871	11480	11758	+	**20.6**	K02965	RP-S19, rpsS; small subunit ribosomal protein S19	2157	824	403	802	105	7
**M13tol**	M_contig_2079	59673	60062	-	**20.1**	K02116	atpI; ATP synthase protein I	300	165	110	103	15	6
**M13tol**	M_contig_71	10759	11853	+	**18.4**	K00052	leuB; 3-isopropylmalate dehydrogenase [EC:1.1.1.85]	196	76	46	67	11	5
**M13tol**	M_contig_1871	16391	16744	+	**17.4**	K02881	RP-L18, MRPL18, rplR; large subunit ribosomal protein L18	1145	260	228	458	66	6
**M13tol**	M_contig_2079	58885	59664	-	**17.1**	K02108	ATPF0A, atpB; F-type H+-transporting ATPase subunit a [EC:3.6.3.14]	384	248	163	136	22	7
**M13tol**	M_contig_1871	8725	9036	+	**16.9**	K02946	RP-S10, MRPS10, rpsJ; small subunit ribosomal protein S10	1265	560	322	599	75	6
**M13tol**	M_contig_976	48366	50648	-	**16.1**	K00656	pflD; formate C-acetyltransferase [EC:2.3.1.54]	867	528	396	243	54	5
**M13tol**	M_contig_1379	10397	11620	+	**15.3**	K09458	fabF; 3-oxoacyl-[acyl-carrier-protein] synthase II [EC:2.3.1.179]	219	68	71	79	14	4
**M13tol**	M_contig_1308	26045	27040	-	**15.2**	K00134	GAPDH, gapA; glyceraldehyde 3-phosphate dehydrogenase	1960	766	553	762	129	6
**M13tol**	M_contig_980	78134	82768	-	**15.1**	K00265	gltB; glutamate synthase (NADPH/NADH) large chain	247	89	78	94	16	5
**M13tol**	M_contig_1871	9059	9697	+	**14.5**	K02906	RP-L3, MRPL3, rplC; large subunit ribosomal protein L3	1328	374	299	556	91	7
**M13tol**	M_contig_976	53744	54868	+	**14.3**	K00133	asd; aspartate-semialdehyde dehydrogenase	149	98	35	47	10	5
**M13tol**	M_contig_2302	5149	5406	+	**14.2**	K02899	RP-L27, MRPL27, rpmA; large subunit ribosomal protein L27	321	107	142	219	23	4
**M13tol**	M_contig_1843	99	2201	-	**13.9**	K02355	fusA, GFM, EFG; elongation factor G	928	258	257	416	67	6
**M13tol**	M_contig_1843	13574	13939	-	**13.9**	K02935	RP-L7, MRPL12, rplL; large subunit ribosomal protein L7/L12	665	176	160	295	48	6
**M13tol**	M_contig_71	11856	13253	+	**13.9**	K01703	leuC; 3-isopropylmalate/(R)-2-methylmalate dehydratase large subunit	174	92	49	69	13	5
**M13tol**	M_contig_1871	13853	14221	+	**13.7**	K02874	RP-L14, MRPL14, rplN; large subunit ribosomal protein L14	1734	498	408	712	127	6
**M13tol**	M_contig_2302	4820	5131	+	**13.6**	K02888	RP-L21, MRPL21, rplU; large subunit ribosomal protein L21	1017	236	271	477	75	5
**M13tol**	M_contig_2079	4266	5189	+	**13.6**	K00826	ilvE; branched-chain amino acid aminotransferase [EC:2.6.1.42]	172	60	32	68	13	5
**OCS H3lac/M2lac/L12lac**													
**H3lac**	H_contig_1010	24726	25034	-	**154.8**	K02946	RP-S10, MRPS10, rpsJ; small subunit ribosomal protein S10	1370	467	37	1868	11	36
**H3lac**	H_contig_1010	20466	20894	-	**146.1**	K02878	RP-L16, MRPL16, rplP; large subunit ribosomal protein L16	1863	830	118	2347	15	38
**H3lac**	H_contig_1010	20894	21547	-	**136.6**	K02982	RP-S3, rpsC; small subunit ribosomal protein S3	1428	593	89	1894	12	37
**H3lac**	H_contig_1010	19022	19327	-	**128.5**	K02895	RP-L24, MRPL24, rplX; large subunit ribosomal protein L24	1149	654	119	1612	11	37
**H3lac**	H_contig_1010	10615	11550	-	**127.7**	K03040	rpoA; DNA-directed RNA polymerase subunit alpha [EC:2.7.7.6]	1493	592	86	1893	14	40
**H3lac**	H_contig_1089	25374	25685	-	**125.0**	K02888	RP-L21, MRPL21, rplU; large subunit ribosomal protein L21	1096	308	28	1230	10	38
**H3lac**	H_contig_1010	24088	24711	-	**119.9**	K02906	RP-L3, MRPL3, rplC; large subunit ribosomal protein L3	1314	526	46	1640	13	40
**H3lac**	H_contig_1010	21924	22202	-	**111.6**	K02965	RP-S19, rpsS; small subunit ribosomal protein S19	1642	703	127	2096	18	35
**H3lac**	H_contig_1010	23151	23438	-	**96.2**	K02892	RP-L23, MRPL23, rplW; large subunit ribosomal protein L23	1371	652	56	1805	17	38
**H3lac**	H_contig_417	44484	44951	+	**95.2**	K02992	RP-S7, MRPS7, rpsG; small subunit ribosomal protein S7	834	282	18	1063	10	37
**H3lac**	H_contig_1010	15122	15571	-	**93.1**	K02876	RP-L15, MRPL15, rplO; large subunit ribosomal protein L15	1131	463	166	1365	14	32
**H3lac**	H_contig_1010	15882	16376	-	**82.5**	K02988	RP-S5, MRPS5, rpsE; small subunit ribosomal protein S5	1595	661	162	1816	23	36
**H3lac**	H_contig_63	73952	74461	+	**82.1**	K02109	ATPF0B, atpF; F-type H+-transporting ATPase subunit b [EC:3.6.3.14]	881	767	163	1132	13	34
**H3lac**	H_contig_417	45235	47310	+	**78.6**	K02355	fusA, GFM, EFG; elongation factor G	673	285	23	856	10	39
**H3lac**	H_contig_210	78621	79316	+	**78.1**	K01834	PGAM, gpmA; 2,3-bisphosphoglycerate-dependent phosphoglycerate mutase	768	329	14	984	12	37
**H3lac**	H_contig_1010	18457	18999	-	**74.0**	K02931	RP-L5, MRPL5, rplE; large subunit ribosomal protein L5	1118	466	97	1506	18	39
**H3lac**	H_contig_1010	18156	18464	+	**72.5**	-		1283	544	155	1653	21	34
**H3lac**	H_contig_208	16165	16674	-	**68.1**	K03111	ssb; single-strand DNA-binding protein	1460	459	67	1456	26	34
**H3lac**	H_contig_1010	12002	12367	-	**67.6**	K02952	RP-S13, rpsM; small subunit ribosomal protein S13	1767	837	112	2275	31	36
**H3lac**	H_contig_1010	22303	23136	-	**67.3**	K02886	RP-L2, MRPL2, rplB; large subunit ribosomal protein L2	772	359	47	1115	14	38
**H3lac**	H_contig_352	11252	12043	-	**62.2**	K02795	PTS-Man-EIIC, manY; PTS system, mannose-specific IIC component	537	937	148	2185	10	39
**H3lac**	H_contig_1010	20270	20476	-	**61.8**	K02904	RP-L29, rpmC; large subunit ribosomal protein L29	1226	668	83	1683	24	37
**H3lac**	H_contig_208	15872	16117	-	**61.7**	K02963	RP-S18, MRPS18, rpsR; small subunit ribosomal protein S18	686	227	51	600	13	34
**H3lac**	H_contig_1010	21560	21904	-	**59.8**	K02890	RP-L22, MRPL22, rplV; large subunit ribosomal protein L22	1659	748	80	1926	33	39
**H3lac**	H_contig_208	26120	28228	-	**57.4**	K01915	glnA; glutamine synthetase [EC:6.3.1.2]	894	650	26	1322	19	40
**H3lac**	H_contig_481	31477	31977	+	**57.4**	K02864	RP-L10, MRPL10, rplJ; large subunit ribosomal protein L10	2038	797	101	2393	42	27
**H3lac**	H_contig_313	7376	8257	+	**54.1**	K01624	FBA, fbaA; fructose-bisphosphate aldolase, class II	1845	1347	108	2516	41	36
**H3lac**	H_contig_1010	16397	16744	-	**53.0**	K02881	RP-L18, MRPL18, rplR; large subunit ribosomal protein L18	1042	409	135	1358	23	37
**H3lac**	H_contig_352	10416	11240	-	**52.6**	K02796	PTS-Man-EIID, manZ; PTS system, mannose-specific IID component	524	824	272	2165	12	40
**H3lac**	H_contig_63	73229	73939	+	**52.1**	K02108	ATPF0A, atpB; F-type H+-transporting ATPase subunit a	701	608	110	845	16	33

Supplementary Table S17. *Continued*

Bin-genom ID	Contig ID	ORF begin	ORF end		Expression dynamics (SP/AcPro)	KO	KO description<br>[Yellow highlight: Fermentation-related metabolic genes]<br>[Blue letter: Ribosomal proteins]	Gene expression level ($mRNA_{RPKM}$)					Ave DNA_{RPKM}
								Con1 SP	Con2 SP+	Con3 OC_{short}	Con4 OC_{long}	Con5 AcPro	
M2lac	M_contig_349	20446	20874	-	**266.7**	K02878	RP-L16, MRPL16, rplP; large subunit ribosomal protein L16	3863	1805	918	2727	14	31
M2lac	M_contig_349	15862	16356	-	**206.5**	K02988	RP-S5, MRPS5, rpsE; small subunit ribosomal protein S5	2592	1076	703	1890	13	31
M2lac	M_contig_349	21904	22182	-	**174.8**	K02965	RP-S19, rpsS; small subunit ribosomal protein S19	3894	1288	752	2700	22	27
M2lac	M_contig_349	19967	20227	-	**153.2**	K02961	RP-S17, MRPS17, rpsQ; small subunit ribosomal protein S17	3648	1331	785	2624	24	29
M2lac	M_contig_349	24068	24691	-	**143.4**	K02906	RP-L3, MRPL3, rplC; large subunit ribosomal protein L3	2855	1192	677	2139	20	33
M2lac	M_contig_349	18136	18444	+	**141.6**	-		2848	929	509	1602	20	28
M2lac	M_contig_137	31526	32026	+	**139.5**	K02864	RP-L10, MRPL10, rplJ; large subunit ribosomal protein L10	3461	1262	774	2487	25	23
M2lac	M_contig_349	19575	19943	-	**135.1**	K02874	RP-L14, MRPL14, rplN; large subunit ribosomal protein L14	2275	990	650	1670	17	30
M2lac	M_contig_349	17583	17981	-	**122.7**	K02994	RP-S8, rpsH; small subunit ribosomal protein S8	1911	681	418	1203	16	34
M2lac	M_contig_129	68780	69964	-	**117.5**	K01999	livK; branched-chain amino acid transport system substrate-binding protein	1232	785	508	1166	10	35
M2lac	M_contig_628	4003	4416	-	**113.5**	K02950	RP-S12, MRPS12, rpsL; small subunit ribosomal protein S12	1703	771	426	1311	15	30
M2lac	M_contig_349	10595	11530	-	**112.5**	K03040	rpoA; DNA-directed RNA polymerase subunit alpha	2987	1211	725	1921	27	34
M2lac	M_contig_272	9719	10495	+	**111.5**	K02967	RP-S2, MRPS2, rpsB; small subunit ribosomal protein S2	1784	644	352	1151	16	33
M2lac	M_contig_364	136452	137333	-	**110.5**	K01624	FBA, fbaA; fructose-bisphosphate aldolase, class I	3113	1940	1400	3424	28	30
M2lac	M_contig_349	22283	23116	-	**110.4**	K02886	RP-L2, MRPL2, rplB; large subunit ribosomal protein L2	1645	646	320	1072	15	30
M2lac	M_contig_272	70130	70441	+	**108.1**	K02888	RP-L21, MRPL21, rplU; large subunit ribosomal protein L21	2152	798	474	1603	20	30
M2lac	M_contig_349	16926	17462	-	**102.7**	K02933	RP-L6, MRPL6, rplF; large subunit ribosomal protein L6	2376	965	553	1788	23	31
M2lac	M_contig_349	16377	16724	-	**97.6**	K02881	RP-L18, MRPL18, rplR; large subunit ribosomal protein L18	1743	780	393	1426	18	33
M2lac	M_contig_349	15672	15851	-	**88.8**	K02907	RP-L30, MRPL30, rpmD; large subunit ribosomal protein L30	3064	1331	653	1954	35	27
M2lac	M_contig_197	50041	51018	-	**73.3**	K00016	LDH, ldh; L-lactate dehydrogenase [EC:1.1.1.27]	1398	757	497	1493	19	24
M2lac	M_contig_349	23418	24044	-	**67.8**	K02926	RP-L4, MRPL4, rplD; large subunit ribosomal protein L4	2016	726	461	1496	30	32
M2lac	M_contig_628	3515	3982	-	**62.1**	K02992	RP-S7, MRPS7, rpsG; small subunit ribosomal protein S7	1650	707	407	1173	27	29
M2lac	M_contig_349	19002	19307	-	**62.1**	K02895	RP-L24, MRPL24, rplX; large subunit ribosomal protein L24	2524	1136	607	1932	41	32
M2lac	M_contig_256	55293	55652	-	**61.8**	K02887	RP-L20, MRPL20, rplT; large subunit ribosomal protein L20	1066	313	190	655	17	29
M2lac	M_contig_256	34978	36219	-	**61.3**	K02945	RP-S1, rpsA; small subunit ribosomal protein S1	1533	579	389	1297	25	27
M2lac	M_contig_349	12510	12728	-	**57.9**	K02518	infA; translation initiation factor IF-1	3285	937	667	2056	57	26
M2lac	M_contig_256	5440	5787	-	**56.5**	K02884	RP-L19, MRPL19, rplS; large subunit ribosomal protein L19	2019	794	356	1269	36	23
M2lac	M_contig_193	113722	114417	+	**56.3**	K01834	PGAM, gpmA; 2,3-bisphosphoglycerate-dependent phosphoglycerate mutase	1509	578	342	1631	27	30
M2lac	M_contig_137	78117	79124	-	**53.7**	K00850	pfkA, PFK; 6-phosphofructokinase 1 [EC:2.7.1.11]	1325	644	498	1387	25	23
M2lac	M_contig_197	11705	12757	+	**53.6**	K07335	bmpA, bmpB, tmpC; basic membrane protein A and related proteins	950	641	671	962	18	26
L12lac	L_contig_916	20430	20858	-	**39.6**	K02878	RP-L16, MRPL16, rplP; large subunit ribosomal protein L16	556	555	559	501	14	10
L12lac	L_contig_3847	86502	87002	-	**37.7**	K02864	RP-L10, MRPL10, rplJ; large subunit ribosomal protein L10	454	520	359	369	12	6
L12lac	L_contig_3853	20044	20844	+	**37.2**	K02358	tuf, TUFM; elongation factor Tu	560	659	494	602	15	9
L12lac	L_contig_917	16864	17745	-	**30.6**	K01624	FBA, fbaA; fructose-bisphosphate aldolase, class II	419	557	428	383	14	8
L12lac	L_contig_916	15846	16340	-	**25.4**	K02988	RP-S5, MRPS5, rpsE; small subunit ribosomal protein S5	310	391	351	390	12	9
L12lac	L_contig_916	23115	23402	-	**25.4**	K02892	RP-L23, MRPL23, rplW; large subunit ribosomal protein L23	533	516	333	443	21	9
L12lac	L_contig_1525	17388	17678	+	**24.5**	-		254	102	124	208	10	8
L12lac	L_contig_3847	85994	86359	-	**22.6**	K02935	RP-L7, MRPL12, rplL; large subunit ribosomal protein L7/L12	373	447	328	317	16	8
L12lac	L_contig_916	19951	20211	-	**22.6**	K02961	RP-S17, MRPS17, rpsQ; small subunit ribosomal protein S17	261	513	413	425	12	11
L12lac	L_contig_3257	38036	39448	-	**15.9**	K02112	ATPF1B, atpD; F-type H+-transporting ATPase subunit beta	237	321	284	176	15	8
L12lac	L_contig_1917	19529	19774	+	**15.1**	K02963	RP-S18, MRPS18, rpsR; small subunit ribosomal protein S18	185	302	146	150	12	9
L12lac	L_contig_1465	50813	52888	+	**14.1**	K02355	fusA, GFM, EFG; elongation factor G	164	233	167	157	12	9
L12lac	L_contig_552	6135	8081	+	**13.8**	K02809	PTS-Scr-EIIB, scrA; PTS system, sucrose-specific IIB component	213	409	1247	469	15	7
L12lac	L_contig_2066	27658	28983	-	**13.0**	K01689	ENO, eno; enolase [EC:4.2.1.11]	531	667	606	490	41	10
L12lac	L_contig_916	18421	18963	-	**11.9**	K02931	RP-L5, MRPL5, rplE; large subunit ribosomal protein L5	199	301	265	238	17	11
L12lac	L_contig_916	21888	22166	-	**11.3**	K02965	RP-S19, rpsS; small subunit ribosomal protein S19	367	693	430	542	32	9
L12lac	L_contig_2462	9445	9735	+	**11.3**	K02899	RP-L27, MRPL27, rpmA; large subunit ribosomal protein L27	117	153	124	156	10	9
L12lac	L_contig_916	1580	2650	-	**9.7**	K00053	ilvC; ketol-acid reductoisomerase [EC:1.1.1.86]	329	389	291	251	34	10
L12lac	L_contig_1525	2324	2605	-	**9.4**	K07729	putative transcriptional regulator	101	79	106	113	11	8
L12lac	L_contig_505	2	322	-	**8.0**	K02884	RP-L19, MRPL19, rplS; large subunit ribosomal protein L19	301	185	336	209	38	8
L12lac	L_contig_1104	1496	2509	+	**7.7**	K00134	GAPDH, gapA; glyceraldehyde 3-phosphate dehydrogenase	616	814	1094	747	80	10
L12lac	L_contig_916	15656	15835	-	**7.5**	K02907	RP-L30, MRPL30, rpmD; large subunit ribosomal protein L30	379	578	300	401	50	8
L12lac	L_contig_2489	24434	25024	+	**7.5**	K01704	leuD; 3-isopropylmalate/(R)-2-methylmalate dehydratase small subunit	77	76	30	43	10	9
L12lac	L_contig_34436	3	1220	-	**7.3**	-		145	201	221	154	20	11
L12lac	L_contig_623	57146	58342	+	**7.3**	K00927	PGK, pgk; phosphoglycerate kinase [EC:2.7.2.3]	147	267	255	211	20	10
L12lac	L_contig_1761	13635	14759	-	**7.1**	-		76	99	336	149	11	9
L12lac	L_contig_3101	4020	5651	+	**6.5**	K04077	groEL, HSPD1; chaperonin GroEL	84	73	114	89	13	11
L12lac	L_contig_1179	123806	124747	+	**6.3**	K02796	PTS-Man-EIID, manZ; PTS system, mannose-specific IID component	121	221	363	157	19	8
L12lac	L_contig_3257	37595	38014	-	**5.7**	K02114	ATPF1E, atpC; F-type H+-transporting ATPase subunit epsilon	203	301	314	184	36	8
L12lac	L_contig_1104	17899	19116	+	**5.3**	-		79	98	133	99	15	10

Supplementary Table S17. *Continued*

Bin-genom ID	Contig ID	ORF begin	ORF end		Expression dynamics (SP/AcPro)	KO	KO description<br>[Yellow highlight: Fermentation-related metabolic genes]<br>[Blue letter: Ribosomal proteins]	Gene expression level ($mRNA_{RPKM}$) Con1 SP	Con2 SP+	Con3 OC_{short}	Con4 OC_{long}	Con5 AcPro	Ave DNA_{RPKM}
**OCS H6fir/M5fir**													
**H6fir**	H_contig_598	12765	13136	+	**109.7**	K02952	RP-S13, rpsM; small subunit ribosomal protein S13	1013	803	896	784	11	12
**H6fir**	H_contig_1053	101257	101487	-	**90.0**	K06960	K06960	892	850	725	914	12	11
**H6fir**	H_contig_598	5465	5833	+	**79.4**	K02874	RP-L14, MRPL14, rplN; large subunit ribosomal protein L14	986	697	851	970	15	13
**H6fir**	H_contig_598	8373	8873	+	**77.7**	K02988	RP-S5, MRPS5, rpsE; small subunit ribosomal protein S5	1065	758	890	936	16	13
**H6fir**	H_contig_2987	2	184	-	**76.5**	K02358	tuf, TUFM; elongation factor Tu	958	564	758	951	15	10
**H6fir**	H_contig_2987	2337	2807	-	**74.2**	K02992	RP-S7, MRPS7, rpsG; small subunit ribosomal protein S7	1444	1084	1155	1724	23	13
**H6fir**	H_contig_1257	9406	9708	-	**72.8**	K10858	PMS2; DNA mismatch repair protein PMS2	1375	1173	992	1979	23	14
**H6fir**	H_contig_598	11451	12197	+	**68.8**	K01265	map; methionyl aminopeptidase [EC:3.4.11.18]	949	712	829	775	16	14
**H6fir**	H_contig_598	6770	6955	+	**67.3**	K02954	RP-S14, MRPS14, rpsN; small subunit ribosomal protein S14	829	670	777	667	15	12
**H6fir**	H_contig_2987	224	2311	-	**63.2**	K02355	fusA, GFM, EFG; elongation factor G	1595	1246	1294	1772	30	14
**H6fir**	H_contig_2987	14068	14502	-	**62.0**	K02867	RP-L11, MRPL11, rplK; large subunit ribosomal protein L11	816	698	680	671	16	13
**H6fir**	H_contig_598	2278	3105	+	**57.8**	K02886	RP-L2, MRPL2, rplB; large subunit ribosomal protein L2	960	643	822	1127	20	12
**H6fir**	H_contig_598	14266	15219	+	**57.7**	K03040	rpoA; DNA-directed RNA polymerase subunit alpha	900	719	843	757	19	14
**H6fir**	H_contig_598	6983	7381	+	**57.7**	K02994	RP-S8, rpsH; small subunit ribosomal protein S8	1324	995	1258	1252	27	13
**H6fir**	H_contig_3257	4242	4526	-	**57.7**	K00960	DNA-directed RNA polymerase [EC:2.7.7.6]	1159	1055	980	1244	24	14
**H6fir**	H_contig_598	3174	3410	+	**56.8**	K02965	RP-S19, rpsS; small subunit ribosomal protein S19	1371	1119	1341	1340	29	12
**H6fir**	H_contig_598	19592	19984	+	**55.6**	K02996	RP-S9, MRPS9, rpsI; small subunit ribosomal protein S9	648	544	456	697	14	12
**H6fir**	H_contig_598	1335	1961	+	**53.3**	K02926	RP-L4, MRPL4, rplD; large subunit ribosomal protein L4	973	719	811	966	22	13
**H6fir**	H_contig_598	7984	8352	+	**52.6**	K02881	RP-L18, MRPL18, rplR; large subunit ribosomal protein L18	1143	843	924	952	26	12
**H6fir**	H_contig_598	19130	19570	+	**52.6**	K02871	RP-L13, MRPL13, rplM; large subunit ribosomal protein L13	683	519	522	647	15	15
**H6fir**	H_contig_2987	12164	12532	-	**52.3**	K02935	RP-L7, MRPL12, rplL; large subunit ribosomal protein L7/L12	649	766	783	406	15	15
**H6fir**	H_contig_598	33223	34296	+	**52.0**	K00826	ilvE; branched-chain amino acid aminotransferase [EC:2.6.1.42]	499	475	482	621	11	14
**H6fir**	H_contig_598	13159	13551	+	**50.9**	K02948	RP-S11, MRPS11, rpsK; small subunit ribosomal protein S11	1336	1041	1296	1050	31	11
**H6fir**	H_contig_598	3	158	+	**50.3**	K02358	tuf, TUFM; elongation factor Tu	1107	980	1105	1343	26	10
**H6fir**	H_contig_1254	27702	28013	+	**50.0**	K02888	RP-L21, MRPL21, rplU; large subunit ribosomal protein L21	917	688	772	909	22	12
**H6fir**	H_contig_598	660	1301	+	**49.9**	K02906	RP-L3, MRPL3, rplC; large subunit ribosomal protein L3	1157	929	1046	1278	28	12
**H6fir**	H_contig_598	7428	7958	+	**49.4**	K02933	RP-L6, MRPL6, rplF; large subunit ribosomal protein L6	639	462	559	549	15	13
**H6fir**	H_contig_1576	1096	2847	-	**48.0**	K00239	sdhA; succinate dehydrogenase flavoprotein subunit	659	927	1054	832	16	14
**H6fir**	H_contig_598	3462	3794	+	**47.9**	K02890	RP-L22, MRPL22, rplV; large subunit ribosomal protein L22	989	824	928	997	25	14
**H6fir**	H_contig_598	3817	4479	+	**45.9**	K02982	RP-S3, rpsC; small subunit ribosomal protein S3	1267	988	1053	1118	33	13
**M5fir**	M_contig_1319	42677	43120	-	**97.5**	K02878	RP-L16, MRPL16, rplP; large subunit ribosomal protein L16	1037	750	1007	225	11	8
**M5fir**	M_contig_1319	41769	42137	-	**78.7**	K02874	RP-L14, MRPL14, rplN; large subunit ribosomal protein L14	1006	672	862	191	13	7
**M5fir**	M_contig_899	105	407	-	**71.8**	-		2238	2307	1540	908	31	10
**M5fir**	M_contig_298	12739	13122	+	**55.6**	K02950	RP-S12, MRPS12, rpsL; small subunit ribosomal protein S12	1367	1379	1265	464	25	7
**M5fir**	M_contig_1319	27618	28010	-	**53.2**	K02996	RP-S9, MRPS9, rpsI; small subunit ribosomal protein S9	639	558	514	389	12	9
**M5fir**	M_contig_588	228772	229644	-	**52.8**	K02528	ksgA; 16S rRNA (adenine1518-N6/adenine1519-N6)-dimethyltransferase	571	113	85	131	11	9
**M5fir**	M_contig_1319	44192	44428	-	**52.3**	K02965	RP-S19, rpsS; small subunit ribosomal protein S19	1041	766	890	248	20	7
**M5fir**	M_contig_1319	46959	47270	-	**49.2**	K02946	RP-S10, MRPS10, rpsJ; small subunit ribosomal protein S10	1490	1033	1319	391	30	9
**M5fir**	M_contig_1319	38071	38511	-	**44.7**	K02876	RP-L15, MRPL15, rplO; large subunit ribosomal protein L15	479	367	448	77	11	7
**M5fir**	M_contig_1318	63973	65016	-	**40.6**	K03522	fixB, etfA; electron transfer flavoprotein alpha subunit	735	1774	1937	396	18	8
**M5fir**	M_contig_1319	44497	45324	-	**40.3**	K02886	RP-L2, MRPL2, rplB; large subunit ribosomal protein L2	918	687	786	222	23	7
**M5fir**	M_contig_1319	42484	42687	-	**40.0**	K02904	RP-L29, rpmC; large subunit ribosomal protein L29	927	875	969	224	23	8
**M5fir**	M_contig_298	13651	15738	+	**38.5**	K02355	fusA, GFM, EFG; elongation factor G	1478	1414	1471	407	38	9
**M5fir**	M_contig_1319	43808	44140	-	**37.4**	K02890	RP-L22, MRPL22, rplV; large subunit ribosomal protein L22	1062	513	772	194	28	8
**M5fir**	M_contig_1318	62552	63958	-	**35.4**	K00104	glcD; glycolate oxidase [EC:1.1.3.15]	713	1483	1596	298	20	8
**M5fir**	M_contig_1319	34466	34837	-	**34.6**	K02952	RP-S13, rpsM; small subunit ribosomal protein S13	879	651	899	178	25	8
**M5fir**	M_contig_1318	49199	49468	+	**33.4**	K02968	RP-S20, rpsT; small subunit ribosomal protein S20	585	824	842	338	17	8
**M5fir**	M_contig_146	124164	125522	-	**32.2**	K03545	tig; trigger factor	335	287	347	161	10	9
**M5fir**	M_contig_1319	47444	47581	-	**32.0**	K02358	tuf, TUFM; elongation factor Tu	1096	943	980	245	34	6
**M5fir**	M_contig_1318	65045	65836	-	**32.0**	K03521	fixA, etfB; electron transfer flavoprotein beta subunit	573	1316	1574	275	18	8
**M5fir**	M_contig_1319	43123	43785	-	**30.9**	K02982	RP-S3, rpsC; small subunit ribosomal protein S3	1321	910	1062	293	43	8
**M5fir**	M_contig_1318	18955	19362	-	**30.6**	-		354	456	492	105	12	7
**M5fir**	M_contig_146	142446	143003	-	**30.1**	K01676	fumA, fumB; fumarate hydratase, class I	510	840	919	353	17	9
**M5fir**	M_contig_189	22195	22638	-	**30.1**	K00962	pnp, PNPT1; polyribonucleotide nucleotidyltransferase	321	303	338	205	11	8
**M5fir**	M_contig_189	86022	86306	-	**29.8**	K00960	DNA-directed RNA polymerase [EC:2.7.7.6]	1482	1826	1423	754	50	11
**M5fir**	M_contig_1319	40848	41387	-	**28.9**	K02931	RP-L5, MRPL5, rplE; large subunit ribosomal protein L5	758	712	864	196	26	8
**M5fir**	M_contig_1319	36814	38070	-	**28.2**	K03076	secY; preprotein translocase subunit SecY	953	841	956	188	34	8
**M5fir**	M_contig_751	118990	119748	-	**26.1**	K02500	hisF; cyclase [EC:4.1.3.-]	325	155	137	23	12	8
**M5fir**	M_contig_1319	45641	46267	-	**25.2**	K02926	RP-L4, MRPL4, rplD; large subunit ribosomal protein L4	947	789	780	192	38	8
**M5fir**	M_contig_298	1461	1895	+	**25.0**	K02867	RP-L11, MRPL11, rplK; large subunit ribosomal protein L11	542	713	755	284	22	9

Supplementary Table S18. Summary of *c*- type cytochrome families coded in *Geobacteraceae* microbes.

CytC family ID[a]		cytC family description; KO/COG<br>*Red letter, 14 key MH-cytC genes in this manuscript (also used for wCCA diagram)<br>*Purple letter, cytC genes used for wCCA diagram in this manuscript<br>*Blue letter, newly-named protein ID in this manuscript	Estimated location	No of CXXCH domain	Locus tag in *Geobacter sulfurreducens*
**2568**	**macA**	cytochrome c peroxidase [EC:1.11.1.5]; K00428/COG1858	IM	2	GSU0466
**1467**	**cbcL**	nonaheme cytochrome c (inner membrane)	IM	9	GSU0274
**49**	**ppcA**	cytochrome c3	PP	3	GSU0364/GSU0612
**2890**	**ppcI**	putative cytochrome c3	PP	3	
**5994**	**ppcK**	putative cytochrome c3	PP	3	
**1653**	**omcB**	polyheme membrane-associated cytochrome c (Lipo protein), part of oma/omb/omcB conduit	OM	12	GSU2731/GSU2737
**51**	**omaB**	polyheme membrane-associated cytochrome c, part of oma/omb/omcB conduit	OM/PP	8	GSU2732/GSU2738
**2177**	**omcE**	extracellular multi-heme cytochrome c	OM	4	GSU0618
**64**	**omcS**	extracellular multi-heme cytochrome c	OM	6	GSU0701/GSU2503/GSU2504
**2504**	**omcX**	outer membrane multi-heme cytochrome c (likely periplasmic side)	OM	11	GSU0670
**2307**	**omcZ**	secreted multi-heme cytochrome c	OM	7	GSU1334/GSU2076
**1253**	**omcY**	outer membrane multi-heme cytochrome c	OM	7	GSU2201
**1292**	**omcQ**	part of Cbc5 complex, =CbcC	OM	12	GSU0592
**1964**	**cbcA**	c-type, 7-heme binding sites, Calcium binding site (EF hand), part of Cbc5 complex	OM/PP	6	GSU0594
**2830**	**imcG**	cytochrome c peroxidase COG1858	IM	2	GSU1538
**2899**	**cyc1**	ubiquinol-cytochrome c reductase cytochrome c1 subunit; K00413/COG2857	IM	2	GSU3334
**2370**	**petJ**	cytochrome c6; K08906/COG2010	IM/PP	1	GSU2743
**3139**	**ppcL**	periplasmic cytochrome c552 (nitrite reductase); COG3303	PP	3	
**3808**	**ppcJ**	tri-heme cytochrome c	PP	3	
**2686**	**cytT**	pfam13435, poly-heme cytochrome c554	PP	4	GSU2299
**2992**	**extA**	predicted to be inner face of outer membrane, part of ExtABCD conduit	OM/PP	11	GSU2645
**1768**	**omcV**	outer membrane polyheme cytochrome c	OM	12	GSU1996
**2898**	**omcA**	TIGR03507, decaheme c-type cytochrome, OmcA/MtrC family (Lipo protein)	OM	10	
**3550**	**dmsE**	TIGR03508: decahem_SO decaheme c-type cytochrome, DmsE family, partial	OM	6	
**266**	**cbcM**	part of Cbc6 complex	OM	12	GSU2935
**267**	**cbcN**	part of Cbc6 complex	OM/PP	10	GSU2934
**271**	**cbcR**	Cytochrome c2 (COG3474)	-	2	GSU2930
**600**	**cbcS**	part of Cbc4 complex (cbcSTS)	OM	4	GSU0068
**2461**	**cbcX**	part of Cbc3 complex (CbcXWY)	-	5	GSU1648
**4316**	**ccoO**	cytochrome c oxidase cbb3-type subunit II; K00405/COG2993	IM	2	
**6764**	**ccoO**	cytochrome c oxidase cbb3-type subunit II; K00405/COG2993	IM	2	
**1899**	**ccoP**	cytochrome c oxidase cbb3-type subunit III; K00406/COG2010	IM	1	GSU2513
Gmet_1088	**ccoP**	cytochrome c oxidase cbb3-type subunit III; K00406/COG2010	IM	1	
**2564**	**coxB**	cytochrome c oxidase subunit II [EC:1.9.3.1]; K02275/COG1622	IM	1	GSU0222
**3257**	**cycC**	Cytochrome c; COG3258	-	1	GSU1740
**cymA**	**cymA**	cytoplasmic membrane-bound, tetraheme cytochrome c	IM	4	
**1654**	**extCF**	part of ExtABCD conduit	OM	5	GSU2643/GSU2725
**3397**	**extD**	lipoprotein attachment site, part of ExtABCD conduit	OM	6	GSU2642
**3244**	**extG**	part of ExtEFG conduit	OM/PP	3	GSU2724
**71**	**extK**	Outer membrane cytochrome, part of extHIJKL conduit	OM	5	GSU2937
**2452**	**extQ**	Outer membrane partner to extR	OM	23	GSU2495
**2451**	**extR**	part of ExtQR conduit	PP?	16	GSU2494
**3236**	**extT**	part of ExtTUVW conduit	PP?	4	GSU3223
**3233**	**extW**	part of ExtTUVW conduit	OM	7	GSU3226
**2277**	**frdA**	fumarate reductase flavoprotein subunit [EC:1.3.99.1]; K00244/COG1053	IM	4	
**2555**	**ftr**	high-affinity iron transporter; K07243/COG2010	-	1	
**1950**	**hsc**	Cytochrome c peroxidase; COG1858	IM	2	
**1674**	**imcH**	membrane-bound tetraheme cytochrome c (Nitrate/TMAO reductases); COG3005	IM	4	GSU3259
**4751**	**macA**	cytochrome c peroxidase [EC:1.11.1.5]; K00428/COG1858	IM	2	GSU2813
**mtrA**	**mtrA**	decaheme cytochrome c	OM	12	
**7063**	**napB**	cytochrome c-type protein NapB; K02568/COG3043	-	2	
**2179**	**ctcB**	cytochrome c (Membrane bound?)	IM/OM	6	GSU0616
**7567**	**napB**	cytochrome c-type protein NapB; K02568/COG3043	-	2	
**5750**	**napC**	Nitrate/TMAO reductases, membrane-bound tetraheme cytochrome c subunit; COG3005	IM	6	
**2192**	**ctcA**	tetra/tri-heme cytochrome c (Membrane bound?)	IM/OM	3	GSU3221
**2225**	**ctcC**	cytochrome c (Membrane bound?)	IM/OM	5	GSU2801
**6771**	**norC**	nitric oxide reductase subunit C; K02305/COG02010	-	2	
**1695**	**nrfA**	periplasmic cytochrome c552 (nitrite reductase); COG3303	PP	5	GSU0357
**88**	**nrfA**	periplasmic cytochrome c552 (nitrite reductase); K03385/COG3303	PP	4	GSU3154
**2126**	**nrfH**	cytochrome c nitrite reductase small subunit; K15876/COG3005	PP	4	GSU3155
**4675**	**omcF**		OM	1	GSU2432
**148**	**omcG**	outer membrane multi-heme cytochrome c	OM	14	GSU0702/GSU2882
**23**	**omcH**	outer membrane multi-heme cytochrome c	OM	24	GSU2883/GSU2884/GSU2912
**1679**	**omcI**	outer membrane multi-heme cytochrome c	OM	7	GSU1228
**4623**	**omcK**	outer membrane multi-heme cytochrome c	OM	10	GSU2203
**3250**	**omcM**		OM	6	GSU2294
**47**	**omcN**	high-molecular-weight cytochrome c	OM	27	GSU2898
**3422**	**omcP**	outer membrane multi-heme cytochrome c	OM	3	GSU2913
**3877**	**omcT**		OM	6	
**45**	**pgcA**	periplasmic or secreted multi-heme cytochrome c	OM	2	GSU1761
**3303**	**ppcC**	cytochrome c3	PP	3	GSU0365
**3948**	**ppcD**	cytochrome c3	PP	3	GSU1024
**3353**	**ppcE**	cytochrome c3	PP	3	GSU1760
**2610**	**ctcD**	cytochrome c	-	2	GSU1785
**4800**	**ppcH**	periplasmic cytochrome c	PP	1	GSU3274
**4297**		cytochrome b5	-	1	
**7346**		cytochrome b5	-	1	
**6817**		cytochrome c class I	-	1	
**3237**		cytochrome c family protein	-	12	GSU3218
**3749**		cytochrome c family protein	-	6	
**3803**		cytochrome C family protein	OM	30	
**5965**		cytochrome C family protein	-	10	

Supplementary Table S18. *Continued.*

**7055**	cytochrome C family protein	-	21	
**7900**	cytochrome c, class I	-	1	
**3677**	Cytochrome c, mono- and diheme variants; COG2010	-	2	
**3984**	cytochrome c3	-	1	
**7405**	fatty acid cistrans isomerase	-	1	
**2752**	FOG: PKD repeat; COG3291	PP	6	GSU3233
**31**	high-molecular-weight cytochrome c	OM	16	GSU2899
**1295**	histidine kinase, HAMP region domain protein	-	1	
**1041**	thiol:disulfide interchange protein DsbC	-	1	
**2778**	type II secretion system protein E	-	1	
**243**		-	2	
**256**		-	8	
**727**		-	1	
**1819**		-	8	GSU3137
**1961**		-	1	
**2014**		-	2	GSU2927
**2106**		-	2	GSU3332
**2180**		-	7	GSU0615
**2189**		-	27	GSU2210
**2245**		-	3	GSU1787
**2246**		-	6	GSU1786
**2258**		-	3	GSU0105
**2274**		-	8	
**2287**		OM	10	
**2320**		OM	7	GSU3228
**2448**		-	22	
**2498**		PP	5	
**2554**		-	5	
**2584**		-	3	GSU3214
**2593**		-	3	GSU0533
**2624**		-	23	GSU2887
**2732**		OM	9	
**2751**		-	1	
**2792**		-	1	
**2827**		-	1	
**3111**		-	2	
**3115**		-	1	
**3242**		-	2	GSU2767
**3391**		-	1	GSU2515
**3413**		-	7	
**3428**		-	2	
**3567**		-	1	GSU1397
**3577**		-	1	GSU0935
**3587**		-	3	
**3597**		-	4	
**3695**		-	6	
**3792**		-	3	
**3907**		-	1	
**3938**		-	4	
**3939**		-	1	
**4042**		-	4	
**4211**		-	2	
**4218**		-	6	GSU2501
**4876**		-	13	
**5312**		OM	12	
**5331**		-	6	
**5408**		-	10	
**5415**		-	1	
**5502**		-	6	
**5753**		-	22	
**6017**		-	6	
**6133**		-	23	
**6215**		-	1	
**6310**		-	1	
**6456**		-	4	
**6516**		-	8	
**6720**		-	5	
**6738**		-	8	
**7072**		-	2	
**7436**		-	1	
**7513**		-	4	
**7534**		-	4	
**8012**		-	3	
Gbem_2674		-	5	
GeobDRAFT_1916		-		
GeobDRAFT_2843		-		
Glov_1156		-		
Glov_1291		-		
Glov_2299		-		
Glov_2846		-		
Glov_3546		-		
Gmet_0910		-		
Gmet_1019		-		
Gmet_1647		-		
Gmet_1703		-	4	
Gmet_2156		-	9	
GSU1257		-		
GSU2811		-		
Gura_0672		-		
Gura_0849		-		
Gura_1317		-		

[a] *c-* type cytochrome families assigned by Bulter et al. (BMC Genomics 11, 40, (2010).)

Supplementary Table S19. Potential conductive pili (e-pili) and flp pillus observed in the metagenomes and their gene expression profiles after stimuli application.

Bin-genom ID	Contig ID	ORF begin	ORF end	Strand	type of pillus	Gene expression level ($mRNA_{RPKM}$)					Ave DNA_{RPKM}
						Con1 SP	Con2 SP+	Con3 OC_{short}	Con4 OC_{long}	Con5 AcPro	
**H1geoB**	H_contig_339	4983	5192	+	PilA	2896	2153	1846	163	2178	44
**H1geoB**	H_contig_2577	1068	1292	+	PilA	3	4	0	0	0	51
**H1geoC**	H_contig_394	4885	5091	+	PilA	647	577	532	79	869	37
**H1geoC**	H_contig_2561	1073	1297	+	PilA	0	0	0	0	0	39
**H1geoD**	H_contig_980	542	751	-	PilA	41	41	39	13	203	11
**H1geoD**	H_contig_980	944	1144	-	PilA	3	0	0	0	0	13
**H1geoD**	H_contig_1782	1806	2030	+	PilA	0	0	0	0	0	13
**H2geo**	H_contig_79	50121	50741	+	PilA	619	1051	627	310	1104	36
**H5geo**	H_contig_77	41085	41309	-	PilA	127	166	112	41	34	20
**H7geo**	H_contig_414	11860	12450	+	PilA	314	260	352	777	196	9
**M1geoB**	M_contig_67	4709	4948	-	PilA	3108	1738	726	762	1200	91
**M1geoC**	M_contig_1602	1	204	+	PilA	1752	1951	922	942	1020	33
**M1geoD**	M_contig_143	7458	7754	-	PilA	582	541	90	34	0	17
**M4geo**	M_contig_387	67409	68029	-	PilA	87	264	145	60	396	14
**M7geo**	M_contig_1802	73694	73918	+	PilA	11	35	11	0	116	4
**M17geo**	M_contig_5885	6587	6796	+	PilA	0	8	0	0	0	2
**M17geo**	M_contig_5885	6194	6394	+	PilA	0	0	0	0	0	1
**M17geo**	M_contig_6413	3304	3528	+	PilA	0	0	0	0	0	2
**L1geo**	L_contig_24	565101	565340	-	PilA	1148	812	448	883	1243	66
**L7ppro**	L_contig_166	27743	27967	-	PilA	155	481	229	423	358	7
**L10gmet**	L_contig_302	83537	83749	+	PilA	40	12	9	19	0	4
**H1geoD**	H_contig_336	3165	3362	+	Flp	233	175	103	780	904	13
**H2geo**	H_contig_67	104576	104779	+	Flp	456	397	161	176	779	39
**H6fir**	H_contig_3091	5088	5309	+	Flp	4	0	0	0	0	10
**M3ppro**	M_contig_212	116401	116583	+	Flp	32	91	121	59	137	15
**M4geo**	M_contig_21	104556	104759	+	Flp	58	76	19	27	183	13
**M7geo**	M_contig_2058	45068	45262	+	Flp	0	0	0	0	0	5
**M15fir**	M_contig_1722	19947	20141	-	Flp	0	0	0	0	38	2
**M17geo**	M_contig_3131	8480	8677	+	Flp	16	9	6	0	102	2
